# Bicyclo[2.2.0]hexene derivatives as a proaromatic platform for group transfer and chemical sensing

**DOI:** 10.1038/s41467-021-24054-3

**Published:** 2021-06-17

**Authors:** Bin Wu, Jianing Wang, Xingchen Liu, Rong Zhu

**Affiliations:** Beijing National Laboratory for Molecular Sciences (BNLMS), Key Laboratory of Bioorganic Chemistry and Molecular Engineering of Ministry of Education, College of Chemistry and Molecular Engineering, Peking University, Beijing, China

**Keywords:** Optical materials, Reaction mechanisms, Organic molecules in materials science

## Abstract

Here we report the design, preparation, synthetic utility, and sensing application of a class of proaromatic structures, namely bicyclo[2.2.0]hexene (BCH) derivatives. Building on a valence isomerism concept, they feature modular and easy synthesis as well as high thermal stability, and can be oxidatively activated under mild conditions. New alkyl transfer reactions using BCHs as a radical donor have been developed to showcase the utility of their proaromaticity. Moreover, the redox-triggered valence isomerization of a quinoline-derived BCH led to colorimetric and fluorescent responses toward vapors of electrophilic reagents in solution and solid phase, respectively. This optical response was shown to involve a 1,3-cyclohexadiene structure that possesses an intramolecular charge transfer excited state with interesting aggregation induced emission (AIE) character. Thus, the potential of BCHs has been demonstrated as a versatile platform for the development of new reagents and functional materials.

## Abstract (web summary)

Cyclohexadienes have been widely explored as proaromatic surrogates for group transfer reactions but limited storage stability and difficult accessibility of these compounds limits the application range. Here, the authors present a class of proaromatic bicyclo[2.2.0]hexene derivatives and demonstrate their application in alkyl transfer reactions and sensing applications.

## Introduction

**Fig. 1:**
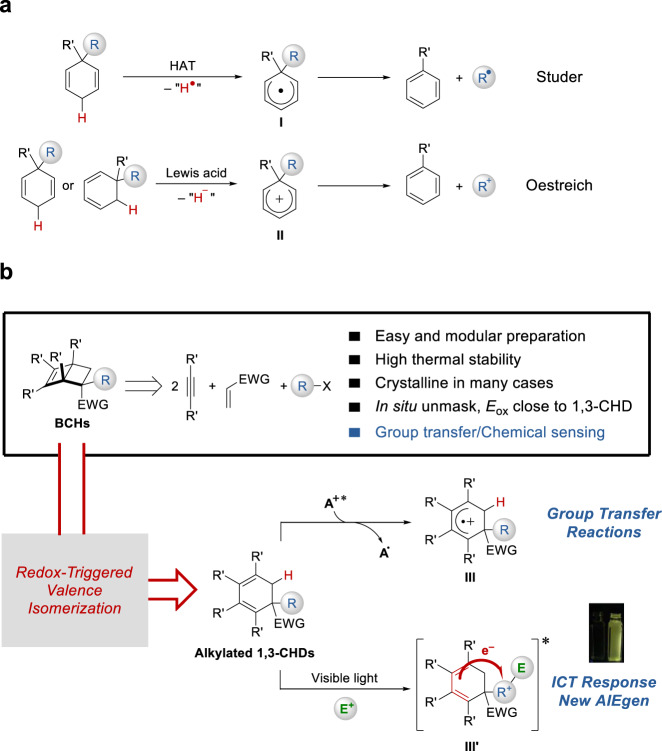
Searching for new benzene-related proaromatic structures. **a** Proaromatic CHDs. **b** Bicyclo[2.2.0]hexene (BCH): proaromaticity via valence isomerism (this work). Utility of BCHs are demonstrated in catalytic group transfer reactions and AIE-active chemical sensing.

Proaromatic structures are of great interest for organic synthesis. Their inherent high-energy nature is often employed to drive reactions forward in an irreversible manner^1–6^. In particular, cyclohexadienes (CHD), a proaromatic form of benzene, have been extensively explored as HX (X = H, CR_3_, SiR_3_, GeR_3_, NR_2_, Br, I, CN) surrogates for group transfer reactions (Fig. 1a)^7–9^. Walton and Cardellini showed that radical chain decomposition of 1,4-CHD derivatives restored the benzene ring with concomitant free radical generation^10,11^. Along this line, Studer reported transfer hydrosilylation and hydroamination of alkenes using 1,4-CHD derivatives as silyl and amino donors, respectively^12–14^. In this scenario, hydrogen abstraction produces a proaromatic radical intermediate **I**, which undergoes subsequent β-scission to release a radical of interest. Alternatively, Oestreich has demonstrated that 1,3-CHD and 1,4-CHD can be activated through hydride abstraction by a Lewis acid to form a Wheland intermediate **II**. Based on this strategy, a broad spectrum of ionic group transfer reactions have been disclosed^15–20^. Despite these elegant examples, CHD derivatives are in general difficult to access^21–24^. Their storage stability is largely limited by the presence of weak allylic C–H bonds (BDE ca. 76 kcal/mol), and reactive dienes in the case of 1,3-CHD. In addition, harsh reaction conditions are often employed for either hydrogen or hydride abstraction triggered activation processes.

Seeking an alternative strategy for proaromatic CHD activation under mild conditions, we proposed to take advantage of the low oxidation potential of alkylated 1,3-CHDs, which allows for activation by single electron transfer (SET) (Fig. 1b)^25–29^. The fate of the resulting radical cation **III** would depend on the nature of R group, which might engage either radical or ionic group transfer through different fragmentation pathways. To circumvent the synthesis and stability issues mentioned above, a valence isomerism strategy was conceived^30^. We envisioned bicyclo[2.2.0]hexene (BCH) derivatives as stable and easily accessible surrogates for 1,3-CHD derivatives that possess a similar oxidation potential.

Herein we report our study on the synthesis and properties of BCH derivatives. Their synthetic utility as proaromatic radical donors is demonstrated in redox-triggered group transfer reactions. The same concept of redox-triggered valence isomerization was applied to a quinoline-derived BCH, where an optical response toward electrophilic reagents was realized. In addition, an interesting aggregation induced emission (AIE) behavior was found in this system (**III**′).

## Results

### Synthesis and characterization of BCHs

**Fig. 2:**
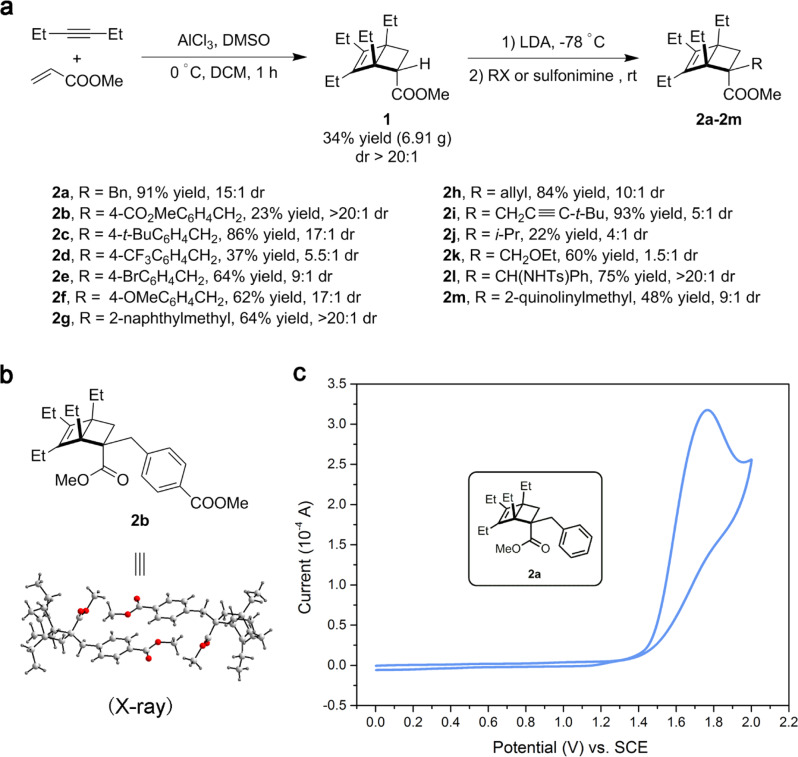
Modular preparation of BCHs and characterizations. **a** Synthetic approach to alkylated BCHs **2a**–**2m**. **b** Determination of the relative stereochemistry of the major diastereomer of **2b**. **c** Cyclic voltammogram of **2a** in MeCN with 0.10 M Bu_4_NPF_6_. The scan rate was 0.10 V/s.

Alkylated BCHs can be readily synthesized from commercially available starting materials in a modular and operationally simple fashion (Fig. 2a). Bicyclo[2.2.0]hexene carboxylic ester **1** was synthesized in multigram scale by the Diels-Alder reaction between an in situ formed cyclobutadiene and methyl acrylate with excellent endo-selectivity^31^. Subsequent base-mediated alkylation of **1** with alkyl halides or sulfonimines afforded BCHs **2a**–**2m** in moderate to good yields with good diastereoselectivity. The relative stereochemistry of the major diastereomer of **2b** was unambiguously determined by X-ray diffraction crystallography (Fig. 2b). It is expected that both diastereomers exhibit nearly identical reactivities in oxidation processes.

As a result of the thermally forbidden ring-opening process as well as the lack of reactive diene structures, **2** typically exhibit high thermal stability in spite of the ring strain. For instance, no detectable decomposition of **2m** was observed when heated in DMF at 110 °C for 5.5 h under a nitrogen atmosphere. The rigid bicyclic structure also provides desired crystallinity in many cases.

A key feature in the design of BCH is that its cyclobutene part should possess a relatively low ionization potential due to the HOMO-raising ring strain and alkyl substituents^32^. To test this hypothesis, we performed cyclic voltammetry experiment with a model BCH **2a**. As shown in Fig. 2c, **2a** displayed an irreversible oxidation wave at *E*_p/2_ = 1.59 V (vs. SCE in MeCN), which corresponds to the oxidation of substituted cyclobutene part into a radical cation that readily opens up to release the strain^33–36^. This indicates that SET between **2** and highly oxidizing photocatalysts, for instance, Fukuzumi acridinium **6**^+^ (excited state *E*_1/2_^red*^ = +2.06 V vs. SCE in MeCN)^37,38^, should be viable, which was validated by Stern-Volmer quenching study (Fig. S6).

### Reaction optimization

Guided by these results, we set out to test the hypothesized proaromaticity of **2** by studying their alkyl transfer reactions with a diazo ester (Table 1). A set of optimal conditions is shown in entry 1. The benzyl transfer reaction between **2a** and di-*tert*-butyl azodicarboxylate (**3a**) proceeded smoothly using **6**^+^ClO_4_^−^ as a photocatalyst under blue light irradiation, affording the hydrobenzylation product **4a** in 81% yield. The use of other solvents such as acetonitrile and dichloroethane in place of dichloromethane led to slightly lower yields (entries 2–3). While triaryloxopyrylium (**7**^+^) was found to display inferior catalytic efficiency, **2a** remained intact using transition-metal photocatalysts Ru(bpy)_3_^2+^ and Ru(bpz)_3_^2+^, which is consistent with the electrochemical data (entries 4–6).

**Table 1:** Evaluation of reaction parameters for transfer hydroalkylation.


Entry	Deviation from “standard conditions”	Yield of 4a (%)^a^
1	None	81
2	MeCN instead of DCM	66
3	DCE instead of DCM	73
4	**7**^+^BF_4_^−^ instead of **6**^+^ClO_4_^−^	39
5^b^	Ru(bpy)_3_^2+^ instead of **6**^+^ClO_4_^−^	0
6^b^	Ru(bpz)_3_^2+^ instead of **6**^+^ClO_4_^−^	0

Standard conditions: **2a** (0.10 mmol), **3a** (0.20 mmol), **6** (2.5 mol%), in 1.0 mL DCM at room temperature under blue light (450 nm) irradiation for 12 h.

^a^Determined by ^1^H NMR analysis of the crude reaction mixture.

^b^Carried out in MeCN.

### Substrate scope

**Fig. 3:**
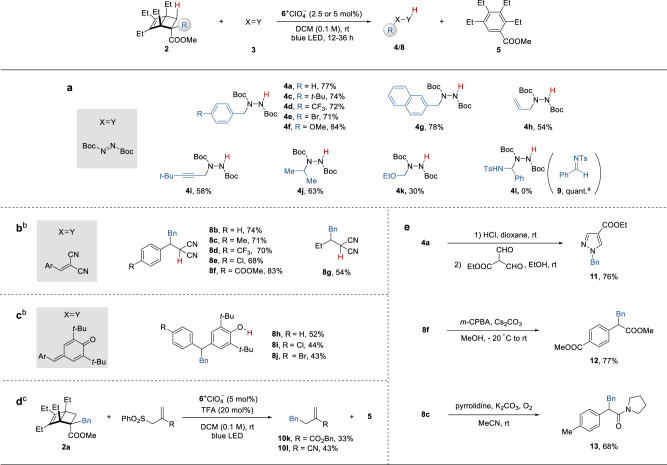
Evaluation of BCHs as alkyl radical donors and derivatization of alkylation products. **a** Scope of alkylated BCHs. **b**–**d** Scope of radical acceptors. **e** Derivatization of alkylation products. Standard conditions: **2** (0.20 mmol), **3** (0.40 mmol), **6** (2.5 or 5 mol%), in 2.0 mL DCM at room temperature under blue light irradiation (450 nm) for 12–36 h. Yields refer to isolated yields after chromatography. ^a^Determined by ^1^H NMR using an internal standard. ^b^In the presence of 20 mol% TFA. ^c^Carried out at a 0.10 mmol scale.

Next, we evaluated the performance of BCHs carrying different alkyl groups under the optimized conditions (Fig. 3a). A diverse range of benzyl groups bearing electron-donating and-withdrawing substituents and a 2-naphthylmethyl group were transferred to **3a** in good yields (**4c**-**4g**). Allyl-substituted and propargyl-substituted BCHs were also found to be viable donors, respectively (**4h**, **4i**). Notably, this constitutes a rare example wherein a noncyclic allyl radical is generated from photochemical oxidation, which might complement existing allylation methods^39^. This method proved amenable for secondary alkyl transfer (**4j**).

In terms of heteroatom-substitution, an ethoxymethyl group is transferrable, albeit in a lower yield due to a relatively high fragmentation barrier (**4k**). Interestingly, an α-amino benzyl substituted BCH gave no desired product under the standard conditions (**4l**). Instead, the corresponding sulfonimine **9** was detected in a quantitative yield. Related secondary α-amino and alkoxyl substituted BCHs were also found unsuitable (Fig. S3). This is presumably due to their propensity to release a stable alkyl cation rather than a radical upon oxidation. On the other hand, the transfer of heteroatom-centered such as Si-centered and N-centered radicals remains challenging using the current method (see SI-19 for discussions).

The scope of the reaction with respect to radical acceptors was demonstrated using **2a** as a benzyl donor (Fig. 3b, c). Electron-deficient alkenes such as malononitrile derivatives furnished the desired hydrobenzylation products in decent yields (**8b**–**8g**). *Para*-quinone methides produced the corresponding functionalized phenols in moderate yields (**8h**–**8j**). Alkyl transfer allylation via a radical addition-fragmentation pathway proved viable (**10k**–**l**, Fig. 3d). For less electron-deficient acceptors, the yields were significantly attenuated, which could be attributable to the slower reduction of the resulting radical adduct that limits the turnover (Fig. S3). Lastly, a few derivatization reactions were performed to demonstrate the synthetic utility of the alkylation products (Fig. 3e). *N*-Benzyl pyrazole **11** was synthesized from hydrazine derivative **4a** through a one-pot sequence in good yield. The dicyanomethyl group can be further transformed to an ester (**12**) or an amide (**13**) in good yields.

### Proposed mechanism and DFT calculations

**Fig. 4:**
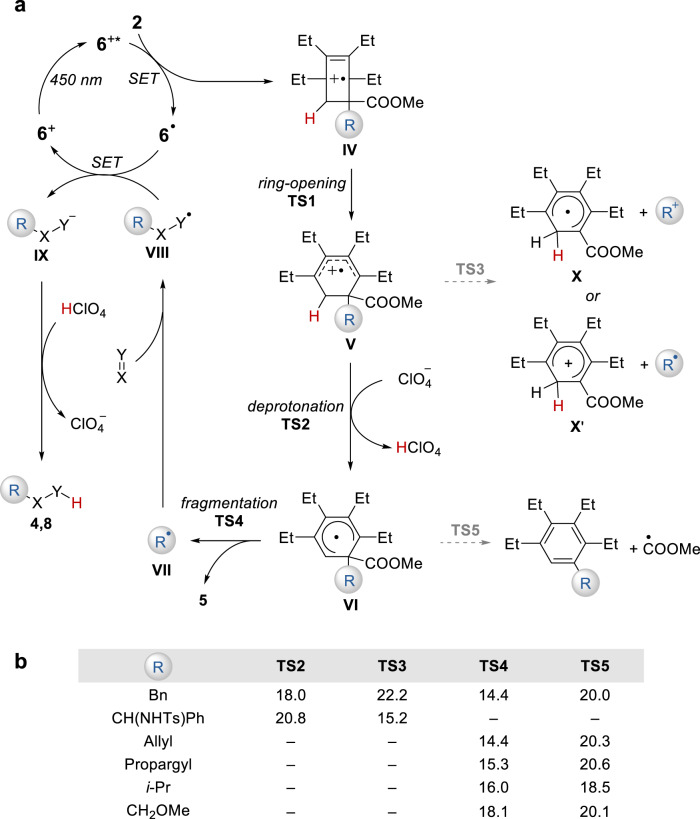
Mechanistic hypothesis and DFT study of the aromatization process. **a** Proposed mechanism of alkyl transfer reaction of alkylated BCHs. **b** Selected DFT calculations for competing pathways. Computed at the SMD(DCM)/UM06-2X/def2-TZVPP//SMD(DCM)/UB3LYP/6-31 + G(d,p) level for **TS2** and **TS3**. Computed at the SMD(DCM)/UM06-2X/def2-TZVPP//UB3LYP/6-31 + G(d,p) level for **TS4** and **TS5**. To simplify our calculations, the ethyl groups and tolylsulfonyl group are replaced by methyl groups and methylsulfonyl group, respectively. Energy in kcal/mol.

On the basis of the experimental results and DFT calculations, a plausible mechanism of the transfer alkylation reaction is depicted in Fig. 4a. Initially, the excited state of the acridinium photocatalyst oxidizes **2** into a strained π-radical cation, followed by barrierless rearrangement to afford an σ-radical cation **IV**, whose spin density mainly locates between the bridgehead carbons. Bridge C–C bond elongation leads to a diene radical cation species **V**. Calculations reveal that the ring-opening process (**IV** to **V**) is exothermic by ca. 40 kcal/mol with a barrier of less than 5 kcal/mol, and shows neglectable dependence on the initial stereochemistry in **2** (Fig. S12). Subsequently, deprotonation of **V** delivers a proaromatic radical intermediate **VI**, which readily fragments to form a nucleophilic alkyl radical **VII** driven by aromatization. **VII** is then intercepted by electron-deficient acceptors, followed by reduction and protonation that eventually produces the transfer hydroalkylation product. Alternatively, **VII** can be trapped by an allylsulfone via a radical addition-fragmentation pathway to deliver the allylation product and release a sulfonyl radical, which is further reduced to regenerate the photocatalyst (Fig. S17).

DFT calculations shed light on the selectivities in several key steps (Fig. 4b). A potential competing pathway from **V** involves the radical cation fragmentation at the quaternary carbon center (**TS3**). To this, our calculations suggest that the fate of **V** is substrate-dependent. For **2a** (R = Bn), deprotonation (**TS2**) is favored by 4.2 kcal/mol. However, in the case of **2l** (R = CH(NHTs)Ph), **TS3** is favored by 5.6 kcal/mol as a result of the cation-stabilizing α-amino group. Indeed, a spin population analysis of the fragmentation products of **2l** indicates the exclusive formation of a proaromatic radical **X** and an α-amino benzyl cation, which is consistent with the experimental observation of the formation of **9**. This implies that ionic group transfer reactions of BCH might be possible based on such a different fragmentation preference. Additionally, we evaluated the fragmentation barriers from **VI** carrying different alkyl groups. The result reveals that releasing a benzyl, an allyl, a propargyl, an isopropyl or a methoxymethyl radical (**TS4**) are all kinetically favored over the formation of a methoxycarbonyl radical (**TS5**), which is in line with the experimental results.

### Optical response of BCHs

Encouraged by the synthetic transformations using BCHs and an external photoredox catalyst, we sought to exploit **2** as a platform for functional materials via an intramolecular charge transfer (ICT) mechanism. The modular synthesis of BCHs allows for the facile incorporation of an electron–acceptor unit, which could bring light-triggered responses through ICT. Based on this hypothesis, a quinoline-derived BCH **2m** was synthesized.

**Fig. 5:**
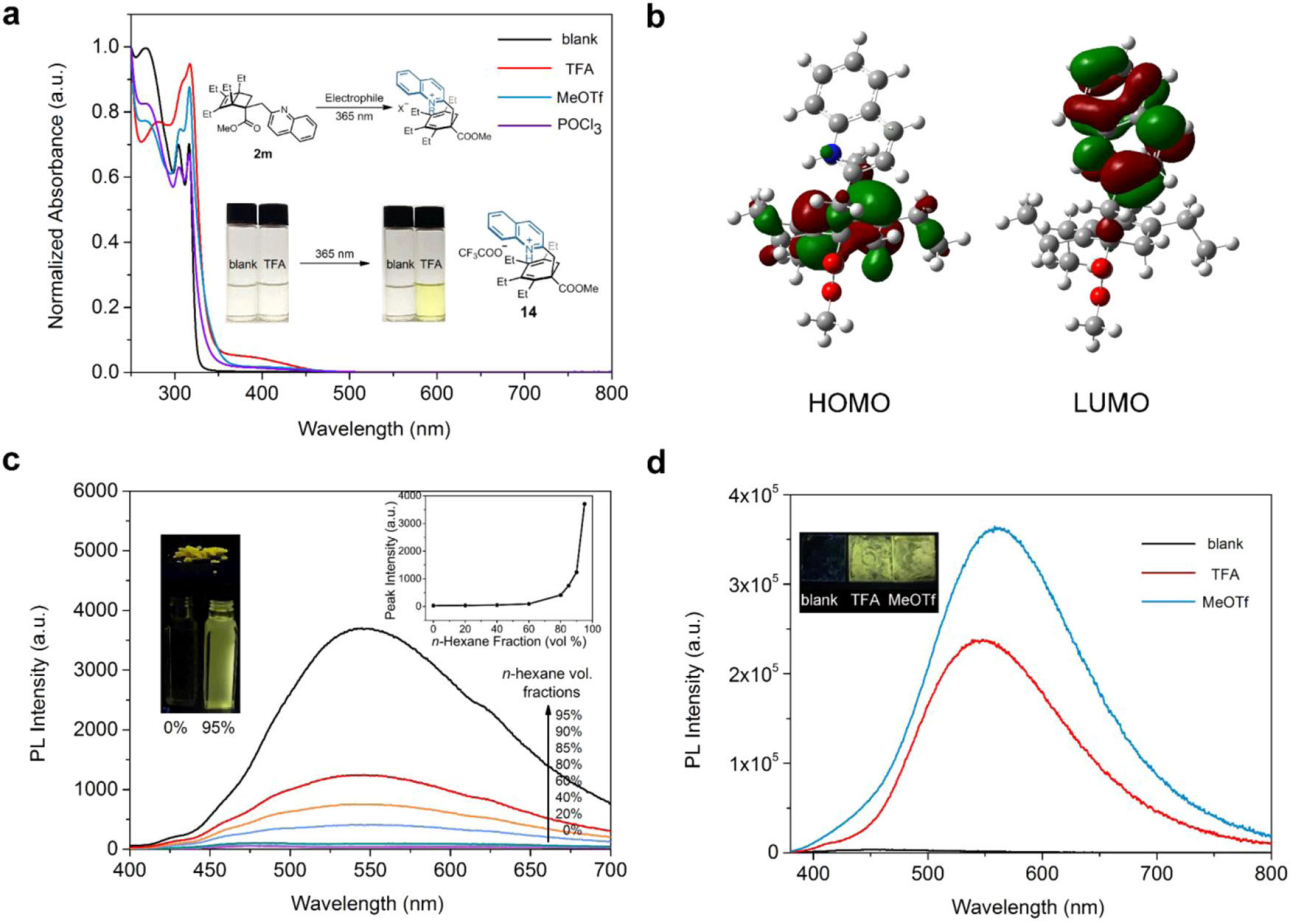
Optical response to electrophilic reagents. **a** UV–vis spectra of a 2.0 mM solution of **2m** in the presence of an electrophilic reagent in dichloromethane upon irradiation with 365 nm light. Inset: photos showing the colorimetric response. **b** Frontier molecular orbitals of cation of **14**. Computed at the SMD(DCM)/B3LYP/ 6-311G(d,p) level. **c** PL spectra of **14** (7.3 mM) in dichloromethane with varying volume fractions of *n*-hexane. Excitation wavelength: 365 nm. Inset 1 (left): fluorescence images of **14** in the solid state, dichloromethane solution, and dichloromethane/*n*-hexane (v/v = 5/95). Inset 2 (right): PL peak intensity as a function of volume fraction of *n*-hexane. **d** PL spectra of thin films of **2m** after exposure to electrophilic reagents. Inset: corresponding fluorescence images.

As shown in Fig. 5a, no obvious color change was observed when trifluoroacetic acid (TFA) was added to a dilute solution of **2m** in dichloromethane. However, upon irradiation at 365 nm for a few seconds, this solution turned yellow instantaneously, while a control sample without TFA remained colorless. In the absorption spectrum, a new broad band emerged around 400 nm corresponding to the formation of diene **14**, which was confirmed by independent synthesis. Similar responses were found with electrophilies such as methyl triflate and POCl_3_, which are often used as mimics for toxic alkylating reagents. Mechanistically, quaternizing the quinoline moiety would activate it as an electron–acceptor, and therefore turn on a photo-induced SET pathway. We hypothesized that such pathway would lead to **14** following a mechanism that is similar to that is depicted in Fig. 4a.

To interrogate the origin of the visible-light absorbance of **14**, preliminary computational studies were performed to elucidate its electronic structure and transitions. DFT calculation reveals that **14** displays well-separated HOMO and LUMO orbitals in the diene and quinolinium moieties, respectively (Fig. 5b). TD-DFT calculation at the SMD(DCM)/wB97XD/6-311G(d,p) level suggests that S_0_ to S_1_ vertical excitation of **14** is comprised of 96% HOMO-LUMO transition with a clear charge-transfer character, which occurs at 378 nm with an oscillator strength *f* of 0.12 and is in good agreement with the spectroscopic data (Fig. S7).

Interestingly, whereas **14** emits bright yellow light in the solid state, it is virtually non-emissive in dichloromethane solution. This phenomenon prompted us to explore the dependence of PL intensities on the solvent composition. As depicted in Fig. 5c, the fluorescence intensified dramatically when the volume fraction of an immiscible solvent, *n-*hexane, was greater than 80%. Such behavior is characteristic of an AIE luminogen, which might arise from the twisted structure of **14** and the restricted motions of the ethyl or quinolinium groups in the aggregated state^40–44^. As a control experiment, 2-methylquinolinium salt in the aggregated state displayed distinct blue emission, indicating that the broad PL emission of **14** is likely not a result of excimer formation (Fig. S10). Finally, this AIE phenomenon has enabled vapor sensing using a thin film of **2m**. To this, solid-state fluorescence response of **2m** towards several electrophilic reagents was explored. As demonstrated in Fig. 5d, whereas **2m** per se was only weakly blue fluorescent under 365 nm irradiation, bright yellow fluorescence was observed for the films exposed to either TFA or methyl triflate vapor.

## Discussion

In summary, we have developed a class of proaromatic structures, namely bicyclo[2.2.0]hexene (BCH) derivatives. They feature modular and easy synthesis as well as high thermal stability based on a valence isomerism strategy. Upon oxidation under mild conditions, BCHs are in situ unmasked through ring-opening and further aromatize to deliver various alkyl radicals. Thus, a new transfer hydroalkylation reaction using BCHs as the radical donor has been developed. The selectivities of several key steps in the aromatization process of BCHs were studied by DFT calculations. In addition, a quinoline-derived BCH was synthesized and demonstrated as a colorimetric and fluorescent sensor for electrophilic reagents. This response was ascribed to a 1,3-CHD structure that possesses an ICT excited state, which is visible-light absorbing, fluorescent, and AIE-active. This study demonstrates proaromatic BCHs’ synthetic utility in group transfer reactions, and their potential as a new platform for designing responsive materials, for example, water-soluble AIE luminogens with ICT characters. It is anticipated that these structurally unique molecules will find more applications in both synthetic chemistry and material sciences.

## Methods

### General procedure for the transfer alkylation reaction

An oven-dried 20 mL re-sealable screw-cap tube equipped with a magnetic stir bar was charged with acridinium photocatalyst **6** (2.1 mg, 0.005 mmol, 2.5 mol%) and di-*tert*-butyl azodicarboxylate (92.1 mg, 0.40 mmol, 2.0 equiv.). The tube was sealed. The tube was evacuated and backfilled with nitrogen. This sequence was repeated for a total of three times. To the tube were added an alkylated BCH **2** (0.20 mmol, 1.0 equiv.) and anhydrous dichloromethane (2.0 mL) via syringe. The reaction mixture was stirred at room temperature under blue light irradiation (450 nm) for 12 or 24 h. The solvent was removed in vacuo and the residue was analyzed by ^1^H NMR spectroscopy using dibromomethane as an internal standard. The solution in NMR tube was collected, concentrated in vacuo and purified by flash column chromatography to afford alkyl transfer product **4**.

An oven-dried 20 mL re-sealable screw-cap tube equipped with a magnetic stir bar was charged with acridinium photocatalyst **6** (4.1 mg, 0.01 mmol, 5 mol%) and radical acceptors **3** (0.40 mmol, 2.0 equiv.). The tube was sealed. The tube was evacuated and backfilled with nitrogen. This sequence was repeated for a total of three times. To the tube were sequentially added **2a** (68.2 mg, 0.20 mmol, 70 μL, 1.0 equiv.), anhydrous dichloromethane (2.0 mL) and trifluoroacetic acid (0.04 mmol, 3.0 μL, 20 mol%) via syringe. The reaction mixture was stirred at room temperature under blue light irradiation (450 nm) for 24 or 36 h. The solvent was removed in vacuo and the residue was analyzed by ^1^H NMR spectroscopy using dibromomethane as an internal standard. The solution in NMR tube was collected, concentrated in vacuo, and purified by flash column chromatography to afford alkyl transfer product **8b**–**g**.

## Supplementary information

Supplementary Information

## Data Availability

Additional data and computational study details supporting the findings described in this manuscript are available in the Supplementary Information. For full characterization data of new compounds and experimental details, see Supplementary Methods and Figures in Supplementary Information file. The X-ray crystallographic coordinates for structure **2b** reported in this study have been deposited at the Cambridge Crystallographic Data Centre (CCDC), under deposition number 2044047 [10.5517/ccdc.csd.cc26m00w]. These data can be obtained free of charge from The Cambridge Crystallographic Data Centre via http://www.ccdc.cam.ac.uk/data_request/cif. All other data are available from the authors upon reasonable request.
