# Supplementary material for: Bicyclo[2.2.0]hexene derivatives as a proaromatic platform for group transfer and chemical sensing
Source: Nat Commun. 2021 Jun 17;12:3680. doi: 10.1038/s41467-021-24054-3 (PMC8211693; doi:10.1038/s41467-021-24054-3)
Supplement: Supplementary file 1 — Supplementary Information [file 41467_2021_24054_MOESM1_ESM.pdf]

Supporting Information

Bicyclo[2.2.0]hexene Derivatives as a Proaromatic Platform for Group Transfer and Chemical Sensing

Bin Wu, Jianing Wang, Xingchen Liu and Rong Zhu*

Beijing National Laboratory for Molecular Sciences (BNLMS), Key Laboratory of Bioorganic Chemistry and Molecular Engineering of Ministry of Education, College of Chemistry and Molecular Engineering, Peking University, Beijing 100871, China

*Corresponding author: rongzhu@pku.edu.cn

Table of Contents

I. General Information **SI-2**

II. General Procedures and Characterizations **SI-3**

II-A. Synthesis and Characterizations of Non-Commercial Substrates SI-3

II-B. General Procedures for the Alkyl Transfer Reaction with **3a**, Characterizations of **4a-k** SI-12

II-C. Unsuccessful Examples SI-18

II-D. General Procedures for the Alkyl Transfer Reaction with **2a** and Characterizations of **8b-j, 10k, 1** SI-20

II-E. Procedures for Derivatization of Alkylation Products and Characterizations of **11-13** SI-26

III. Cyclic Voltammetry and Stern-Volmer Quenching Experiments **SI-28**

IV. Light-Triggered Responses of 2m **SI-31**

IV-A. Independent Synthesis of **14** and Neutral 1,3-CHD **S1** SI-31

IV-B. UV-Vis Absorption and Excitation Spectra of **14** SI-33

IV-C. Fluorescence Sensing for $POCl_3$ and Emission Spectrum of 2-Methyl Quinolinium SI-34

IV-D. Excitation Spectrum of the **2m** (Film) Exposed to TFA SI-35

V. DFT Studies **SI-36**

V-A. Computed Energies for the Stationary Points SI-36

V-B. Energy Levels, Excitation Energies and Oscillator Strengths for **14** SI-41

V-C. Cartesian Coordinates for the Stationary Points SI-42

VI. X-Ray Crystal Analysis **SI-58**

VII. Mechanism for Alkyl Transfer Allylation Reaction **SI-60**

VIII. SI References and Notes **SI-61**

IX. NMR Spectra **SI-63**

I. General Information

All reactions were carried out with dry solvents under anhydrous conditions, unless otherwise noted. Reagents and solvents were purchased from commercial suppliers and used without further purification. Yields refer to spectroscopically (^{1}H NMR) pure materials, unless otherwise noted. The TLC analysis was carried out on silica gel plates (HSGF254) supplied by Yantai Chemicals (China). Further visualization was achieved by irradiation with 254 nm light, staining with iodine or phosphomolybdic acid followed by heating. Flash silica gel chromatography was performed using silica gel (200-300 mesh) supplied by Tsingtao Haiyang. ^{1}H and ^{13}C NMR spectra were recorded on a Bruker 400 spectrometer and were calibrated using $CDCl_3$ as internal reference (7.26 ppm for ^{1}H NMR and 77.16 ppm for ^{13}C NMR). The following abbreviations were used to explain the multiplicities: s = singlet, d = doublet, t = triplet, dd = doublet of doublets, dt = doublet of triplets, q = quartet, m = multiplet, br = broad. IR spectra were recorded on a Thermo Scientific Nicolet iS5 FT-IR spectrometer (iD5 ATR). All high-resolution mass spectra were recorded on a Solarix XR Fourier Transform Ion Cyclotron Resonance Mass Spectrometer. Cyclic voltammetry experiments were performed on a CH Instruments 630E potentiostat. Excitation and emission spectrum were recorded on a Shimadzu RF-6000 Fluorescence Spectrometer. X-ray crystal diffraction data were collected on a XtaLAB PRO 007HF(Mo) single crystal X-ray diffractometer.

II. General Procedures and Characterizations

II-A. Synthesis and Characterizations of Non-Commercial Substrates

Synthesis of Radical Acceptors

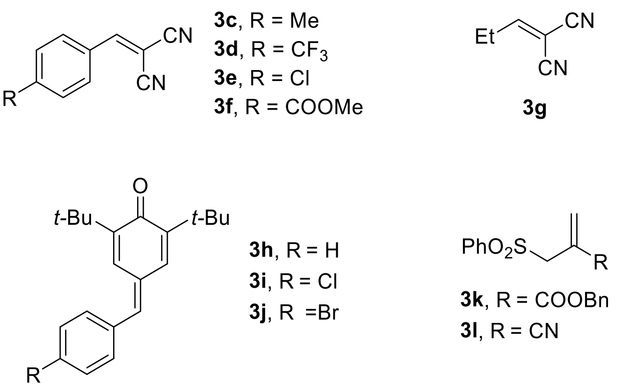

Fig. S1 Scope of radical acceptors

3c-g,[1] **3h-j**,[2] **3k-l**[3] were prepared according to literature procedures.

Methyl 1,4,5,6-tetraethylbicyclo[2.2.0]hex-5-ene-2-carboxylate (1)

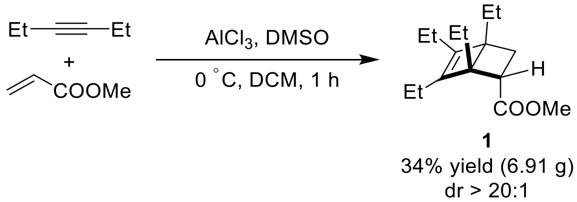

Adapted from a literature procedure,[4] a Schlenk flask equipped with a magnetic stir bar was charged with aluminum chloride (10.68 g, 80.0 mmol, 1.0 equiv.). The Schlenk flask was sealed, evacuated and backfilled with nitrogen. This sequence was repeated for a total of three times. Anhydrous DCM (80 mL) was added via syringe and the resulting slurry was cooled to -15°C. 3-Hexyne (13.14 g, 160.0 mmol, 20.0 mL, 2.0 equiv.) was added dropwise via syringe and the mixture was stirred for additional 15 min to prepare a solution of cyclobutadiene–aluminum chloride complex. Another Schlenk flask equipped with a magnetic stir bar was sealed, evacuated and backfilled with nitrogen. This sequence was repeated for a total of three times. To the flask was added anhydrous DCM (20 mL), methyl acrylate (6.89 g, 80.0 mmol, 7.2 mL, 1.0 equiv.) and DMSO (12.50 g, 160.0 mmol, 11.6 mL, 2.0 equiv.). The resulting solution was cooled to 0 °C before a solution of cyclobutadiene–aluminum chloride complex was added via syringe. Then the reaction mixture was stirred at 0 °C for 1 h before it was poured onto ice and diluted with ether. The aqueous phase was extracted with ether and the collected organic layers were washed with brine, dried

over anhydrous Na_2SO_4, filtered, concentrated *in vacuo*. The residue was purified by silica gel column chromatography (PE – PE:EtOAc = 400:1) to afford **1** (6.91 g, 34%, dr > 20:1) as a yellow oil.

^{1}H NMR (400 MHz, Chloroform-*d*) δ 3.59 (s, 3H), 2.90 (dd, *J* = 10.0, 6.1 Hz, 1H), 2.21 – 1.97 (m, 4H), 1.91 – 1.79 (m, 2H), 1.73 – 1.58 (m, 2H), 1.52 (q, *J* = 7.5 Hz, 2H), 1.05 (t, *J* = 7.6 Hz, 3H), 0.99 (t, *J* = 7.6 Hz, 3H), 0.95 (t, *J* = 7.5 Hz, 3H), 0.88 (t, *J* = 7.5 Hz, 3H).

^{13}C NMR (101 MHz, Chloroform-*d*) δ 175.1, 148.3, 143.1, 57.9, 51.7, 51.0, 40.5, 28.0, 25.54, 24.46, 20.6, 20.2, 13.2, 12.9, 10.1, 9.8.

FTIR (cm^{-1}): 2964, 2935, 2877, 2846, 1734, 1459, 1433, 1376, 1169, 1046, 909, 838, 732.

HRMS (ESI) Calcd. For ($[C_{16}H_{26}O_2]+H^+$): 251.2006; Found: 251.2001.

R_f (PE:EtOAc = 20:1) = 0.70.

General Procedure for the Synthesis of Alkylated BCHs 2a-2m

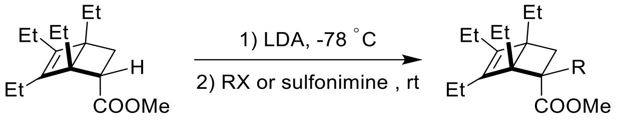

1 **2a-2m**

General Procedure A: A 50 mL Schlenk tube equipped with a magnetic stir bar was charged with methyl 1,4,5,6-tetraethylbicyclo[2.2.0]hex-5-ene-2-carboxylate **1** (1.0 equiv.). The Schlenk tube was sealed, cooled to -78 °C, evacuated and backfilled with nitrogen. This sequence was repeated for a total of three times. The tube was warmed to room temperature and anhydrous THF was added via syringe (concentration: 1.0 g/mL in THF). The solution was cooled to -78 °C and LDA solution (2.0 M, 1.5 equiv.) was added dropwise via syringe. The solution was stirred at -78 °C for 6 h before an alkyl halide or sulfonimine was added via syringe (a solution in anhydrous THF was added for solid alkylation reagents). The resulting solution was allowed to warm to room temperature and stirred at room temperature for additional 14 – 36 h. The reaction mixture was quenched with aqueous saturated NH_4Cl solution. The aqueous phase was extracted with ethyl acetate for 3 times and the collected organic layers were washed with 0.1 M aqueous HCl solution, brine, dried over anhydrous Na_2SO_4, filtered, concentrated *in vacuo*. The residue was purified by silica gel column chromatography.

Methyl 2-benzyl-1,4,5,6-tetraethylbicyclo[2.2.0]hex-5-ene-2-carboxylate (2a)

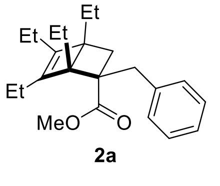

2a

Following General Procedure A, the title compound was synthesized from **1** (1.20 g, 4.8 mmol, 1.0 equiv.) and benzyl bromide (1.7 mL, 14.4 mmol, 3.0 equiv.). The reaction mixture

was stirred at room temperature for 19 h. The product was purified by silica gel flash column chromatography (PE – PE:EtOAc = 100:1) to afford **2a** (1.48 g, 91%, dr = 15:1) as a yellow oil.

^{1}H NMR (400 MHz, Chloroform-*d*) δ 7.25 – 7.07 (m, 5H), 3.57 (s, 3H), 3.36 (d, *J* = 13.4 Hz, 1H), 2.81 (d, *J* = 13.5 Hz, 1H), 2.28 (dd, *J* = 12.3, 1.3 Hz, 1H), 2.19 – 1.58 (m, 9H), 1.09 – 0.95 (m, 12H).

^{13}C NMR (101 MHz, Chloroform-*d*) δ 175.7, 150.3, 142.9, 138.8, 129.2, 128.3, 126.3, 60.4, 51.0, 50.7, 50.3, 40.9, 34.0, 24.8, 20.7, 20.1, 20.0, 13.2, 12.7, 10.7, 10.0.

FTIR (cm^{-1}): 2963, 2934, 2876, 1735, 1719, 1454, 1432, 1170, 1088, 1031, 741, 699.

HRMS (ESI) Calcd. For ([$C_{23}H_{32}O_2$]+H^+): 341.2475; Found: 341.2477.

R$_f$ (PE:EtOAc = 20:1) = 0.60.

Methyl 1,4,5,6-tetraethyl-2-(4-(methoxycarbonyl)benzyl)bicyclo[2.2.0]hex-5-ene-2-carboxylate (2b)

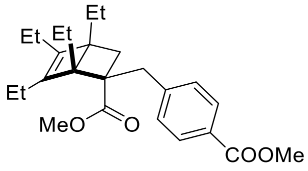

2b

Following General Procedure A, the title compound was synthesized from **1** (0.75 g, 3.0 mmol, 1.0 equiv.) and methyl 4-(bromomethyl)benzoate (1.03 g, 4.5 mmol, 1.5 equiv.). The reaction mixture was stirred at room temperature for 12 h. The product was purified by silica gel flash column chromatography (PE:EtOAc = 60:1 – 30:1) followed by recrystallization from *n*-hexane to afford **2b** (0.28 g, 23%, single diastereomer) as a white solid.

^{1}H NMR (400 MHz, Chloroform-*d*) δ 7.90 (d, *J* = 8.3 Hz, 2H), 7.17 (d, *J* = 8.3 Hz, 2H), 3.88 (s, 3H), 3.56 (s, 3H), 3.40 (d, *J* = 13.4 Hz, 1H), 2.86 (d, *J* = 13.4 Hz, 1H), 2.30 (dd, *J* = 12.3, 1.2 Hz, 1H), 2.20 – 1.51 (m, 9H), 1.10 – 0.95 (m, 12H).

^{13}C NMR (101 MHz, Chloroform-*d*) δ 175.4, 167.2, 150.5, 144.5, 142.9, 129.7, 129.2, 128.4, 60.5, 52.1, 51.1, 50.6, 50.4, 41.0, 34.1, 24.8, 20.8, 20.2, 20.0, 13.2, 12.7, 10.7, 10.1.

FTIR (cm^{-1}): 2964, 2846, 1720, 1611, 1459, 1434, 1276, 1180, 1106, 1020, 758, 732, 705.

HRMS (ESI) Calcd. For ([$C_{25}H_{34}O_4$]+H^+): 399.2530; Found: 399.2523.

R$_f$ (PE:EtOAc = 9:1) = 0.40.

Methyl 2-(4-(*tert*-butyl)benzyl)-1,4,5,6-tetraethylbicyclo[2.2.0]hex-5-ene-2-carboxylate (2c)

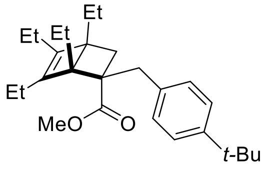

2c

Following General Procedure A, the title compound was synthesized from **1** (1.00 g, 4.0 mmol, 1.0 equiv.) and 1-(bromomethyl)-4-(tert-butyl)benzene (2.2 mL, 12.0 mmol, 3.0 equiv.). The reaction mixture was stirred at room temperature for 23 h. The product was purified by silica gel flash column chromatography (PE – PE:EtOAc = 200:1) to afford **2c** (1.37 g, 86%, dr = 17:1) as a yellow oil.

^{1}H NMR (400 MHz, Chloroform-*d*) δ 7.26 – 7.20 (m, 2H), 7.04 – 6.98 (m, 2H), 3.59 (s, 3H), 3.33 (d, *J* = 13.5 Hz, 1H), 2.77 (d, *J* = 13.5 Hz, 1H), 2.29 (dd, *J* = 12.3, 1.3 Hz, 1H), 2.20 – 1.51 (m, 9H), 1.28 (s, 9H), 1.09 – 0.94 (m, 12H).

^{13}C NMR (101 MHz, Chloroform-*d*) δ 175.9, 150.3, 149.0, 142.9, 135.7, 128.8, 125.2, 60.4, 51.0, 50.5, 50.3, 40.3, 34.4, 34.0, 31.5, 24.8, 20.8, 20.1, 20.0, 13.2, 12.7, 10.8, 10.1.

FTIR (cm^{-1}): 2963, 2935, 2875, 1720, 1512, 1460, 1432, 1193, 1169, 1121, 908, 836, 732.

HRMS (ESI) Calcd. For ([$C_{27}H_{40}O_2$]+H^+): 397.3101; Found: 397.3087.

R$_f$ (PE:EtOAc = 20:1) = 0.75.

Methyl 1,4,5,6-tetraethyl-2-(4-(trifluoromethyl)benzyl)bicyclo[2.2.0]hex-5-ene-2-carboxylate (2d)

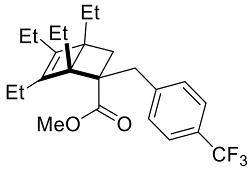

2d

Following General Procedure A, the title compound was synthesized from **1** (1.00 g, 4.0 mmol, 1.0 equiv.) and 1-(bromomethyl)-4-(trifluoromethyl)benzene (1.9 mL, 12.0 mmol, 3.0 equiv.). The reaction mixture was stirred at room temperature for 36 h. The product was purified by silica gel flash column chromatography (PE – PE:EtOAc = 200:1) to afford **2d** (0.61 g, 37%, dr = 5.5:1) as a yellow solid.

^{1}H NMR (400 MHz, Chloroform-*d*) δ 7.48 (d, *J* = 8.1 Hz, 2H), 7.21 (d, *J* = 8.2 Hz, 2H), 3.57 (s, 3H), 3.41 (d, *J* = 13.5 Hz, 1H), 2.86 (d, *J* = 13.7, 1H), 2.31 (dd, *J* = 12.3, 1.2 Hz, 1H), 2.19 – 1.59 (m, 9H), 1.21 – 0.86 (m, 12H).

^{13}C NMR (101 MHz, Chloroform-d) for the major isomer δ 175.4, 150.5, 143.1, 142.9, 129.5, 128.7 (q, *J* = 32.3 Hz), 125.3 (q, *J* = 3.8 Hz), 124.4 (q, *J* = 271.8 Hz), 60.5, 51.2, 50.6, 50.4, 40.7, 34.1, 24.8, 20.8, 20.2, 20.0, 13.2, 12.7, 10.7, 10.0.

^{13}C NMR (101 MHz, Chloroform-d) for the minor isomer δ 175.8, 151.6, 129.4, 60.1, 53.8,

51.4, 49.5, 41.4, 32.2, 23.8, 22.1, 21.8, 21.1, 13.1, 10.6, 9.7.
FTIR (cm^{-1}): 2965, 2936, 1732, 1618, 1460, 1322, 1162, 1112, 1067, 1019, 843, 733.
HRMS (ESI) Calcd. For ($[C_{24}H_{31}F_3O_2]+H^+$): 409.2349; Found: 409.2352.
$\mathbf{R_f}$ (PE:EtOAc = 20:1) = 0.80.

Methyl 2-(4-bromobenzyl)-1,4,5,6-tetraethylbicyclo[2.2.0]hex-5-ene-2-carboxylate (2e)

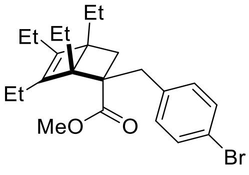

2e

Following General Procedure A, the title compound was synthesized from **1** (0.75 g, 3.0 mmol, 1.0 equiv.) and 1-bromo-4-(bromomethyl)benzene (2.25 g, 9.0 mmol, 3.0 equiv.). The reaction mixture was stirred at room temperature for 14 h. The product was purified by silica gel flash column chromatography (PE – PE:EtOAc = 400:1) to afford **2e** (0.80 g, 64%, dr = 9:1) as a pale yellow oil.
^{1}H NMR (400 MHz, Chloroform-*d*) δ 7.36 – 7.31 (m, 2H), 6.99 – 6.95 (m, 2H), 3.56 (s, 3H), 3.30 (d, *J* = 13.5 Hz, 1H), 2.76 (d, *J* = 13.5 Hz, 1H), 2.28 (dd, *J* = 12.3, 1.2 Hz, 1H), 2.20 – 1.59 (m, 9H), 1.11 – 0.93 (m, 12H).
^{13}C NMR (101 MHz, Chloroform-*d*) δ 175.5, 150.4, 142.9, 137.8, 131.4, 130.9, 120.4, 60.4, 51.1, 50.6, 50.3, 40.3, 34.0, 24.8, 20.8, 20.1, 20.0, 13.2, 12.7, 10.7, 10.1.
FTIR (cm^{-1}): 2964, 2875, 1732, 1719, 1487, 1459, 1433, 1308, 1168, 1072, 1033, 834, 731.
HRMS (ESI) Calcd. For ($[C_{23}H_{31}BrO_2]+H^+$): 419.1586; Found: 419.1608.
$\mathbf{R_f}$ (PE:EtOAc = 20:1) = 0.80.

Methyl 1,4,5,6-tetraethyl-2-(4-methoxybenzyl)bicyclo[2.2.0]hex-5-ene-2-carboxylate (2f)

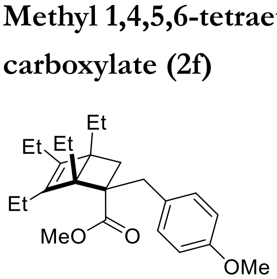

2f

Following General Procedure A, the title compound was synthesized from **1** (0.75 g, 3.0 mmol, 1.0 equiv.) and 1-(bromomethyl)-4-methoxybenzene (0.66 mL, 4.5 mmol, 1.5 equiv.). The reaction mixture was stirred at room temperature for 14 h. The product was purified by silica gel flash column chromatography (PE – PE:EtOAc = 100:1) to afford **2f** (0.69 g, 62%, dr = 17:1) as a yellow oil.

^{1}H NMR (400 MHz, Chloroform-*d*) δ 7.04 – 6.97 (m, 2H), 6.80 – 6.72 (m, 2H), 3.76 (s, 3H), 3.57 (s, 3H), 3.29 (d, *J* = 13.5 Hz, 1H), 2.74 (d, *J* = 13.5 Hz, 1H), 2.26 (dd, *J* = 12.4, 1.2 Hz, 1H), 2.19 – 1.57 (m, 9H), 1.15 – 0.85 (m, 12H).
^{13}C NMR (101 MHz, Chloroform-*d*) δ 175.8, 158.2, 150.3, 143.0, 130.8, 130.1, 113.8, 60.3, 55.2, 51.0, 50.9, 50.2, 40.0, 34.0, 24.8, 20.7, 20.1, 20.0, 13.2, 12.7, 10.8, 10.0.
FTIR (cm^{-1}): 2963, 2934, 2876, 1733, 1611, 1512, 1460, 1247, 1178, 1113, 1037, 733.
HRMS (ESI) Calcd. For ([$C_{24}H_{34}O_3$]+H^+): 371.2581; Found: 371.2582.
R$_f$ (PE:EtOAc = 9:1) = 0.50.

Methyl 1,4,5,6-tetraethyl-2-(naphthalen-2-ylmethyl)bicyclo[2.2.0]hex-5-ene-2-carboxylate (2g)

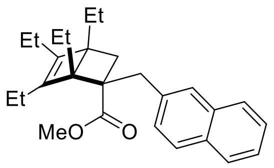

2g

Following General Procedure A, the title compound was synthesized from **1** (0.75 g, 3.0 mmol, 1.0 equiv.) and 2-(bromomethyl)naphthalene (1.99 g, 9.0 mmol, 3.0 equiv.). The reaction mixture was stirred at room temperature for 30 h. The product was purified by silica gel flash column chromatography (PE – PE:EtOAc = 100:1) to afford **2g** (0.75 g, 64%, dr > 20:1) as a yellow oil.
^{1}H NMR (400 MHz, Chloroform-*d*) δ 7.79 – 7.69 (m, 3H), 7.55 (d, *J* = 1.6 Hz, 1H), 7.45 – 7.37 (m, 2H), 7.23 (dd, *J* = 8.4, 1.8 Hz, 1H), 3.57 (s, 3H), 3.52 (d, *J* = 13.5 Hz, 1H), 2.98 (d, *J* = 13.5 Hz, 1H), 2.32 (dd, *J* = 12.4, 1.3 Hz, 1H), 2.22 – 1.59 (m, 9H), 1.10 – 0.98 (m, 12H).
^{13}C NMR (101 MHz, Chloroform-*d*) δ 175.8, 150.4, 143.0, 136.4, 133.6, 132.3, 127.83, 127.83, 127.71, 127.65, 127.6, 125.9, 125.4, 60.5, 51.1, 50.8, 50.4, 41.1, 34.2, 24.8, 20.8, 20.2, 20.1, 13.3, 12.7, 10.8, 10.1.
FTIR (cm^{-1}): 3055, 2963, 2933, 2875, 1732, 1718, 1459, 1433, 1192, 1152, 906, 818, 742.
HRMS (ESI) Calcd. For ([$C_{27}H_{34}O_2$]+H^+): 391.2632; Found: 391.2636.
R$_f$ (PE:EtOAc = 20:1) = 0.65.

Methyl 2-allyl-1,4,5,6-tetraethylbicyclo[2.2.0]hex-5-ene-2-carboxylate (2h)

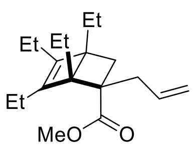

2h

Following General Procedure A, the title compound was synthesized from **1** (0.75 g, 3.0

mmol, 1.0 equiv.) and allyl bromide (0.8 mL, 9.0 mmol, 3.0 equiv.). The reaction mixture was stirred at room temperature for 20 h The product was purified by silica gel flash column chromatography (PE – PE:EtOAc = 100:1) to afford **2h** (0.73 g, 84%, dr = 10:1) as a yellow oil.

^{1}H NMR (400 MHz, Chloroform-*d*) δ 5.63 (ddt, *J* = 17.0, 10.1, 7.0 Hz, 1H), 5.08 – 4.92 (m, 2H), 3.60 (s, 3H), 2.70 (ddd, *J* = 13.3, 7.0, 1.3 Hz, 1H), 2.33 (dd, *J* = 12.2, 1.3 Hz, 1H), 2.28 – 1.74 (m, 6H), 1.62 – 1.47 (m, 4H), 1.05 (t, *J* = 7.6 Hz, 3H), 1.04 (t, *J* = 7.6 Hz, 3H), 0.93 (t, *J* = 7.5 Hz, 3H), 0.92 (t, *J* = 7.5 Hz, 3H).

^{13}C NMR (101 MHz, Chloroform-*d*) for the major isomer δ 175.9, 150.3, 143.0, 134.5, 117.1, 59.7, 51.1, 50.3, 49.5, 40.2, 34.4, 24.7, 20.7, 20.10, 20.05, 13.2, 12.8, 10.7, 9.9.

^{13}C NMR (101 MHz, Chloroform-*d*) for the minor isomer δ 176.3, 150.7, 143.5, 134.9, 116.6, 59.3, 52.5, 51.3, 49.4, 40.7, 31.8, 24.0, 22.0, 21.7, 21.0, 13.1, 13.0, 10.5, 9.7.

FTIR (cm^{-1}): 2965, 2935, 2877, 1735, 1460, 1433, 1310, 1210, 1153, 1136, 914, 734.

HRMS (ESI) Calcd. For ([$C_{19}H_{30}O_2$]+H^+): 291.2319; Found: 291.2328.

R$_f$ (PE:EtOAc = 20:1) = 0.65.

Methyl 2-(4,4-dimethylpent-2-yn-1-yl)-1,4,5,6-tetraethylbicyclo[2.2.0]hex-5-ene-2-carboxylate (2i)

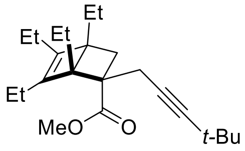

2i

Following General Procedure A, the title compound was synthesized from **1** (0.71 g, 2.8 mmol, 1.0 equiv.) and 1-iodo-4,4-dimethylpent-2-yne (2.95 g, 72 wt% solution in *n*-hexane, 10.6 mmol, 3.7 equiv.). The reaction mixture was stirred at room temperature for 12 h The product was purified by silica gel flash column chromatography (PE – PE:EtOAc = 200:1) to afford **2i** (0.91 g, 93%, dr = 5:1) as a yellow oil.

^{1}H NMR (400 MHz, Chloroform-*d*) δ 3.68 & 3.60 (s, 3H), 2.74 – 2.65 (m, 1H), 2.42 – 2.22 (m, 2H), 2.19 – 1.66 (m, 6H), 1.59 – 1.46 (m, 3H), 1.13 & 1.11 (s, 9H), 1.08 – 0.83 (m, 12H).

^{13}C NMR (101 MHz, Chloroform-*d*) for the major isomer δ 175.2, 150.3, 142.8, 91.0, 75.3, 59.0, 51.3, 50.2, 49.6, 34.7, 31.3, 27.4, 25.8, 24.5, 20.8, 20.1, 19.9, 13.2, 12.8, 10.6, 9.8.

^{13}C NMR (101 MHz, Chloroform-*d*) for the minor isomer δ 175.7, 151.3, 143.1, 89.8, 75.9, 58.5, 52.9, 51.4, 49.0, 32.8, 31.4, 27.4, 26.4, 23.9, 21.8, 21.7, 20.9, 12.9, 10.4, 9.6.

FTIR (cm^{-1}): 2965, 2934, 2876, 1738, 1459, 1433, 1311, 1265, 1206, 1193, 1151, 734.

HRMS (ESI) Calcd. For ([$C_{23}H_{36}O_2$]+H^+): 345.2788; Found: 345.2780.

R$_f$ (PE:EtOAc = 20:1) = 0.65.

Methyl 1,4,5,6-tetraethyl-2-isopropylbicyclo[2.2.0]hex-5-ene-2-carboxylate (2j)

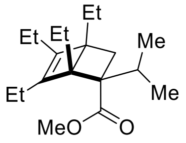

2j

Following General Procedure A, the title compound was synthesized from **1** (1.00 g, 4.0 mmol, 1.0 equiv.) and isopropyl iodide (2.0 mL, 20.0 mmol, 5.0 equiv.). The reaction mixture was stirred at -78 °C for 4 h, -40 °C for 5.5 h and room temperature for 5 h The product was purified by silica gel flash column chromatography (PE:EtOAc = 200:1 – 50:1) to afford an inseparable mixture of **1** and **2j**. Then the mixture was treated with LDA (3.0 mL, 1.5 equiv.) and 1-(bromomethyl)-3,5-dimethoxybenzene (1.85 g, 8.0 mmol, 2.0 equiv.) to remove **1**. The reaction mixture was quenched with aqueous NH_4Cl solurion, extracted with EA, dried over Na_2SO_4, filtered and concentrated *in vacuo*. The residue was purified by silica gel column chromatography (PE:EtOAc = 400:1) followed by preparative TLC (SiO_2, PE:EtOAc = 20:1)to afford **2j** (0.25 g, 22%, dr = 4:1) as a colorless oil.

^{1}H NMR (400 MHz, Chloroform-*d*) δ 3.66 & 3.60 (s, 3H), 2.62 & 2.26 (d, *J* = 12.1 Hz & 12.3 Hz, 1H), 2.18 – 1.65 (m, 8H), 1.55 – 1.49 (m, 1H), 1.16 – 0.98 (m, 7H), 0.97 – 0.72 (m, 12H).

^{13}C NMR (101 MHz, Chloroform-*d*) for two isomers δ 176.2, 175.9, 151.7, 150.5, 143.5, 142.6, 60.5, 59.1, 56.9, 54.9, 50.8, 50.6, 50.2, 48.6, 35.6, 33.3, 32.1, 24.5, 23.4, 23.2, 21.4, 20.9, 20.8, 20.5, 20.4, 20.2, 19.6, 17.5, 13.3, 13.2, 13.1, 12.9, 10.9, 10.7, 9.70, 9.65.

FTIR (cm^{-1}): 2965, 2935, 2877, 1724, 1461, 1376, 1256, 1194, 1152, 1127, 1055, 908, 734.

HRMS (ESI) Calcd. For ($[C_{19}H_{32}O_2]+H^+$): 293.2475; Found: 293.2472.

R_f (PE:EtOAc = 20:1) = 0.65.

Methyl 2-(ethoxymethyl)-1,4,5,6-tetraethylbicyclo[2.2.0]hex-5-ene-2-carboxylate (2k)

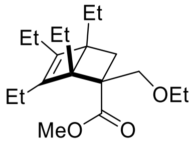

2k

Following General Procedure A, the title compound was synthesized from **1** (1.25 g, 5.0 mmol, 1.0 equiv.) and (chloromethoxy)ethane (80% purity, 3.8 mL, 2.4 equiv.). The reaction mixture was stirred at room temperature for 14 h The product was purified by silica gel flash column chromatography (PE – PE:EtOAc = 100:1) to afford **2k** (0.93 g, 60%, dr = 1.5:1) as a yellow oil.

^{1}H NMR (400 MHz, Chloroform-*d*) δ 3.95&3.85 (dd, *J* = 8.5, 1.0 Hz & *J* = 8.5, 1.4 Hz, 1H), 3.69&3.62 (s, 3H), 3.55 – 3.33 (m, 3H), 2.61&2.38 (d&dd, *J* = 12.4 Hz & *J* = 12.2, 1.5

Hz, 1H), 2.21 – 1.27 (m, 9H), 1.16 – 0.99 (m, 9H), 0.90 (dt, *J* = 12.6, 7.5 Hz, 5H), 0.80 (t, *J* = 7.4 Hz, 1H).

^{13}C NMR (101 MHz, Chloroform-*d*) for two isomers δ 175.4, 175.3, 150.7, 150.3, 143.1, 142.6, 75.2, 74.5, 66.9, 66.7, 59.0, 58.3, 53.4, 51.5, 51.3, 50.8, 50.1, 50.0, 33.8, 31.4, 24.5, 24.0, 22.0, 21.7, 20.9, 20.7, 20.1, 20.0, 15.13, 15.07, 13.2, 13.0, 12.9, 12.8, 10.8, 10.4, 9.8, 9.6.

FTIR (cm^{-1}): 2965, 2935, 2875, 1727, 1459, 1433, 1376, 1311, 1220, 1194, 1159, 1106, 908.

HRMS (ESI) Calcd. For ([$C_{19}H_{32}O_3$]+Na^+): 331.2244; Found: 331.2235.

R$_f$ (PE:EtOAc = 20:1) = 0.50.

Methyl 1,4,5,6-tetraethyl-2-(((4-methylphenyl)sulfonamido)(phenyl)methyl) bicyclo[2.2.0]hex-5-ene-2-carboxylate (2l)

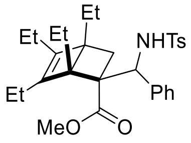

2l

Following General Procedure A, the title compound was synthesized from **1** (0.15 g, 0.60 mmol, 1.0 equiv.) and *N*-benzylidene-4-methylbenzenesulfonamide (0.31 g, 1.20 mmol, 2.0 equiv.). The reaction mixture was stirred at room temperature for 17 h The product was purified by silica gel flash column chromatography (PE:EtOAc = 20:1) to afford **2l** (0.23 g, 75%, single diastereomer) as a white solid.

^{1}H NMR (400 MHz, Chloroform-*d*) δ 7.18 (d, *J* = 7.9 Hz, 2H), 7.02 (t, *J* = 7.3 Hz, 1H), 6.94 (t, *J* = 7.5 Hz, 2H), 6.86 (t, *J* = 9.2 Hz, 3H), 6.78 (d, *J* = 7.5 Hz, 2H), 4.68 (d, *J* = 7.9 Hz, 1H), 3.59 (s, 3H), 2.33 – 2.25 (m, 1H), 2.24 (s, 3H), 2.18 – 1.84 (m, 6H), 1.69 – 1.59 (m, 3H), 1.07 (t, *J* = 7.6 Hz, 3H), 1.01 (t, *J* = 7.5 Hz, 3H), 0.95 (t, *J* = 7.6 Hz, 3H), 0.93 (t, *J* = 7.2 Hz, 3H).

^{13}C NMR (101 MHz, Chloroform-*d*) δ 175.5, 151.1, 142.2, 141.9, 138.8, 137.5, 128.8, 128.1, 127.9, 127.3, 126.5, 62.2, 61.7, 53.5, 52.0, 50.2, 33.6, 24.7, 21.4, 20.8, 20.7, 19.7, 12.8, 12.5, 10.7, 9.9.

FTIR (cm^{-1}): 3325, 2966, 2253, 1735, 1694, 1456, 1312, 1158, 906, 730, 702, 669, 560.

HRMS (ESI) Calcd. For ([$C_{30}H_{39}NO_4S$]+H^+): 510.2673; Found: 510.2675.

R$_f$ (PE:EtOAc = 5:1) = 0.50.

Methyl 1,4,5,6-tetraethyl-2-(quinolin-2-ylmethyl)bicyclo[2.2.0]hex-5-ene-2-carboxylate (2m)

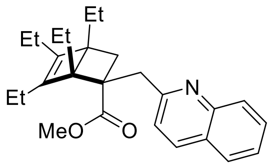

2m

Modified from General Procedure A, washing the extracts with 0.1 M HCl solution was omitted. The title compound was synthesized from **1** (0.79 g, 3.2 mmol, 1.0 equiv.) and 2-(bromomethyl)quinoline (0.84 g, 3.8 mmol, 1.2 equiv.). The reaction mixture was stirred at room temperature for 13 h. The product was purified by silica gel flash column chromatography (PE:EtOAc = 40:1 – 30:1) to afford **2m** (0.59 g, 48%, dr = 9:1) as a yellow oil.

^{1}H NMR (400 MHz, Chloroform-*d*) δ 8.03 – 7.94 (m, 2H), 7.73 (dd, *J* = 8.1, 1.4 Hz, 1H), 7.64 (ddd, *J* = 8.4, 6.9, 1.5 Hz, 1H), 7.45 (ddd, *J* = 8.1, 6.9, 1.2 Hz, 1H), 7.17 (d, *J* = 8.4 Hz, 1H), 3.75 (dd, *J* = 14.0, 1.3 Hz, 1H), 3.64 (s, 3H), 3.22 (d, *J* = 14.0 Hz, 1H), 2.35 (dd, *J* = 12.3, 1.3 Hz, 1H), 2.25 – 1.70 (m, 7H), 1.62 (q, *J* = 7.5 Hz, 2H), 1.12 – 0.93 (m, 12H).

^{13}C NMR (101 MHz, Chloroform-*d*) δ 176.0, 159.7, 150.3, 148.0, 142.9, 136.1, 129.30, 129.26, 127.5, 126.9, 125.9, 121.6, 60.3, 51.2, 50.4, 49.6, 44.0, 34.3, 24.8, 20.8, 20.4, 20.2, 13.3, 12.7, 10.8, 10.0.

FTIR (cm^{-1}): 2964, 2934, 2876, 1731, 1599, 1504, 1459, 1428, 1308, 1192, 1168, 908, 731.

HRMS (ESI) Calcd. For ([$C_{26}H_{33}NO_2$]+H^+): 392.2584; Found: 392.2568.

R_f (PE:EtOAc = 9:1) = 0.35.

II-B. General Procedures for the Alkyl Transfer Reaction with 3a and Characterizations of 4a-k

Evaluation of reaction conditions (Table 1, "Standard Conditions"): An oven-dried 20 mL re-sealable screw-cap tube equipped with a magnetic stir bar was charged with photocatalyst (0.0025 mmol, 2.5 mol%) and di-*tert*-butyl azodicarboxylate (46.1 mg, 0.20 mmol, 2.0 equiv.). The tube was sealed. The tube was evacuated and backfilled with nitrogen. This sequence was repeated for a total of three times. To the tube were added **2a** (34.1 mg, 35 μL, 0.10 mmol, 1.0 equiv.) and anhydrous solvent (1.0 mL) via syringe. The reaction mixture was stirred at room temperature under blue light irradiation (450 nm) for 12 h. The solvent was removed *in vacuo* and the residue was analyzed by ^{1}H NMR spectroscopy using dibromomethane as an internal standard.

General Procedure B (Fig. 3a): An oven-dried 20 mL re-sealable screw-cap tube equipped with a magnetic stir bar was charged with acridinium photocatalyst **6** (2.1 mg, 0.005 mmol, 2.5 mol%) and di-*tert*-butyl azodicarboxylate (92.1 mg, 0.40 mmol, 2.0 equiv.). The tube

was sealed. The tube was evacuated and backfilled with nitrogen. This sequence was repeated for a total of three times. To the tube were added an alkylated BCH **2** (0.20 mmol, 1.0 equiv.) and anhydrous dichloromethane (2.0 mL) via syringe. The reaction mixture was stirred at room temperature under blue light irradiation (450 nm) for 12 or 24 h. The solvent was removed *in vacuo* and the residue was analyzed by ^{1}H NMR spectroscopy using dibromomethane as an internal standard. The solution in NMR tube was collected, concentrated *in vacuo* and purified by flash column chromatography to afford alkyl transfer product **4**.

Methyl 2,3,4,5-tetraethylbenzoate (5)

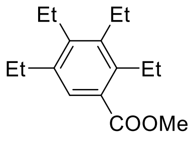

5

^{1}H NMR (400 MHz, Chloroform-*d*) δ 7.47 (s, 1H), 3.88 (s, 3H), 2.92 (q, *J* = 7.4 Hz, 2H), 2.69 (ddt, *J* = 18.9, 15.1, 7.6 Hz, 6H), 1.28 – 1.13 (m, 12H).

^{13}C NMR (101 MHz, Chloroform-*d*) δ 169.5, 144.3, 141.1, 140.7, 139.6, 128.7, 128.1, 51.9, 25.7, 23.0, 22.3, 21.8, 16.2, 15.9, 15.6, 15.4.

The spectroscopic data obtained is consistent with data reported in literature.[5]

Di-*tert*-butyl 1-benzylhydrazine-1,2-dicarboxylate (4a)

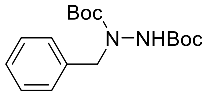

4a

Following General Procedure B, the title compound was synthesized from methyl 2-benzyl-1,4,5,6-tetraethylbicyclo[2.2.0]hex-5-ene-2-carboxylate (**2a**) (68.1 mg, 70 μL, 0.20 mmol) (reaction time = 12 h). The product was purified by silica gel column chromatography (PE:EtOAc = 40:1 – 20:1) to afford **4a** (49.5 mg, 77%) as a white solid.

^{1}H NMR (400 MHz, Chloroform-*d*) δ 7.36 – 7.22 (m, 5H), 6.29&6.06 (br, s, 1H), 4.64 (br, s, 2H), 1.48 (s, 9H), 1.44 (s, 9H).

^{13}C NMR (126 MHz, Chloroform-*d*) δ 155.2, 137.3, 128.6, 127.6, 81.3, 54.4, 53.0, 28.32, 28.25.

The spectroscopic data obtained is consistent with data reported in literature.[6]

Di-*tert*-butyl 1-(4-(*tert*-butyl)benzyl)hydrazine-1,2-dicarboxylate (4c)

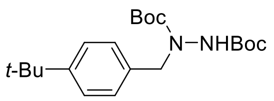

4c

Following General Procedure B, the title compound was synthesized from methyl 2-(4-(*tert*-butyl)benzyl)-1,4,5,6-tetraethylbicyclo[2.2.0]hex-5-ene-2-carboxylate (**2c**) (79.3 mg, 84 μL, 0.20 mmol) (reaction time = 12 h). The product was purified by silica gel column chromatography (PE:EtOAc = 40:1 – 20:1) to afford **4c** (56.3 mg, 74%) as a yellow oil.
^{1}H NMR (400 MHz, Chloroform-*d*) δ 7.35 (d, *J* = 8.3 Hz, 2H), 7.22 (d, *J* = 7.9 Hz, 2H), 6.27&6.01 (br, s, 1H), 4.61 (br, s, 2H), 1.49 (s, 9H), 1.45 (s, 9H), 1.31 (s, 9H).
^{13}C NMR (126 MHz, Chloroform-*d*) δ 155.3, 150.6, 134.3, 129.0, 128.3, 125.5, 81.3, 54.0, 52.7, 34.6, 31.5, 28.4, 28.3.
The spectroscopic data obtained is consistent with data reported in literature.[6]

Di-*tert*-butyl 1-(4-(trifluoromethyl)benzyl)hydrazine-1,2-dicarboxylate (4d)

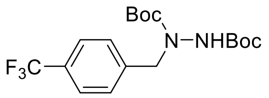

4d

Modified from General Procedure B, **2d** was added to the reaction tube before evacuation-refill sequence. The title compound was synthesized from methyl 1,4,5,6-tetraethyl-2-(4-(trifluoromethyl)benzyl)bicyclo[2.2.0]hex-5-ene-2-carboxylate (**2d**) (81.7 mg, 0.20 mmol) (reaction time = 12 h). The product was purified by silica gel column chromatography (PE:EtOAc = 40:1 – 25:1) to afford **4d** (56.5 mg, 72%) as a yellow oil.
^{1}H NMR (400 MHz, Chloroform-*d*) δ 7.58 (d, *J* = 8.0 Hz, 2H), 7.41 (d, *J* = 7.9 Hz, 2H), 6.30&6.14 (br, s, 1H), 4.68 (br, s, 2H), 1.47 (s, 9H), 1.44 (s, 9H).
^{13}C NMR (126 MHz, Chloroform-*d*) δ 155.2, 141.5, 128.8, 125.5 (q, *J* = 3.9 Hz), 124.3 (q, *J* = 271.9 Hz), 81.8, 54.4, 53.0, 28.31, 28.25.
The spectroscopic data obtained is consistent with data reported in literature.[7]

Di-*tert*-butyl 1-(4-bromobenzyl)hydrazine-1,2-dicarboxylate (4e)

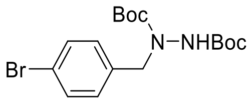

4e

Following General Procedure B, the title compound was synthesized from methyl 2-(4-bromobenzyl)-1,4,5,6-tetraethylbicyclo[2.2.0]hex-5-ene-2-carboxylate (**2e**) (83.9 mg, 73 μL, 0.20 mmol) (reaction time = 12 h). The product was purified by silica gel column chromatography (PE:EtOAc = 40:1 – 20:1) to afford **4e** (56.8 mg, 71%) as a yellow oil.

^{1}H NMR (400 MHz, Chloroform-*d*) δ 7.43 (d, *J* = 8.3 Hz, 2H), 7.16 (d, *J* = 7.9 Hz, 2H), 6.28&6.09 (br, s, 1H), 4.57 (br, s, 2H), 1.46 (s, 9H), 1.43 (s, 9H).
^{13}C NMR (126 MHz, Chloroform-*d*) δ 155.2, 136.4, 131.7, 130.3, 121.5, 81.6, 54.0, 52.6, 28.31, 28.25.
FTIR (cm^{-1}): 3316, 2978, 1702, 1488, 1392, 1367, 1253, 1151, 1013, 907, 852, 729.
HRMS (ESI) Calcd. For ($[C_{17}H_{25}BrN_2O_4]+H^+$): 401.1070; Found: 401.1069.
R$_f$ (PE:EtOAc = 5:1) = 0.50.

Di-*tert*-butyl 1-(4-methoxybenzyl)hydrazine-1,2-dicarboxylate (4f)

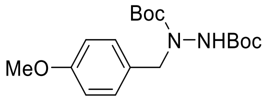

4f

Following General Procedure B, the title compound was synthesized from methyl 1,4,5,6-tetraethyl-2-(4-methoxybenzyl)bicyclo[2.2.0]hex-5-ene-2-carboxylate (**2f**) (74.1 mg, 74 μL, 0.20 mmol) (reaction time = 12 h). The product was purified by silica gel column chromatography (PE:EtOAc = 20:1 – 15:1) to afford **4f** (59.0 mg, 84%) as a yellow oil.
^{1}H NMR (400 MHz, Chloroform-*d*) δ 7.20 (d, *J* = 8.0 Hz, 2H), 6.84 (d, *J* = 8.6 Hz, 2H), 6.26&6.00 (br, s, 1H), 4.55 (br, s, 2H), 3.78 (s, 3H), 1.47 (s, 9H), 1.43 (s, 9H).
^{13}C NMR (126 MHz, Chloroform-*d*) δ 159.2, 155.3, 129.9, 129.3, 114.0, 81.3, 55.4, 53.7, 52.3, 28.34, 28.26.
The spectroscopic data obtained is consistent with data reported in literature.[6]

Di-*tert*-butyl 1-(naphthalen-2-ylmethyl)hydrazine-1,2-dicarboxylate (4g)

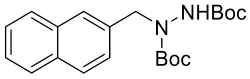

4g

Following General Procedure B, the title compound was synthesized from methyl 1,4,5,6-tetraethyl-2-(naphthalen-2-ylmethyl)bicyclo[2.2.0]hex-5-ene-2-carboxylate (**2g**) (78.1 mg, 77 μL, 0.20 mmol) (reaction time = 12 h). The product was purified by silica gel column chromatography (PE:EtOAc = 20:1) to afford **4g** (58.4 mg, 78%) as a yellow oil.
^{1}H NMR (400 MHz, Chloroform-*d*) δ 7.88 – 7.65 (m, 4H), 7.52 – 7.37 (m, 3H), 6.30&6.09 (br, s, 1H), 4.82 (br, s, 2H), 1.51 (s, 9H), 1.45 (s, 9H).
^{13}C NMR (126 MHz, Chloroform-*d*) δ 155.3, 134.7, 133.4, 132.9, 128.4, 127.9, 127.8, 126.2, 126.0, 81.4, 54.6, 53.2, 28.4, 28.3.
The spectroscopic data obtained is consistent with data reported in literature.[7]

Di-*tert*-butyl 1-allylhydrazine-1,2-dicarboxylate (4h)

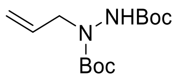

4h

Following General Procedure B, the title compound was synthesized from methyl 2-allyl-1,4,5,6-tetraethylbicyclo[2.2.0]hex-5-ene-2-carboxylate (**2h**) (58.1 mg, 61 μL, 0.20 mmol) (reaction time = 24 h). The product was purified by silica gel column chromatography (PE:EtOAc = 30:1 – 20:1) to afford **4h** (29.2 mg, 54%) as a white solid.

^{1}H NMR (400 MHz, Chloroform-*d*) δ 6.35&6.08 (br, s, 1H), 5.83 (ddt, *J* = 16.6, 10.1, 6.2 Hz, 1H), 5.24 – 5.07 (m, 2H), 4.04 (br, s, 2H), 1.46 (s, 9H), 1.45 (s, 9H).

^{13}C NMR (126 MHz, Chloroform-*d*) δ 155.3, 133.3, 117.7, 81.4, 52.4, 28.3.

The spectroscopic data obtained is consistent with data reported in literature.[8]

Di-*tert*-butyl 1-(4,4-dimethylpent-2-yn-1-yl)hydrazine-1,2-dicarboxylate (4i)

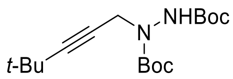

4i

Following General Procedure B, the title compound was synthesized from methyl 2-(4,4-dimethylpent-2-yn-1-yl)-1,4,5,6-tetraethylbicyclo[2.2.0]hex-5-ene-2-carboxylate (**2i**) (68.9 mg, 76 μL, 0.20 mmol) (reaction time = 12 h). The product was purified by silica gel column chromatography (PE:EtOAc = 60:1 – 25:1) to afford **4i** (37.6 mg, 58%) as a pale yellow solid.

^{1}H NMR (400 MHz, Chloroform-*d*) δ 6.44&6.14 (br, s, 1H), 4.20 (br, s, 2H), 1.46 (s, 18H), 1.19 (s, 9H).

^{13}C NMR (126 MHz, Chloroform-*d*) δ 154.9, 92.9, 81.7, 81.3, 73.0, 39.3, 31.1, 28.4, 28.3, 27.5.

FTIR (cm^{-1}): 3322, 2971, 2931, 1708, 1478, 1392, 1366, 1254, 1156, 1051, 859, 733.

HRMS (ESI) Calcd. For ([$C_{17}H_{30}N_2O_4$]+H^+): 327.2278; Found: 327.2277.

R_f (PE:EtOAc = 5:1) = 0.50.

Di-*tert*-butyl 1-isopropylhydrazine-1,2-dicarboxylate (4j)

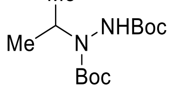

4j

Following General Procedure B, the title compound was synthesized from methyl 1,4,5,6-tetraethyl-2-isopropylbicyclo[2.2.0]hex-5-ene-2-carboxylate (**2j**) (58.4 mg, 64 μL, 0.20 mmol) (reaction time = 24 h). The product was purified by silica gel column chromatography (PE:EtOAc = 40:1 – 20:1) to afford **4j** (34.3 mg, 63%) as a white solid.

^{1}H NMR (400 MHz, Chloroform-*d*) δ 6.07&5.84 (br, s, 1H), 4.37 (br, s, 1H), 1.45 (s, 9H), 1.44 (s, 9H), 1.09 (d, *J* = 6.7 Hz, 6H).
^{13}C NMR (126 MHz, Chloroform-*d*) δ 155.9, 154.8, 81.5, 80.9, 48.5, 28.4, 28.3, 19.9
The spectroscopic data obtained is consistent with data reported in literature.[9]

Di-*tert*-butyl 1-(ethoxymethyl)hydrazine-1,2-dicarboxylate (4k)

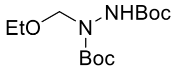

4k

Following General Procedure B, the title compound was synthesized from methyl 2-(ethoxymethyl)-1,4,5,6-tetraethylbicyclo[2.2.0]hex-5-ene-2-carboxylate (**2k**) (61.7 mg, 66 μL, 0.20 mmol) (reaction time = 24 h). The product was purified by silica gel column chromatography (PE:EtOAc = 40:1 – 20:1) to afford **4k** (17.6 mg, 30%) as a pale yellow oil.
^{1}H NMR (400 MHz, Chloroform-*d*) δ 6.45&6.17 (br, s, 1H), 4.85 (br, s, 2H), 3.57 (q, *J* = 7.1 Hz, 2H), 1.47 (s, 9H), 1.46 (s, 9H), 1.20 (t, *J* = 7.0 Hz, 3H).
^{13}C NMR (126 MHz, Chloroform-*d*) δ 154.9, 81.5, 79.4, 64.2, 28.32, 28.26, 15.2.
FTIR (cm^{-1}): 3313, 2978, 2931, 1714, 1456, 1368, 1247, 1150, 1093, 908, 732.
HRMS (ESI) Calcd. For ([$C_{13}H_{26}N_2O_5$]+Na^+): 313.1734; Found: 313.1730.
R_f (PE:EtOAc = 5:1) = 0.40.

II-C. Unsuccessful Examples

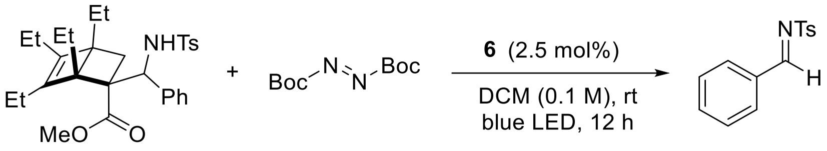

2l **3a** **9**,100%

Modified from General Procedure B, **2l** was added to the reaction tube before the evacuation-refill sequence. Sulfonimine **9** was synthesized from methyl 1,4,5,6-tetraethyl-2-(((4-methylphenyl)sulfonamido)(phenyl)methyl)bicyclo[2.2.0]hex-5-ene-2-carboxylate (**2l**) (101.9 mg, 0.20 mmol) (reaction time = 12 h). m(CH_2Br_2) = 48.1 mg.

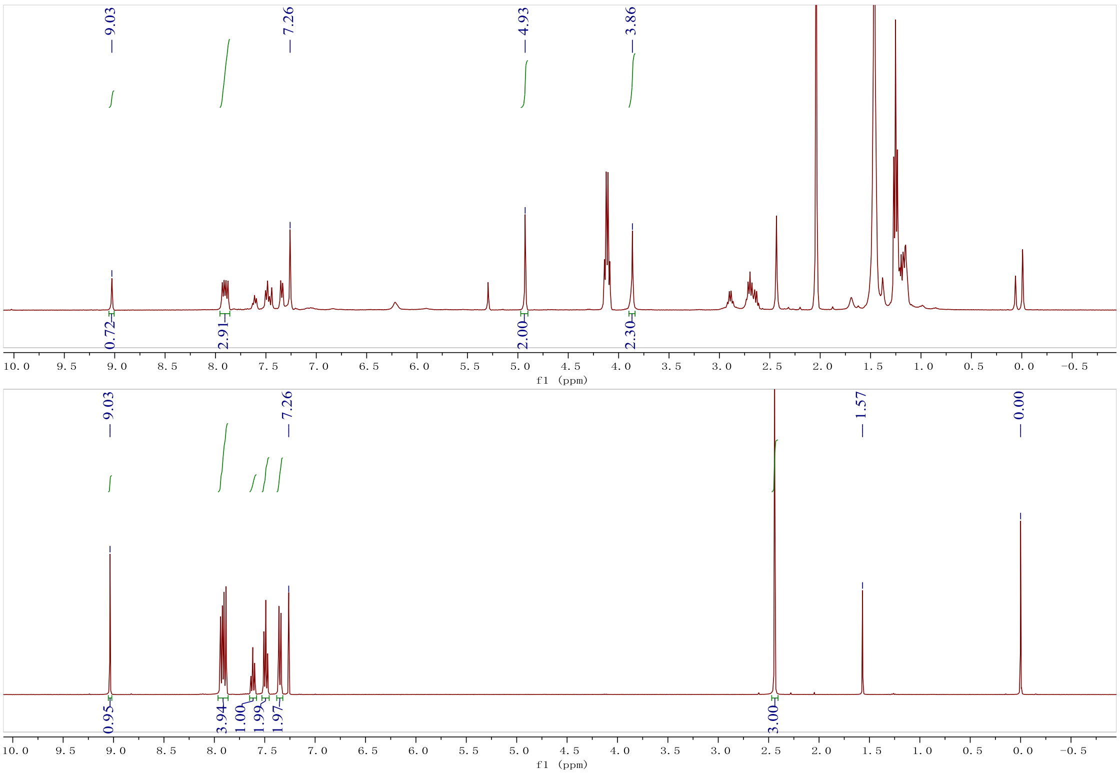

Fig. S2 NMR analysis of the reaction of **2l** with di-*tert*-butyl azodicarboxylate. Above: crude reaction mixture, below: sulfonimine **9**

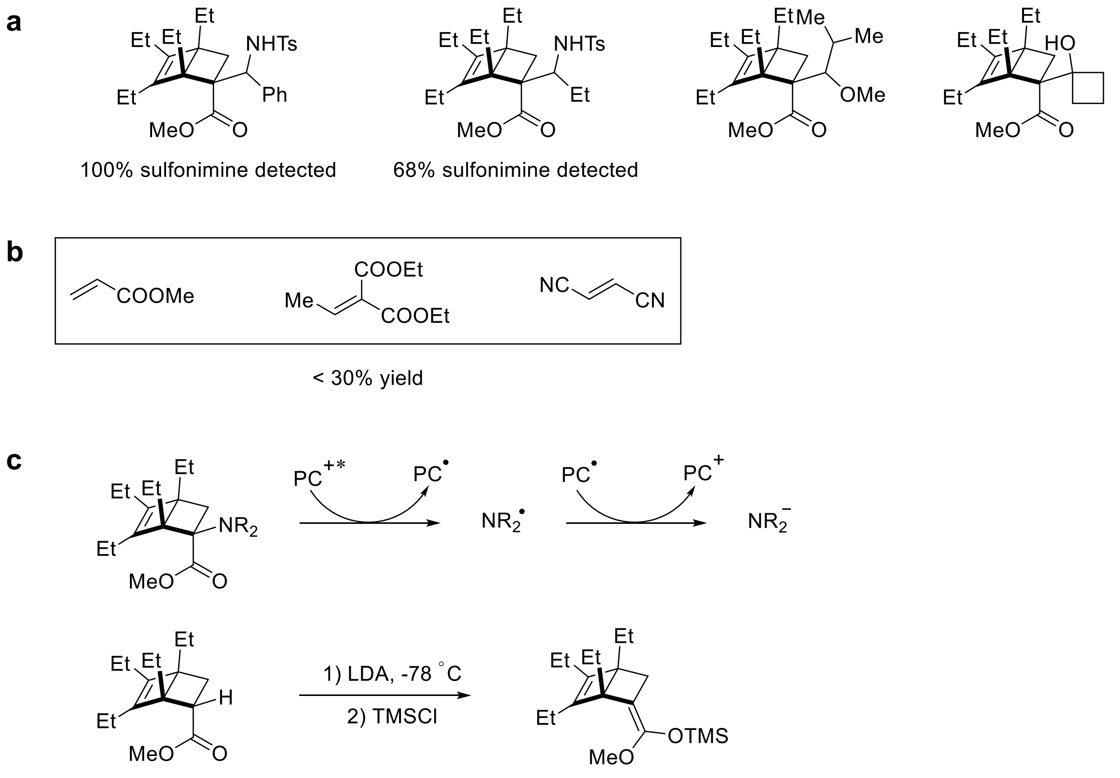

Fig. S3 Unsuccessful Examples. **a** Unsuitable BCHs due to cation releasing. **b** Less electron-deficient acceptors afforded low yields likely due to turnover-limiting radical reduction. **c** Potential problems with aminyl and silyl radical donors.

In general, heteroatom-based radicals, such as Si and N-centered radicals, remain challenging target for the current method for the following reasons:

1) While carbon-centered alkyl radicals are mostly nucleophilic, nitrogen-centered radicals are mostly electrophilic. Such a polarity difference would lead to a sluggish radical addition process to electron-deficient acceptors. More importantly, the reduction of N-centered radical species by the reduced form of the photocatalyst would outcompete radical addition and will eventually lead to BCH elimination without group transfer. Alternative redox systems are being pursuit to enable the use of electrophilic radicals.
2) Heteroatom-centered radicals that are nucleophilic and not susceptible to reduction in principle can engage in the transfer reaction similarly, for instance, silicon. Indeed, we attempted to synthesize silylated BCHs by reacting TMSCl with enolate. As expected, this reaction exclusively leads to silyl ether due to the strong Si-O affinity as well as steric hindrance. Alternative synthetic pathways are being investigated for the synthesis of silylated BCHs.

II-D. General Procedures for the Alkyl Transfer Reaction with 2a and Characterizations of 8b-j, 10k, 1

General Procedure C (Fig. 3b)**:** An oven-dried 20 mL re-sealable screw-cap tube equipped with a magnetic stir bar was charged with acridinium photocatalyst **6** (4.1 mg, 0.01 mmol, 5 mol%) and radical acceptors **3** (0.40 mmol, 2.0 equiv.). The tube was sealed. The tube was evacuated and backfilled with nitrogen. This sequence was repeated for a total of three times. To the tube were sequentially added **2a** (68.2 mg, 0.20 mmol, 70 μL, 1.0 equiv.), anhydrous dichloromethane (2.0 mL) and trifluoroacetic acid (0.04 mmol, 3.0 μL, 20 mol%) via syringe. The reaction mixture was stirred at room temperature under blue light irradiation for 24 or 36 h. The solvent was removed *in vacuo* and the residue was analyzed by ^{1}H NMR spectroscopy using dibromomethane as an internal standard. The solution in NMR tube was collected, concentrated *in vacuo* and purified by flash column chromatography to afford alkyl transfer product **8b-g**.

General Procedure D (Fig. 3c)**:** Modified from General Procedure C, the reaction mixture was stirred at room temperature under blue light irradiation for 12 h using 2.5 mol% acridinium photocatalyst to afford alkylation product **8h-j**.

General Procedure E (Fig. 3d)**:** Modified from General Procedure C, the reaction was performed at a 0.10 mmol scale. The product **10** was purified by preparative TLC.

2-(1,2-Diphenylethyl)malononitrile (8b)

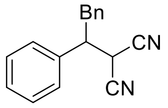

8b

Following General Procedure C, the title compound was synthesized from 2-benzylidenemalononitrile (**3b**) (61.7 mg, 0.40 mmol) (reaction time = 24 h). The product was purified by silica gel column chromatography (PE:EtOAc = 40:1 – 30:1) to afford **8b** (36.4 mg, 74%) as a yellow oil.

^{1}H NMR (400 MHz, Chloroform-*d*) δ 7.44 – 7.35 (m, 5H), 7.34 – 7.23 (m, 3H), 7.20 – 7.15 (m, 2H), 3.83 (d, *J* = 5.1 Hz, 1H), 3.45 (td, *J* = 7.8, 5.2 Hz, 1H), 3.25 (dd, *J* = 7.9, 3.4 Hz, 2H).

^{13}C NMR (101 MHz, Chloroform-*d*) δ 136.7, 136.5, 129.3, 129.23, 129.15, 129.0, 128.1, 127.7, 112.2, 111.6, 48.4, 38.6, 28.6.

The spectroscopic data obtained is consistent with data reported in literature.[10]

2-(2-Phenyl-1-(*p*-tolyl)ethyl)malononitrile (8c)

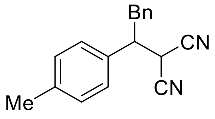

8c

Following General Procedure C, the title compound was synthesized from 2-(4-methylbenzylidene)malononitrile (**3c**) (67.3 mg, 0.40 mmol) (reaction time = 24 h). The product was purified by silica gel column chromatography (PE:DCM = 4:1 – 3:1) to afford **8c** (37.2 mg, 71%) as a yellow oil.

^{1}H NMR (400 MHz, Chloroform-*d*) δ 7.28 – 7.06 (m, 9H), 3.73 (d, *J* = 5.1 Hz, 1H), 3.35 (td, *J* = 7.8, 5.1 Hz, 1H), 3.23 – 3.09 (m, 2H), 2.28 (s, 3H).

^{13}C NMR (101 MHz, Chloroform-*d*) δ 139.0, 136.9, 133.5, 130.0, 129.2, 129.0, 128.0, 127.6, 112.3, 111.6, 48.1, 38.6, 28.8, 21.3.

The spectroscopic data obtained is consistent with data reported in literature.[10]

2-(2-Phenyl-1-(4-(trifluoromethyl)phenyl)ethyl)malononitrile (8d)

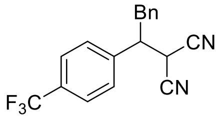

8d

Following General Procedure C, the title compound was synthesized from 2-(4-(trifluoromethyl)benzylidene)malononitrile (**3d**) (88.9 mg, 0.40 mmol) (reaction time = 24 h). The product was purified by silica gel column chromatography (PE:DCM = 4:1 – 3:1) to afford **8d** (43.9 mg, 70%) as a white solid.

^{1}H NMR (400 MHz, Chloroform-*d*) δ 7.70 (d, *J* = 8.0 Hz, 2H), 7.55 (d, *J* = 8.0 Hz, 2H), 7.40 – 7.28 (m, 3H), 7.20 (d, *J* = 7.2 Hz, 2H), 3.89 (d, *J* = 5.1 Hz, 1H), 3.60 – 3.50 (m, 1H), 3.36 – 3.21 (m, 2H).

^{13}C NMR (101 MHz, Chloroform-*d*) δ 140.4, 136.0, 131.4 (q, *J* = 32.8 Hz), 129.4, 129.0, 128.7, 128.0, 126.3 (q, *J* = 3.7 Hz), 123.9 (q, *J* = 272.3 Hz), 111.8, 111.2, 48.1, 38.4, 28.3.

The spectroscopic data obtained is consistent with data reported in literature.[10]

2-(1-(4-Chlorophenyl)-2-phenylethyl)malononitrile (8e)

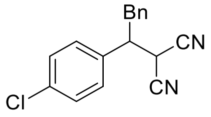

8e

Following General Procedure C, the title compound was synthesized from 2-(4-chlorobenzylidene)malononitrile (**3e**) (75.4 mg, 0.40 mmol) (reaction time = 24 h). The product was purified by silica gel column chromatography (PE:DCM = 4:1) to afford **8e** (38.0 mg, 68%) as a pale yellow solid.

^{1}H NMR (400 MHz, Chloroform-*d*) δ 7.42 – 7.24 (m, 7H), 7.20 – 7.14 (m, 2H), 3.83 (d, *J* = 5.1 Hz, 1H), 3.44 (td, *J* = 7.9, 5.1 Hz, 1H), 3.29 – 3.16 (m, 2H).
^{13}C NMR (101 MHz, Chloroform-*d*) δ 136.3, 135.2, 134.9, 129.53, 129.50, 129.3, 129.0, 127.8, 112.0, 111.3, 47.8, 38.5, 28.5.
FTIR (cm^{-1}): 3030, 2901, 2255, 1599, 1494, 1455, 1413, 1094, 1030, 909, 837, 734, 701.
HRMS (ESI) Calcd. For ([$C_{17}H_{13}ClN_2$]+Na^+): 303.0659; Found: 303.0656.
R$_f$ (PE:DCM = 1:1) = 0.30.

Methyl 4-(1,1-dicyano-3-phenylpropan-2-yl)benzoate (8f)

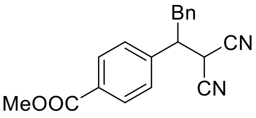

8f

Following General Procedure C, the title compound was synthesized from methyl 4-(2,2-dicyanovinyl)benzoate (**3f**) (84.9 mg, 0.40 mmol) (reaction time = 24 h). The product was purified by silica gel column chromatography (PE:DCM = 1:1 – 2:3) to afford **8f** (50.7 mg, 83%) as a yellow oil.
^{1}H NMR (400 MHz, Chloroform-*d*) δ 8.08 (d, *J* = 8.2 Hz, 2H), 7.46 (d, *J* = 8.1 Hz, 2H), 7.35 – 7.23 (m, 3H), 7.19 – 7.12 (m, 2H), 3.95 – 3.88 (m, 4H), 3.53 (td, *J* = 7.8, 5.4 Hz, 1H), 3.35 – 3.19 (m, 2H).
^{13}C NMR (101 MHz, Chloroform-*d*) δ 166.5, 141.3, 136.2, 131.0, 130.5, 129.3, 129.0, 128.3, 127.8, 111.9, 111.3, 52.4, 48.2, 38.5, 28.3.
The spectroscopic data obtained is consistent with data reported in literature.[10]

2-(1-Phenylbutan-2-yl)malononitrile (8g)

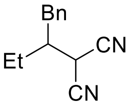

8g

Modified from General Procedure C, 2-propylidenemalononitrile (**3g**) was added after evacuation-refill sequence. The title compound was synthesized from 2-propylidenemalononitrile (**3g**) (42.5 mg, 46 μL, 0.40 mmol) (reaction time = 36 h). The product was purified by silica gel column chromatography (PE:DCM = 4:1 – 3:1) to afford **8g** (21.5 mg, 54%) as a yellow oil.
^{1}H NMR (400 MHz, Chloroform-*d*) δ 7.40 – 7.27 (m, 3H), 7.23 – 7.16 (m, 2H), 3.62 (d, *J* = 4.0 Hz, 1H), 3.04 (dd, *J* = 14.2, 5.8 Hz, 1H), 2.66 (dd, *J* = 14.2, 9.3 Hz, 1H), 2.25 – 2.15 (m, 1H), 1.83 (dtd, *J* = 14.9, 7.4, 5.4 Hz, 1H), 1.73 – 1.60 (m, 1H), 1.13 (t, *J* = 7.4 Hz, 3H).

^{13}**C NMR** (101 MHz, Chloroform-*d*) δ 137.1, 129.3, 129.0, 127.6, 112.6, 111.7, 44.4, 37.2, 26.5, 24.5, 11.4.
The spectroscopic data obtained is consistent with data reported in literature.[10]

2,6-Di-*tert*-butyl-4-(1,2-diphenylethyl)phenol (8h)

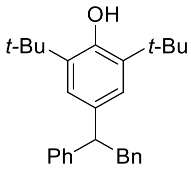

8h

Following General Procedure D, the title compound was synthesized from 4-benzylidene-2,6-di-*tert*-butylcyclohexa-2,5-dien-1-one (**3h**) (117.8 mg, 0.40 mmol). The product was purified by silica gel column chromatography (PE:DCM = 25:1) to afford **8h** (40.2 mg, 52%) as a white solid.
^{1}**H NMR** (400 MHz, Chloroform-*d*) δ 7.27 – 7.07 (m, 8H), 6.98 – 6.91 (m, 4H), 5.01 (s, 1H), 4.12 (t, *J* = 7.8 Hz, 1H), 3.36 – 3.22 (m, 2H), 1.37 (s, 18H).
^{13}**C NMR** (101 MHz, Chloroform-*d*) δ 152.1, 144.9, 140.9, 135.5, 135.2, 129.3, 128.3, 128.3, 128.1, 126.1, 125.9, 124.7, 53.4, 43.1, 34.5, 30.5.
FTIR (cm^{-1}): 3639, 3026, 2954, 1601, 1435, 1361, 1317, 1233, 1155, 1121, 698, 529.
HRMS (ESI) Calcd. For ([$C_{28}H_{34}O$]+Na^{+}): 409.2502; Found: 409.2497.
R$_f$ (PE:DCM = 5:1) = 0.55.

2,6-Di-*tert*-butyl-4-(1-(4-chlorophenyl)-2-phenylethyl)phenol (8i)

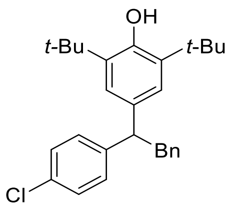

8i

Following General Procedure D, the title compound was synthesized from 2,6-di-*tert*-butyl-4-(4-chlorobenzylidene)cyclohexa-2,5-dien-1-one (**3i**) (131.6 mg, 0.40 mmol). The product was purified by silica gel column chromatography (PE – PE:DCM = 200:1) to afford **8i** (37.4 mg, 44%) as a pale yellow oil.
^{1}**H NMR** (400 MHz, Chloroform-*d*) δ 7.25 – 7.11 (m, 7H), 7.02 – 6.92 (m, 4H), 5.06 (s, 1H), 4.13 (t, *J* = 7.7 Hz, 1H), 3.29 (d, *J* = 7.8 Hz, 2H), 1.41 (s, 18H).
^{13}**C NMR** (101 MHz, Chloroform-*d*) δ 152.3, 143.5, 140.4, 135.8, 134.8, 131.8, 129.7, 129.3, 128.4, 128.2, 126.0, 124.5, 52.7, 42.9, 34.5, 30.5.
FTIR (cm^{-1}): 3637, 3026, 2955, 1490, 1434, 1234, 1154, 1092, 1014, 907, 732, 699.

HRMS (ESI) Calcd. For ($[C_{28}H_{33}ClO] - H^+$): 419.2147; Found: 419.2143.
$\mathbf{R_f}$ (PE:DCM = 5:1) = 0.55.

4-(1-(4-Bromophenyl)-2-phenylethyl)-2,6-di-*tert*-butylphenol (8j)

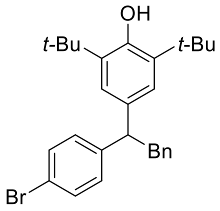

8j

Following General Procedure D, the title compound was synthesized from 4-(4-bromobenzylidene)-2,6-di-*tert*-butylcyclohexa-2,5-dien-1-one (**3j**) (149.3 mg, 0.40 mmol). The product was purified by silica gel column chromatography (PE:DCM = 200:1 – 100:1) to afford **8j** (40.1 mg, 43%) as a pale yellow oil.
^{1}**H NMR** (400 MHz, Chloroform-*d*) δ 7.38 (d, *J* = 8.4 Hz, 2H), 7.23 – 7.12 (m, 3H), 7.09 (d, *J* = 8.4 Hz, 2H), 7.02 – 6.91 (m, 4H), 5.08 (s, 1H), 4.11 (t, *J* = 7.8 Hz, 1H), 3.29 (d, *J* = 8.7 Hz, 2H), 1.41 (s, 18H).
^{13}**C NMR** (101 MHz, Chloroform-*d*) δ 152.3, 143.9, 140.4, 135.7, 134.7, 131.4, 130.0, 129.3, 128.2, 126.0, 124.5, 119.8, 52.8, 42.8, 34.5, 30.4.
The spectroscopic data obtained is consistent with data reported in literature.[11]

Benzyl 2-methylene-4-phenylbutanoate (10k)

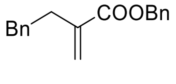

10k

Following General Procedure E, the title compound was synthesized from benzyl 2-((phenylsulfonyl)methyl)acrylate (**3k**) (63.3 mg, 0.20 mmol) (reaction time = 48 h). The product was purified by purified by preparative TLC (SiO_2, PE:EtOAc = 15:1) to afford **10k** (8.7 mg, 33%) as a colorless oil.
^{1}**H NMR** (400 MHz, Chloroform-*d*) δ 7.41 – 7.22 (m, 7H), 7.21 – 7.12 (m, 3H), 6.20 (s, 1H), 5.52 (s, 1H), 5.20 (s, 2H), 2.78 (dd, *J* = 9.4, 6.3 Hz, 2H), 2.63 (t, *J* = 7.9 Hz, 2H).
^{13}**C NMR** (101 MHz, Chloroform-d) δ 167.0, 141.5, 139.9, 136.2, 128.7, 128.6, 128.5, 128.3, 128.2, 126.1, 126.0, 66.6, 35.0, 34.1.
The spectroscopic data obtained is consistent with data reported in literature.[12]

2-Methylene-4-phenylbutanenitrile (10l)

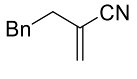

10l

Following General Procedure E, the title compound was synthesized from 2-((phenylsulfonyl)methyl)acrylonitrile (**31**) (41.4 mg, 0.20 mmol) (reaction time = 24 h). The product was purified by purified by preparative TLC (SiO_2, PE:EtOAc = 10:1) to afford **101** (6.7 mg, 43%) as a pale yellow oil.

^{1}H NMR (400 MHz, Chloroform-*d*) δ 7.35 – 7.26 (m, 2H), 7.25 – 7.14 (m, 3H), 5.83 (s, 1H), 5.64 (s, 1H), 2.88 (t, *J* = 7.7 Hz, 2H), 2.56 (t, *J* = 7.8 Hz, 2H).

^{13}C NMR (101 MHz, Chloroform-*d*) δ 139.7, 131.2, 128.7, 128.6, 126.6, 122.4, 118.7, 36.6, 34.0.

The spectroscopic data obtained is consistent with data reported in literature.[13]

II-E. Procedures for Derivatization of Alkylation Products and Characterizations of 11-13

Ethyl 1-benzyl-1H-pyrazole-4-carboxylate (11)

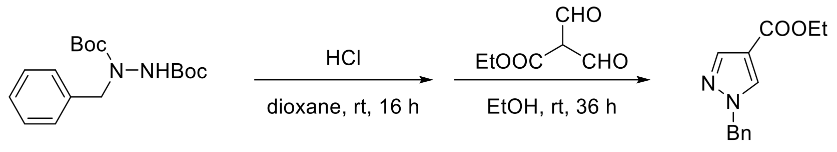

4a **11**, 76%

Adapted from a literature procedure,[14] HCl (4 M in dioxane, 0.85 mL, 3.4 mmol, 24.0 equiv.) was added to di-*tert*-butyl 1-benzylhydrazine-1,2-dicarboxylate (**4a**) (45.7 mg, 0.142 mmol, 1.0 equiv.) and the reaction mixture was stirred at room temperature for 16 h. The solvent was removed *in vacuo* and to the residue was added EtOH (0.70 mL) and ethyl 2-formyl-3-oxopropanoate (23.2 mg, 20 μL, 1.1 equiv.). The reaction mixture was stirred at room temperature for 36 h. Then the solvent was removed *in vacuo* and the residue was purified by preparative TLC (SiO_2, PE:EtOAc = 3:1) to afford **11** (24.9 mg, 76%) as a yellow oil.
^{1}**H NMR** (400 MHz, Chloroform-*d*) δ 7.94 (s, 1H), 7.86 (s, 1H), 7.46 – 7.16 (m, 5H), 5.30 (s, 2H), 4.27 (q, *J* = 7.1 Hz, 2H), 1.32 (t, *J* = 7.1 Hz, 3H).
^{13}**C NMR** (101 MHz, Chloroform-*d*) δ 163.1, 141.3, 135.4, 132.7, 129.1, 128.6, 128.1, 115.7, 60.3, 56.6, 14.5.
The spectroscopic data obtained is consistent with data reported in literature.[15]

Methyl 4-(1-methoxy-1-oxo-3-phenylpropan-2-yl)benzoate (12)

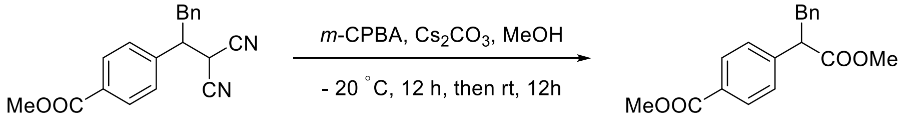

8f **12**, 77%

Adapted from a literature procedure,[16] at -20 °C, *m*-CPBA (75% purity, 53.5 mg, 0.233 mmol, 1.5 equiv.) was added to a solution of methyl 4-(1,1-dicyano-3-phenylpropan-2-yl)benzoate (**8f**) (47.2 mg, 0.155 mmol, 1.0 equiv.) in MeOH (1.0 mL). After 12 hours, the reaction was warmed to room temperature and stirred for another 12 hours. The reaction mixture was filtered through a short column of SiO_2 and eluted with PE:EtOAc = 1:2. The filtrate was concentrated *in vacuo* and the residue was purified by silica gel column chromatography (PE:EtOAc = 25:1 – 20:1) to afford **12** (35.4 mg, 77%) as a colorless oil.
^{1}**H NMR** (400 MHz, Chloroform-*d*) δ 7.97 (d, *J* = 8.1 Hz, 2H), 7.36 (d, *J* = 8.1 Hz, 2H), 7.25 – 7.14 (m, 3H), 7.12 – 7.05 (m, 2H), 3.96 – 3.86 (m, 4H), 3.62 (s, 3H), 3.43 (dd, *J* = 13.7, 8.3 Hz, 1H), 3.04 (dd, *J* = 13.7, 7.3 Hz, 1H).

^{13}**C NMR** (101 MHz, Chloroform-*d*) δ 173.3, 166.9, 143.7, 138.5, 130.0, 129.4, 129.0, 128.5, 128.2, 126.6, 53.7, 52.3, 52.2, 39.7.
FTIR (cm^{-1}): 2952, 1719, 1610, 1435, 1278, 1182, 1161, 1111, 1020, 906, 729, 700.
HRMS (ESI) Calcd. For ([$C_{18}H_{18}O_4$]+H^+): 299.1278; Found: 299.1273.
$\mathbf{R_f}$ (PE:EtOAc = 10:1) = 0.25.

3-Phenyl-1-(pyrrolidin-1-yl)-2-(*p*-tolyl)propan-1-one (13)

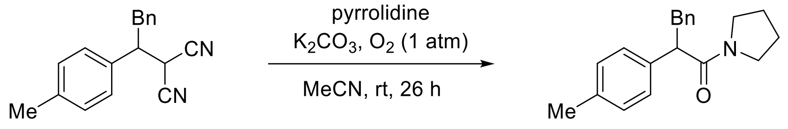

8c **13**, 68%

Adapted from a literature procedure,[16] Under O_2 atmosphere, pyrrolidine (22 μL, 0.26 mmol, 2.0 equiv.) was added to a solution of 2-(2-phenyl-1-(*p*-tolyl)ethyl)malononitrile (**8c**) (33.9 mg, 0.13 mmol, 1.0 equiv.) and K_2CO_3 (35.9 mg, 0.26 mmol, 2.0 equiv.) in MeCN (1.4 mL). The resulting mixture was stirred at room temperature for 26 h. Then the reaction mixture was filtered through a short column of SiO_2 and eluted with EtOAc. The filtrate was concentrated *in vacuo* and the residue was purified by silica gel column chromatography (PE:EtOAc = 10:1 – 6:1) to afford **13** (26.0 mg, 68%) as a colorless oil.
^{1}**H NMR** (400 MHz, Chloroform-*d*) δ 7.25 – 7.05 (m, 9H), 3.79 (dd, *J* = 8.2, 6.5 Hz, 1H), 3.54 – 3.31 (m, 3H), 3.29 – 3.09 (m, 2H), 2.92 (dd, *J* = 13.5, 6.5 Hz, 1H), 2.32 (s, 3H), 1.82 – 1.63 (m, 4H).
^{13}**C NMR** (101 MHz, Chloroform-*d*) δ 171.3, 140.4, 136.6, 129.4, 129.3, 128.23, 128.17, 126.1, 52.8, 46.3, 46.0, 41.1, 26.1, 24.2, 21.2.
FTIR (cm^{-1}): 2973, 1628, 1438, 905, 725, 700, 648, 555.
HRMS (ESI) Calcd. For ([$C_{20}H_{23}NO$]+H^+): 294.1852; Found: 294.1860.
$\mathbf{R_f}$ (PE:EtOAc = 4:1) = 0.20.

III. Cyclic Voltammetry and Stern-Volmer Quenching Experiments

A cyclic voltammetry experiment was performed on a CH Instruments 630E potentiostat using a glassy carbon working electrode, Ag/AgCl in 3 M KCl reference electrode, and a Pt counter electrode. The voltammogram was taken in an anhydrous 100 mM MeCN solution of tetrabutylammonium hexafluorophosphate containing 10 mM of **2a** at room temperature under a N_2 atmosphere. The scan rate was 0.1 V/s.

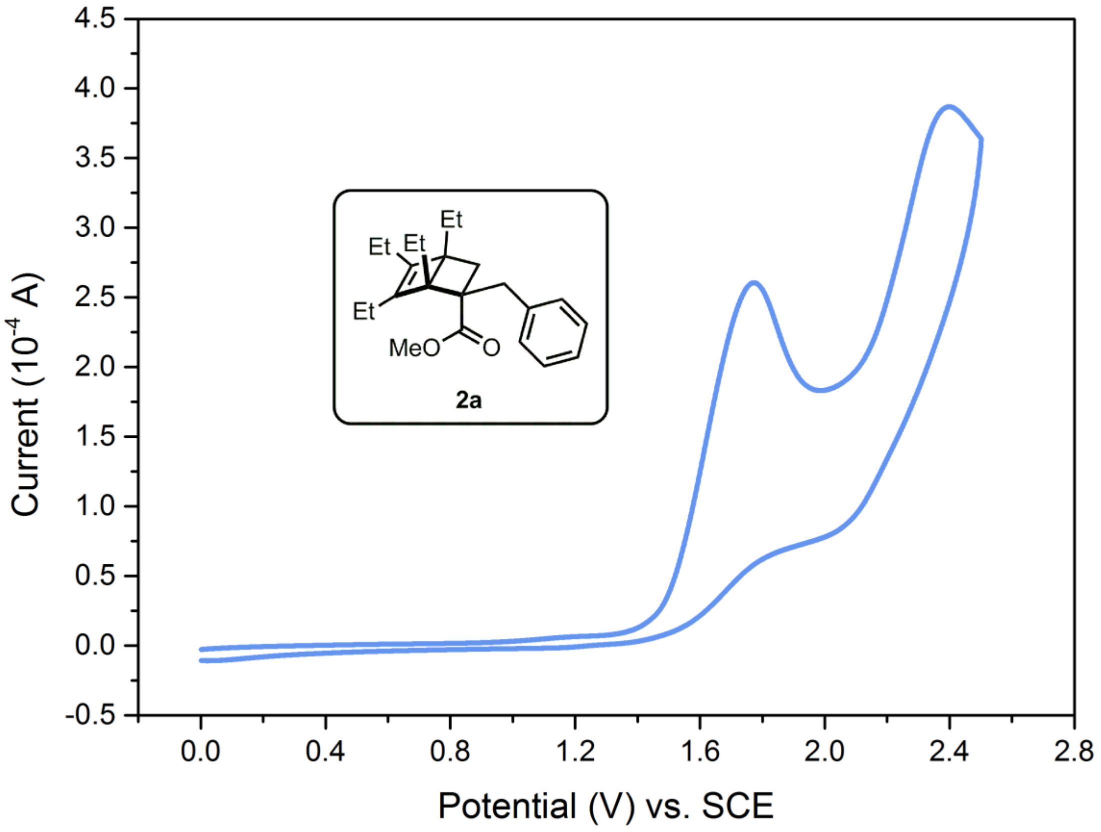

Fig. S4 Cyclic voltammetry of methyl 2-benzyl-1,4,5,6-tetraethylbicyclo[2.2.0]hex-5-ene-2-carboxylate (**2a**)

$E_{1/2}{}^{ox}$ (**2a**) = +1.59 V vs. SCE in MeCN

$E_{1/2}{}^{ox'}$ (**2a**) = +2.11 V vs. SCE in MeCN

Emission intensities were recorded on a Shimadzu RF-6000 Fluorescence Spectrometer for all experiments. All 9-mesityl-10-methylacridinium perchlorate solutions (0.50 mM in DCM) were excited at 450 nm and the emission intensity at 510 nm was collected at room temperature under a N_2 atmosphere. Samples were prepared by adding appropriate amount of **2a** (0 – 49 μL) to a 3.6 mL 0.50 mM solution of 9-mesityl-10-methylacridinium perchlorate in anhydrous DCM.

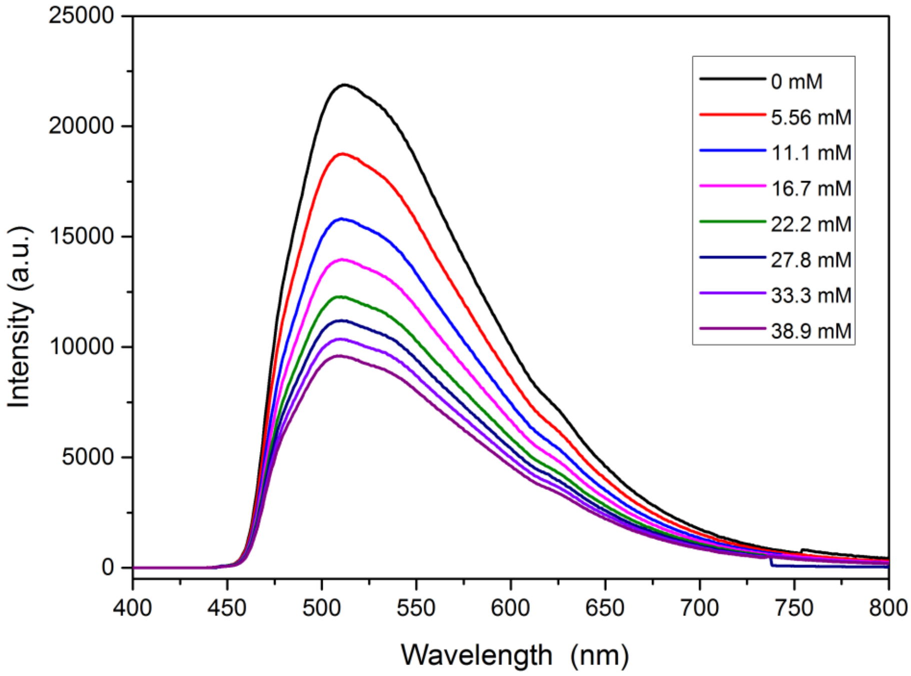

Fig. S5 Quenching the excited state of 9-mesityl-10-methylacridinium perchlorate with varying concentrations of **2a**

Table S1. Fluorescence Intensity at 510 nm with different concentrations of **2a**

Concentration of **2a** /mM	Intensity /a.u.
0	21815
5.56	18737
11.1	15811
16.7	13969
22.2	12293
27.8	11198
33.3	10364
38.9	9587

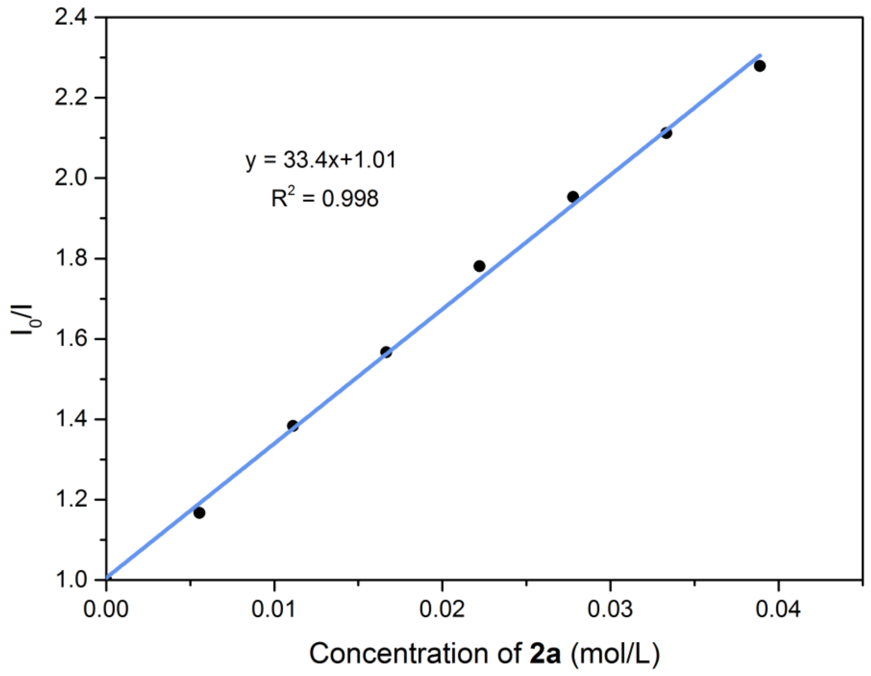

Fig. S6 Steady State Stern-Volmer linear fitting

IV. Light-Triggered Responses of 2m

IV-A. Independent Synthesis of 14 and Neutral 1,3-CHD (S1)

2-((2,3,4,5-Tetraethyl-1-(methoxycarbonyl)cyclohexa-2,4-dien-1-yl)methyl)quinolin-1-ium 2,2,2-trifluoroacetate (14)

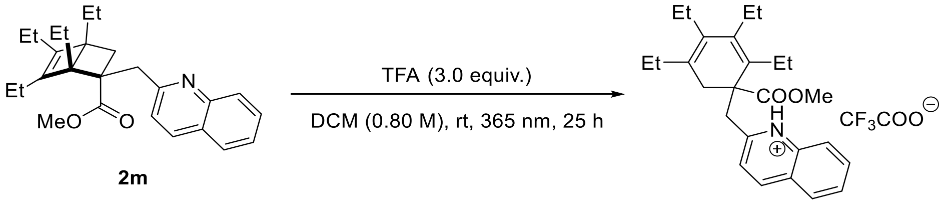

Procedure: An oven-dried 20 mL re-sealable screw-cap tube equipped with a magnetic stir bar was sealed. The tube was evacuated and backfilled with nitrogen. This sequence was repeated for a total of three times. To the tube were added a solution of **2m** (1.63 mmol, 0.64 g, 1.0 equiv.) in anhydrous dichloromethane (2.0 mL) and TFA (4.90 mmol, 0.36 mL, 3.0 equiv.) via syringe. The reaction mixture was stirred at room temperature under 365 nm irradiation for 25 h. The reaction mixture was concentrated *in vacuo* and purified by silica gel column chromatography (DCM:CH_3OH = 100:1 – 50:1) to afford **14** (0.34 g, 42%) as a yellow oil. **14** was dissolved in *n*-hexane/DCM and evaporation of the solvent affords **14** as a yellow solid.

^{1}H NMR (400 MHz, Chloroform-*d*) δ 8.70 (d, *J* = 8.5 Hz, 1H), 8.44 (d, *J* = 8.5 Hz, 1H), 8.01 – 7.91 (m, 2H), 7.76 (t, *J* = 7.7 Hz, 1H), 7.46 (d, *J* = 8.5 Hz, 1H), 3.75 (s, 3H), 3.73 (d, *J* = 12.7 Hz, 1H), 3.54 (d, *J* = 13.3 Hz, 1H), 2.96 (d, *J* = 16.8 Hz, 1H), 2.35 – 2.00 (m, 7H), 1.97 – 1.85 (m, 1H), 1.81 – 1.68 (m, 1H), 1.05 (t, *J* = 7.7 Hz, 3H), 1.00 (t, *J* = 7.7 Hz, 3H), 0.94 (t, *J* = 7.5 Hz, 3H), 0.73 (t, *J* = 7.5 Hz, 3H).

^{13}C NMR (101 MHz, Chloroform-*d*) δ 175.8, 159.0, 142.1, 139.4, 138.1, 133.8, 133.1, 132.1, 130.0, 129.1, 128.0, 127.1, 123.6, 122.6, 53.5, 52.6, 37.8, 37.1, 26.5, 24.0, 21.6, 20.2, 15.5, 15.1, 14.8, 12.7.

FTIR (cm^{-1}): 2968, 1727, 1670, 1387, 1199, 1136, 905, 729, 649.

HRMS (ESI) Calcd. For ([$C_{26}H_{33}NO_2$]+H^+): 392.2584; Found: 392.2583.

R$_f$ (DCM:CH_3OH = 40:1) = 0.65.

Methyl 2,3,4,5-tetraethyl-1-(quinolin-2-ylmethyl)cyclohexa-2,4-diene-1-carboxylate (S1)

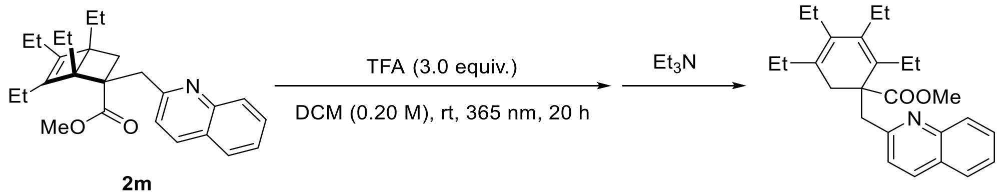

2m

S1, 58%

Procedure: An oven-dried 20 mL re-sealable screw-cap tube equipped with a magnetic stir bar was sealed. The tube was evacuated and backfilled with nitrogen. This sequence was repeated for a total of three times. To the tube were added **2m** (0.10 mmol, 38 μL, 1.0 equiv.), TFA (0.30 mmol, 22 μL, 3.0 equiv.) and anhydrous dichloromethane (0.50 mL) via syringe. The reaction mixture was stirred at room temperature under 365 nm irradiation for 20 h. The reaction mixture was quenched with Et_3N. The solvent was removed *in vacuo* and the residue was analyzed by ^{1}H NMR spectroscopy using dibromomethane as an internal standard. The solution in NMR tube was collected, concentrated *in vacuo* and purified by preparative TLC (SiO_2, DCM:EtOAc = 50:1) to afford product **S1** (22.8 mg, 58%) as a pale yellow oil .

^{1}H NMR (400 MHz, Chloroform-*d*) δ 7.96 (d, *J* = 8.4 Hz, 2H), 7.73 (d, *J* = 8.1 Hz, 1H), 7.64 (t, *J* = 7.7 Hz, 1H), 7.45 (t, *J* = 7.5 Hz, 1H), 7.21 (d, *J* = 8.4 Hz, 1H), 3.70 (s, 3H), 3.48 (d, *J* = 14.5 Hz, 1H), 3.14 (d, *J* = 14.5 Hz, 1H), 2.74 (d, *J* = 16.4 Hz, 1H), 2.38 (d, *J* = 16.4 Hz, 1H), 2.33 – 2.01 (m, 8H), 1.05 (t, *J* = 7.4 Hz, 3H), 1.00 (t, *J* = 7.4 Hz, 3H), 0.91 (t, *J* = 7.4 Hz, 3H), 0.78 (t, *J* = 7.4 Hz, 3H).

^{13}C NMR (101 MHz, Chloroform-*d*) δ 176.8, 160.0, 147.7, 136.0, 135.3, 133.6, 133.4, 131.3, 129.4, 129.0, 127.5, 126.6, 125.7, 123.1, 51.7, 51.6, 41.1, 35.0, 26.5, 22.9, 21.6, 20.4, 15.5, 15.3, 15.2, 13.0.

FTIR (cm^{-1}): 2962, 2931, 2871, 1727, 1600, 1503, 1426, 1221, 1201, 1173, 1046, 756.

HRMS (ESI) Calcd. For ($[C_{26}H_{33}NO_2]+H^+$): 392.2584; Found: 392.2595.

R_f (DCM:EtOAc = 20:1) = 0.45.

IV-B. UV-Vis Absorption and Excitation Spectra of 14

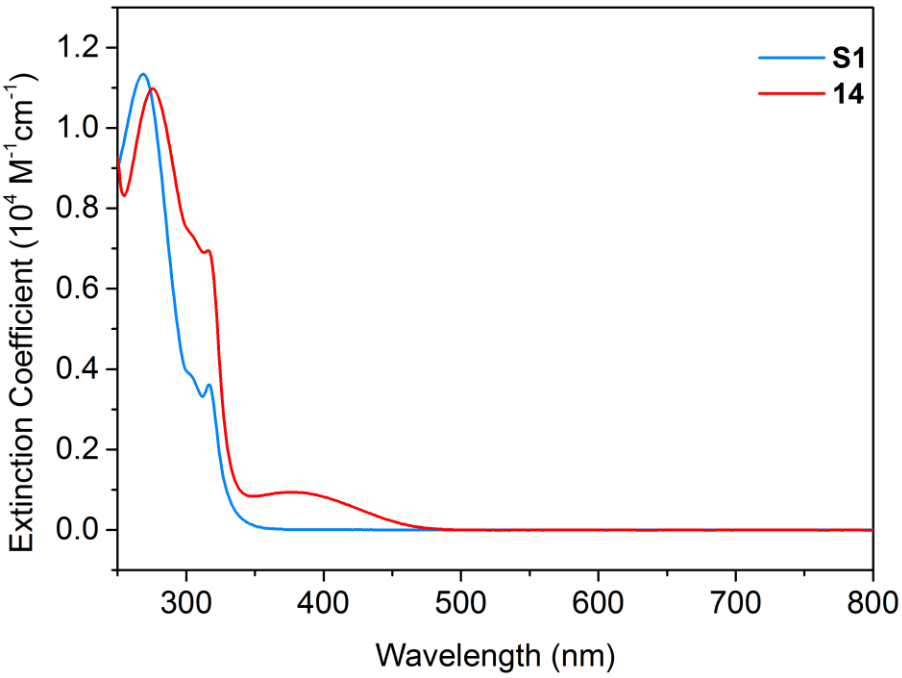

Fig. S7 UV-Vis spectra of a 1.0×10^{-4} mol/L solution of **S1** and **14** in dichloromethane, respectively

ε (276 nm) = 1.1×10^4 M^{-1} cm^{-1}, ε (377 nm) = 9.4×10^2 M^{-1} cm^{-1}

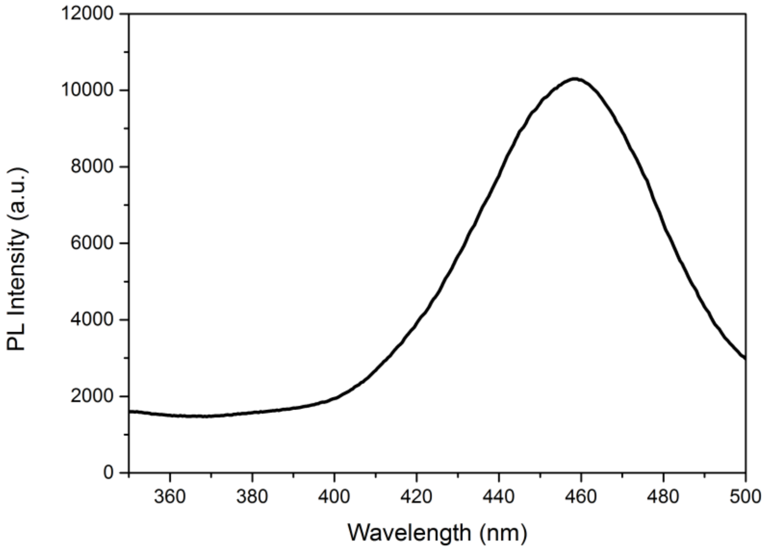

Fig. S8 Excitation spectrum of a 7.3 mM solution of **14** in dichloromethane/*n*-hexane (v/v = 10/90). Emission wavelength: 546 nm.

IV-C. Fluorescence Sensing for $POCl_3$ and Emission Spectrum of 2-Methyl Quinolinium

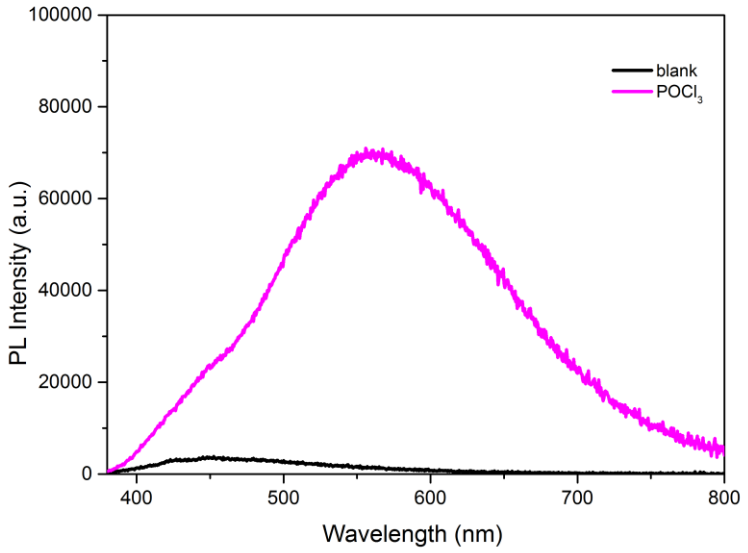

Fig. S9 Fluorescence spectrum of **2m** (film) after $POCl_3$ exposure (irradiated at 365 nm).

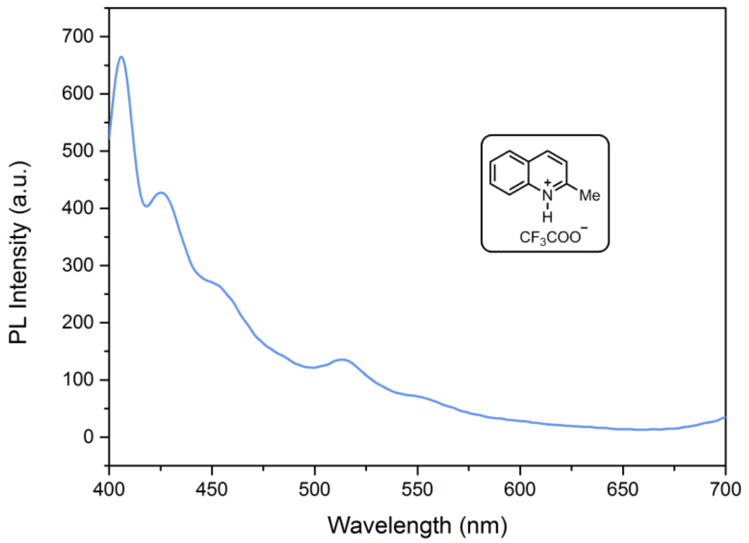

Fig. S10 Emission spectrum of 2-methyl quinolinium. Concentration: 7.3 mM in dichloromethane/*n*-hexane (v/v = 10/90). Excitation wavelength: 365 nm.

IV-D. Excitation Spectrum of 2m (Film) Exposed to TFA

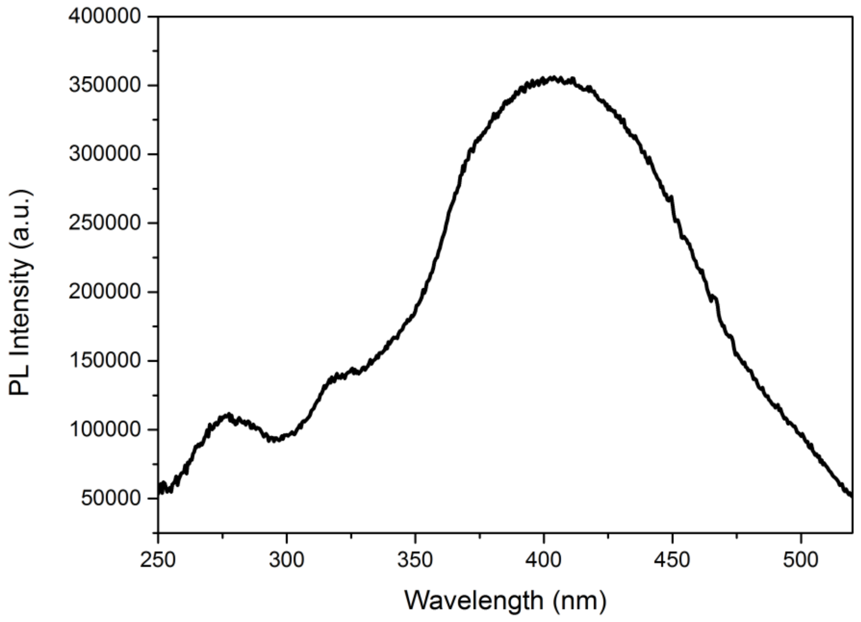

Fig. S11 Excitation spectrum of a film of **2m** exposed to TFA vapor after irradiation at 365 nm. Emission wavelength: 548 nm

V. DFT Studies

Computational Methods. All DFT calculations were performed with the Gaussian 09 program.[17] Geometry optimizations were carried out in the gas phase using B3LYP functional[18] and Pople basis set[19] for neutral radical intermediates. The SMD solvation model[20] was employed for ionic species during geometry optimizations. The vibrational frequencies were computed at the same level to check whether each optimized structure is an energy minimum (no imaginary frequency) or a transition state (one imaginary frequency) and to evaluate its zero-point energy and the thermal corrections at 298 K. Intrinsic reaction coordinates (IRC) were calculated to confirm the connection between the transition states and the reactants/products.[21] On the basis of the optimized structures, single-point energy was refined at the SMD(DCM)/UM06-2X[22]/def2-TZVPP[23] level. All discussed energy differences were based on Gibbs energies at 298 K (standard states are the hypothetical states at 1 mol/L in DCM). The CYL View software was employed to show the 3D structures of the studied species.[24] The vertical excitation energies of **14** are calculated using TDDFT method[25] and long-range hybrid-corrected functional wB97XD as this functional has been shown effective for delocalized and CT-like excited states.[26]

V-A. Computed Energies for the Stationary Points

Table S2. Computed thermal correction to Gibbs free energies (TCG), single point energies (SPE) for the stationary points

	TCG^a (a. u.)	SPE^b (a. u.)
**Endo-INT-IV**	0.327492	-888.628350
**Exo-INT-IV**	0.330417	-888.627156
**Endo-TS1**	0.328820	-888.622542
**Exo-TS1**	0.328842	-888.620810
**INT-V**	0.332869	-888.695795
	TCG^c (a. u.)	SPE^d (a. u.)
**2a-INT-V+ClO$_4$**	0.333741	-1649.691440
**2l-INT-V+ClO$_4$**	0.385839	-2292.997307
**2a-TS2**	0.332366	-1649.661398
**2l-TS2**	0.382724	-2292.961007
**2a-INT-V**	0.334061	-888.694270
**2l-INT-V**	0.381409	-1531.990737
**2a-TS3**	0.327620	-888.652404
**2l-TS3**	0.381900	-1531.966951

	TCG[e] (a. u.)	SPE[f] (a. u.)
**Bn-INT-VI**	0.319528	-888.263324
**Allyl-INT-VI**	0.274914	-734.605800
**Propargyl-INT-VI**	0.251773	-733.364178
***i*-Pr-INT-VI**	0.298467	-735.831611
**CH_2OMe-INT-VI**	0.274281	-771.735873
**Bn-TS4**	0.317314	-888.238182
**Allyl-TS4**	0.273363	-734.581358
**Propargyl-TS4**	0.249845	-733.337818
***i*-Pr-TS4**	0.295523	-735.803215
**CH_2OMe-TS4**	0.272672	-771.705351
**Bn-TS5**	0.315776	-888.227704
**Allyl-TS5**	0.271488	-734.569961
**Propargyl-TS5**	0.248277	-733.327854
***i*-Pr-TS5**	0.295329	-735.799026
**CH_2OMe-TS5**	0.271107	-771.700633

[a]Computed at the SMD(DCM)/UB3LYP/6-311G(d,p) level

[b]Computed at the SMD(DCM)/UM06-2X/def2-TZVPP// SMD(DCM)/UB3LYP/6-311G(d,p) level

[c]Computed at the SMD(DCM)/UB3LYP/6-31+G(d,p) level

[d]Computed at the SMD(DCM)/UM06-2X/def2-TZVPP// SMD(DCM)/UB3LYP/6-31+G(d,p) level

[e]Computed at the UB3LYP/6-31+G(d,p) level

[f]Computed at the SMD(DCM)/UM06-2X/def2-TZVPP// UB3LYP/6-31+G(d,p) level

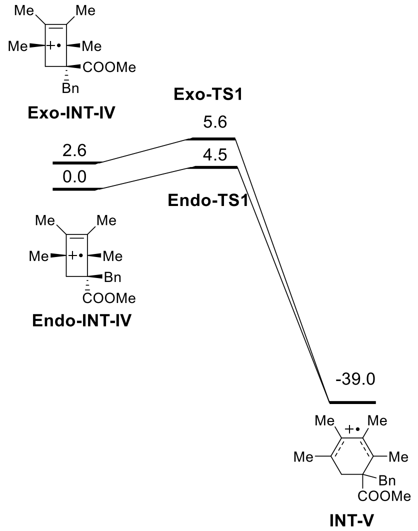

Fig. S12 Gibbs energy profile for the ring-opening processes. Energy in kcal/mol.

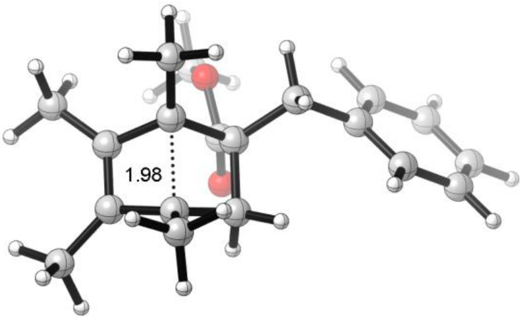

Endo-INT-IV

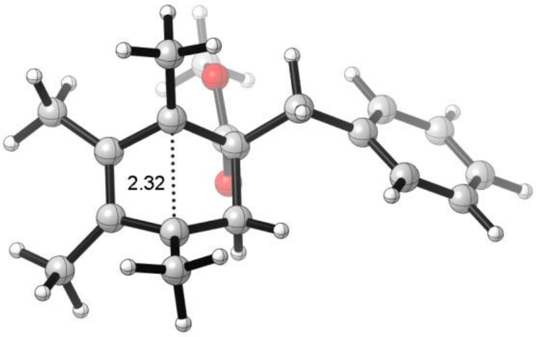

Endo-TS1

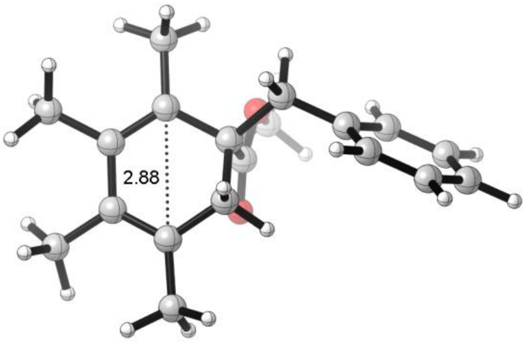

INT-V

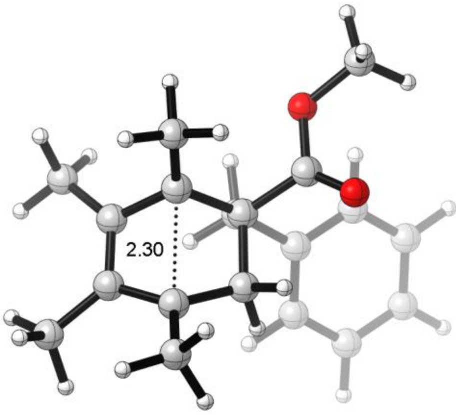

Exo-TS1

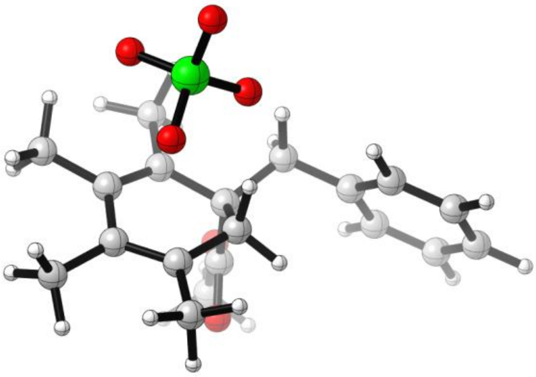

2a-INT-V+ClO_4

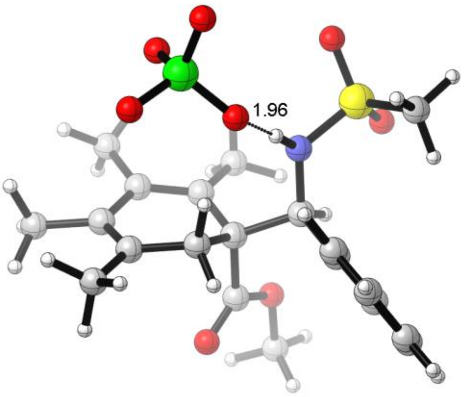

2l-INT-V+ClO_4

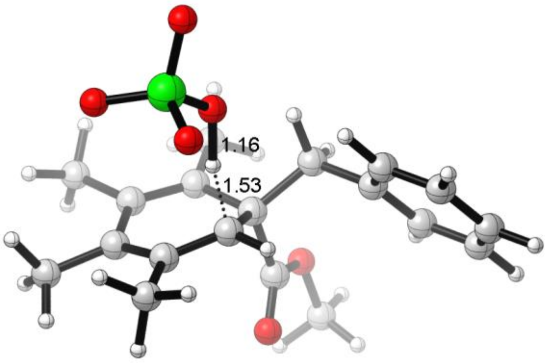

2a-TS2

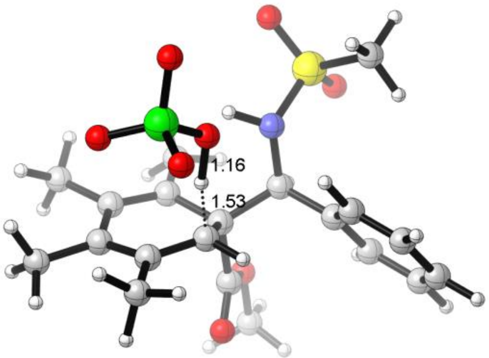

2l-TS2

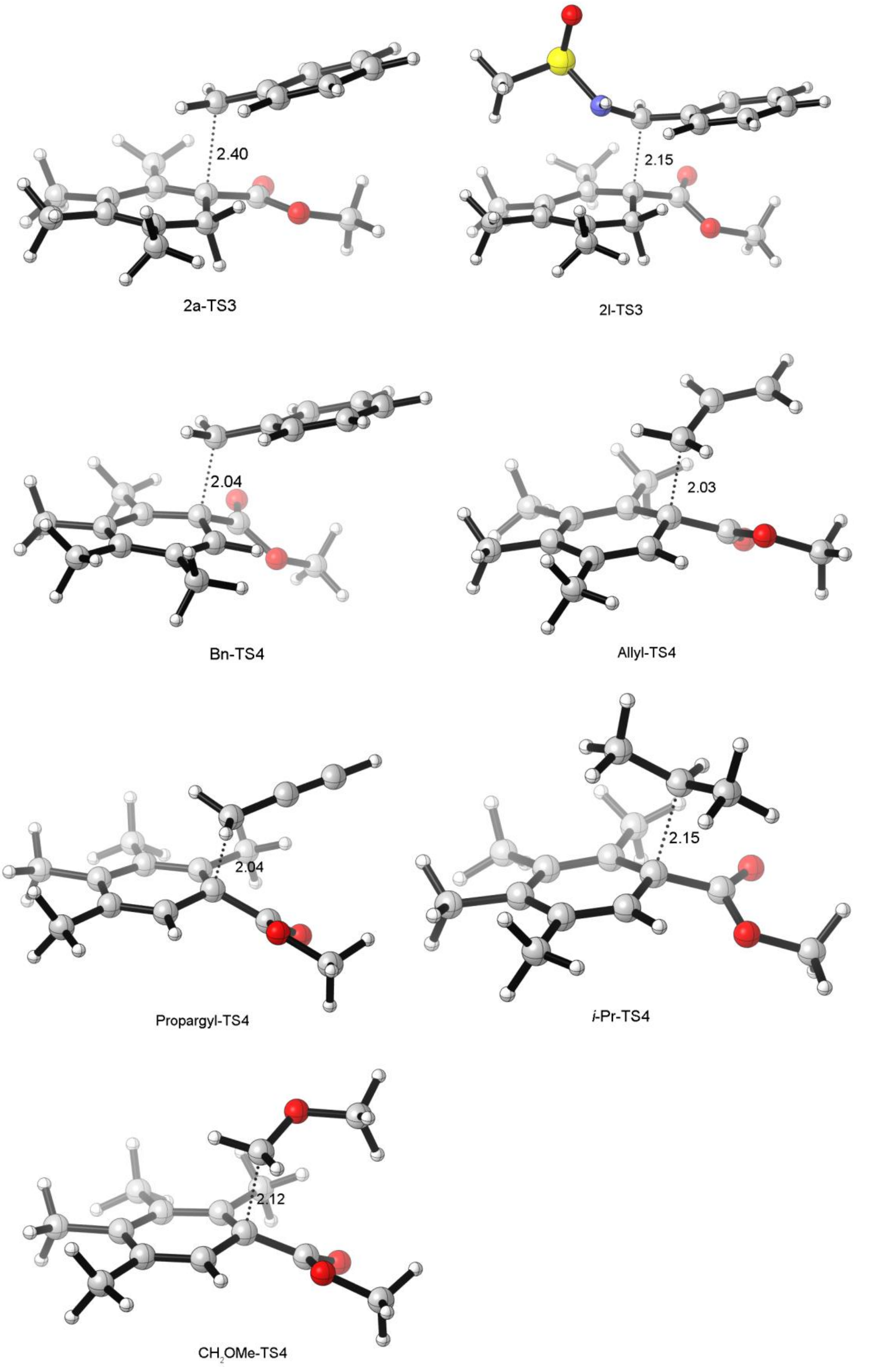

Fig. S13 Key structures of several stationary points. The bond lengths in the structures are given in Å.

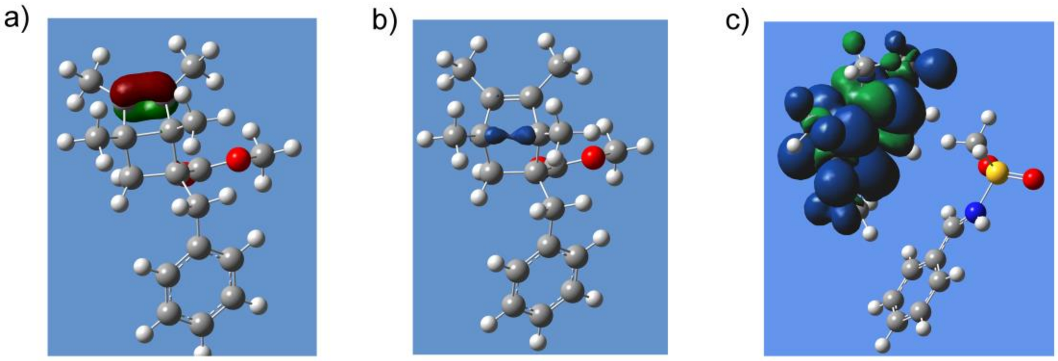

Fig. S14 Frontier molecular orbital and spin distribution analysis of several stationary points. (a) HOMO orbital of *endo*-**2a** (isovalue =0.1) (b) Spin distribution of **Endo-INT-IV** (isovalue =0.027) (c) Spin distribution of the fragmentation products of **2l-INT-V** through **TS3** (isovalue =0.0004)

V-B. Energy Levels, Excitation Energies and Oscillator Strengths for 14

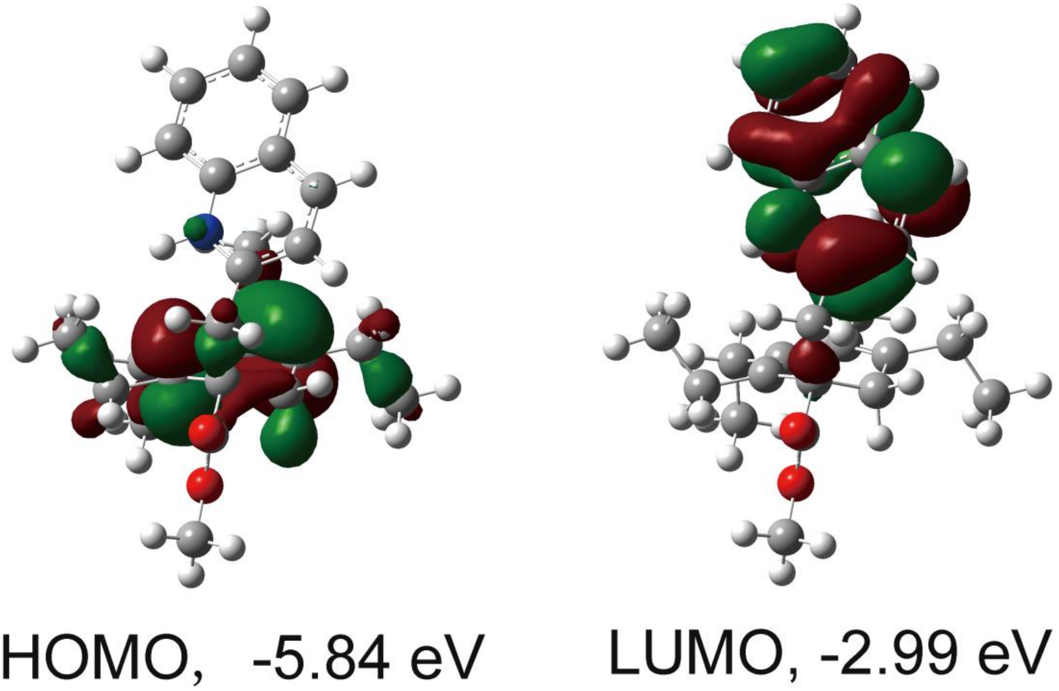

Fig. S15 Frontier molecular orbitals and corresponding energy levels of cation of **14**. Computed at the SMD(DCM)/B3LYP/6-311G(d,p) level (isovalue =0.03)

Table S3. Excitation energies and oscillator strengths for **14**

Electronic Transition	Energy / eV	Wavelength / nm	Oscillator Strength	Configurations[a]
S_0-S_1	3.2784	378.19	0.1195	HOMO→LUMO (0.69140) 96%
S_0-S_2	4.3146	287.36	0.0640	HOMO-1→LUMO (0.68837) 95%
S_0-S_3	4.5292	273.74	0.2206	HOMO-2→LUMO (-0.17498) 6%<br>HOMO→LUMO+2 (0.66256) 88%
S_0-S_4	4.5630	271.72	0.1537	HOMO-3→LUMO (-0.33318) 22%<br>HOMO-2→LUMO (0.49677) 49%<br>HOMO-1→LUMO+1 (-0.26707) 14%<br>HOMO→LUMO+2 (0.21387) 9%

Computed at the SMD(DCM)/wB97XD/6-311G(d,p) level

[a]Only the main configurations are presented

V-C. Cartesian Coordinates for the Stationary Points

Endo-INT-IV

C	-0.44313900	-1.31311400	-0.67433700
C	-1.89011800	-1.59710600	-0.28634500
C	-1.57009800	-0.08414700	0.95549300
C	-2.69715000	0.41313900	0.12320100
C	-2.90267100	-0.61343900	-0.74035400
C	-3.96590500	-0.86282300	-1.74775500
H	-4.59277800	-1.71165900	-1.45473800
H	-4.60079200	0.01384100	-1.87795800
H	-3.51285100	-1.11986200	-2.71176000
C	-3.49548600	1.63968300	0.39797600
H	-2.85017900	2.52370100	0.37228300
H	-4.29756200	1.75981100	-0.33082300
H	-3.93680300	1.59809300	1.39940100
C	-1.72571500	-0.13617200	2.43327800
H	-1.06001100	-0.86303700	2.89993200
H	-2.75786700	-0.35167000	2.71419900
C	-2.29541400	-2.95578400	0.15402400
H	-3.28745600	-2.97226900	0.60745100
H	-2.32013500	-3.60366600	-0.73621000
H	-1.56062800	-3.38837500	0.83662300
C	-0.17195400	-0.11159100	0.25624100
H	0.18980300	-2.16140200	-0.42248400
H	-0.33806500	-1.07853300	-1.73496300
C	0.04225100	1.19763800	-0.52190300
O	0.22019200	1.27286100	-1.71128200
O	-0.00086600	2.25004800	0.30506400
C	0.19957700	3.55483000	-0.29638600
H	0.16061200	4.26144800	0.52906200
H	1.16987300	3.59737000	-0.79093000
H	-0.59120700	3.76407500	-1.01716100
C	1.00169700	-0.35556600	1.24719300
H	0.99947100	0.44699900	1.98730600
H	0.80117100	-1.28974000	1.77473800
H	-1.46691000	0.85049400	2.84485500
C	2.36693600	-0.43746400	0.59257900
C	3.11073100	0.72320100	0.34049300
C	2.92468300	-1.67383800	0.24372300
C	4.36509500	0.65164400	-0.26197600
H	2.71300800	1.68976800	0.63112500
C	4.17956500	-1.74847500	-0.35917600
H	2.37846500	-2.58695600	0.45653700
C	4.90154800	-0.58489500	-0.61864800
H	4.92597400	1.56137300	-0.44604500
H	4.59504400	-2.71588800	-0.61928800
H	5.87861400	-0.64151300	-1.08534100

Exo-INT-IV

C	-0.62446300	-0.57649300	1.25088500
C	-2.14623900	-0.50592100	1.12786700
C	-1.70115300	0.60333600	-0.42919800
C	-2.42251100	-0.53561700	-1.05771300
C	-2.74934300	-1.28935200	0.02481300
C	-3.64682900	-2.46391800	0.18248300
H	-4.51262200	-2.21231600	0.80410100
H	-4.00115000	-2.82671100	-0.78255600
H	-3.11429800	-3.27369200	0.69405800
C	-2.87037500	-0.59869700	-2.47550200
H	-2.00625000	-0.58327500	-3.14807000
H	-3.45328700	-1.50035600	-2.66625400
H	-3.48463600	0.27263100	-2.72692600
C	-2.22227900	1.98374300	-0.63736900
H	-1.96140200	2.65194600	0.18631500
H	-3.30508300	1.98304900	-0.76906600
C	-2.96515000	-0.03839900	2.27517700
H	-3.99742700	0.17783300	1.99659300
H	-2.97807000	-0.84076300	3.02924800
H	-2.51227200	0.83234500	2.75462600
C	-0.24158600	0.31922200	0.05104300
H	-0.29213900	-0.13162800	2.18607400
H	-0.25991200	-1.60123400	1.17796600
C	0.62092000	-0.37062800	-1.05308800
H	-1.77005400	2.40131000	-1.54726700
H	0.06333500	-1.24183900	-1.40019200
C	0.41862100	1.62235500	0.54081600
O	0.58486500	1.92466300	1.69735100
O	0.77783200	2.39438500	-0.48958900
C	1.41300900	3.65501000	-0.16149600
H	2.31181000	3.48138900	0.42996500
H	1.66493500	4.10783400	-1.11739100
H	0.72269600	4.29162300	0.39285300
H	0.72046300	0.31866300	-1.89295500
C	1.99000500	-0.81574700	-0.58047300
C	3.09528900	0.04455300	-0.64065000

C	2.18442000	-2.11286000	-0.08568000
C	4.35008100	-0.37294600	-0.20146800
H	2.97630300	1.04264300	-1.04642500
C	3.43832000	-2.53221900	0.35499900
H	1.35079500	-2.80706200	-0.06123800
C	4.52506700	-1.66102000	0.30315500
H	5.19314100	0.30672800	-0.26007700
H	3.56669800	-3.54172100	0.72993300
H	5.50202400	-1.98618200	0.64298200

Endo-TS1

C	-0.38908000	-1.32281600	-0.51609200
C	-1.80636900	-1.74630300	-0.28541400
C	-1.53435500	0.11333100	1.07502700
C	-2.73788700	0.27355200	0.21398100
C	-2.87261400	-0.78530000	-0.61596400
C	-3.93528000	-1.11098200	-1.61584100
H	-4.49225100	-2.00682100	-1.32236200
H	-4.63918000	-0.28588700	-1.72226800
H	-3.49003500	-1.31348000	-2.59569300
C	-3.67167900	1.42708300	0.38858400
H	-3.12423000	2.36946200	0.27633600
H	-4.48591600	1.40523500	-0.33532300
H	-4.10083100	1.43014800	1.39554100
C	-1.61692700	0.44461200	2.52196000
H	-0.88784000	-0.10461200	3.11911200
H	-2.61679900	0.24577100	2.91266400
C	-2.12227200	-3.14529800	0.02405400
H	-3.13985600	-3.29188800	0.38410700
H	-2.02140600	-3.68831500	-0.93480200
H	-1.38573200	-3.58889000	0.69677100
C	-0.19722100	-0.03109500	0.31061000
H	0.29583000	-2.10370000	-0.19309300
H	-0.23875900	-1.15478400	-1.59012200
C	-0.01477200	1.19880100	-0.60449600
O	0.11717800	1.15715200	-1.80272200
O	-0.03895600	2.33050300	0.11057300
C	0.10767400	3.56856900	-0.62940600
H	0.10325900	4.35415800	0.12243300
H	1.04713500	3.57256500	-1.18202100
H	-0.72746900	3.69506200	-1.31915200
C	1.01733800	-0.16079100	1.28280100
H	1.03795800	0.72958500	1.91317300
H	0.81942100	-1.01553400	1.93293700
H	-1.41417900	1.51606300	2.67597000
C	2.36898500	-0.33915700	0.62144100
C	3.10901100	0.76835000	0.18561900
C	2.92742700	-1.61374900	0.46200700
C	4.35772600	0.60488800	-0.41080700
H	2.71429300	1.76910500	0.32632500
C	4.17621200	-1.78091000	-0.13491600
H	2.38685700	-2.48311200	0.82199800
C	4.89423200	-0.67137100	-0.57713500
H	4.91507900	1.47582400	-0.73782000
H	4.59086600	-2.77699700	-0.24497600
H	5.86711800	-0.79868800	-1.03862700

Exo-TS1

C	-0.68065200	-0.49134600	1.23485700
C	-2.16364700	-0.81249400	1.13100100
C	-1.64870300	0.87104700	-0.34695500
C	-2.40843600	-0.17901100	-1.08040500
C	-2.79657700	-1.09060600	-0.15089700
C	-3.78091600	-2.21181200	-0.32281200
H	-4.75368700	-1.95155500	0.10604000
H	-3.93083100	-2.43763500	-1.37906500
H	-3.43149000	-3.11782000	0.17923500
C	-2.69706900	-0.07933000	-2.53723400
H	-1.76772500	0.06767900	-3.09872600
H	-3.20239800	-0.96883700	-2.91337700
H	-3.32629800	0.79426100	-2.74437600
C	-2.24341700	2.20651600	-0.13782900
H	-1.87396600	2.70021300	0.76378800
H	-3.33370000	2.15541600	-0.11873500
C	-2.84526800	-1.11814900	2.41000500
H	-3.87670400	-1.44456800	2.29146800
H	-2.27616400	-1.88941600	2.95051300
H	-2.81871100	-0.23424800	3.05905200
C	-0.25286400	0.46016100	0.08889100
H	-0.50252300	0.02588400	2.17776700
H	-0.11178500	-1.42936500	1.27689200
C	0.55527500	-0.24089900	-1.05998800
H	-1.96411800	2.84482200	-0.99254700
H	-0.06509400	-1.04467300	-1.45773600
C	0.54714800	1.65526500	0.63919500
O	0.87376000	1.80554200	1.79096600
O	0.83261300	2.52019700	-0.33964900
C	1.57967800	3.70269500	0.04141600

H	2.53428800	3.42021000	0.48499600
H	1.73510600	4.25498900	-0.88211900
H	1.00325700	4.29808900	0.75015600
H	0.70434900	0.48779900	-1.85775300
C	1.88983100	-0.81880700	-0.63483100
C	3.05286800	-0.03762800	-0.63721400
C	1.99245900	-2.16343000	-0.25376800
C	4.27758200	-0.57902200	-0.25082900
H	3.00471600	0.99708900	-0.95802600
C	3.21616200	-2.70755500	0.13284200
H	1.10860500	-2.79312600	-0.27459800
C	4.36254800	-1.91467200	0.13979300
H	5.16712400	0.04117900	-0.26270100
H	3.27463300	-3.75161100	0.42035000
H	5.31610300	-2.33623600	0.43753800

INT-V

C	0.33425500	-1.29011600	-0.09949700
C	1.62636200	-1.69879300	0.50896500
C	1.51863300	0.70061100	-1.05738300
C	2.72988700	0.06786100	-0.75900100
C	2.79163400	-0.99402000	0.20059000
C	4.11052100	-1.37488700	0.80998100
H	4.63941000	-2.10139300	0.18109500
H	4.75926000	-0.50546400	0.92114900
H	3.97547400	-1.82642500	1.79208100
C	4.00831500	0.54440700	-1.39519300
H	4.40708800	1.41711000	-0.86436100
H	4.77357400	-0.22999400	-1.38032500
H	3.84572900	0.83808400	-2.43238300
C	1.48060100	1.90929600	-1.93241200
H	1.26428700	1.60858600	-2.96755200
H	2.42887200	2.44397900	-1.94134500
C	1.61403800	-2.91082200	1.36767500
H	2.55581500	-3.45844100	1.35758100
H	1.41834000	-2.60002500	2.40436900
H	0.79671200	-3.57603100	1.08132200
C	0.22583100	0.20316100	-0.42470600
H	0.23010300	-1.86217100	-1.03384300
H	-0.48909500	-1.60565400	0.54075800
C	0.10860000	0.97398100	0.92725700
O	0.30475400	0.46480800	2.00300400
O	-0.19413700	2.25818500	0.74481100
C	-0.27368100	3.08107400	1.93856400
H	-0.53212700	4.07547700	1.58346800
H	-1.04432700	2.70029400	2.60845300
H	0.69002500	3.09298000	2.44812600
C	-1.01461000	0.47522700	-1.34223100
H	-1.10146900	1.54571700	-1.51577200
H	-0.80344700	0.00459300	-2.30524900
H	0.69422200	2.59820000	-1.62937400
C	-2.33727300	-0.04680500	-0.81515100
C	-3.11376200	0.71507300	0.06832700
C	-2.82865800	-1.29208100	-1.22647700
C	-4.33561700	0.23765000	0.53996100
H	-2.76997400	1.69618300	0.37694500
C	-4.05058600	-1.77164900	-0.75754000
H	-2.25435300	-1.88716700	-1.92896800
C	-4.80650000	-1.00936900	0.13156500
H	-4.92375200	0.84487600	1.21935200
H	-4.41429500	-2.73696800	-1.09259400
H	-5.75894400	-1.37910300	0.49478700

2a-INT-V+ClO_4

C	0.20924100	0.05363300	0.82429900
C	-0.61115500	-0.89506800	1.62050900
C	0.02590600	-1.53043700	-1.10486300
C	-1.00406300	-2.18705700	-0.41476000
C	-1.16827600	-2.02177800	1.00126500
C	-1.99605300	-3.00116400	1.78623500
H	-3.05498500	-2.70877300	1.78688900
H	-1.93222200	-4.00648200	1.36373900
H	-1.66777500	-3.04777700	2.82657200
C	-1.94376100	-3.11128300	-1.14742800
H	-1.49591600	-4.10397400	-1.28928800
H	-2.87776300	-3.24155000	-0.60007900
H	-2.19005700	-2.71580100	-2.13503800
C	0.27215200	-1.78510800	-2.55574000
H	-0.11413200	-0.94160200	-3.14784500
H	-0.21585000	-2.69063600	-2.91314900
C	-0.87508300	-0.53982700	3.04132500
H	-1.84454600	-0.89025200	3.40097700
H	-0.09788900	-1.00105500	3.67102900
H	-0.80102200	0.54159100	3.18603100
C	0.95986500	-0.58455000	-0.35137700
H	-0.49226500	0.80619300	0.42486900
H	0.89740200	0.58789100	1.48144000
C	2.10053300	-1.47600200	0.22628100

O	2.24651500	-1.70453000	1.41137400
O	2.87952500	-1.98153600	-0.73454700
C	3.95834500	-2.85557000	-0.31118600
H	4.47133800	-3.13989300	-1.22911600
H	4.63595400	-2.32332000	0.35900500
H	3.55065700	-3.73644100	0.18970300
C	1.55187200	0.52647600	-1.28926700
H	2.11014400	0.05218600	-2.09711300
H	0.69927500	1.04389400	-1.73865100
H	1.34064700	-1.85791000	-2.77014200
C	2.45023300	1.54276500	-0.60645600
C	3.82798800	1.30672100	-0.46475300
C	1.93115900	2.75724000	-0.12872700
C	4.65879500	2.24647500	0.15301500
H	4.25897200	0.38864900	-0.85489300
C	2.75883000	3.70016800	0.48796500
H	0.87228200	2.97112000	-0.24728700
C	4.12598200	3.44574100	0.63566500
H	5.72229400	2.04439400	0.24816800
H	2.33549100	4.63412600	0.84748700
H	4.77091500	4.17876600	1.11226100
Cl	-3.82472400	1.53288200	-0.32057600
O	-2.39699700	1.99418900	-0.45611200
O	-4.74913500	2.64142700	-0.73218700
O	-4.09121600	1.15756900	1.11025100
O	-4.04565400	0.33655700	-1.20350200

2l-INT-V+ClO_4

C	-0.08000800	1.13955000	-1.13779400
C	-0.86254700	2.39236400	-1.29317100
C	0.13092400	1.69399800	1.31687200
C	-0.85381300	2.67836600	1.13356700
C	-1.21007200	3.15278000	-0.17087000
C	-2.00065700	4.42341800	-0.31719600
H	-3.07709400	4.22961600	-0.21314100
H	-1.72635200	5.15253000	0.44863300
H	-1.84246300	4.88035300	-1.29530300
C	-1.50961000	3.30593200	2.33514800
H	-0.91739000	4.15482000	2.70413600
H	-2.50551700	3.67983000	2.09275500
H	-1.60487100	2.59349000	3.15574300
C	0.58890000	1.37523200	2.70457000
H	-0.21246900	0.86875300	3.25768500
H	0.80991700	2.29855700	3.25287700
C	-1.32088800	2.73183100	-2.66910000
H	-2.31628200	3.18193100	-2.68521500
H	-0.62305500	3.46094800	-3.10989200
H	-1.31076200	1.84763800	-3.31146600
C	0.81857500	1.06845300	0.10492000
H	-0.82558900	0.32837600	-1.10774300
H	0.50596100	0.96628900	-2.04224900
C	2.07307900	1.96250000	-0.16201700
O	2.18019400	2.70021300	-1.12096100
O	2.98204500	1.84931000	0.80784900
C	4.17582800	2.66795900	0.68860800
H	4.77377300	2.42776900	1.56672000
H	4.71553700	2.41436300	-0.22593500
H	3.90428700	3.72567500	0.68533000
C	1.28783700	-0.41922200	0.38409800
H	2.02670300	-0.37317200	1.18150800
H	1.46979700	0.74062200	2.73496800
C	1.97427500	-1.07456700	-0.81764000
C	3.37619800	-1.05299400	-0.89225100
C	1.25821900	-1.72062700	-1.83772400
C	4.04663300	-1.64288900	-1.96838700
H	3.95064600	-0.58578500	-0.09675700
C	1.92665900	-2.31333100	-2.91182200
H	0.17471200	-1.77719100	-1.79786400
C	3.32283400	-2.27221500	-2.98445800
H	5.13224400	-1.61906700	-2.00421800
H	1.35381500	-2.81043900	-3.68969500
H	3.84029200	-2.73592900	-3.81945700
Cl	-3.64350700	-1.21612500	-0.69171200
O	-2.18168000	-1.58948800	-0.87033600
O	-4.45628600	-2.46873400	-0.60144800
O	-4.07612000	-0.40436700	-1.87562200
O	-3.79053100	-0.41075000	0.56314700
N	0.14574100	-1.15857200	0.94150800
S	0.36843000	-2.43161800	2.00235900
C	0.76188300	-3.91402000	1.06156400
H	-0.06022500	-4.11983600	0.37361500
H	1.69860200	-3.75887400	0.52503300
H	0.86470900	-4.72556900	1.78643300
H	-0.69025300	-1.28773600	0.36261400
O	-0.93852500	-2.65397500	2.64264800
O	1.54220100	-2.09258800	2.82974400

2a-TS2

C	-0.09770400	-0.15293300	0.75466300
C	-1.02256800	-0.96031700	1.42351900
C	0.08614500	-1.86656900	-1.06934600
C	-0.97713500	-2.53161200	-0.47939200
C	-1.43675500	-2.19112500	0.84044200
C	-2.41205300	-3.08081200	1.56353700
H	-3.45133800	-2.85342500	1.28367300
H	-2.23920100	-4.13553300	1.33427200
H	-2.33806800	-2.95737200	2.64614200
C	-1.69777200	-3.63902800	-1.22524200
H	-1.19858200	-4.60823500	-1.09451700
H	-2.72547800	-3.75074400	-0.87491200
H	-1.74278800	-3.43454200	-2.29685500
C	0.59431300	-2.25046400	-2.43456100
H	0.12527500	-1.63269500	-3.21450800
H	0.37812200	-3.29338800	-2.67242800
C	-1.63133900	-0.47165500	2.71091200
H	-2.72436500	-0.53092000	2.70207000
H	-1.28046000	-1.09069700	3.54658500
H	-1.33497000	0.55955300	2.91524700
C	0.81281700	-0.75302400	-0.29847700
H	-1.15991300	0.62073100	-0.02215900
H	0.31569200	0.68538800	1.31209400
C	2.00035300	-1.45256100	0.44748700
O	2.07774700	-1.59609700	1.65208300
O	2.92705600	-1.91299300	-0.40885700
C	4.05410100	-2.61580200	0.16822800
H	4.69204300	-2.87685900	-0.67590900
H	4.58947700	-1.96993100	0.86737100
H	3.71342700	-3.51786400	0.68210900
C	1.38753100	0.35245700	-1.26222300
H	2.05243500	-0.12437600	-1.98231100
H	0.54286200	0.75865400	-1.82595300
H	1.67294300	-2.11102200	-2.52000500
C	2.13669300	1.48949300	-0.58920600
C	3.51031000	1.38373600	-0.31008400
C	1.48543300	2.68956800	-0.25876500
C	4.20503600	2.43343000	0.29872100
H	4.04570000	0.47905300	-0.58456100
C	2.17689900	3.74290500	0.34717700
H	0.42947500	2.80424600	-0.48606500
C	3.53966000	3.61685100	0.63358300
H	5.26732500	2.32894900	0.50303200
H	1.65045500	4.66211800	0.58998000
H	4.07883700	4.43464900	1.10366200
Cl	-3.19421100	1.79815200	-0.36032400
O	-1.73240300	1.24837900	-0.81398000
O	-3.59875600	2.69483700	-1.46012800
O	-3.01009100	2.50941500	0.92308500
O	-4.08119300	0.62276600	-0.22675100

2l-TS2

C	-0.67631600	0.73757800	-0.96234500
C	-1.89543200	1.42041200	-1.06455800
C	-0.38631600	1.62173800	1.37721000
C	-1.68815000	2.09589200	1.29838900
C	-2.40651800	2.13387300	0.05565900
C	-3.71708000	2.86577200	-0.04044300
H	-4.54676600	2.25027900	0.33695300
H	-3.70670500	3.78542400	0.55120400
H	-3.95604900	3.13323900	-1.07112400
C	-2.37357500	2.62099700	2.54230200
H	-2.19942500	3.69858900	2.66486200
H	-3.45364700	2.46658000	2.49869900
H	-2.00683100	2.12838100	3.44420300
C	0.36307300	1.68210800	2.68508300
H	-0.06040900	0.97801900	3.41338600
H	0.28879900	2.67946500	3.13246800
C	-2.68179900	1.32113600	-2.34329000
H	-3.72963300	1.06110400	-2.17057500
H	-2.66790000	2.29267400	-2.85419700
H	-2.23884300	0.58741500	-3.02050200
C	0.32667100	1.16436000	0.09495900
H	-1.35826300	-0.56752400	-0.60813700
H	-0.24160700	0.39460600	-1.89856500
C	1.04595600	2.45874000	-0.44598500
O	0.76312500	3.02289500	-1.48282200
O	1.98177500	2.89589100	0.40757600
C	2.67695100	4.11519000	0.04388700
H	3.37902700	4.29740200	0.85686200
H	3.20879400	3.98180900	-0.90055400
H	1.96672200	4.94063800	-0.04104300
C	1.46031900	0.08627100	0.35139500
H	2.18345200	0.54621500	1.02547000
H	1.42181700	1.45308000	2.58462500
C	2.22771200	-0.30720200	-0.91186800
C	3.43774300	0.34016400	-1.20557900
C	1.77267000	-1.30787800	-1.78477700

C	4.16381200	0.01634400	-2.35574600
H	3.82381700	1.09237000	-0.52320400
C	2.49817300	-1.63511900	-2.93331700
H	0.85839000	-1.84610400	-1.55725500
C	3.69350000	-0.97038600	-3.22642500
H	5.09980600	0.52783900	-2.56271500
H	2.13024100	-2.41318200	-3.59663700
H	4.25790500	-1.22716600	-4.11854300
Cl	-3.19388000	-2.10350600	-0.60335100
O	-1.66431000	-1.66061500	-0.29281400
O	-3.35752000	-3.37534700	0.12729000
O	-3.31570900	-2.27156300	-2.06803800
O	-4.07159300	-1.02909000	-0.08884000
N	0.86217000	-1.09274600	1.00920900
S	1.78508500	-2.04255800	2.05314700
C	2.25220700	-3.47146000	1.07759400
H	1.34382800	-3.97238500	0.73958700
H	2.85932600	-3.14136700	0.23382600
H	2.83312600	-4.12222700	1.73563900
H	-0.05135800	-0.95431600	1.43468300
O	0.85610900	-2.49235000	3.10868400
O	3.02064700	-1.32635500	2.42644700

2a-INT-V

C	0.32122100	-1.27972000	-0.06130900
C	1.61840200	-1.70546100	0.52926900
C	1.52184800	0.68675300	-1.06020600
C	2.73364400	0.04931500	-0.75356600
C	2.79170100	-1.00917700	0.21349100
C	4.11127100	-1.40396000	0.81650500
H	4.62999700	-2.13880100	0.18567100
H	4.77133700	-0.54008600	0.92283000
H	3.97667700	-1.85296500	1.80203800
C	4.01460100	0.51197400	-1.39902100
H	4.41940000	1.39132200	-0.87998000
H	4.77800500	-0.26663000	-1.37595700
H	3.85170000	0.79299800	-2.44165100
C	1.49282100	1.87519300	-1.96698000
H	1.30356100	1.54595000	-3.00058000
H	2.43940600	2.41717300	-1.96845300
C	1.60672600	-2.92651900	1.37971300
H	2.53701500	-3.49559400	1.32868200
H	1.45784700	-2.62547300	2.42877200
H	0.76433600	-3.57210400	1.11528600
C	0.22527300	0.21159100	-0.40990500
H	0.18758200	-1.86525700	-0.98557000
H	-0.49540400	-1.57476100	0.60043500
C	0.10628600	1.01173900	0.92457200
O	0.26581400	0.51483800	2.02240900
O	-0.15471500	2.30373700	0.71581000
C	-0.23966100	3.15320100	1.89137400
H	-0.45293600	4.14952500	1.50667100
H	-1.04433000	2.81290800	2.54583400
H	0.71100900	3.14265900	2.42847500
C	-1.01587200	0.47246000	-1.33518700
H	-1.10725300	1.54335100	-1.51607200
H	-0.79606300	-0.00416200	-2.29534000
H	0.69555300	2.56950500	-1.70134700
C	-2.33987200	-0.05320100	-0.81118400
C	-3.12029200	0.70740800	0.07565000
C	-2.83168500	-1.30043700	-1.22838900
C	-4.34622900	0.22720900	0.54590200
H	-2.77714500	1.68971700	0.38898300
C	-4.05810600	-1.78333500	-0.76219700
H	-2.25439300	-1.89475000	-1.93230100
C	-4.81789300	-1.02223700	0.13135800
H	-4.93590800	0.83359000	1.22797200
H	-4.42112300	-2.74959100	-1.10186000
H	-5.77275700	-1.39389200	0.49237400

2l-INT-V

C	1.10097600	-0.49484300	-1.32551800
C	2.55386000	-0.51969800	-1.63520900
C	1.64241000	0.96692700	0.65357800
C	2.93886700	1.11916400	0.13315000
C	3.45263300	0.23882800	-0.87490400
C	4.92828700	0.18978000	-1.15517400
H	5.21894300	0.97304600	-1.86875600
H	5.51048500	0.34561700	-0.24428300
H	5.21672100	-0.77074600	-1.58548100
C	3.83953300	2.19174700	0.68562700
H	4.33689900	1.84576500	1.60215600
H	4.61713100	2.46871800	-0.02709800
H	3.27659100	3.09122900	0.94168100
C	1.21095200	1.82723500	1.80065600
H	1.04988900	2.86030100	1.46022700
H	1.99605600	1.87583900	2.56298200
C	2.97593500	-1.31427400	-2.82040000

H	3.80163500	-0.85327200	-3.36763300
H	3.32204200	-2.30335300	-2.48088800
H	2.13407000	-1.48436600	-3.49622500
C	0.74335000	-0.15166600	0.12723200
H	0.65979100	-1.45116200	-1.61045000
C	1.07232500	-1.39791100	1.01620400
O	1.58172000	-2.41179500	0.58628100
O	0.75857800	-1.18594600	2.29319900
C	1.03345700	-2.26511600	3.22843000
H	0.69225200	-1.89521800	4.19414600
H	0.48218000	-3.16201600	2.94036900
H	2.10552000	-2.47139400	3.25054100
C	-0.80134500	0.16564100	0.27041700
H	-0.99042300	0.37341000	1.32423800
H	0.29753300	1.48516900	2.28002100
C	-1.69697200	-1.01159500	-0.11568200
C	-2.18628500	-1.85073500	0.89717800
C	-2.04977800	-1.28557900	-1.44654100
C	-2.98427200	-2.95600400	0.58739600
H	-1.95195900	-1.63365600	1.93581100
C	-2.84972400	-2.38814900	-1.75715600
H	-1.71411800	-0.62845000	-2.24228300
C	-3.31388800	-3.23140200	-0.74227400
H	-3.35481700	-3.59262400	1.38579300
H	-3.11354300	-2.58527300	-2.79248900
H	-3.93603200	-4.08800500	-0.98553200
N	-1.10071200	1.36999700	-0.54032600
S	-2.38994800	2.38049100	-0.07484300
C	-3.76790700	1.86211800	-1.09479800
H	-3.48417900	1.96492500	-2.14283900
H	-4.02626000	0.83141300	-0.85057900
H	-4.59615200	2.53175100	-0.84962200
H	-0.30466200	1.99150800	-0.68075100
O	-1.97099000	3.73042300	-0.49916300
O	-2.74536400	2.14006900	1.33603800
H	0.65251400	0.25418600	-1.99740900

2a-TS3

C	-0.79924600	0.49373800	1.21153700
C	-1.93724000	1.39022100	0.89650600
C	-1.74625100	-1.18699900	-0.38796000
C	-2.90956600	-0.37009600	-0.46715200
C	-2.95936800	0.95862000	0.07331900
C	-4.14280400	1.84847700	-0.22778600
H	-4.45556500	1.75871700	-1.27183500
H	-5.00769700	1.58739200	0.39548800
H	-3.90836500	2.89727800	-0.04137400
C	-4.13135300	-0.90407200	-1.16222000
H	-5.04247400	-0.44790900	-0.77296500
H	-4.09390900	-0.68708300	-2.23975700
H	-4.21766500	-1.98654300	-1.05318100
C	-1.71784100	-2.47743800	-1.16981800
H	-2.30428600	-2.39131600	-2.08689300
H	-2.14414900	-3.30011000	-0.58082500
C	-1.94817700	2.71935100	1.59183200
H	-1.76820200	3.53365400	0.87656200
H	-2.91411000	2.92073600	2.06624600
H	-1.16825700	2.76962600	2.35563800
C	-0.64118900	-0.73282700	0.36809300
H	0.13133200	1.07078800	1.26279000
H	-0.94183200	0.16941700	2.26110500
C	0.45848100	-1.66731600	0.79025300
O	0.60280600	-2.82392800	0.42768500
O	1.28376500	-1.07489600	1.67110500
C	2.35092900	-1.89170300	2.20720600
H	2.91151600	-1.23144100	2.86814200
H	1.93491400	-2.73141200	2.76884100
H	2.99029900	-2.26309500	1.40448400
C	0.56164400	0.15534100	-1.50754300
H	-0.22873600	0.89305500	-1.59342600
H	-0.70260000	-2.76893000	-1.42677000
C	1.87134300	0.57990700	-1.15397300
C	2.98115200	-0.30188600	-1.28249200
C	2.10763000	1.90121300	-0.68273200
C	4.26255600	0.12482600	-0.96134800
H	2.81424100	-1.31214900	-1.64532400
C	3.39382600	2.31879600	-0.36278500
H	1.27150600	2.58910800	-0.59331800
C	4.47336200	1.43331100	-0.49833800
H	5.10354700	-0.55339400	-1.07062800
H	3.56426300	3.33276400	-0.01344800
H	5.47781800	1.76299100	-0.24932000
H	0.43851300	-0.77033500	-2.05816200

2l-TS3

C	0.20234000	-1.61766000	1.15497100
C	-1.08306400	-1.45186100	1.87995400
C	-1.06074100	-1.44895600	-1.00484500

C	-2.25076300	-1.57783800	-0.25359400
C	-2.27477000	-1.42984700	1.17772600
C	-3.59597800	-1.30447900	1.89705100
H	-4.31327700	-0.71302000	1.32253300
H	-4.05028800	-2.28927100	2.06865800
H	-3.47536500	-0.82688700	2.87090900
C	-3.54763300	-1.87039100	-0.96436100
H	-4.27350700	-2.33494500	-0.29637600
H	-4.00407600	-0.95239100	-1.36093800
H	-3.39334000	-2.54278600	-1.81072600
C	-1.14444100	-1.45654900	-2.50997200
H	-2.11490200	-1.10449500	-2.85915800
H	-1.00489300	-2.47750700	-2.89260100
C	-1.00684300	-1.41720200	3.37696400
H	-1.15133600	-0.39207500	3.74755300
H	-1.77785800	-2.03601900	3.84514500
H	-0.02722800	-1.75515000	3.72519100
C	0.19652900	-1.28363500	-0.31817500
H	1.00802500	-1.09930100	1.68451900
H	0.47446300	-2.68391100	1.26249200
C	1.45307300	-1.68989600	-1.05570600
O	1.69527600	-1.46440600	-2.22954300
O	2.31188200	-2.33496900	-0.24844800
C	3.55125400	-2.78617000	-0.84772600
H	4.09250200	-3.28199000	-0.04259100
H	3.34258600	-3.48763700	-1.65847200
H	4.12207000	-1.93659800	-1.22723200
C	0.48528100	0.84205800	-0.38900500
H	-0.36638000	-0.84381200	-2.95864100
C	1.83089600	1.06905800	0.17120000
C	2.92305900	1.12964200	-0.71823300
C	2.06117400	1.27280500	1.54685500
C	4.20647400	1.39774500	-0.24378100
H	2.75533100	0.98684200	-1.78046500
C	3.34719400	1.53349600	2.01630700
H	1.24013600	1.23607800	2.25777100
C	4.42362000	1.59553000	1.12363200
H	5.03475800	1.45888800	-0.94336200
H	3.50971700	1.69253400	3.07807200
H	5.42379800	1.80400300	1.49184900
N	-0.60561300	1.42298500	0.24632900
H	-0.44950100	1.78297800	1.18686700
S	-1.68478000	2.45021700	-0.63131000
O	-1.34609900	3.84292800	-0.30415700
H	0.42186000	0.87388000	-1.47187200
C	-3.26793600	2.06590300	0.10681600
H	-3.52627200	1.03258300	-0.12096900
H	-3.20303700	2.24093500	1.18272800
H	-3.98613300	2.75581400	-0.34400400
O	-1.63661300	1.99374500	-2.02493900

Bn-INT-VI

C	0.59769500	0.22270900	-1.40135800
C	0.28460700	1.55113400	-1.33880100
C	0.76110500	0.12433600	1.10960400
C	0.41252600	1.45703000	1.13679800
C	0.15844100	2.20068300	-0.06705300
C	-0.22917400	3.65525900	0.02831500
H	-1.20175600	3.78390300	0.52438000
H	0.49493200	4.23357200	0.61635300
H	-0.30332500	4.12864500	-0.95097300
C	0.30638200	2.19877200	2.45556100
H	0.41509300	1.53928700	3.31613400
H	1.07534100	2.97750500	2.53837600
H	-0.66278700	2.70128700	2.54910200
C	1.10891100	-0.64535700	2.36648600
H	0.25456800	-0.74202700	3.04897400
H	1.91587100	-0.15938400	2.92636400
C	0.06320600	2.31167000	-2.62999900
H	-0.95365500	2.71645000	-2.69309900
H	0.75565400	3.15577900	-2.72936700
H	0.21406700	1.65646100	-3.49204600
C	0.80555500	-0.65266200	-0.19259500
H	0.68408900	-0.27027400	-2.36691800
C	2.19846600	-1.30328100	-0.41355200
O	2.39223500	-2.34124200	-1.01522100
O	3.19820600	-0.54378900	0.07951600
C	4.53357900	-1.02145000	-0.17338100
H	5.19458800	-0.28952600	0.29020700
H	4.67758700	-2.00925200	0.27115500
H	4.71802900	-1.08190600	-1.24875800
C	-0.24530100	-1.83956600	-0.20619500
H	-0.07344500	-2.40018400	-1.12857700
H	-0.00311900	-2.51946400	0.61686400
H	1.44827600	-1.65917100	2.13741500
C	-1.69487600	-1.42039900	-0.11052600
C	-2.43658300	-1.11460300	-1.26260300
C	-2.34880500	-1.35326600	1.12923800

C	-3.77937200	-0.73874300	-1.17865700
H	-1.95920200	-1.17945400	-2.23640200
C	-3.69163700	-0.97830800	1.22058000
H	-1.80423800	-1.60937000	2.03418800
C	-4.41211500	-0.66511500	0.06528100
H	-4.33283800	-0.51054800	-2.08550900
H	-4.17551900	-0.93808500	2.19269600
H	-5.45739700	-0.37705000	0.13222800

Allyl-INT-VI

C	0.13363100	-0.35395000	-1.42435400
C	1.39486900	-0.84109700	-1.22358900
C	0.01066600	0.36644700	0.98802700
C	1.30022500	-0.09386100	1.14118600
C	2.01428800	-0.72492900	0.06438300
C	3.41224200	-1.24143500	0.30078400
H	4.13377300	-0.42210500	0.43707300
H	3.47111700	-1.85708300	1.20626200
H	3.77289300	-1.84996000	-0.52922100
C	2.01063300	0.03942200	2.47547800
H	1.48887200	0.70879500	3.15922100
H	2.11020300	-0.93284600	2.97606100
H	3.02365500	0.43405900	2.34635400
C	-0.77425400	0.98242500	2.12431700
H	-1.84113400	1.03611400	1.88915700
H	-0.44407100	2.00779400	2.34119400
C	2.12317000	-1.48657900	-2.38497800
H	3.06724200	-0.97655400	-2.60925100
H	2.36365500	-2.53661000	-2.17994800
H	1.50677500	-1.45859700	-3.28740700
C	-0.69544600	0.31286400	-0.35457100
H	-0.32884300	-0.42842300	-2.40609000
C	-1.99702400	-0.53075400	-0.28019500
O	-2.98882900	-0.33262800	-0.95389600
O	-1.88368400	-1.56778600	0.57430000
C	-3.01016000	-2.46428400	0.62707600
H	-2.74099400	-3.22315300	1.36135300
H	-3.91141700	-1.92925100	0.93607700
H	-3.18071700	-2.91789900	-0.35241000
C	-1.11589900	1.75122100	-0.83824400
H	-1.61582600	1.62424900	-1.80577500
H	-1.86534900	2.16787000	-0.15733300
H	-0.68065800	0.40379700	3.04823300
C	0.04043100	2.70071800	-0.97501600
H	0.88844600	2.34409100	-1.55935100
C	0.08089400	3.93229100	-0.45692200
H	0.93411700	4.58661600	-0.61157600
H	-0.74124200	4.33141100	0.13384500

Propargyl-INT-VI

C	0.10637700	0.02474400	-1.42369400
C	1.30104500	-0.62962500	-1.32285500
C	-0.00902900	0.22995700	1.08785600
C	1.21699600	-0.39453000	1.14659900
C	1.88640600	-0.86522700	-0.03540300
C	3.21065600	-1.57660200	0.09215700
H	4.02189900	-0.88523400	0.36586200
H	3.18499100	-2.34696400	0.87135700
H	3.50694400	-2.06400400	-0.83750100
C	1.90326800	-0.61542800	2.48181100
H	1.44921000	-0.03259400	3.28305500
H	1.87168600	-1.67152300	2.78110300
H	2.95916000	-0.33089300	2.43359800
C	-0.75558200	0.68623100	2.32178700
H	-1.81278400	0.86788700	2.10495500
H	-0.34911700	1.62374300	2.72669800
C	1.99937500	-1.07624300	-2.59055500
H	3.00061400	-0.63899500	-2.67755800
H	2.11636600	-2.16592600	-2.62793200
H	1.42626400	-0.77413700	-3.47098400
C	-0.68004000	0.52573000	-0.24002300
H	-0.32626800	0.22419100	-2.40127100
C	-2.06395400	-0.17181600	-0.34621900
O	-3.02061600	0.27951000	-0.94525300
O	-2.06873700	-1.38014500	0.24795500
C	-3.28181900	-2.14547100	0.10793600
H	-3.10229800	-3.07205400	0.65241400
H	-4.12822900	-1.60264100	0.53540100
H	-3.48179300	-2.34954800	-0.94679500
C	-0.95864600	2.06819900	-0.41451000
H	-1.47739000	2.20927600	-1.36784400
H	-1.65609300	2.40281900	0.36168700
H	-0.72080700	-0.05868200	3.12185700
C	0.24754100	2.89081500	-0.37205200
C	1.23225800	3.59319900	-0.33093600
H	2.10336300	4.20674900	-0.30112000

***i*-Pr-INT-VI**

C	0.09893200	0.54949800	1.20114300
C	1.43118100	0.83472700	1.12429600
C	0.23942800	-0.79141300	-0.92841900
C	1.58827100	-0.49318000	-0.96035300
C	2.21321300	0.33520500	0.03085000
C	3.68401700	0.65578600	-0.07828600
H	4.31488200	-0.21370800	0.16112600
H	3.95858300	0.96700500	-1.09277400
H	3.97967700	1.45892300	0.59800000
C	2.46743400	-1.05224700	-2.06582300
H	1.99831200	-1.88440000	-2.59041400
H	2.70779900	-0.28504200	-2.81427300
H	3.42002100	-1.41566000	-1.66860200
C	-0.41093900	-1.65052100	-1.99381300
H	-0.15797300	-2.71379700	-1.87245400
H	-0.09130900	-1.36036400	-2.99996800
C	2.06189400	1.68931600	2.20469200
H	2.89927900	1.17895300	2.69477000
H	2.44942900	2.63305300	1.80207000
H	1.32647300	1.93706900	2.97477400
C	-0.66015000	-0.28336800	0.20620700
H	-0.47594400	0.95835200	2.02667200
C	-1.76106400	0.57875400	-0.47784200
O	-2.79323700	0.14576600	-0.95405200
O	-1.42439700	1.88331000	-0.54090700
C	-2.35430200	2.74119500	-1.22928500
H	-1.92588200	3.74114300	-1.16434100
H	-2.45746400	2.43249900	-2.27254900
H	-3.33459300	2.70824300	-0.74776200
C	-1.38593900	-1.49774500	0.92606900
H	-1.97666400	-1.99703400	0.15207700
H	-1.49856600	-1.57211500	-1.96791000
C	-2.36662200	-1.05435800	2.02531100
C	-0.37316300	-2.50254300	1.49366800
H	-2.93580300	-1.92118900	2.37877700
H	-1.84479900	-0.63572600	2.89277300
H	-3.09011500	-0.31776700	1.66371200
H	-0.90001200	-3.35745900	1.93192800
H	0.30459100	-2.88254700	0.72358100
H	0.23800100	-2.04567400	2.27995400

CH_2OMe-INT-VI

C	0.15900000	0.05197400	-1.40765200
C	1.48386300	-0.27417500	-1.35953200
C	0.04706700	0.02026700	1.11292900
C	1.39372500	-0.26669000	1.12072400
C	2.13665800	-0.45250500	-0.09538100
C	3.60333500	-0.80010000	-0.02729100
H	4.21207000	0.05709900	0.29818400
H	3.79826300	-1.60728400	0.68820400
H	3.99511700	-1.12076400	-0.99340400
C	2.14159200	-0.40332100	2.43419700
H	3.09621600	0.13112800	2.40196400
H	1.57710400	-0.00146500	3.27559700
H	2.37171000	-1.45307800	2.66068700
C	-0.76413500	0.17389000	2.37952300
H	-0.61255000	1.15774400	2.84572900
H	-0.51241300	-0.58572400	3.12505000
C	2.24900400	-0.41813300	-2.65907500
H	3.09871800	0.27255800	-2.70884400
H	2.64726100	-1.43177300	-2.78768900
H	1.59873800	-0.20781400	-3.51236900
C	-0.70866400	0.23161100	-0.18718900
H	-0.32965300	0.21143700	-2.36613300
C	-1.86367800	-0.79267500	-0.34427000
O	-2.92958900	-0.55413900	-0.87927000
O	-1.53133300	-2.01036600	0.12524600
C	-2.50876400	-3.04981500	-0.07305700
H	-2.07244700	-3.94426900	0.37075000
H	-3.44860200	-2.79160600	0.42090500
H	-2.69330200	-3.19888100	-1.13983800
C	-1.37383500	1.63998700	-0.23255100
H	-1.88242300	1.76437800	-1.19823400
H	-2.13565700	1.73108700	0.55678300
H	-1.83650500	0.08056900	2.18166300
O	-0.36843800	2.62101000	-0.06150800
C	-0.87179000	3.93946300	-0.16525600
H	-0.02745400	4.61743300	-0.02053200
H	-1.31672200	4.12465600	-1.15520400
H	-1.63286200	4.14386900	0.60425300

Bn-TS4

C	-0.65714800	0.56217200	0.96895100
C	-1.76677300	1.37697800	0.91899700
C	-1.74891800	-1.11468200	-0.48021100
C	-2.87921700	-0.28935400	-0.49857800
C	-2.88946500	0.98165300	0.13842400
C	-4.10129800	1.88063400	0.03564100

H	-4.49665200	1.91341500	-0.98456300
H	-4.92196000	1.54065100	0.68404100
H	-3.86905600	2.90620200	0.32677200
C	-4.12990800	-0.76850900	-1.21362800
H	-4.26473600	-1.84730400	-1.10902300
H	-5.03070900	-0.29202600	-0.82392400
H	-4.08762100	-0.55484100	-2.29083600
C	-1.78459000	-2.43933100	-1.21352300
H	-2.16455300	-2.31432000	-2.23463200
H	-2.45641800	-3.15093300	-0.71435700
C	-1.77664900	2.65874300	1.72620800
H	-1.82449000	3.54776700	1.08511800
H	-2.63419400	2.70722700	2.40715000
H	-0.86786100	2.73685600	2.32881800
C	-0.52347300	-0.62951800	0.15870000
H	0.18877500	0.85493800	1.57840300
C	0.48502500	-1.64935000	0.65430200
O	0.87090000	-2.63720300	0.05405300
O	0.95863500	-1.33639300	1.88103100
C	1.96430900	-2.22048200	2.41067000
H	2.21604000	-1.81356300	3.38981900
H	1.56929700	-3.23486500	2.50304900
H	2.84183100	-2.23196200	1.76039900
C	0.54825400	0.00361900	-1.45892700
H	0.54223400	-0.92694700	-2.01975300
H	-0.18757100	0.72934300	-1.79551800
H	-0.80324000	-2.90339900	-1.26479000
C	1.86476200	0.53544500	-1.12385400
C	3.01125900	-0.29210800	-1.12920200
C	2.03145300	1.89070100	-0.75906100
C	4.26376700	0.21550600	-0.78929200
H	2.90602300	-1.33680900	-1.40774900
C	3.28526800	2.39523000	-0.41811200
H	1.16558400	2.54732200	-0.75686300
C	4.40908400	1.56106000	-0.42980700
H	5.13254100	-0.43699700	-0.81132500
H	3.38927600	3.44264500	-0.14792800
H	5.38673400	1.95593500	-0.16916100

Allyl-TS4

C	0.42351700	1.38048600	0.05253100
C	1.79151100	1.42905300	0.22036200
C	0.51881200	-1.03377800	-0.47699000
C	1.90517600	-0.96435700	-0.32922500
C	2.55423300	0.23782500	0.07591200
C	4.05065500	0.25804100	0.29035200
H	4.39420900	-0.62903600	0.83203100
H	4.60143800	0.28085700	-0.66149500
H	4.36631300	1.13161400	0.86313400
C	2.75429200	-2.18917600	-0.61988800
H	2.28338100	-2.84211900	-1.35636200
H	3.73481600	-1.91365700	-1.01449400
H	2.92731900	-2.78967700	0.28419600
C	-0.14642600	-2.34168500	-0.84740400
H	0.31071600	-3.18315800	-0.31556500
H	-0.06009000	-2.54374200	-1.92254700
C	2.45055100	2.75935500	0.51989900
H	2.91406100	2.77041800	1.51405500
H	3.23613500	3.00061900	-0.20516700
H	1.71327500	3.56627400	0.49361800
C	-0.29423200	0.13711500	-0.14725800
H	-0.15985000	2.29083800	0.13491200
C	-1.61487300	0.30760800	-0.86752000
O	-2.02931700	-0.39045100	-1.77143600
O	-2.30655400	1.38179200	-0.41189400
C	-3.54540800	1.65815700	-1.08986000
H	-3.94612100	2.54982800	-0.60751500
H	-3.36690100	1.84206300	-2.15216600
H	-4.23522800	0.81785100	-0.98308900
C	-0.97631700	-0.21765700	1.72704500
H	0.00532700	-0.41337500	2.15073400
H	-1.35515800	0.77686100	1.94941400
H	-1.21133500	-2.33327400	-0.62532400
C	-1.94915700	-1.29027600	1.82755200
C	-3.28954700	-1.11677100	1.89731500
H	-3.73115000	-0.12354400	1.91290300
H	-3.96890600	-1.96151500	1.95309200
H	-1.56199600	-2.30848800	1.82534200

Propargyl-TS4

C	0.42378100	1.39136200	0.09989300
C	1.79683700	1.39531700	0.23170100
C	0.42861100	-1.01712600	-0.45955900
C	1.82126500	-0.99280800	-0.35119300
C	2.51796800	0.18353600	0.04686700
C	4.01974100	0.15716400	0.22058100
H	4.34933000	-0.73781000	0.75788700
H	4.54403900	0.15819100	-0.74600800

H	4.37838000	1.02270500	0.77962200
C	2.61477600	-2.24459800	-0.68033700
H	2.13102400	-2.83000300	-1.46467300
H	3.62055800	-2.00562600	-1.03079400
H	2.72239000	-2.90134000	0.19409300
C	-0.29001600	-2.29898700	-0.81549700
H	0.18205900	-3.16170200	-0.33516400
H	-0.28199900	-2.47315500	-1.89900900
C	2.50365100	2.70060400	0.53257000
H	2.99706400	2.68180700	1.51203700
H	3.27353900	2.93095800	-0.21249100
H	1.79002200	3.52881000	0.54128500
C	-0.33476100	0.17711400	-0.10921000
H	-0.12899900	2.31804400	0.20930800
C	-1.66964600	0.39638000	-0.79151500
O	-2.11214000	-0.26243600	-1.71055200
O	-2.32829600	1.46275400	-0.27761700
C	-3.59683300	1.76580600	-0.88645000
H	-3.96508800	2.64786900	-0.36278800
H	-3.47006400	1.97330000	-1.95176900
H	-4.28654600	0.92782900	-0.76203300
C	-1.01723500	-0.19607600	1.78033600
H	-0.08041600	-0.55779500	2.19902400
H	-1.25365300	0.82492900	2.06981200
H	-1.33410600	-2.27316200	-0.51122000
C	-2.11247000	-1.09322200	1.84247100
C	-3.06688500	-1.85064300	1.84836200
H	-3.89674000	-2.51932800	1.86411800

_i_-Pr-TS4

C	-0.04642600	-1.20385000	-0.29893000
C	-1.39939400	-1.47215400	-0.35339600
C	-0.46729100	1.22419400	-0.25483000
C	-1.83451800	0.93963500	-0.36734400
C	-2.32544800	-0.39423900	-0.37000200
C	-3.81318600	-0.66500100	-0.42055200
H	-4.36438900	-0.03464100	0.28537500
H	-4.23462000	-0.47003100	-1.41708200
H	-4.04349500	-1.70241000	-0.17401700
C	-2.81794900	2.08946400	-0.50488200
H	-2.41373000	2.88945200	-1.12996900
H	-3.75584800	1.76939300	-0.96056600
H	-3.06568700	2.53618100	0.46826000
C	-0.00944500	2.66683500	-0.18544600

H	-0.72981800	3.28283600	0.36055500
H	0.09827300	3.10541800	-1.18749800
C	-1.85787600	-2.91354400	-0.44436700
H	-2.44945000	-3.21288800	0.42970700
H	-2.47962600	-3.09119400	-1.32944700
H	-0.99705700	-3.58517400	-0.50314500
C	0.48558500	0.12590600	-0.14010700
H	0.65433700	-2.02828300	-0.34658900
C	1.89308000	0.34563200	-0.64307500
O	2.51830100	1.39124100	-0.62497200
O	2.44206700	-0.79213400	-1.14261400
C	3.79046800	-0.67232500	-1.62789100
H	4.05744200	-1.66710400	-1.98479700
H	3.84088500	0.05561200	-2.44111500
H	4.46352200	-0.35634100	-0.82659500
C	1.09596400	0.20546600	1.91874600
H	1.53234700	1.20214100	1.86436000
H	0.96256100	2.76224400	0.29229200
C	2.09544600	-0.89390100	2.16877400
C	-0.17562100	0.12171800	2.71662100
H	2.39793600	-0.89522000	3.22788000
H	1.66571300	-1.88126400	1.96040900
H	3.00661100	-0.78125600	1.57450600
H	0.04631600	0.20998800	3.79168100
H	-0.87827900	0.91831100	2.45841700
H	-0.67723200	-0.84165700	2.56815900

CH_2OMe-TS4

C	0.46521400	1.40415400	0.08558400
C	1.83387600	1.40876300	0.28074700
C	0.51691500	-0.97054200	-0.59531800
C	1.90494600	-0.93958400	-0.42913100
C	2.57567200	0.21881100	0.05753800
C	4.07096800	0.19492000	0.28701900
H	4.38691100	-0.71477800	0.80849000
H	4.63381900	0.23071900	-0.65706400
H	4.40182300	1.04329300	0.88814500
C	2.72360500	-2.16738800	-0.78857000
H	2.28958900	-2.70475500	-1.63444200
H	3.74623800	-1.90457400	-1.06503100
H	2.78545800	-2.87693200	0.04835400
C	-0.17036100	-2.24935100	-1.02363900
H	0.33360300	-3.12521000	-0.60539700
H	-0.18258100	-2.35745500	-2.11592000

C	2.51102500	2.70167600	0.68719500
H	2.97492400	2.62691500	1.67839000
H	3.30015500	2.99045000	-0.01662700
H	1.78481600	3.51817000	0.72627300
C	-0.26454200	0.20157900	-0.22943700
H	-0.09602600	2.32198100	0.21711600
C	-1.60452600	0.41861000	-0.87616100
O	-2.12214500	-0.28456600	-1.72498200
O	-2.21993600	1.54345400	-0.41481400
C	-3.47375000	1.86804900	-1.03774600
H	-3.79426800	2.80141700	-0.57448300
H	-3.34549900	1.99659900	-2.11527400
H	-4.21029200	1.07955900	-0.86096600
C	-0.96705200	-0.29195600	1.70806600
H	-1.20827800	-2.27505200	-0.69857000
H	-1.50165500	0.62957400	1.95189800
O	-1.67742300	-1.44653100	1.88112300
C	-3.09088100	-1.35481500	1.70243400
H	-3.50975800	-2.29876000	2.05485900
H	-3.50182800	-0.52693200	2.29507000
H	-3.34813300	-1.21631000	0.64692900
H	0.03035900	-0.39432700	2.12615200

Bn-TS5

C	0.67918300	0.42540100	-1.36850300
C	1.54910600	1.46671300	-1.09895100
C	0.32955400	0.01081100	1.02129400
C	1.18875400	1.08388400	1.29634900
C	1.83863700	1.79234200	0.25186800
C	2.81086300	2.90537500	0.57832200
H	2.29545700	3.81237300	0.92488000
H	3.50640300	2.61368900	1.37221700
H	3.40925100	3.18885900	-0.28860400
C	1.41268600	1.50625400	2.73679300
H	0.51206300	1.36855600	3.33938800
H	2.21185400	0.92005000	3.21185600
H	1.69416600	2.55781300	2.81414900
C	-0.33408700	-0.75559100	2.14382500
H	-0.64294700	-1.75038600	1.82113600
H	-1.22984500	-0.23726500	2.51133000
C	2.15653700	2.23720400	-2.25190100
H	1.96576100	3.31389700	-2.17658300
H	3.24436100	2.10389000	-2.30021000
H	1.74260900	1.89177000	-3.20318200
C	0.11844200	-0.40699000	-0.34838000
H	0.44927000	0.18252400	-2.40365000
C	1.44128400	-2.08219700	-0.33692700
O	1.13304200	-3.12981600	0.17692500
O	2.67398000	-1.70320700	-0.68005200
C	3.73958100	-2.65227900	-0.39678900
H	4.65288500	-2.17363500	-0.74805900
H	3.79002000	-2.84639300	0.67709000
H	3.56027300	-3.58794700	-0.93102100
C	-1.13176100	-1.21114300	-0.72273300
H	-1.05654900	-1.45856600	-1.78977700
H	-1.14032100	-2.16833900	-0.19709700
H	0.33953500	-0.88946300	2.99525700
C	-2.45647100	-0.50184200	-0.47197300
C	-2.67035800	0.83511400	-0.84213800
C	-3.52271900	-1.20570100	0.10716100
C	-3.91104700	1.44540300	-0.64274100
H	-1.85834200	1.40512600	-1.28405800
C	-4.76664100	-0.60010100	0.30727900
H	-3.37692200	-2.24158400	0.40464800
C	-4.96583200	0.73051700	-0.06754700
H	-4.05327600	2.48180100	-0.93696500
H	-5.57606000	-1.16758000	0.75841000
H	-5.93009400	1.20594800	0.08801000

Allyl-TS5

C	0.20094900	-0.27724000	1.47938800
C	1.49595600	-0.69977100	1.23902800
C	-0.40199400	-0.56951100	-0.87811700
C	0.90344300	-1.02399600	-1.12050600
C	1.87334300	-1.05130200	-0.08370000
C	3.29504900	-1.48157500	-0.37763400
H	3.37087100	-2.56546300	-0.54297500
H	3.68748000	-0.99462100	-1.27647800
H	3.96997400	-1.23554900	0.44311600
C	1.27716200	-1.51919100	-2.50720200
H	0.47439400	-2.11663700	-2.94709300
H	1.47617500	-0.68770700	-3.19699000
H	2.16885300	-2.14656300	-2.49103400
C	-1.43790400	-0.50459600	-1.97688800
H	-2.03208700	0.41015100	-1.89491300
H	-2.13551200	-1.35064800	-1.91964300
C	2.46833800	-0.78723700	2.39627000
H	2.91419600	-1.78423500	2.48779300

H	3.29323800	-0.07200600	2.28906500
H	1.96159500	-0.56198700	3.33863200
C	-0.76005900	-0.09155500	0.43896600
H	-0.09216400	-0.01836300	2.49465900
C	-0.61538600	2.01044200	0.02523900
O	-1.45723400	2.59056800	-0.61621200
O	0.61268000	2.45423700	0.30126200
C	0.95600600	3.76443500	-0.23050000
H	1.97532300	3.95325800	0.10403800
H	0.90067600	3.74983300	-1.32144200
H	0.27129200	4.51956000	0.16190300
C	-2.23182700	-0.05408900	0.86793600
H	-2.28804400	0.50774000	1.81016800
H	-2.84176600	0.49233900	0.14514300
H	-0.98931900	-0.50946000	-2.97109800
C	-2.80180300	-1.43488800	1.08873900
H	-2.23440500	-2.08267100	1.75776200
C	-3.93192400	-1.89446500	0.54296700
H	-4.30162700	-2.89327000	0.75735500
H	-4.52878800	-1.28490800	-0.13229100

Propargyl-TS5

C	-0.06631000	-0.40568700	-1.44124100
C	-1.39511600	-0.72090300	-1.22667500
C	0.41416200	-0.52398000	0.96054200
C	-0.93386900	-0.84558200	1.18312200
C	-1.85547400	-0.91073100	0.10420600
C	-3.31665300	-1.21761900	0.35989900
H	-3.46542500	-2.25050300	0.70224800
H	-3.73949100	-0.56253400	1.12950800
H	-3.92049400	-1.08785200	-0.53840200
C	-1.39691300	-1.16300700	2.59524500
H	-0.78766000	-1.95550200	3.04263300
H	-1.32011200	-0.28892100	3.25368700
H	-2.43168800	-1.50138100	2.62619500
C	1.42661600	-0.46811500	2.08003000
H	1.95195200	0.49433800	2.08042000
H	2.18744100	-1.24993700	1.96142000
C	-2.31540400	-0.86570900	-2.42045100
H	-2.81085600	-1.84296500	-2.44436300
H	-3.10258300	-0.10187100	-2.42307600
H	-1.75176500	-0.75733300	-3.35102300
C	0.85082900	-0.18321400	-0.37219100
H	0.29242000	-0.27181800	-2.45902300
C	0.82420400	1.95157600	-0.14004700
O	1.67203600	2.52256400	0.50281200
O	-0.36176200	2.44328600	-0.49959200
C	-0.65216000	3.80211500	-0.06642200
H	-1.64334500	4.02392100	-0.45977900
H	-0.64536100	3.85404300	1.02472800
H	0.09131800	4.49161500	-0.47226800
C	2.34571800	-0.24521800	-0.72104800
H	2.49656100	0.22857800	-1.69826200
H	2.93049400	0.34056300	-0.00592300
H	0.97415000	-0.59503800	3.06278800
C	2.86296300	-1.61682900	-0.77333100
C	3.29712000	-2.74579700	-0.82054900
H	3.67396500	-3.74198900	-0.86379500

***i*-Pr-TS5**

C	0.08103500	0.44623100	-1.37777200
C	1.45094100	0.24941900	-1.39114800
C	0.07745000	0.71304400	1.05272400
C	1.46927400	0.53378100	1.03874700
C	2.16573500	0.26334800	-0.16662000
C	3.65950400	0.01773400	-0.14908200
H	4.22630800	0.94183900	0.03223900
H	3.94416800	-0.68837500	0.63820300
H	4.01339900	-0.39372600	-1.09522500
C	2.24090900	0.64479000	2.34118700
H	1.83313900	1.42998800	2.98296300
H	2.20157600	-0.29063600	2.91682400
H	3.29348400	0.87846400	2.17547500
C	-0.63077100	0.95527000	2.36914800
H	-0.48707600	1.98416300	2.72657400
H	-0.25169300	0.29179000	3.15307300
C	2.14940800	0.03993300	-2.71823400
H	2.96116100	0.75934200	-2.87577800
H	2.58654500	-0.96339800	-2.79456400
H	1.44194600	0.15129200	-3.54443100
C	-0.68172500	0.58815700	-0.17661800
H	-0.44729700	0.44106900	-2.32535000
C	-1.38034400	-1.40582100	0.20720500
O	-2.23342600	-1.62846600	1.03154300
O	-0.53803900	-2.29997100	-0.31719800
C	-0.67039500	-3.66597600	0.16460700
H	0.08325900	-4.23687100	-0.37650800
H	-0.48666100	-3.70146600	1.24099000

H	-1.67257200	-4.04446300	-0.04876800
C	-2.07267000	1.24644700	-0.28643200
H	-2.66097500	0.92815200	0.57872600
H	-1.70305300	0.77729400	2.29644700
C	-2.87104300	0.83135900	-1.53274700
C	-1.93936900	2.78261800	-0.23580400
H	-3.89609200	1.20876800	-1.45420600
H	-2.44474600	1.24488200	-2.45355200
H	-2.92413900	-0.25685300	-1.63838000
H	-2.92880200	3.25339200	-0.26866200
H	-1.43657700	3.11999000	0.67538700
H	-1.36122400	3.15086200	-1.09118900

CH_2OMe-TS5

C	-0.10098600	-0.39301800	-1.40310900
C	-1.43007500	-0.75830600	-1.30076400
C	0.19421900	-0.54718800	1.02587900
C	-1.16361800	-0.88114900	1.14003100
C	-1.98959900	-0.97570600	-0.01268800
C	-3.45225100	-1.34552500	0.12414300
H	-3.58215500	-2.32950100	0.59172200
H	-3.99591300	-0.62389000	0.74627000
H	-3.95625800	-1.37853400	-0.84168300
C	-1.74229700	-1.18468300	2.51205300
H	-2.82740100	-1.27864500	2.49214600
H	-1.34492500	-2.12445900	2.91584500
H	-1.50016000	-0.39747400	3.23331300
C	1.12272600	-0.52164000	2.21619600
H	2.00092600	-1.14795200	2.02586700
H	0.64938700	-0.88713600	3.12685100
C	-2.24375000	-0.92211200	-2.56755100
H	-2.67130100	-1.92745100	-2.65708400
H	-3.07604800	-0.20942200	-2.61471300
H	-1.61670300	-0.75119900	-3.44675600
C	0.72456900	-0.16581500	-0.26205000
H	0.33310300	-0.22901400	-2.38644300
C	0.64504100	1.95580000	0.00492100
O	1.33659400	2.52810800	0.81244100
O	-0.46036900	2.43494800	-0.56840400
C	-0.85870600	3.77678900	-0.17220800
H	-1.76197400	3.98916000	-0.74277100
H	-1.06080800	3.80341900	0.90101100
H	-0.06808800	4.49019700	-0.41512600
C	2.22657100	-0.17763400	-0.49408800
H	2.44910600	0.30518900	-1.45881600
H	2.75639500	0.38172800	0.28706300
H	1.48459900	0.49685900	2.40942000
O	2.67791700	-1.53062400	-0.51764700
C	4.07274800	-1.63094400	-0.74040600
H	4.32336100	-2.69421500	-0.73720500
H	4.35715100	-1.19769700	-1.71218200
H	4.64704100	-1.12263200	0.05000100

14

C	-0.39293900	1.72756000	0.45502300
C	-0.63640400	1.66957600	-0.87399200
C	-1.63212200	0.66419400	-1.39723800
C	-1.68112600	-0.66552800	-0.60742400
C	-1.58527600	-0.41767200	0.90087400
C	-2.15320700	-1.49510700	1.82020400
C	0.49880500	2.80434100	1.05563100
H	-2.75072300	-1.02430200	2.60497700
H	-2.85534400	-2.12278700	1.26696800
H	0.03396600	3.20175300	1.96054000
H	0.56049100	3.65015600	0.37080700
H	-1.44494200	0.44909700	-2.45351900
C	-1.01401400	0.72154000	1.36806100
C	-1.03608100	1.03897700	2.85637900
H	-1.14603100	0.12720200	3.44047500
H	-0.08577200	1.47714700	3.16791100
C	-0.08482300	2.62483600	-1.90823000
H	0.21808500	2.04207100	-2.78570800
H	0.81637700	3.12066100	-1.54508900
C	-0.60385900	-1.69317800	-1.11507500
H	-0.68933900	-2.60545600	-0.52161800
H	-0.86618000	-1.95690600	-2.13936500
H	-2.62489700	1.12474500	-1.35134100
C	0.82301900	-1.24621000	-1.12231100
C	1.46373000	-0.74407700	-2.27170700
C	2.89981000	-1.11428200	0.08800600
C	2.80003700	-0.42698300	-2.24039600
H	0.88477000	-0.63431700	-3.17786800
C	3.56026300	-0.60094500	-1.05820800
H	3.29120500	-0.04719500	-3.12926500
C	4.94205300	-0.30459900	-0.96351300
C	3.59444500	-1.33460400	1.29010900
C	5.61518200	-0.51833700	0.21636500
H	6.67204800	-0.29148600	0.28758900

C	4.93874100	-1.03690600	1.34337100
H	5.48587900	-1.20365800	2.26361400
H	5.45173000	0.08950500	-1.83489100
H	3.06934500	-1.73503100	2.14951200
N	1.55860000	-1.40415500	-0.01450100
H	1.10178400	-1.78201600	0.81238300
C	-1.12281100	-2.43190100	2.47870400
H	-0.58500400	-3.02696500	1.73491200
H	-0.38727700	-1.88376000	3.07291400
H	-1.62851900	-3.13520200	3.14732600
C	-2.18115500	1.99835000	3.23014900
H	-3.15339400	1.55344800	3.00034900
H	-2.15798200	2.22570500	4.30019300
H	-2.11433700	2.94352200	2.68474300
C	-1.09973600	3.69166000	-2.36123100
H	-2.00432900	3.23891300	-2.77621600
H	-1.39867900	4.33052600	-1.52530500
H	-0.66307100	4.33032200	-3.13463100
C	1.92542400	2.34124200	1.39263400
H	2.49337800	3.15451300	1.85500500
H	1.92595100	1.50017200	2.09111000
H	2.46214200	2.03003000	0.49249500
C	-3.04378400	-1.30718600	-0.95504600
O	-3.19377100	-2.31976600	-1.59882200
O	-4.06319700	-0.57659900	-0.48500600
C	-5.39391800	-1.05575100	-0.78769200
H	-6.06958700	-0.34276400	-0.32063700
H	-5.55335200	-1.07939000	-1.86652200
H	-5.54437600	-2.05232500	-0.37040300

VI. X-Ray Crystal Analysis

Crystallographic Data of (±)-2b

Compound (±)-**2b** was dissolved in *n*-hexane. The solvent was slowly evaporated at ambient temperature to afford the single crystal.

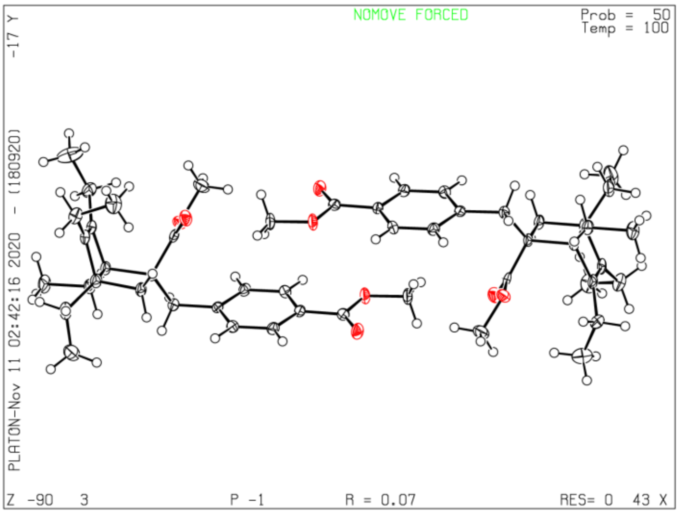

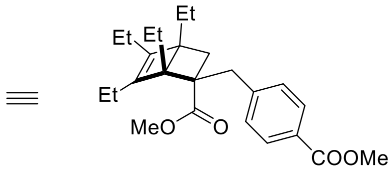

2b

CCDC 2044047

Fig. S16 Crystallographic data of (±)-**2b**.

Table S4. Crystal data and structure refinement for (±)-**2b**.

Empirical formula	$C_{25}H_{34}O_4$
Formula weight	398.52
Temperature/K	100 K
Space group	P -1
Hall group	-P 1
a/Å	11.4311(6)
b/Å	11.4678(6)
c/Å	17.8123(9)
α/°	97.954(4)
β/°	103.843(4)
γ/°	95.162(4)
Volume/Å^3	2226.9(2)
Z	4
ϱ g/cm^3	1.189
Mu/mm^{-1}	0.079
F(000)	864.0
h,k,l_{max}	13,13,21
N_{ref}	7830
T_{min},T_{max}	0.612,1.000

VII. Mechanism for Alkyl Transfer Allylation Reaction

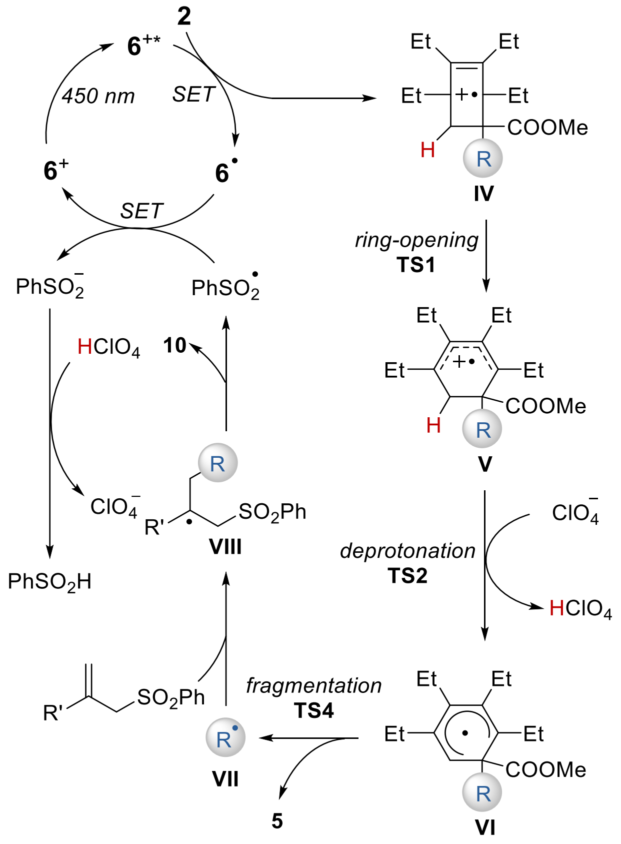

Fig. S17 Proposed mechanism for alkyl transfer allylation reaction.

VIII. SI References and Notes

(1) Li, J.; Lear, M. J.; Hayashi, Y. Sterically Demanding Oxidative Amidation of A-Substituted Malononitriles with Amines Using O2. *Angew. Chem., Int. Ed.* **2016**, *55*, 9060-9064.

(2) Xiang, L.; Liu, X.; He, Y.; Zhang, K. Eye-Readable Dynamic Covalent Click Reaction and Its Application in Polymer Synthesis. *Macromolecules* **2020**, *53*, 5434-5444.

(3) Liu, H.; Ge, L.; Wang, D.-X.; Chen, N.; Feng, C. Photoredox-Coupled F-Nucleophilic Addition: Allylation of Gem-Difluoroalkenes. *Angew. Chem., Int. Ed.* **2019**, *58*, 3918-3922.

(4) Van Rantwijk, F.; Van der Stoel, R. E.; Van Bekkum, H. Preparation and Aluminum Trichloride-Induced Cationic Rearrangements of Bicyclo[2.2.0]Hexane Carboxylic Esters. *Tetrahedron* **1978**, *34*, 569-575.

(5) Chen, C.; Yan, X.; Xi, C. Cycloaddition of Zirconacyclopentadiene with 2-Bromoacrylate, 2-Bromoacrylaldehyde, and 3-Bromofuran-2,5-Dione in the Presence of CuCl: A New Pathway for the Formation of Benzene Derivatives and Isobenzofuran-1,3-Dione. *Synth. Commun.* **2010**, *40*, 570-579.

(6) Nakajima, K.; Zhang, Y.; Nishibayashi, Y. Alkylation Reactions of Azodicarboxylate Esters with 4-Alkyl-1,4-Dihydropyridines under Catalyst-Free Conditions. *Org. Lett.* **2019**, *21*, 4642-4645.

(7) Feng, G.; Wang, X.; Jin, J. Decarboxylative C–C and C–N Bond Formation by Ligand-Accelerated Iron Photocatalysis. *Eur. J. Org. Chem.* **2019**, *2019*, 6728-6732.

(8) Rasmussen, L. K. Facile Synthesis of Mono-, Di-, and Trisubstituted Alpha-Unbranched Hydrazines. *J. Org. Chem.* **2006**, *71*, 3627-3629.

(9) Yatham, V. R.; Bellotti, P.; König, B. Decarboxylative Hydrazination of Unactivated Carboxylic Acids by Cerium Photocatalysis. *Chem. Commun.* **2019**, *55*, 3489-3492.

(10) Uchikura, T.; Moriyama, K.; Toda, M.; Mouri, T.; Ibáñez, I.; Akiyama, T. Benzothiazolines as Radical Transfer Reagents: Hydroalkylation and Hydroacylation of Alkenes by Radical Generation under Photoirradiation Conditions. *Chem. Commun.* **2019**, *55*, 11171-11174.

(11) Wu, Q.-Y.; Min, Q.-Q.; Ao, G.-Z.; Liu, F. Radical Alkylation of Para-Quinone Methides with 4-Substituted Hantzsch Esters/Nitriles Via Organic Photoredox Catalysis. *Org. Biomol. Chem.* **2018**, *16*, 6391-6394.

(12) Wang, X.; Dong, J.; Li, Y.; Liu, Y.; Wang, Q. Visible-Light-Mediated Manganese-Catalyzed Allylation Reactions of Unactivated Alkyl Iodides. *J. Org. Chem.* **2020**, *85*, 7459-7467.

(13) Chen, H.; Sun, S.; Liu, Y. A.; Liao, X. Nickel-Catalyzed Cyanation of Aryl Halides and Hydrocyanation of Alkynes Via C–Cn Bond Cleavage and Cyano Transfer. *ACS Catal.* **2020**, *10*, 1397-1405.

(14) Merchant, R. R.; Lang, S. B.; Yu, T.; Zhao, S.; Qi, Z.; Suzuki, T.; Bao, J. A General One-Pot Protocol for Hindered N-Alkyl Azaheterocycles from Tertiary Carboxylic Acids. *Org. Lett.* **2020**, *22*, 4180-4184.

(15) Ha, H.; Shin, C.; Bae, S.; Joo, J. M. Divergent Palladium-Catalyzed Cross-Coupling of Nitropyrazoles with Terminal Alkynes. *Eur. J. Org. Chem.* **2018**, *2018*, 2645-2650.

(16) Zhou, R.; Liu, H.; Tao, H.; Yu, X.; Wu, J. Metal-Free Direct Alkylation of Unfunctionalized Allylic/Benzylic Sp3 C–H Bonds Via Photoredox Induced Radical Cation Deprotonation. *Chem. Sci.* **2017**, *8*, 4654-4659.

(17) Gaussian 09, Revision D.01, Frisch, M. J.; Trucks, G. W.; Schlegel, H. B.; Scuseria, G. E.; Robb, M. A.; Cheeseman, J. R.; Scalmani, G.; Barone, V.; Mennucci, B.; Petersson, G. A.; Nakatsuji, H.; Caricato, M.; Li, X.; Hratchian, H. P.; Izmaylov, A. F.; Bloino, J.; Zheng, G.; Sonnenberg, J. L.; Hada, M.; Ehara, M.; Toyota, K.; Fukuda, R.; Hasegawa, J.; Ishida, M.; Nakajima, T.; Honda, Y.; Kitao, O.; Nakai, H.; Vreven, T.; Montgomery, Jr., J. A.; Peralta, J. E.; Ogliaro, F.; Bearpark, M.; Heyd, J. J.; Brothers, E.; Kudin, K. N.; Staroverov, V. N.; Keith, T.; Kobayashi, R.; Normand, J.; Raghavachari, K.; Rendell, A.; Burant, J. C.; Iyengar,

S. S.; Tomasi, J.; Cossi, M.; Rega, N.; Millam, J. M. ; Klene, M.; Knox, J. E.; Cross, J. B.; Bakken, V.; Adamo, C.; Jaramillo, J.; Gomperts, R.; Stratmann, R. E.; Yazyev, O.; Austin, A. J.; Cammi, R.; Pomelli, C.; Ochterski, J. W.; Martin, R. L.; Morokuma, K.; Zakrzewski, V. G.; Voth, G. A.; Salvador, P.; Dannenberg, J. J.; Dapprich, S.; Daniels, A. D.; Farkas, O.; Foresman, J. B.; Ortiz, J. V.; Cioslowski, J.; Fox, D. J. Gaussian, Inc., Wallingford CT, 2013.

(18) (a) Becke, A. D. Density Functional Thermochemistry. III. The Role of Exact Exchange. *J. Chem. Phys.* **1993**, *98*, 5648−5652. (b) Lee, C.; Yang, W.; Parr, R. G. Development of the Colle-Salvetti Correlation-Energy Formula into a Functional of the Electron Density. *Phys. Rev. B: Condens. Matter Mater. Phys.* **1988**, *37*, 785−789.

(19) W. J. Hehre, L. Radom, P. v. R. Schleyer, J. A. Pople, Ab Initio Molecular Orbital Theory; Wiley: New York, 1986.

(20) Marenich, A. V.; Cramer, C. J.; Truhlar, D. G. Universal Solvation Model Based on Solute Electron Density and on a Continuum Model of the Solvent Defined by the Bulk Dielectric Constant and Atomic Surface Tensions. *J. Phys. Chem. B* **2009**, *113*, 6378−6396.

(21) (a) Fukui, K. Formulation of the Reaction Coordinate. *J. Phys. Chem.* **1970**, *74*, 4161−4163. (b) Fukui, K. The Path of Chemical Reactions-the IRC Approach. *Acc. Chem. Res.* **1981**, *14*, 363−368.

(22) Zhao, Y.; Truhlar, D. G. The M06 Suite of Density Functionals for Main Group Thermochemistry, Thermochemical Kinetics, Noncovalent Interactions, Excited States, and Transition Elements: Two New Functionals and Systematic Testing of four M06-Class Functionals and 12 Other Functionals. *Theor. Chem. Acc.* **2008**, *120*, 215-241.

(23) Weigend, F.; Ahlrichs, R. Balanced Basis Sets of Split Valence,Triple Zeta Valence and Quadruple Zeta Valence Quality for H to Rn: Design and Assessment of Accuracy. *Phys. Chem. Chem. Phys.* **2005**, *7*, 3297−3305.

(24) C. Y. Legault, CYLview, 1.0b, Universitede Sherbrooke, 2009. http://www.cylview.org

(25) Runge, E.; Gross, E. K. U. Density-Functional Theory for Time-Dependent Systems. *Phys. Rev. Lett.* **1984**, *52*, 997-1000

(26) Chai, J.-D.; Head-Gordon, M. Systematic Optimization of Long-Range Corrected Hybrid Density Functionals. *J. Chem. Phys.* **2008**, *128*, 084106.

IX. NMR Spectra

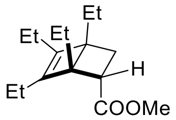

1

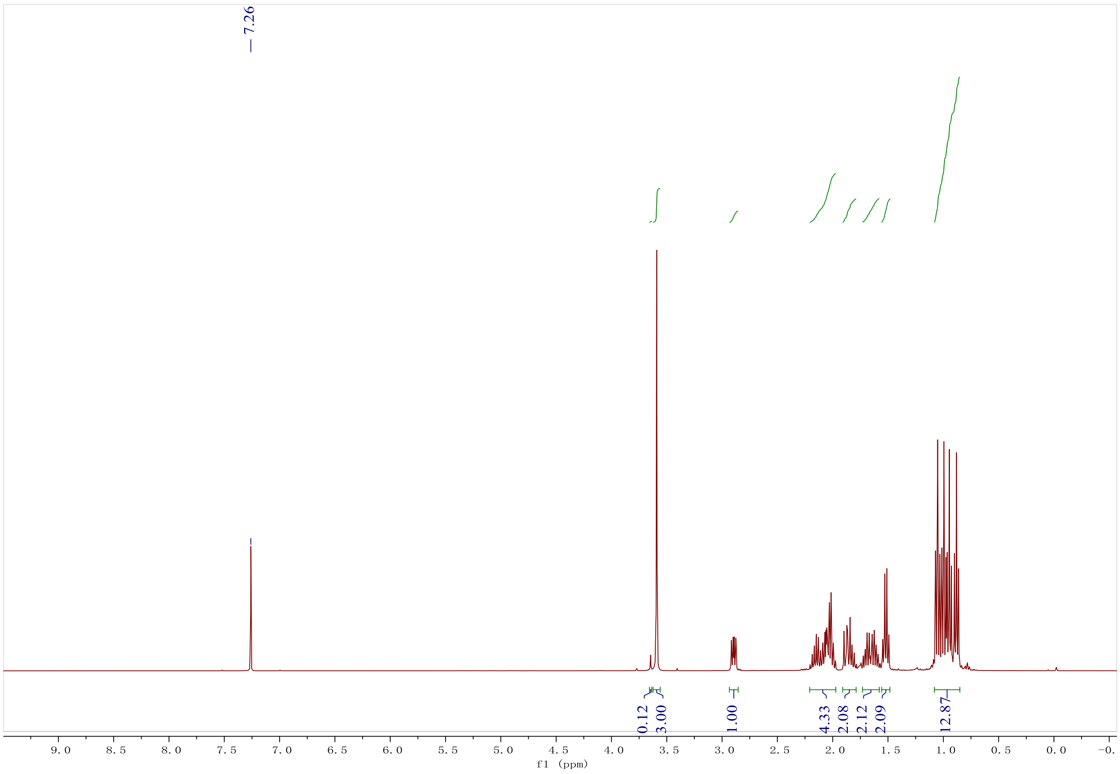

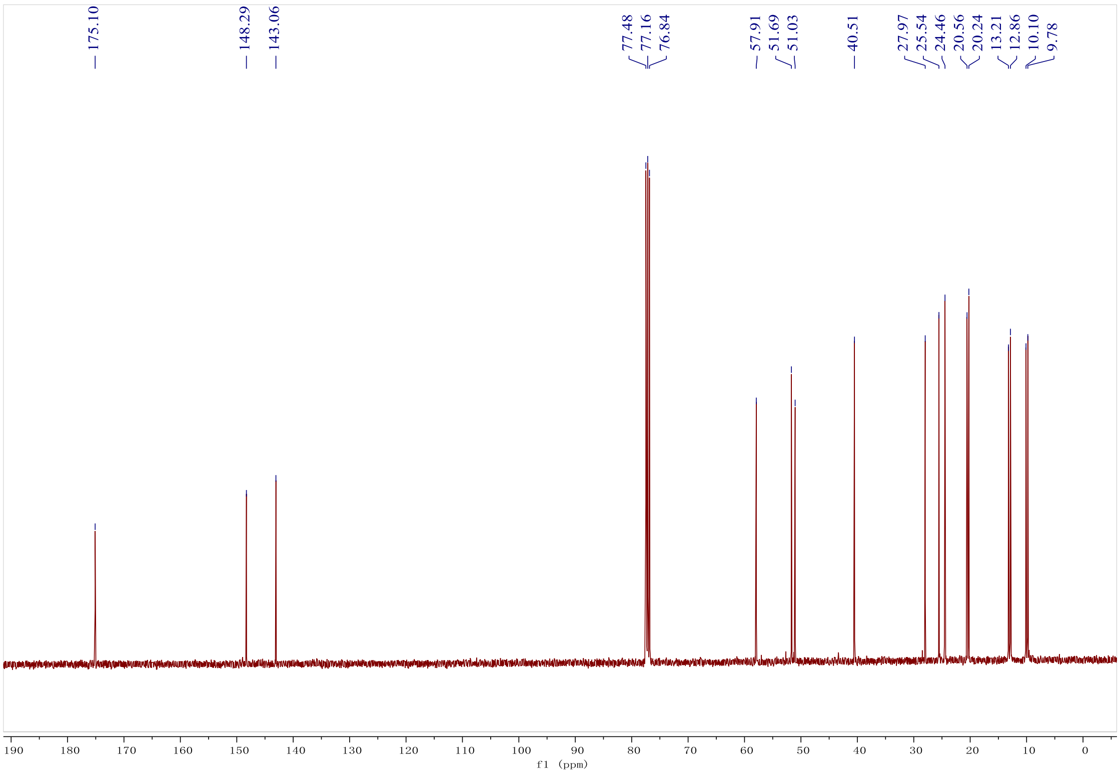

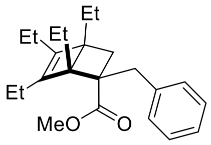
Et
Et
Et
Et
MeO
O

2a

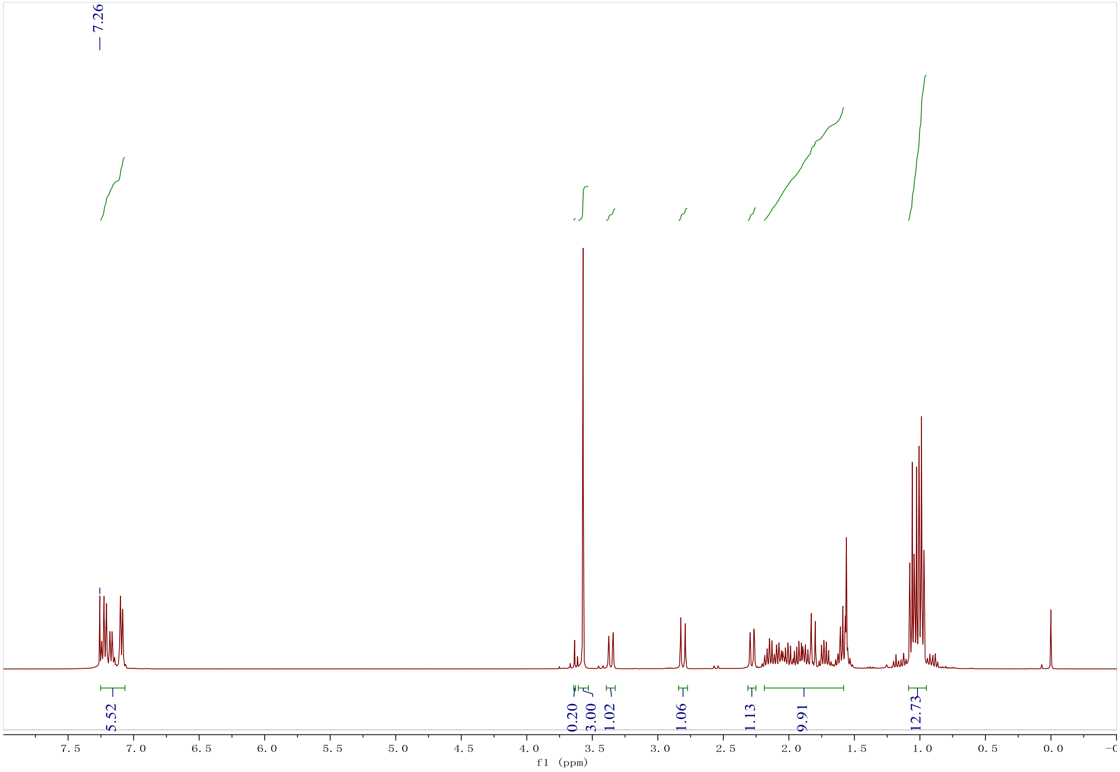
7.26
5.52
0.20
3.00
1.02
1.06
1.13
9.91
12.73
7.5
7.0
6.5
6.0
5.5
5.0
4.5
4.0
3.5
3.0
2.5
2.0
1.5
1.0
0.5
0.0
f1 (ppm)

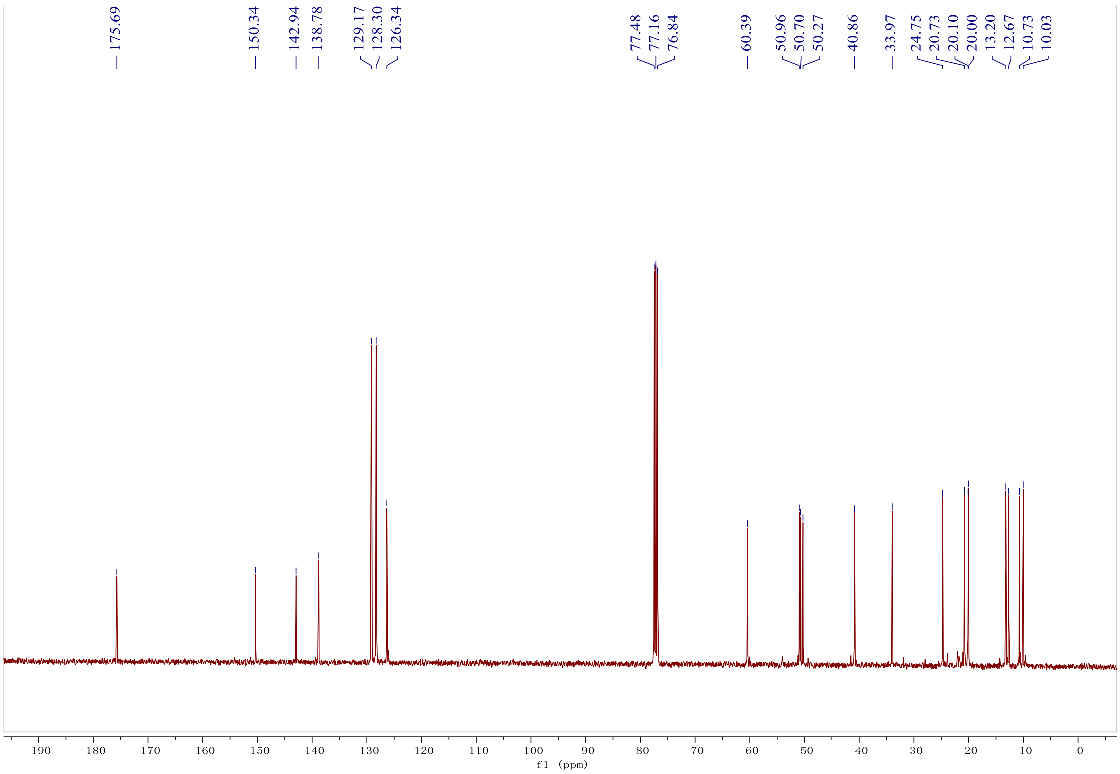
175.69
150.34
142.94
138.78
129.17
128.30
126.34
77.48
77.16
76.84
60.39
50.96
50.70
50.27
40.86
33.97
24.75
20.73
20.10
20.00
13.20
12.67
10.73
10.03
190
180
170
160
150
140
130
120
110
100
90
80
70
60
50
40
30
20
10
0
f1 (ppm)

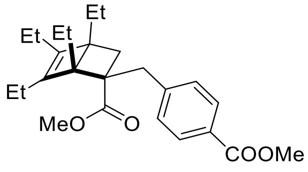
Et
Et
Et
Et
MeO
O
COOMe

2b

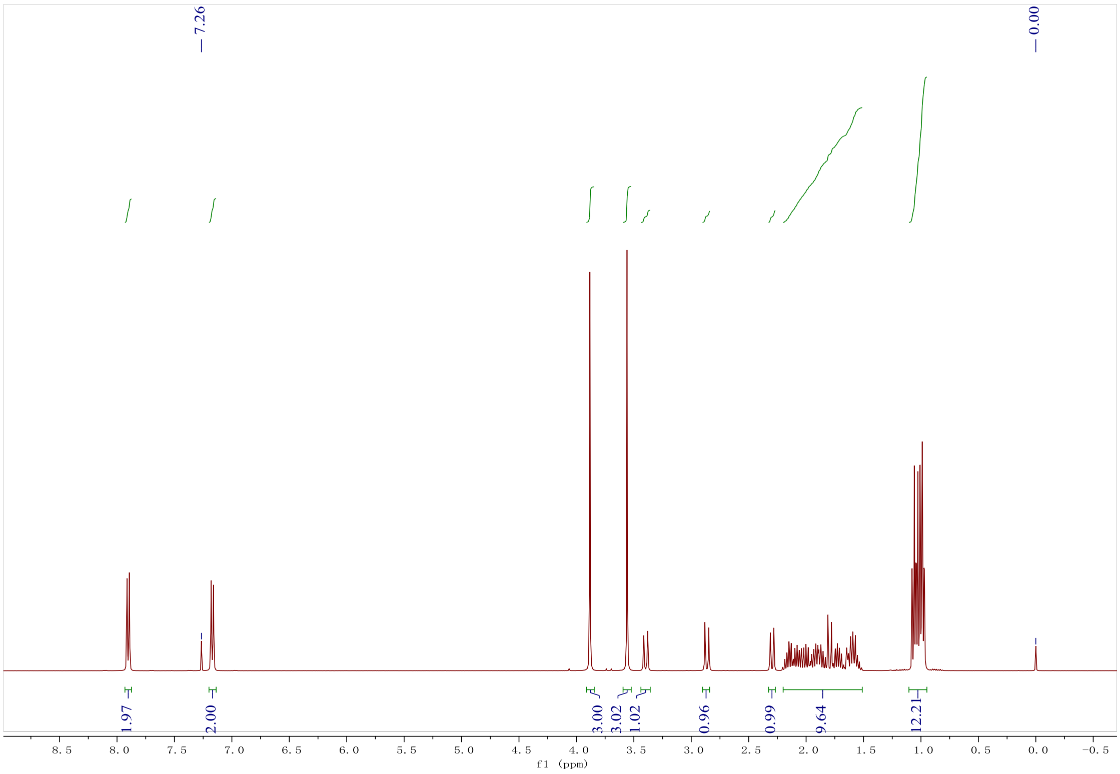
7.26
0.00
1.97
2.00
3.00
3.02
1.02
0.96
0.99
9.64
12.21
8.5 8.0 7.5 7.0 6.5 6.0 5.5 5.0 4.5 4.0 3.5 3.0 2.5 2.0 1.5 1.0 0.5 0.0 -0.5
f1 (ppm)

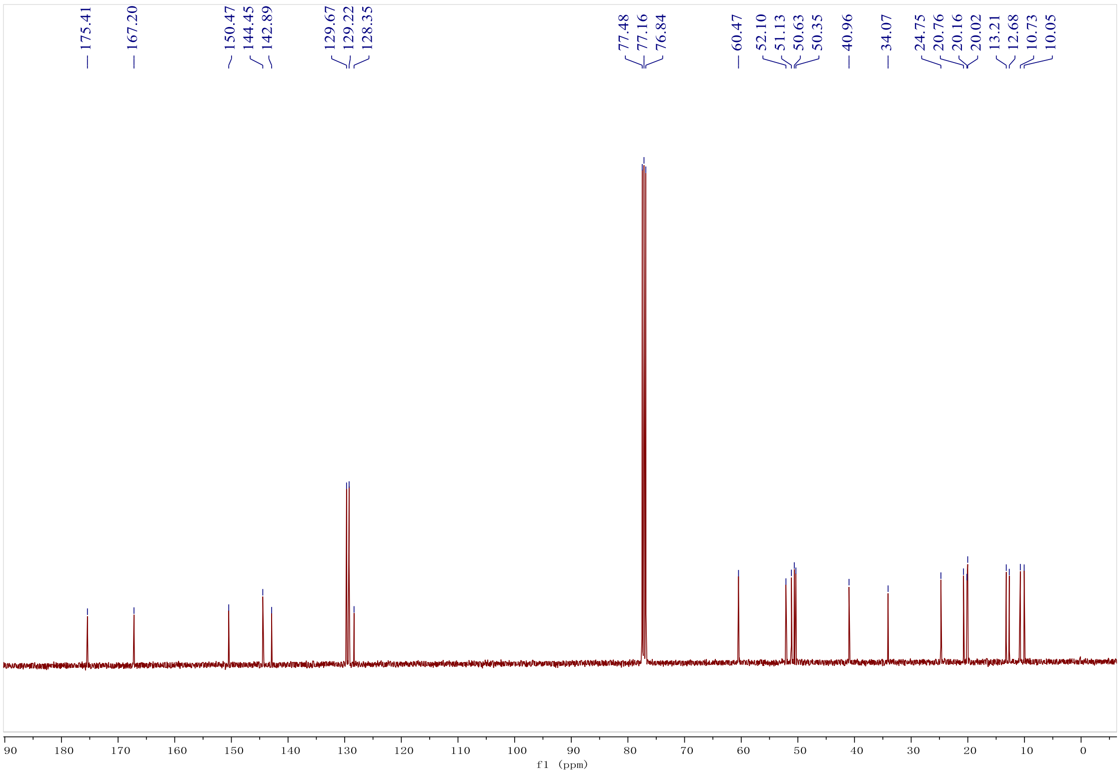
175.41
167.20
150.47
144.45
142.89
129.67
129.22
128.35
77.48
77.16
76.84
60.47
52.10
51.13
50.63
50.35
40.96
34.07
24.75
20.76
20.16
20.02
13.21
12.68
10.73
10.05
190 180 170 160 150 140 130 120 110 100 90 80 70 60 50 40 30 20 10 0
f1 (ppm)

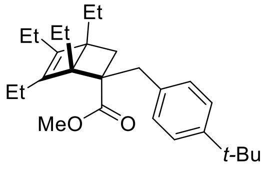
Et
Et
Et
Et
MeO
O
t-Bu

2c

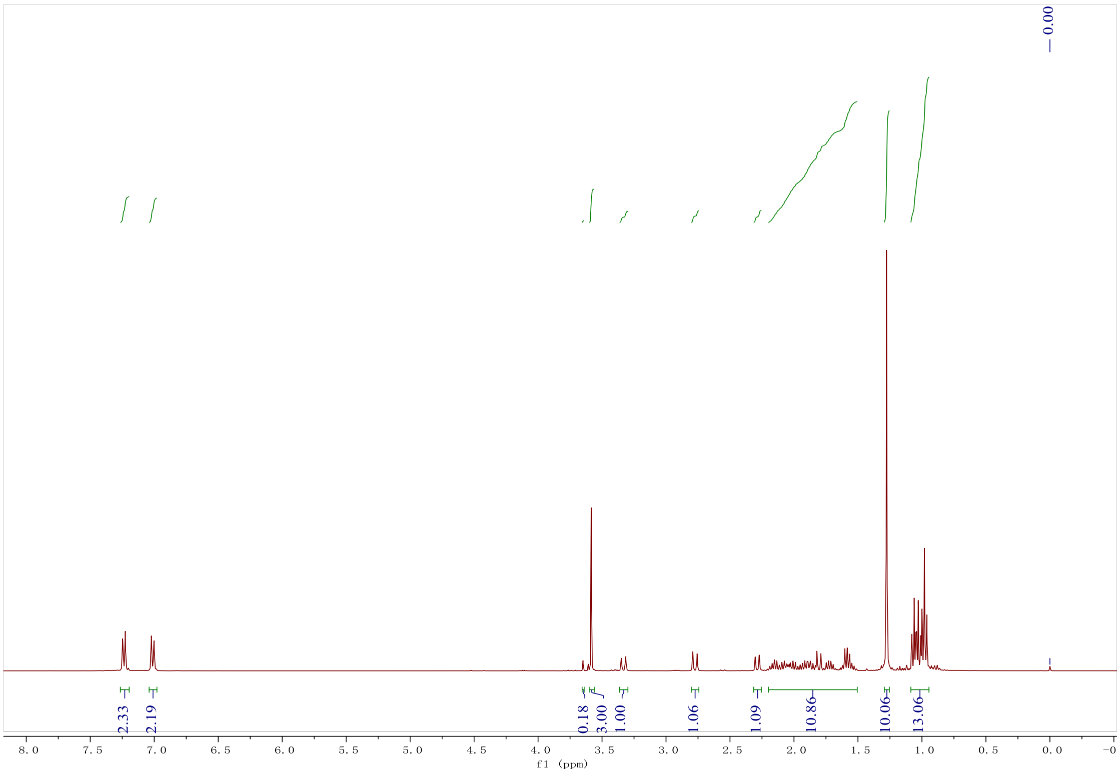
0.00
2.33
2.19
0.18
3.00
1.00
1.06
1.09
10.86
10.06
13.06
f1 (ppm)

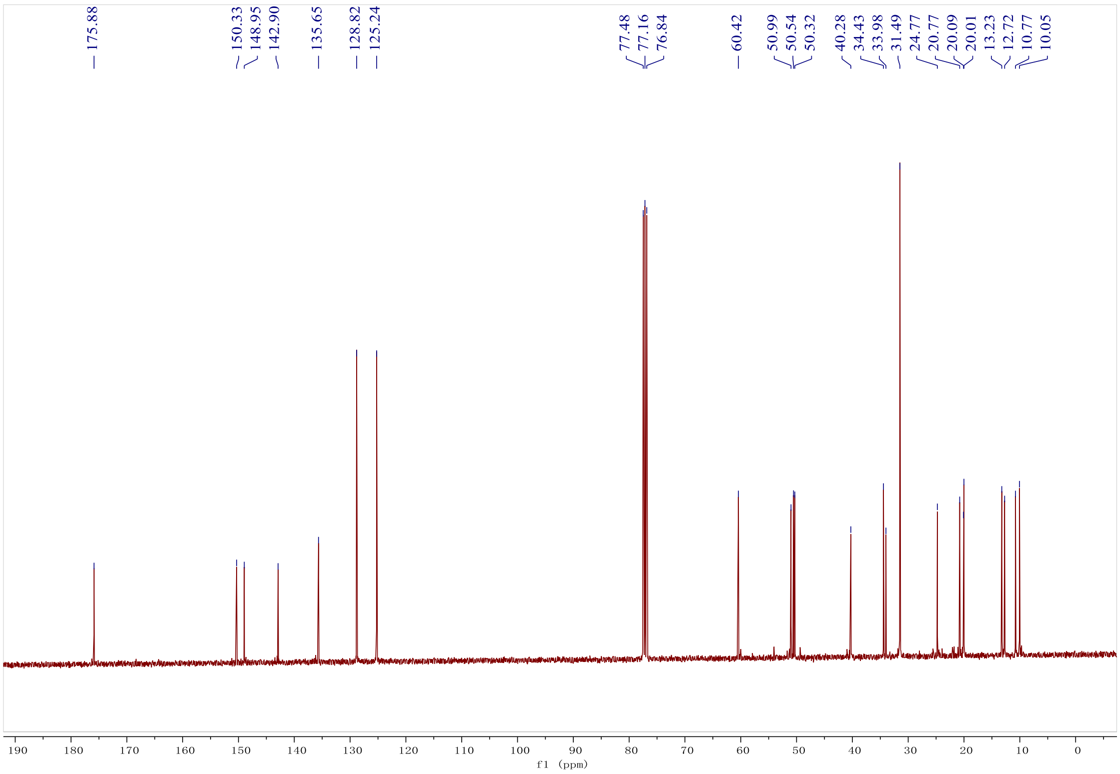
175.88
150.33
148.95
142.90
135.65
128.82
125.24
77.48
77.16
76.84
60.42
50.99
50.54
50.32
40.28
34.43
33.98
31.49
24.77
20.77
20.09
20.01
13.23
12.72
10.77
10.05
f1 (ppm)

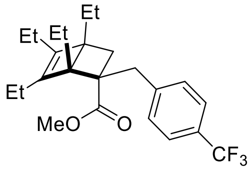

2d

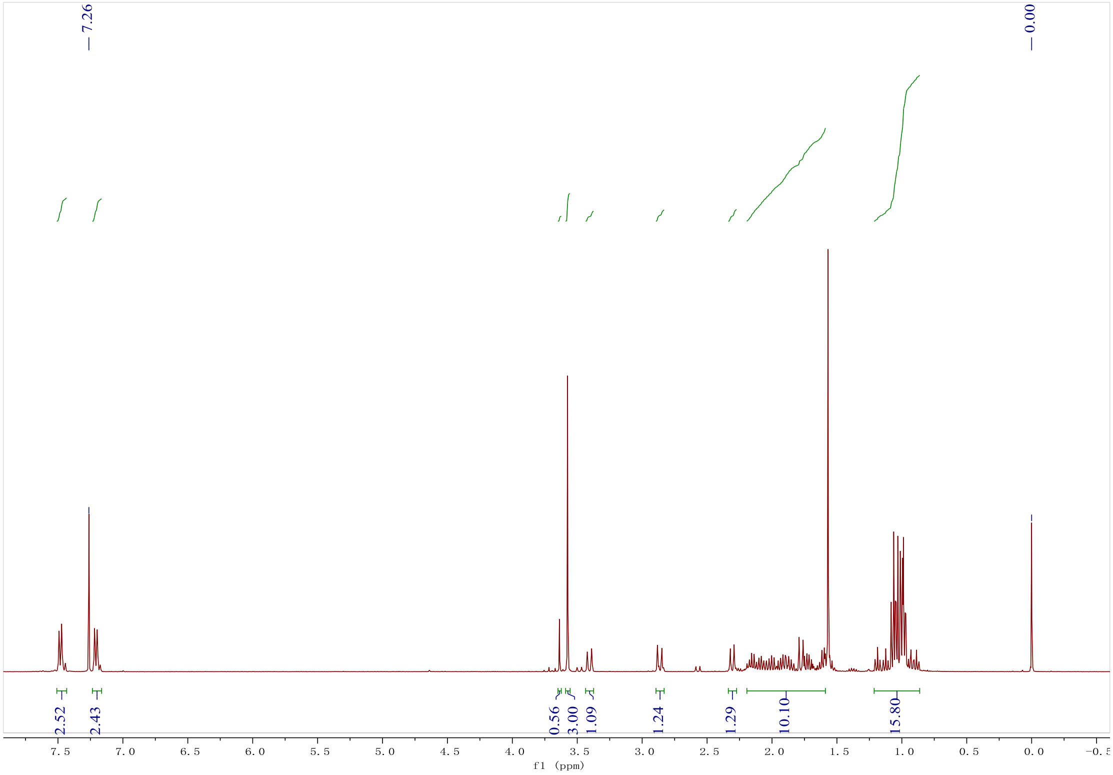

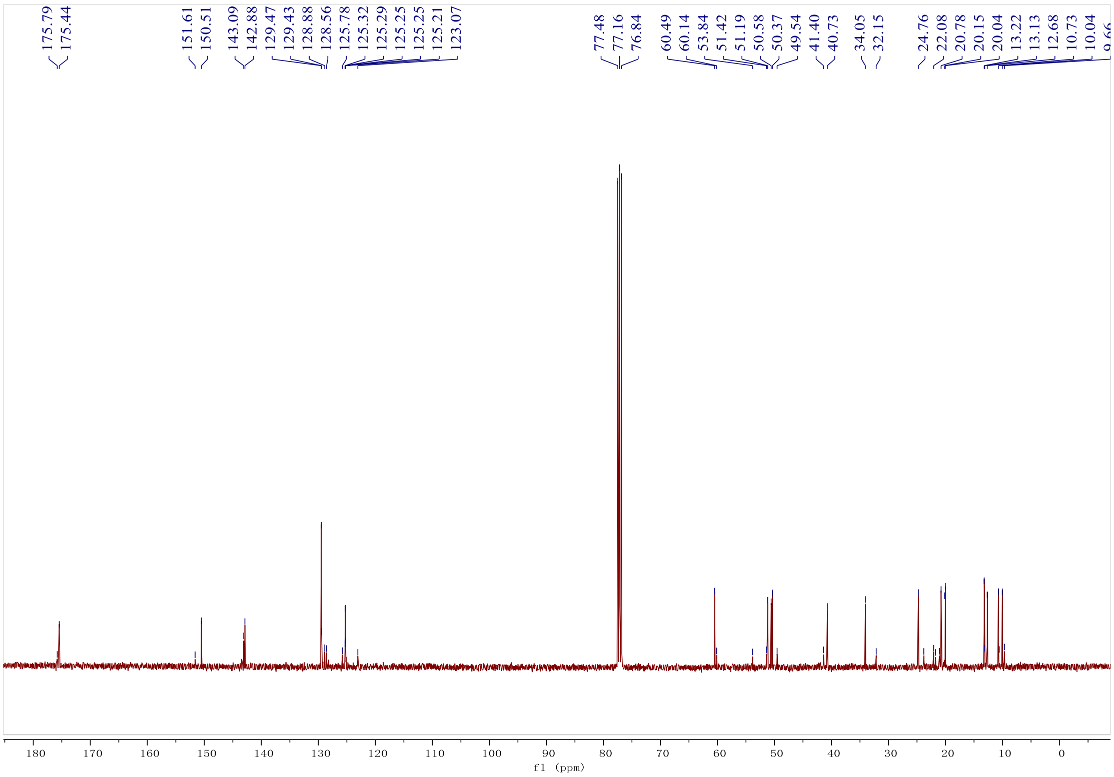

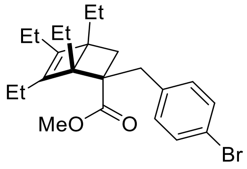
Et
Et
Et
Et
MeO
O
Br

2e

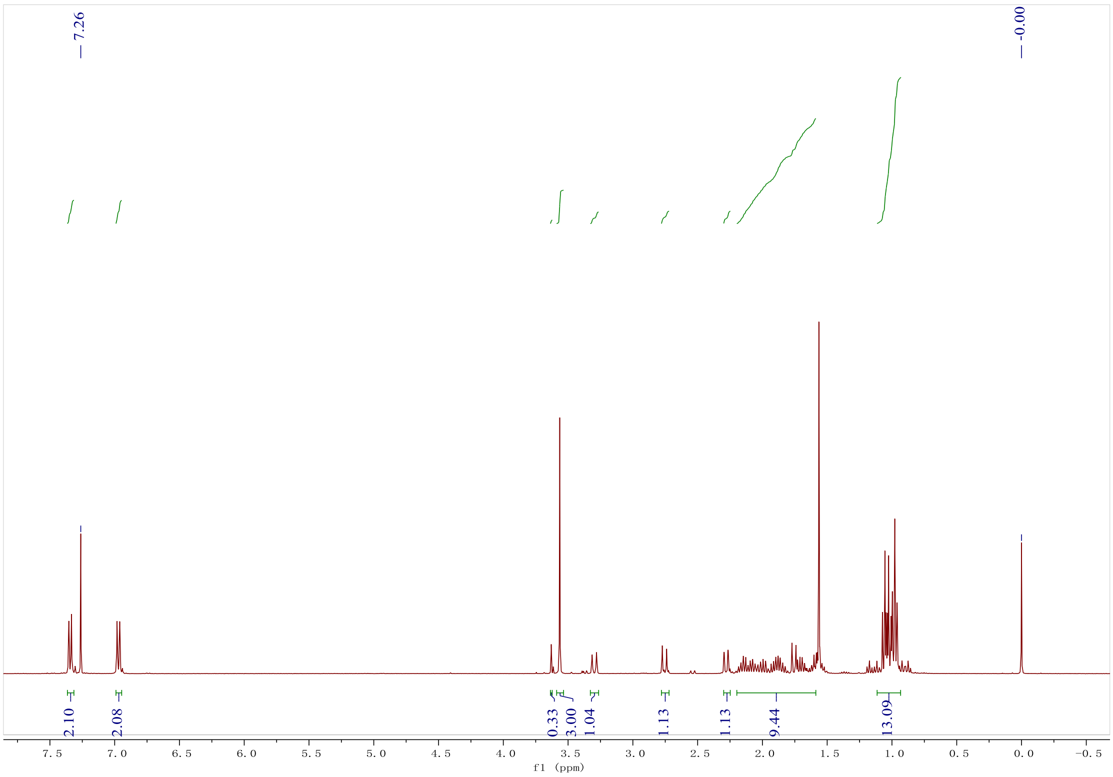
7.26
-0.00
2.10
2.08
0.33
3.00
1.04
1.13
1.13
9.44
13.09
7.5 7.0 6.5 6.0 5.5 5.0 4.5 4.0 3.5 3.0 2.5 2.0 1.5 1.0 0.5 0.0 -0.5
f1 (ppm)

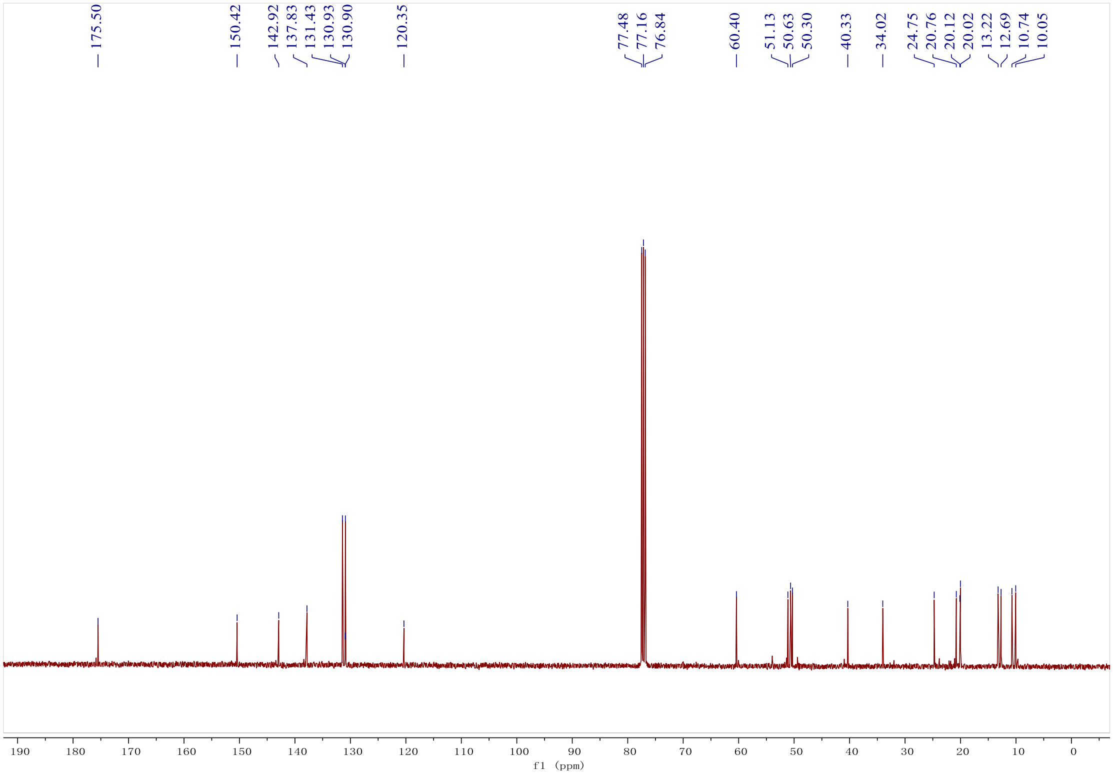
175.50
150.42
142.92
137.83
131.43
130.93
130.90
120.35
77.48
77.16
76.84
60.40
51.13
50.63
50.30
40.33
34.02
24.75
20.76
20.12
20.02
13.22
12.69
10.74
10.05
190 180 170 160 150 140 130 120 110 100 90 80 70 60 50 40 30 20 10 0
f1 (ppm)

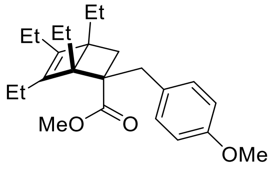
Et
Et
Et
Et
MeO
O
OMe

2f

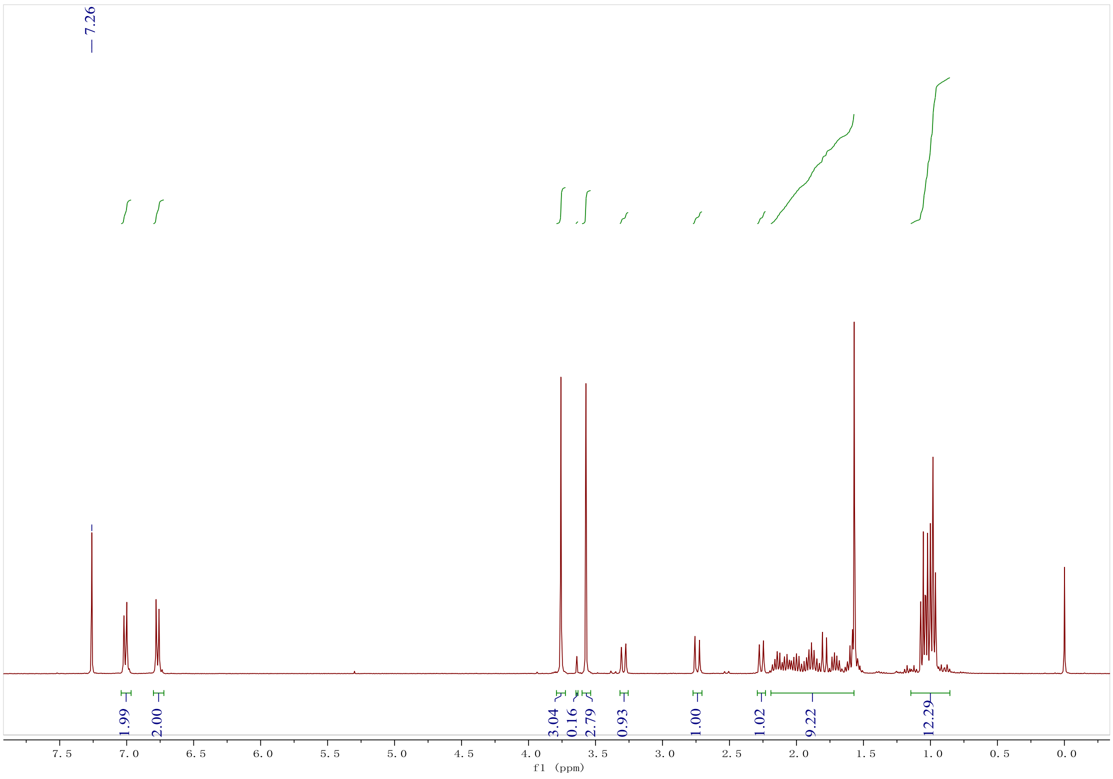
7.26
1.99
2.00
3.04
0.16
2.79
0.93
1.00
1.02
9.22
12.29
f1 (ppm)

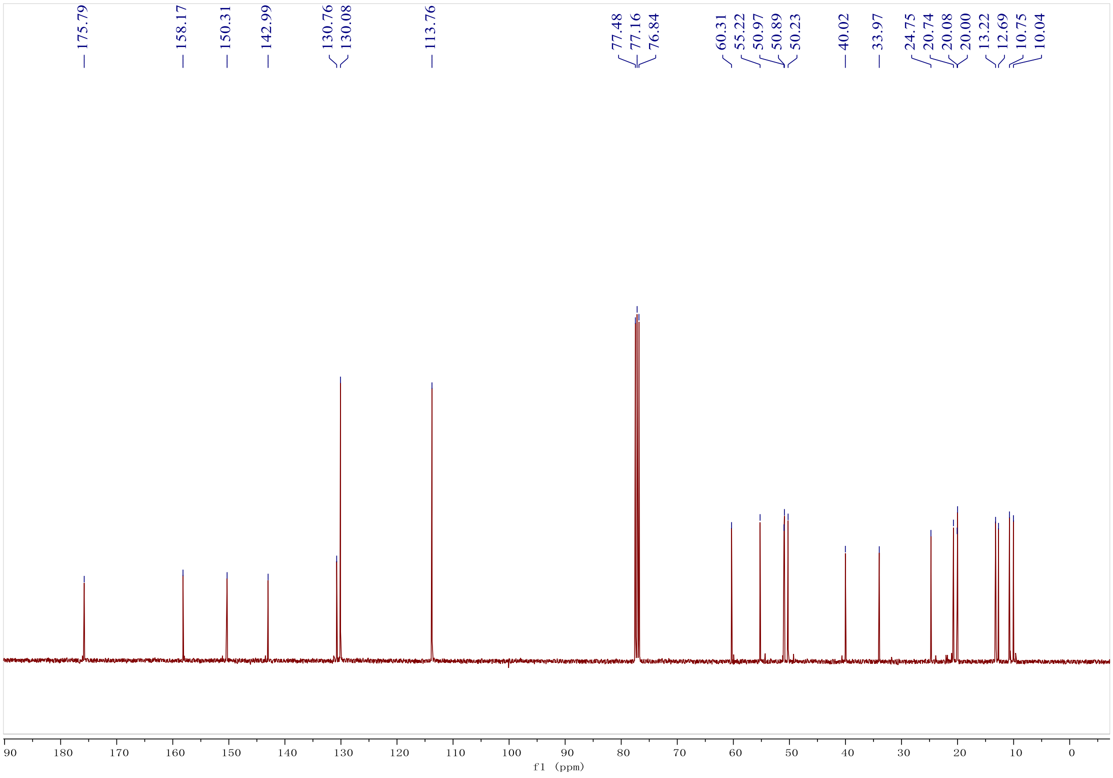
175.79
158.17
150.31
142.99
130.76
130.08
113.76
77.48
77.16
76.84
60.31
55.22
50.97
50.89
50.23
40.02
33.97
24.75
20.74
20.08
20.00
13.22
12.69
10.75
10.04
f1 (ppm)

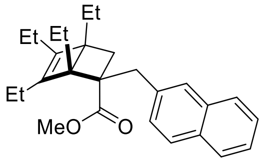
Et
Et
Et
Et
MeO
O

2g

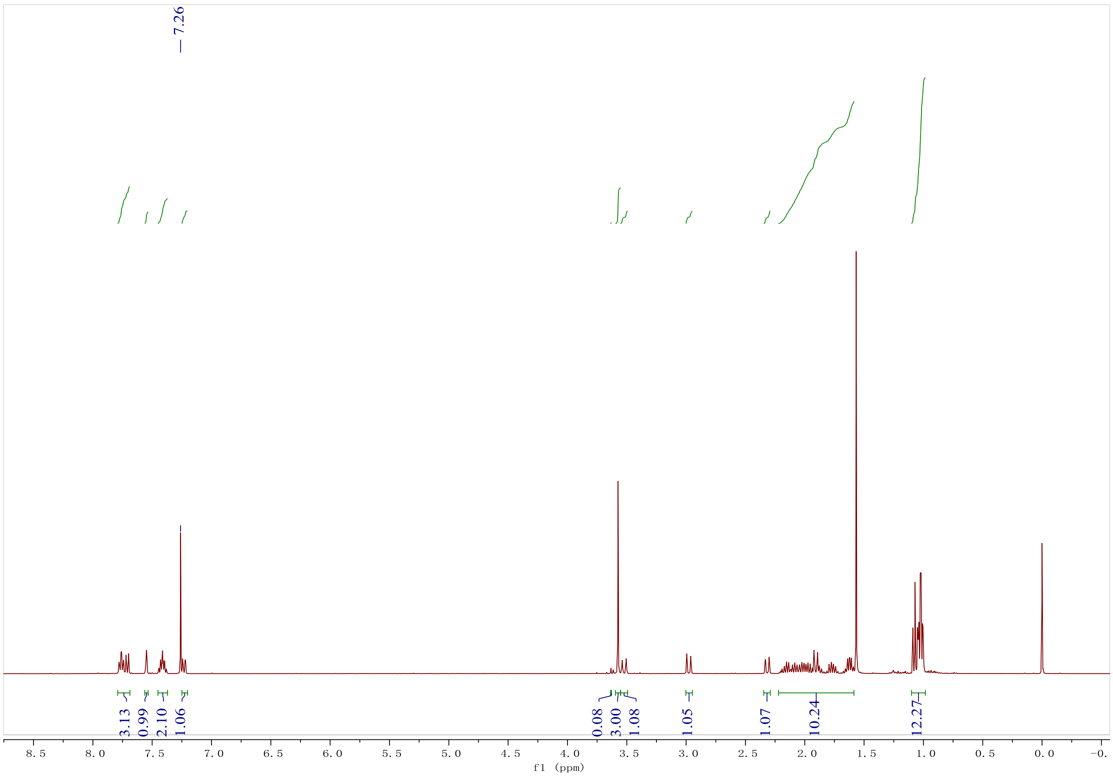
7.26
3.13
0.99
2.10
1.06
0.08
3.00
1.08
1.05
1.07
10.24
12.27
8.5 8.0 7.5 7.0 6.5 6.0 5.5 5.0 4.5 4.0 3.5 3.0 2.5 2.0 1.5 1.0 0.5 0.0
f1 (ppm)

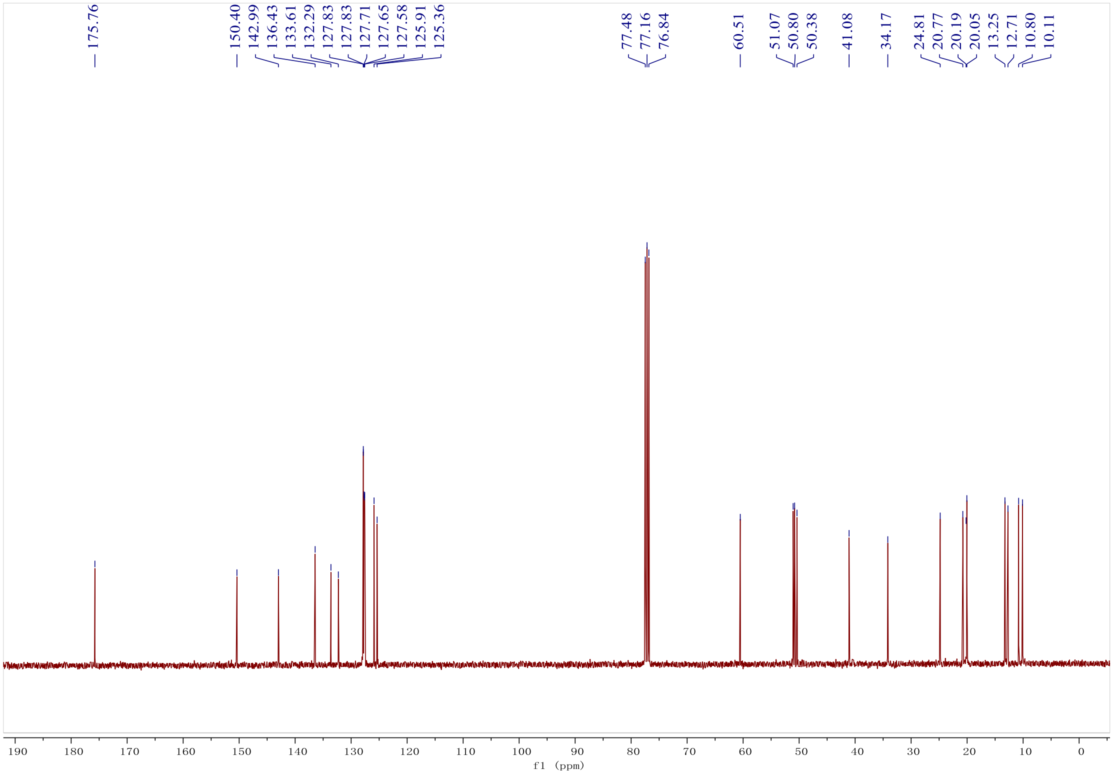
175.76
150.40
142.99
136.43
133.61
132.29
127.83
127.83
127.71
127.65
127.58
125.91
125.36
77.48
77.16
76.84
60.51
51.07
50.80
50.38
41.08
34.17
24.81
20.77
20.19
20.05
13.25
12.71
10.80
10.11
190 180 170 160 150 140 130 120 110 100 90 80 70 60 50 40 30 20 10 0
f1 (ppm)

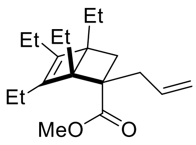
Et
Et
Et
Et
MeO
O

2h

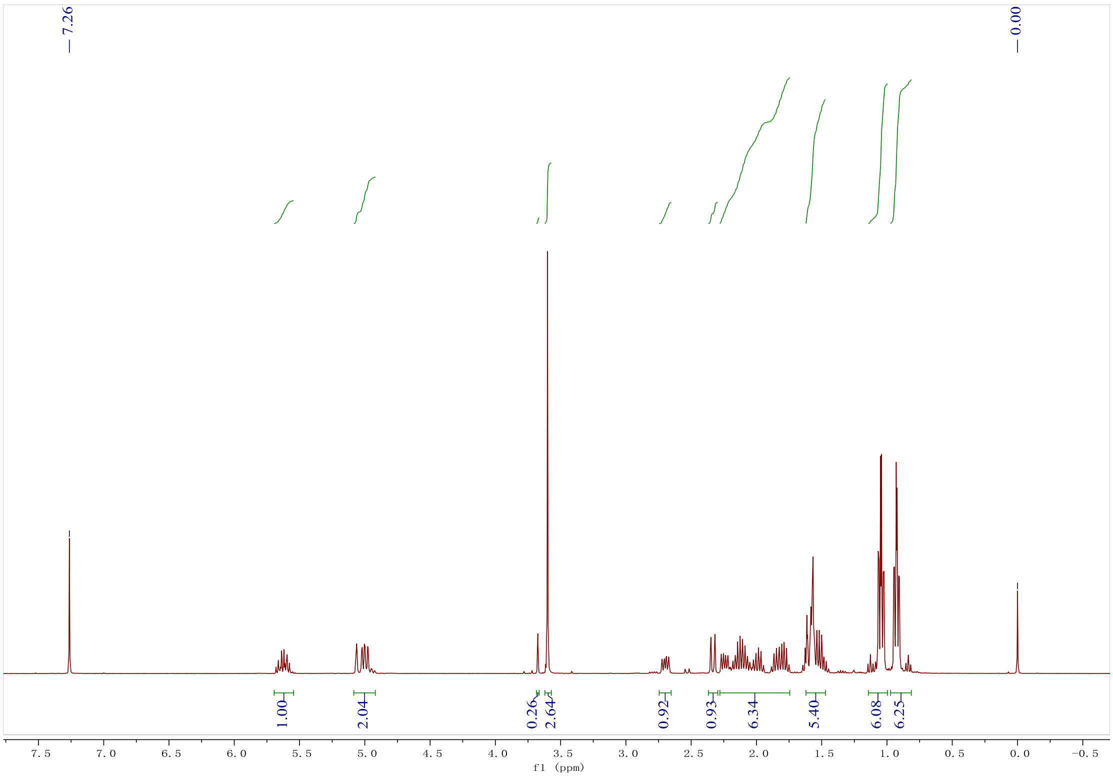
7.26
0.00
1.00
2.04
0.26
2.64
0.92
0.93
6.34
5.40
6.08
6.25
f1 (ppm)

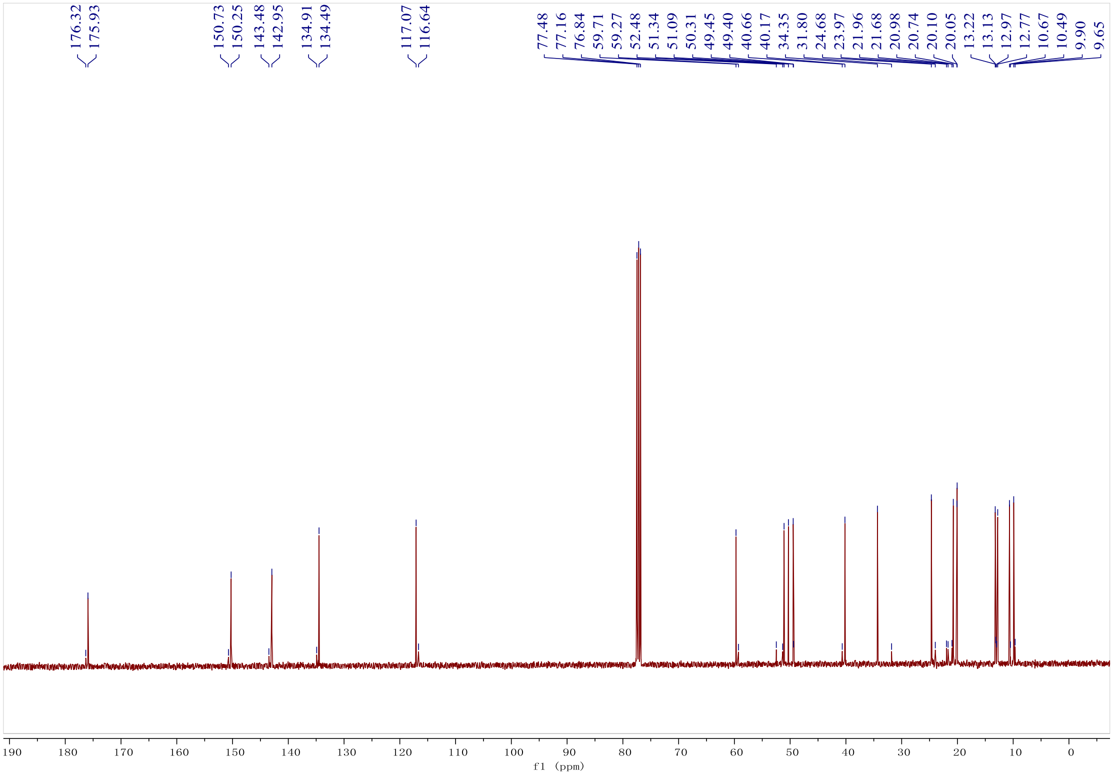
176.32
175.93
150.73
150.25
143.48
142.95
134.91
134.49
117.07
116.64
77.48
77.16
76.84
59.71
59.27
52.48
51.34
51.09
50.31
49.45
49.40
40.66
40.17
34.35
31.80
24.68
23.97
21.96
21.68
20.98
20.74
20.10
20.05
13.22
13.13
12.97
12.77
10.67
10.49
9.90
9.65
f1 (ppm)

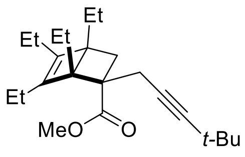
Et
Et
Et
Et
MeO
O
t-Bu

2i

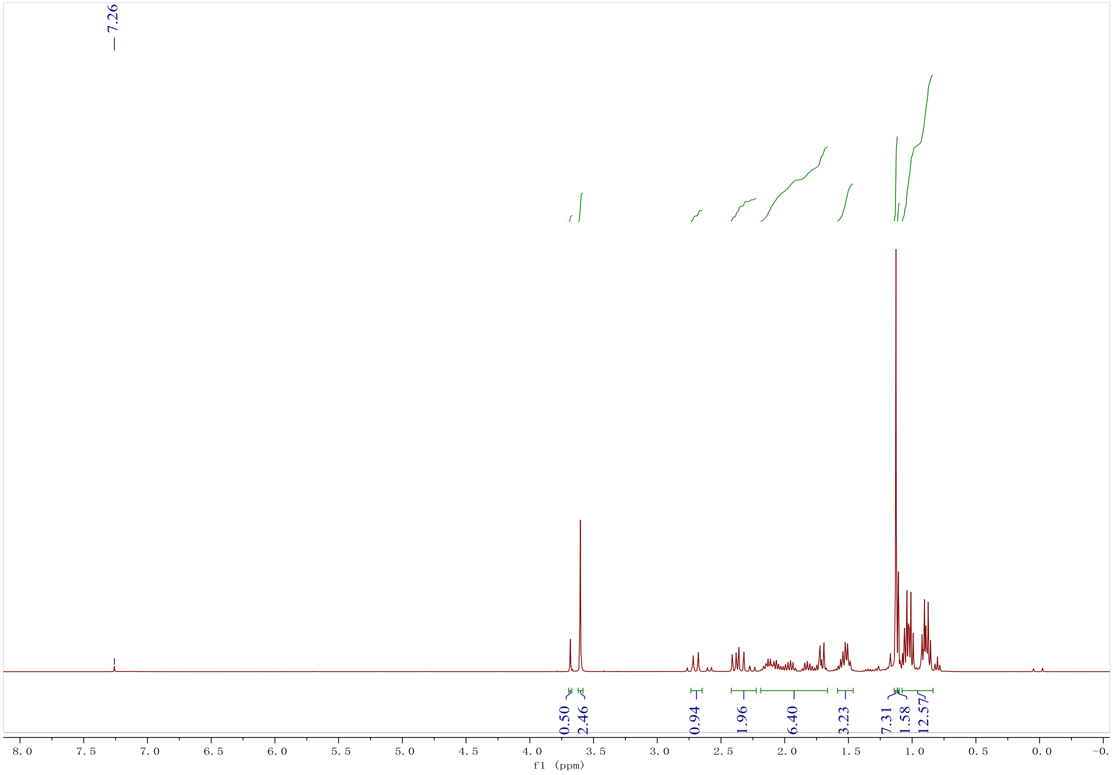
7.26
0.50
2.46
0.94
1.96
6.40
3.23
7.31
1.58
12.57
8.0
7.5
7.0
6.5
6.0
5.5
5.0
4.5
4.0
3.5
3.0
2.5
2.0
1.5
1.0
0.5
0.0
f1 (ppm)

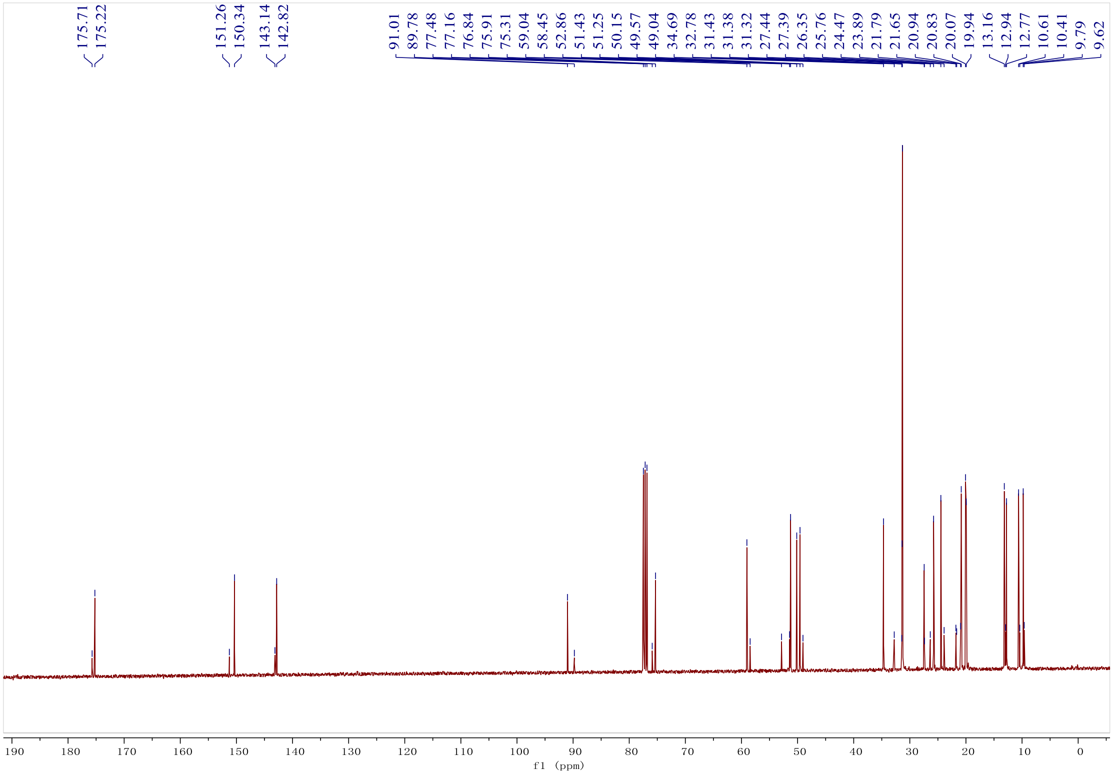
175.71
175.22
151.26
150.34
143.14
142.82
91.01
89.78
77.48
77.16
76.84
75.91
75.31
59.04
58.45
52.86
51.43
51.25
50.15
49.57
49.04
34.69
32.78
31.43
31.38
31.32
27.44
27.39
26.35
25.76
24.47
23.89
21.79
21.65
20.94
20.83
20.07
19.94
13.16
12.94
12.77
10.61
10.41
9.79
9.62
190
180
170
160
150
140
130
120
110
100
90
80
70
60
50
40
30
20
10
0
f1 (ppm)

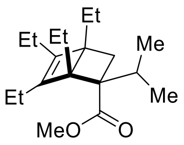
Et
Et
Et
Et
Me
Me
MeO
O

2j

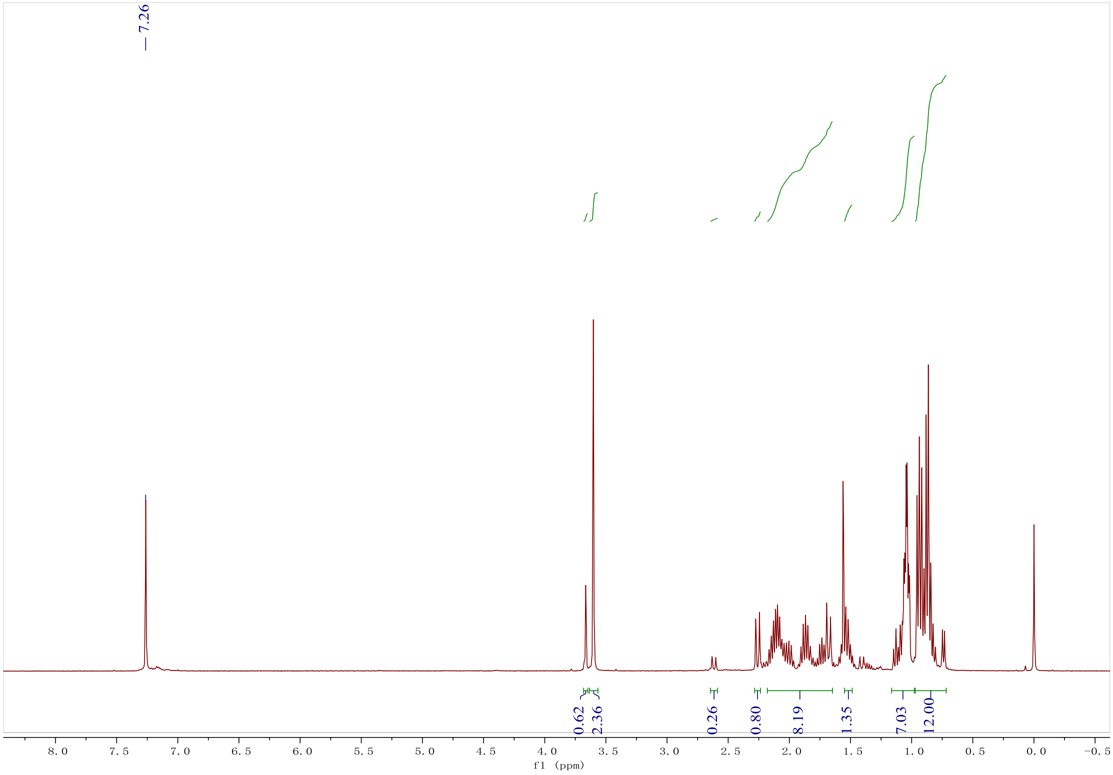
7.26
0.62
2.36
0.26
0.80
8.19
1.35
7.03
12.00
8.0 7.5 7.0 6.5 6.0 5.5 5.0 4.5 4.0 3.5 3.0 2.5 2.0 1.5 1.0 0.5 0.0 -0.5
f1 (ppm)

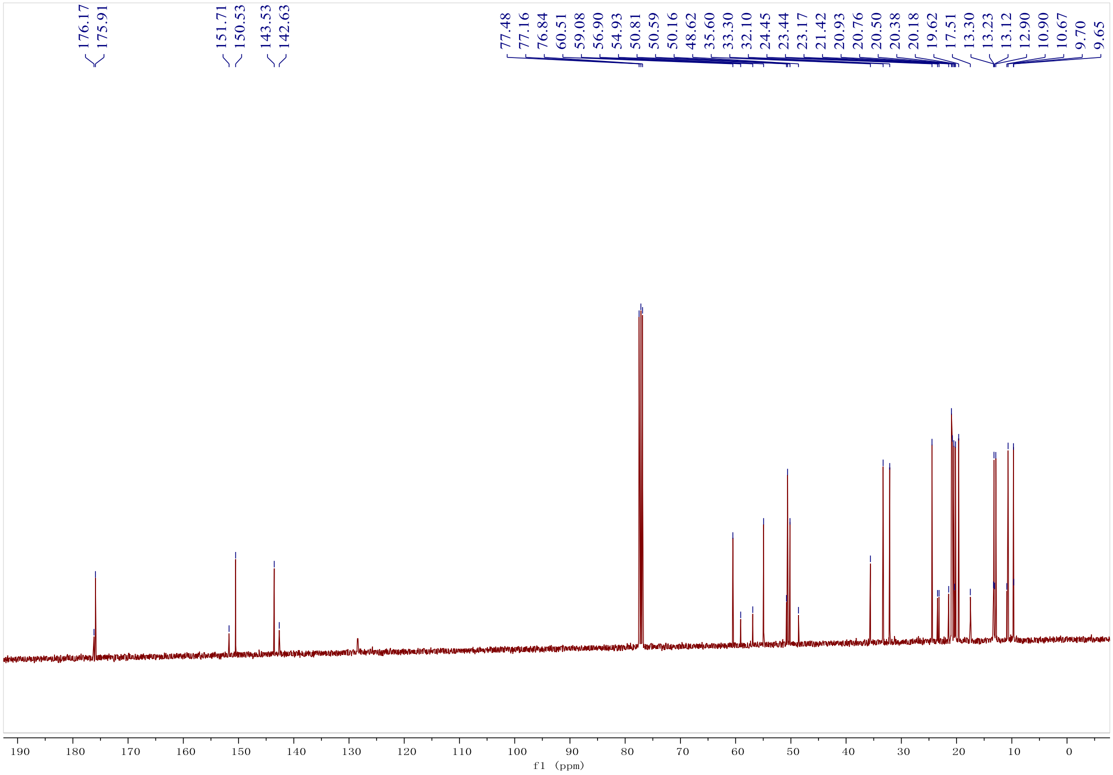
176.17
175.91
151.71
150.53
143.53
142.63
77.48
77.16
76.84
60.51
59.08
56.90
54.93
50.81
50.59
50.16
48.62
35.60
33.30
32.10
24.45
23.44
23.17
21.42
20.93
20.76
20.50
20.38
20.18
19.62
17.51
13.30
13.23
13.12
12.90
10.90
10.67
9.70
9.65
190 180 170 160 150 140 130 120 110 100 90 80 70 60 50 40 30 20 10 0
f1 (ppm)

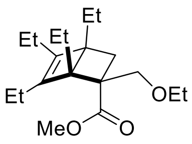
Et
Et
Et
Et
OEt
MeO
O

2k

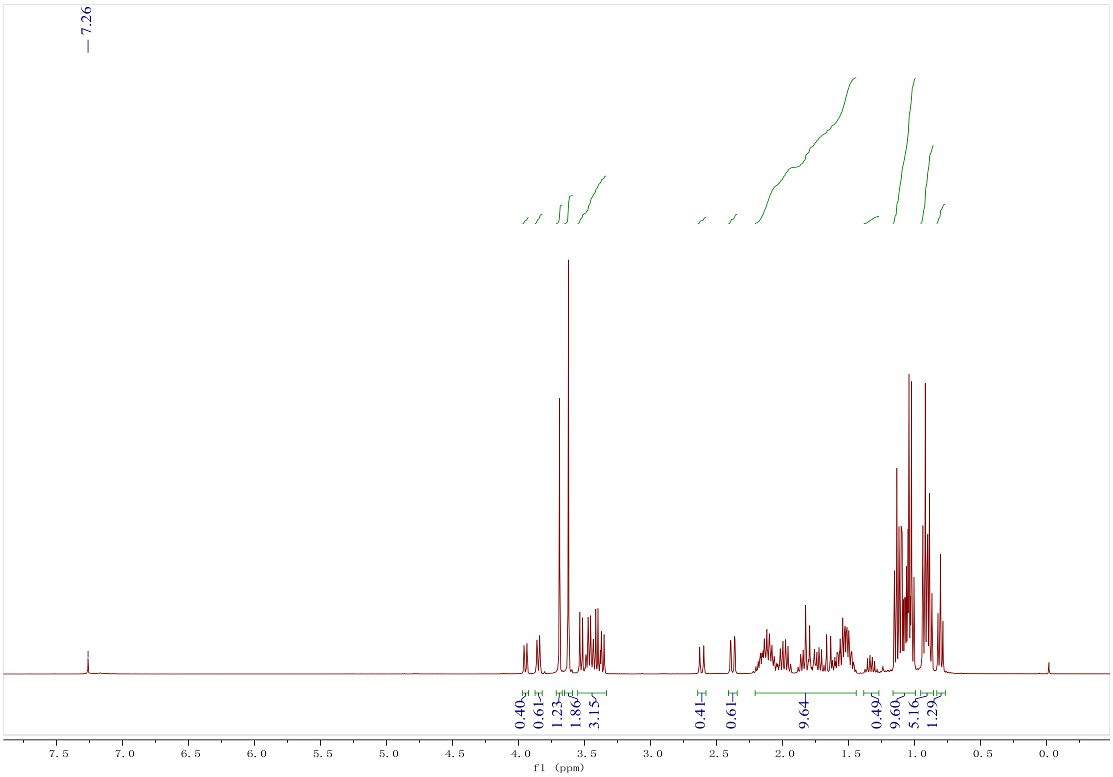
7.26
0.40
0.61
1.23
1.86
3.15
0.41
0.61
9.64
0.49
9.60
5.16
1.29
7.5 7.0 6.5 6.0 5.5 5.0 4.5 4.0 3.5 3.0 2.5 2.0 1.5 1.0 0.5 0.0
f1 (ppm)

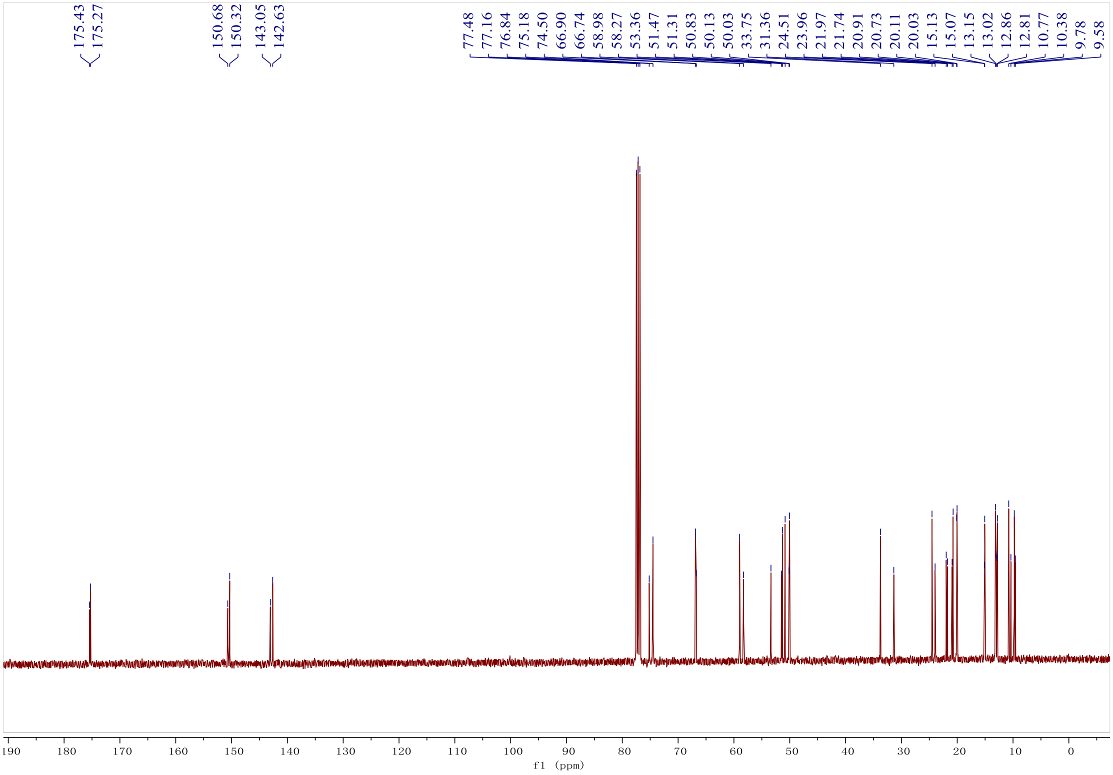
175.43
175.27
150.68
150.32
143.05
142.63
77.48
77.16
76.84
75.18
74.50
66.90
66.74
58.98
58.27
53.36
51.47
51.31
50.83
50.13
50.03
33.75
31.36
24.51
23.96
21.97
21.74
20.91
20.73
20.11
20.03
15.13
15.07
13.15
13.02
12.86
12.81
10.77
10.38
9.78
9.58
190 180 170 160 150 140 130 120 110 100 90 80 70 60 50 40 30 20 10 0
f1 (ppm)

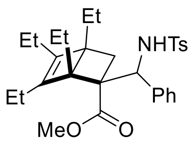
Et
Et
Et
NHTs
Ph
Et
MeO
O

2l

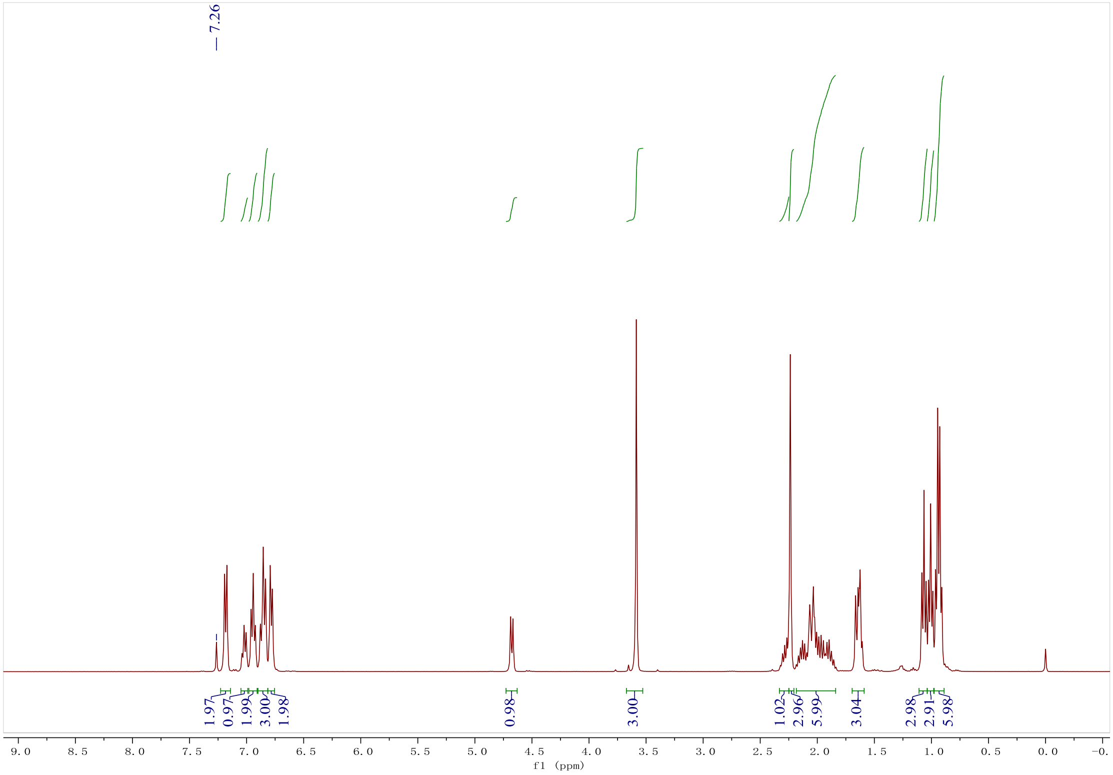
7.26
1.97
0.97
1.99
3.00
1.98
0.98
3.00
1.02
2.96
5.99
3.04
2.98
2.91
5.98
9.0 8.5 8.0 7.5 7.0 6.5 6.0 5.5 5.0 4.5 4.0 3.5 3.0 2.5 2.0 1.5 1.0 0.5 0.0
f1 (ppm)

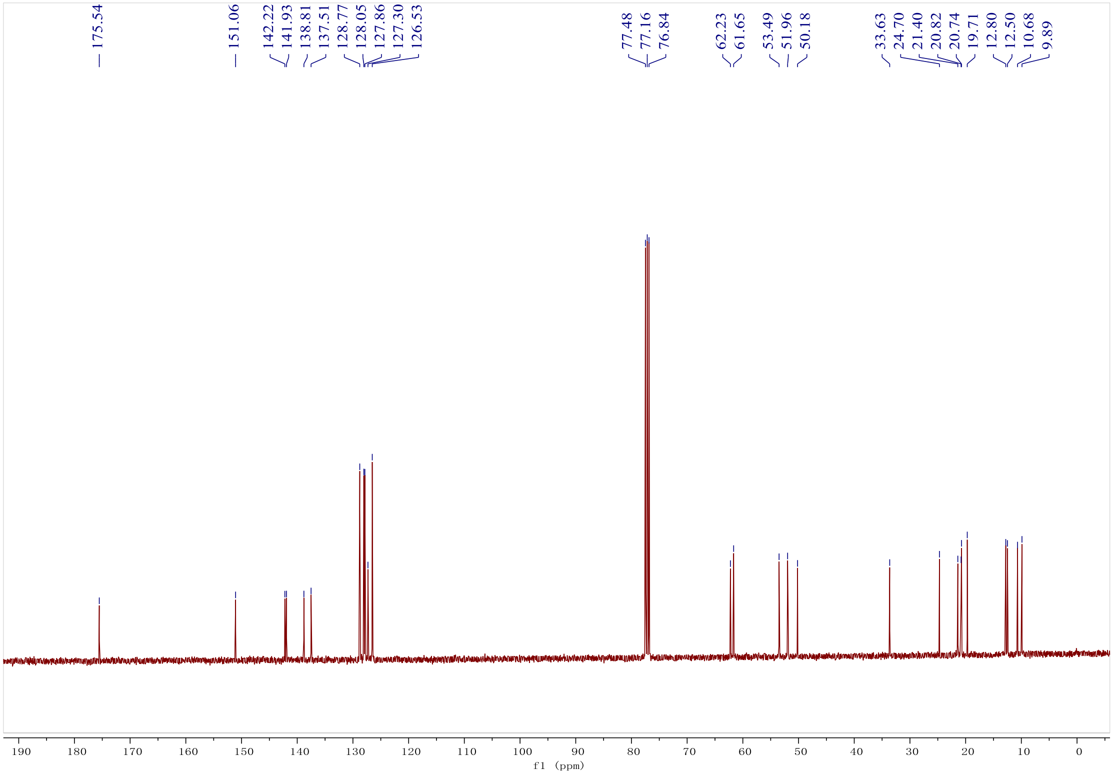
175.54
151.06
142.22
141.93
138.81
137.51
128.77
128.05
127.86
127.30
126.53
77.48
77.16
76.84
62.23
61.65
53.49
51.96
50.18
33.63
24.70
21.40
20.82
20.74
19.71
12.80
12.50
10.68
9.89
190 180 170 160 150 140 130 120 110 100 90 80 70 60 50 40 30 20 10 0
f1 (ppm)

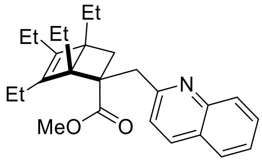

2m

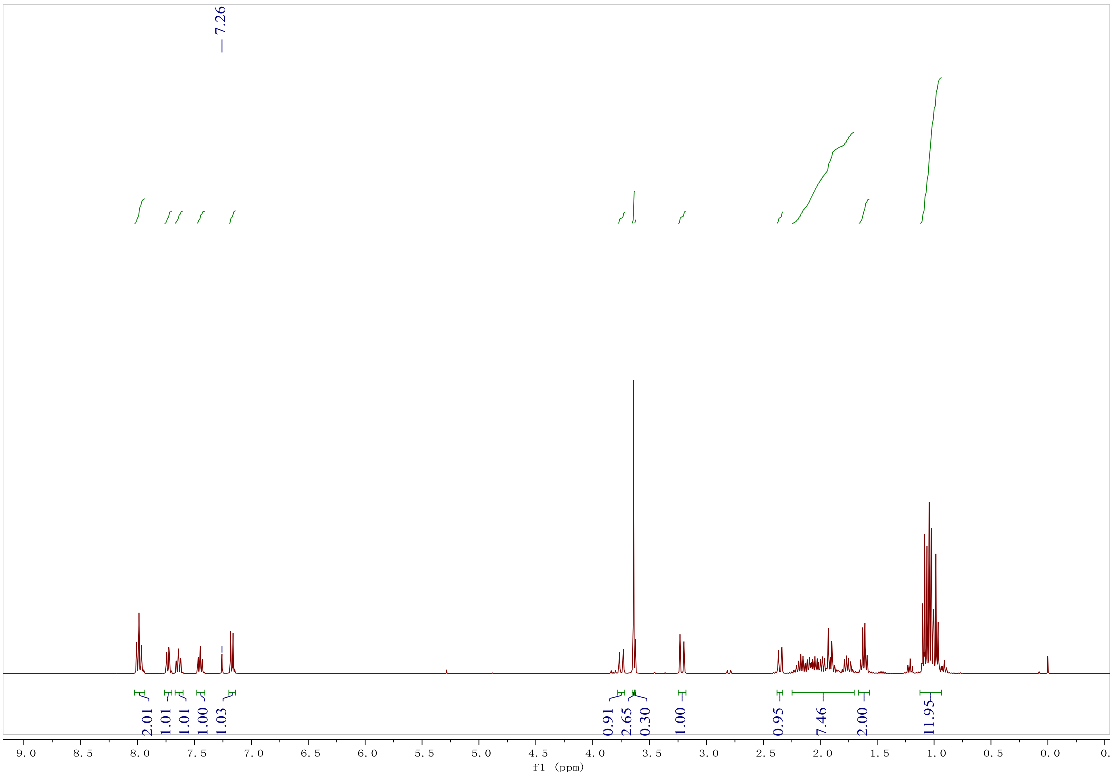
7.26
2.01
1.01
1.01
1.00
1.03
0.91
2.65
0.30
1.00
0.95
7.46
2.00
11.95
f1 (ppm)

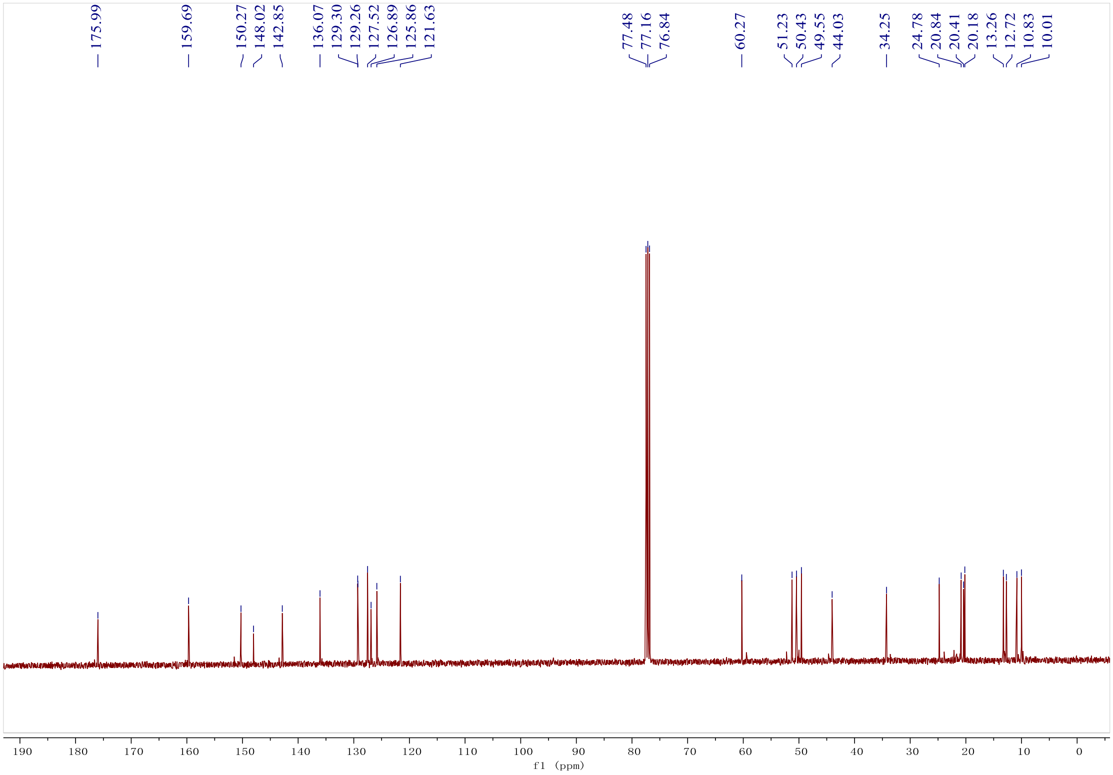
175.99
159.69
150.27
148.02
142.85
136.07
129.30
129.26
127.52
126.89
125.86
121.63
77.48
77.16
76.84
60.27
51.23
50.43
49.55
44.03
34.25
24.78
20.84
20.41
20.18
13.26
12.72
10.83
10.01
f1 (ppm)

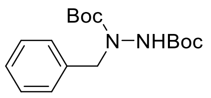
Boc
N–NHBoc

4a

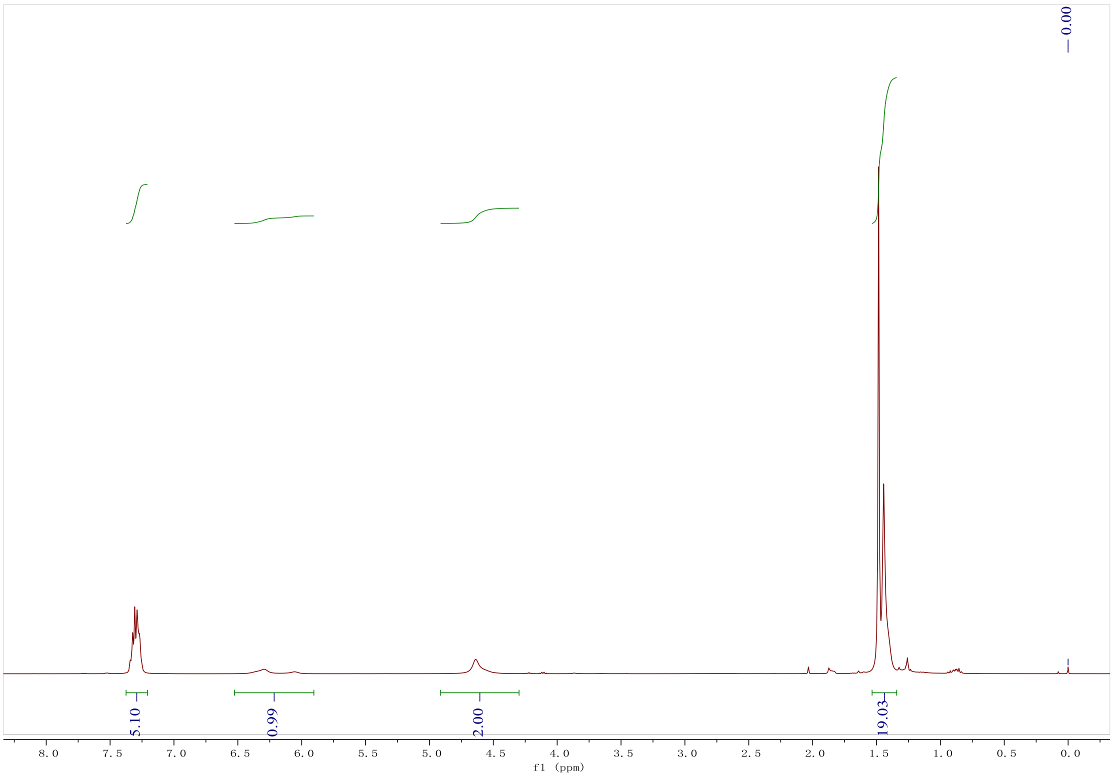
0.00
5.10
0.99
2.00
19.03
8.0
7.5
7.0
6.5
6.0
5.5
5.0
4.5
4.0
3.5
3.0
2.5
2.0
1.5
1.0
0.5
0.0
f1 (ppm)

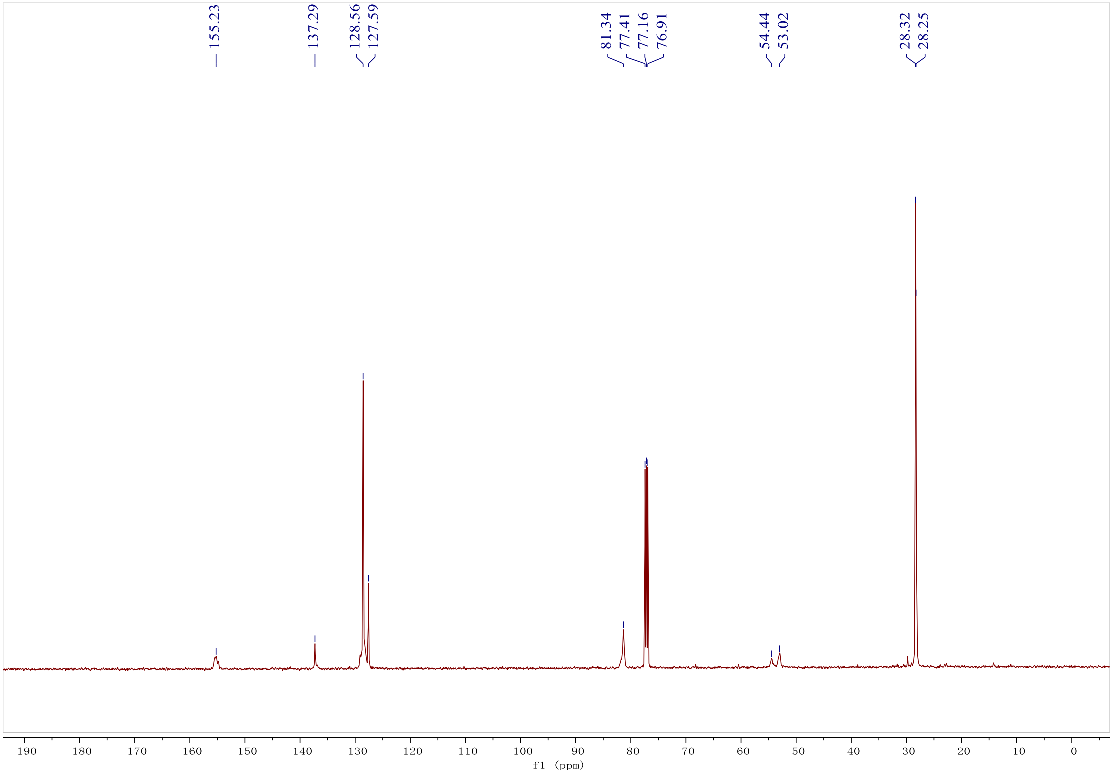
155.23
137.29
128.56
127.59
81.34
77.41
77.16
76.91
54.44
53.02
28.32
28.25
190
180
170
160
150
140
130
120
110
100
90
80
70
60
50
40
30
20
10
0
f1 (ppm)

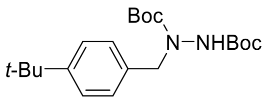
Boc
N−NHBoc
t-Bu

4c

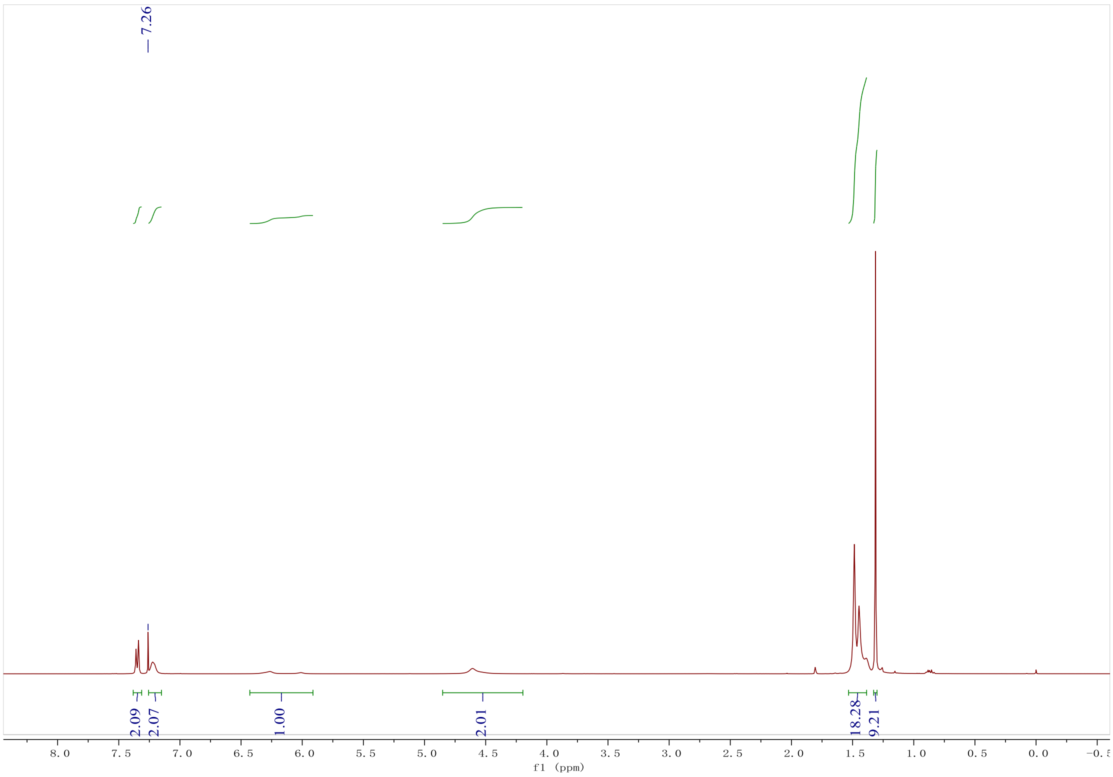
7.26
2.09
2.07
1.00
2.01
18.28
9.21
8.0 7.5 7.0 6.5 6.0 5.5 5.0 4.5 4.0 3.5 3.0 2.5 2.0 1.5 1.0 0.5 0.0
f1 (ppm)

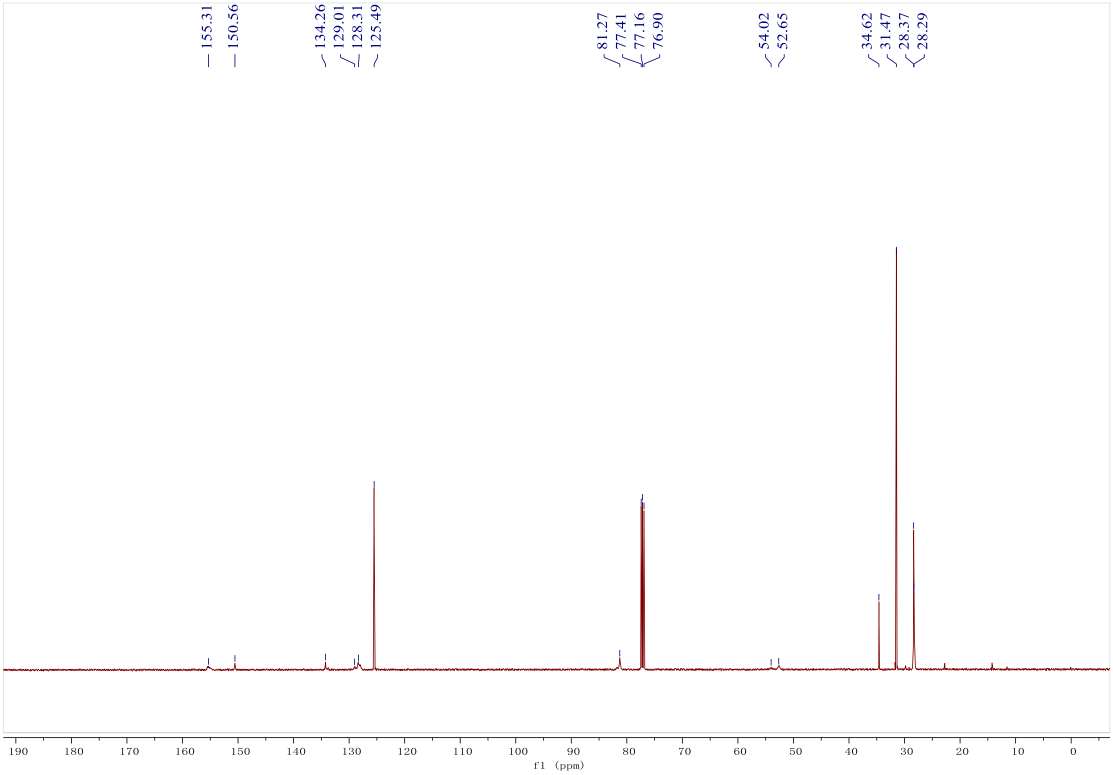
155.31
150.56
134.26
129.01
128.31
125.49
81.27
77.41
77.16
76.90
54.02
52.65
34.62
31.47
28.37
28.29
190 180 170 160 150 140 130 120 110 100 90 80 70 60 50 40 30 20 10 0
f1 (ppm)

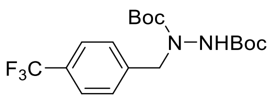
Boc
N–NHBoc
F3C

4d

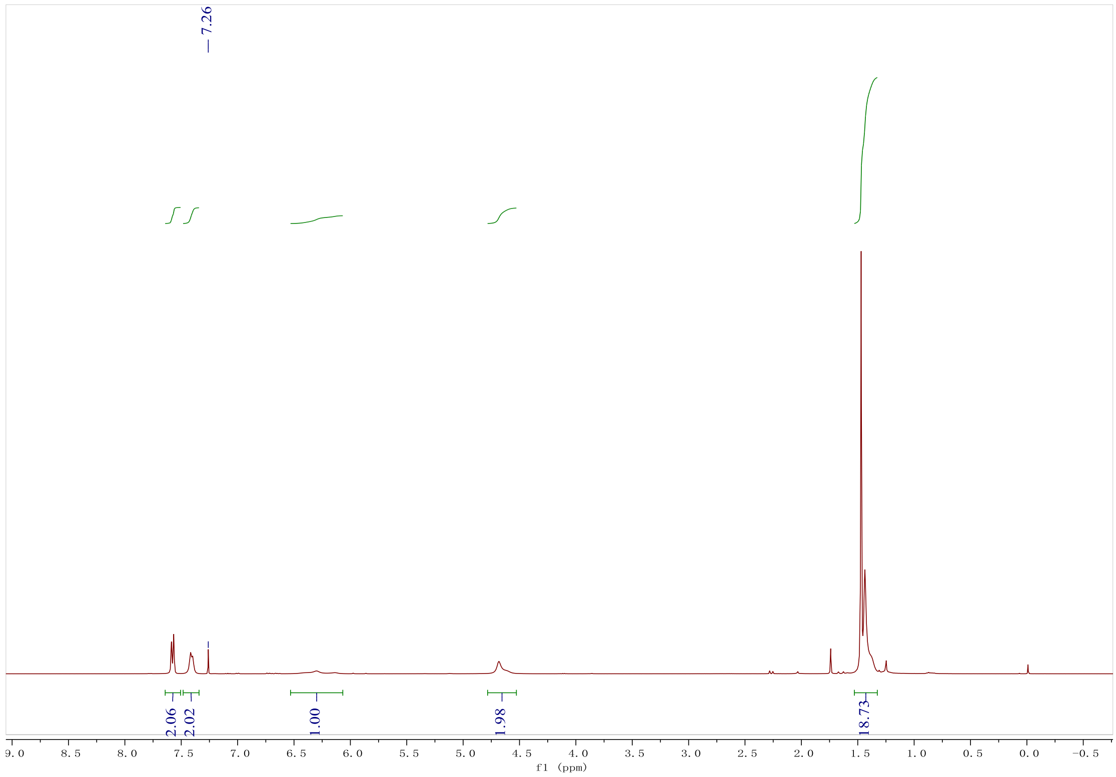
7.26
2.06
2.02
1.00
1.98
18.73
9.0 8.5 8.0 7.5 7.0 6.5 6.0 5.5 5.0 4.5 4.0 3.5 3.0 2.5 2.0 1.5 1.0 0.5 0.0 -0.5
f1 (ppm)

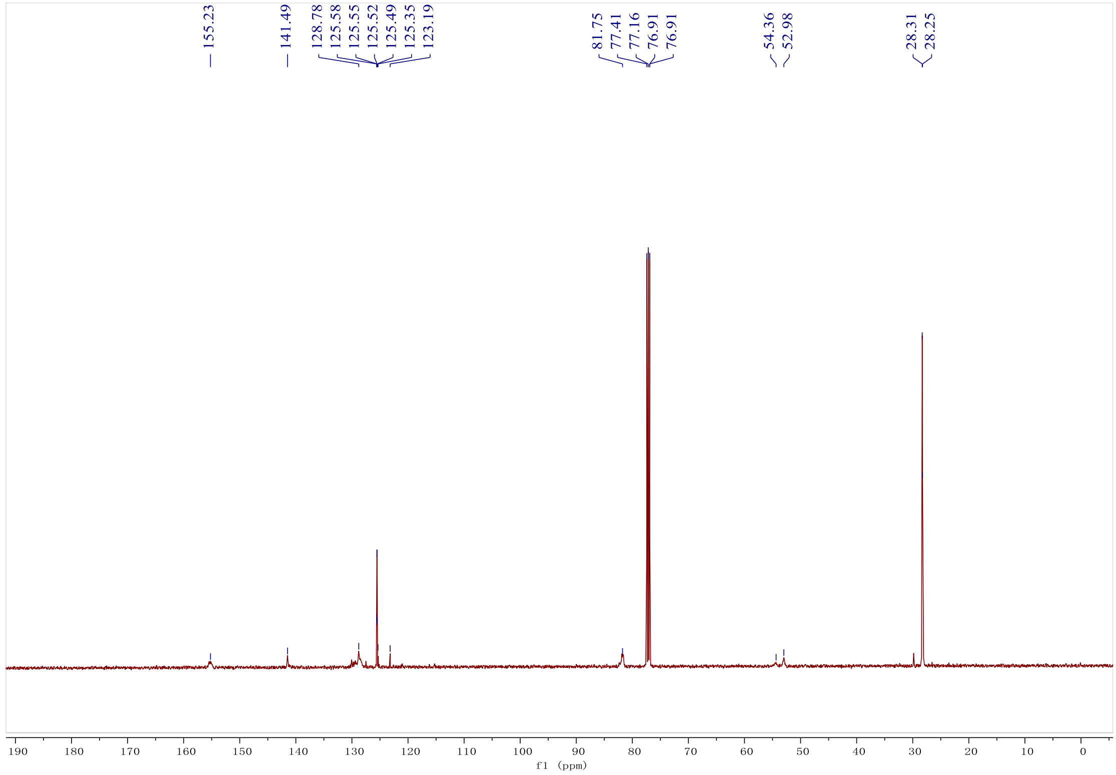
155.23
141.49
128.78
125.58
125.55
125.52
125.49
125.35
123.19
81.75
77.41
77.16
76.91
76.91
54.36
52.98
28.31
28.25
190 180 170 160 150 140 130 120 110 100 90 80 70 60 50 40 30 20 10 0
f1 (ppm)

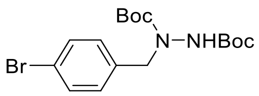
Boc
N–NHBoc
Br

4e

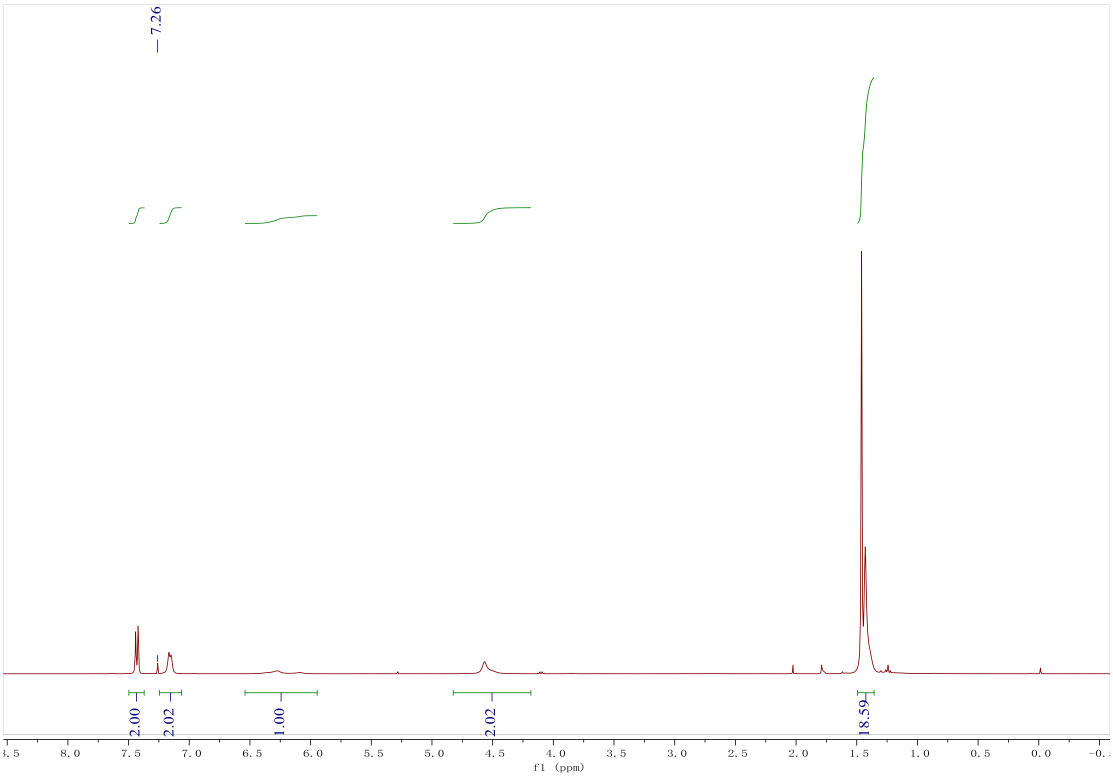
7.26
2.00
2.02
1.00
2.02
18.59
f1 (ppm)

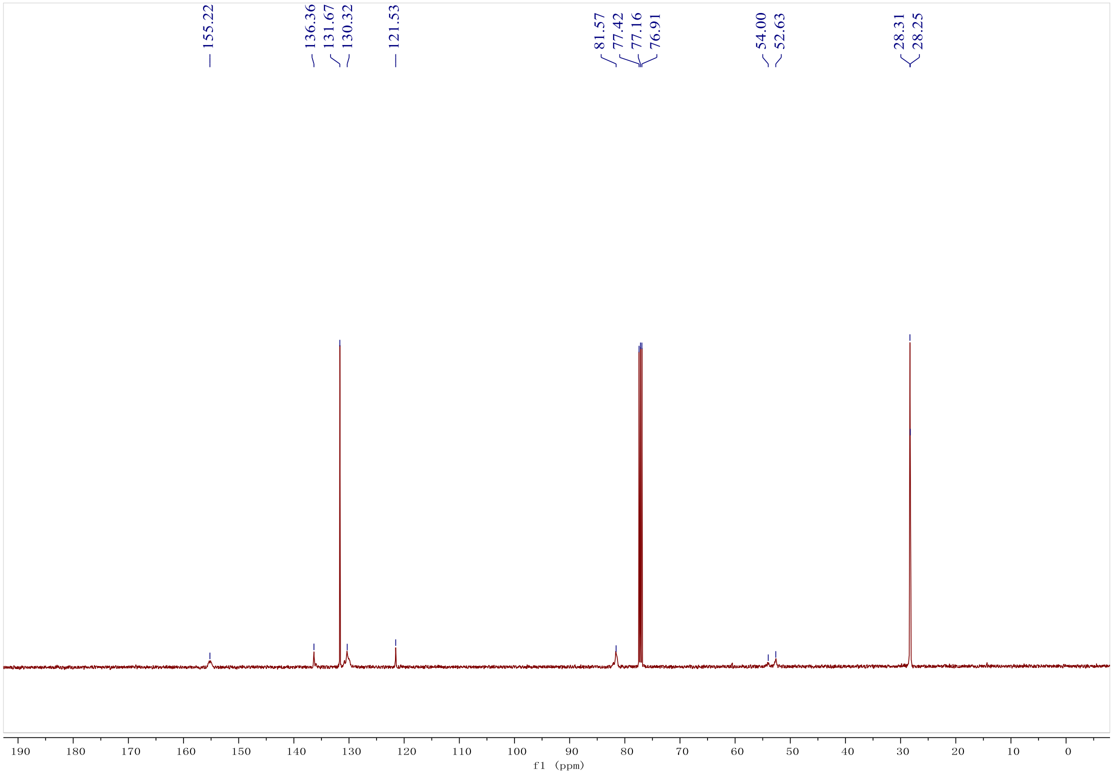
155.22
136.36
131.67
130.32
121.53
81.57
77.42
77.16
76.91
54.00
52.63
28.31
28.25
f1 (ppm)

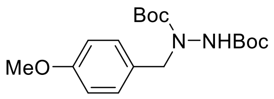
Boc
N−NHBoc
MeO

4f

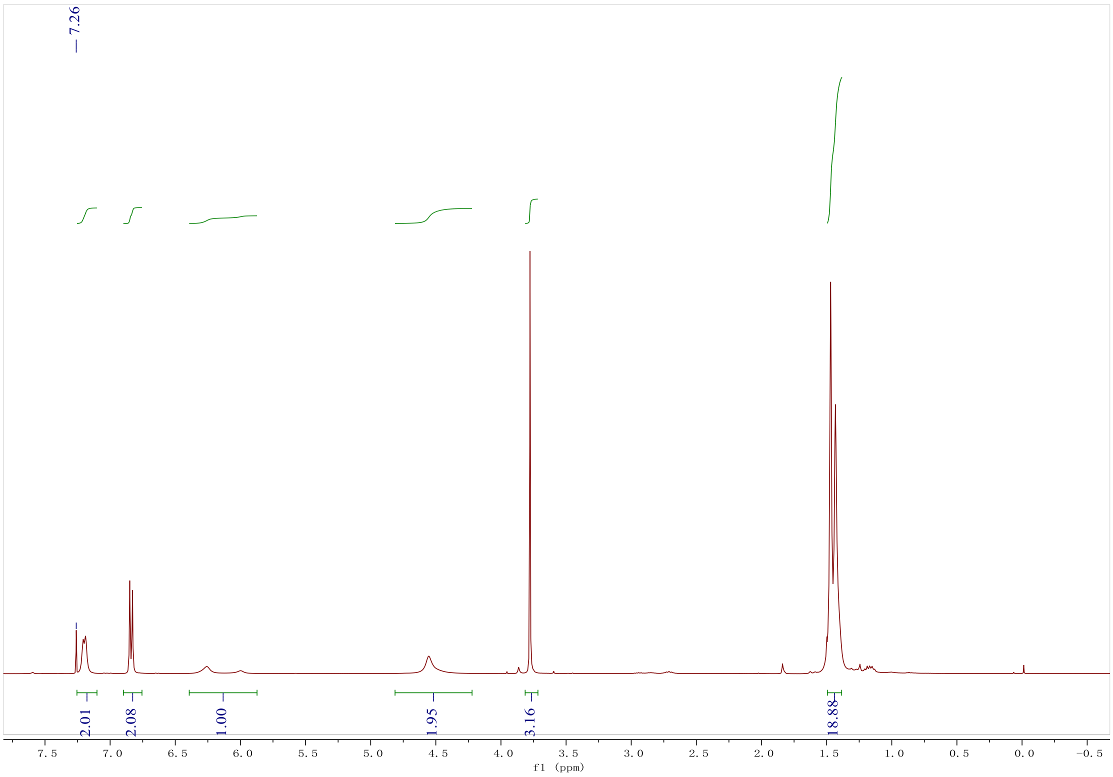
7.26
2.01
2.08
1.00
1.95
3.16
18.88
7.5 7.0 6.5 6.0 5.5 5.0 4.5 4.0 3.5 3.0 2.5 2.0 1.5 1.0 0.5 0.0 -0.5
f1 (ppm)

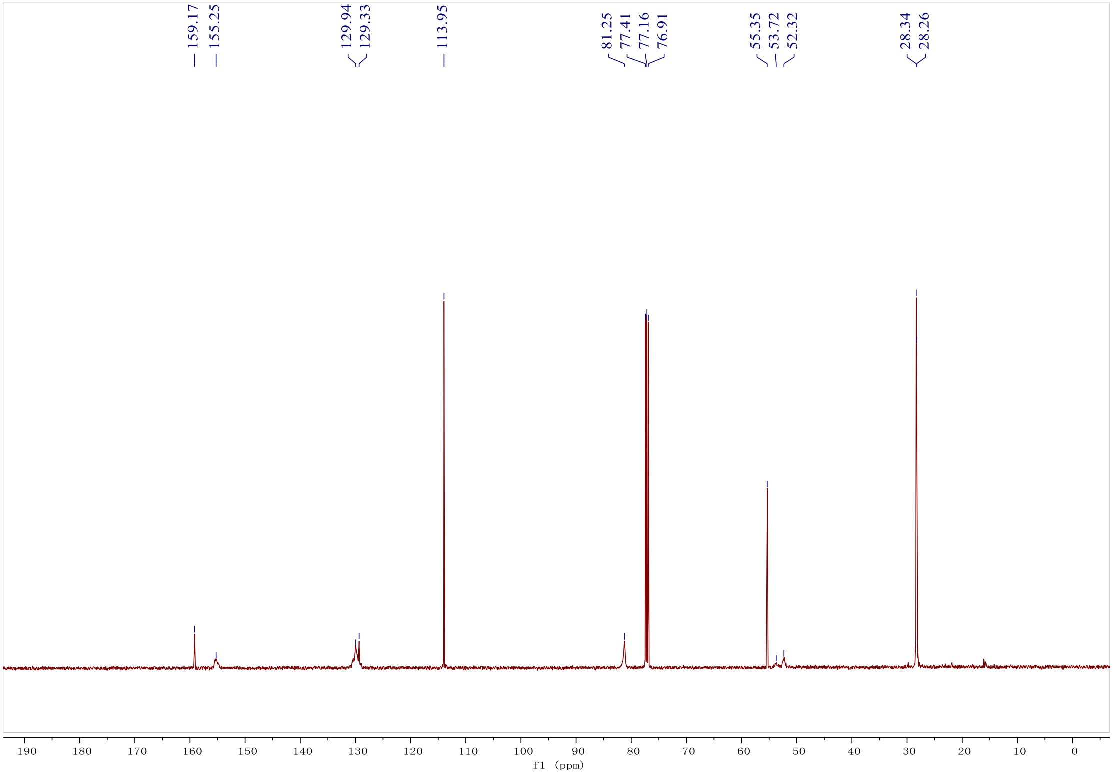
159.17
155.25
129.94
129.33
113.95
81.25
77.41
77.16
76.91
55.35
53.72
52.32
28.34
28.26
190 180 170 160 150 140 130 120 110 100 90 80 70 60 50 40 30 20 10 0
f1 (ppm)

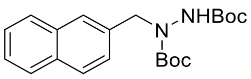
NHBoc
N
Boc

4g

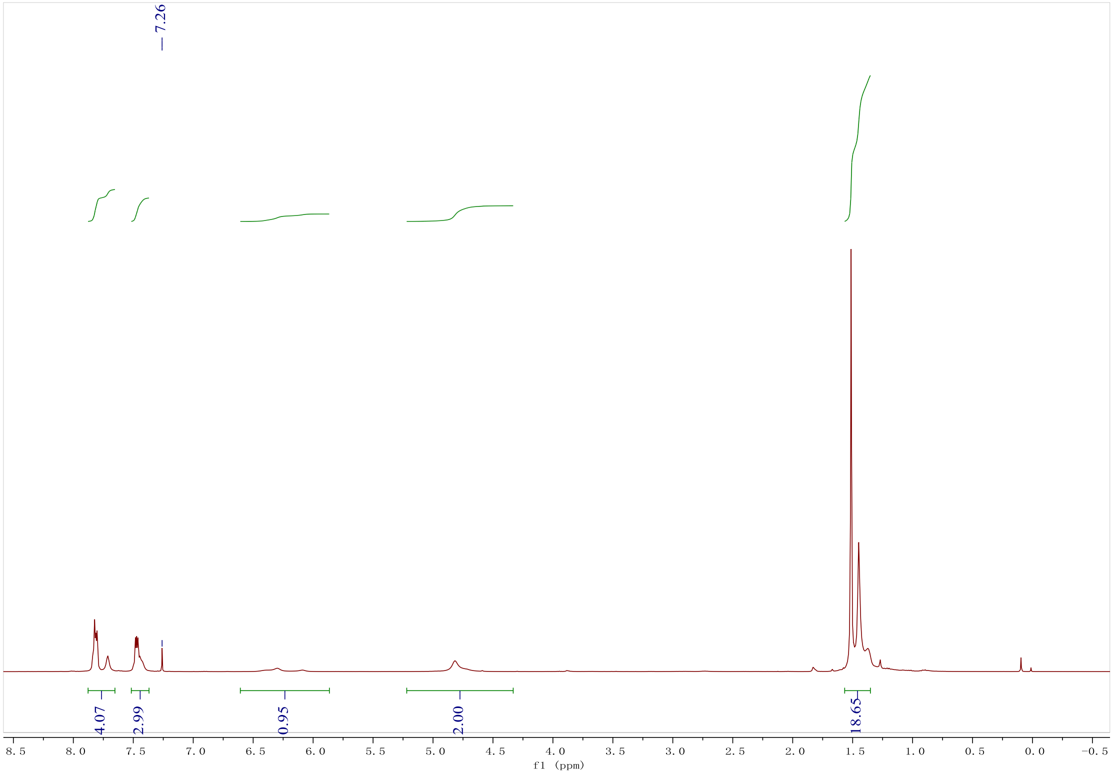
7.26
4.07
2.99
0.95
2.00
18.65
8.5
8.0
7.5
7.0
6.5
6.0
5.5
5.0
4.5
4.0
3.5
3.0
2.5
2.0
1.5
1.0
0.5
0.0
-0.5
f1 (ppm)

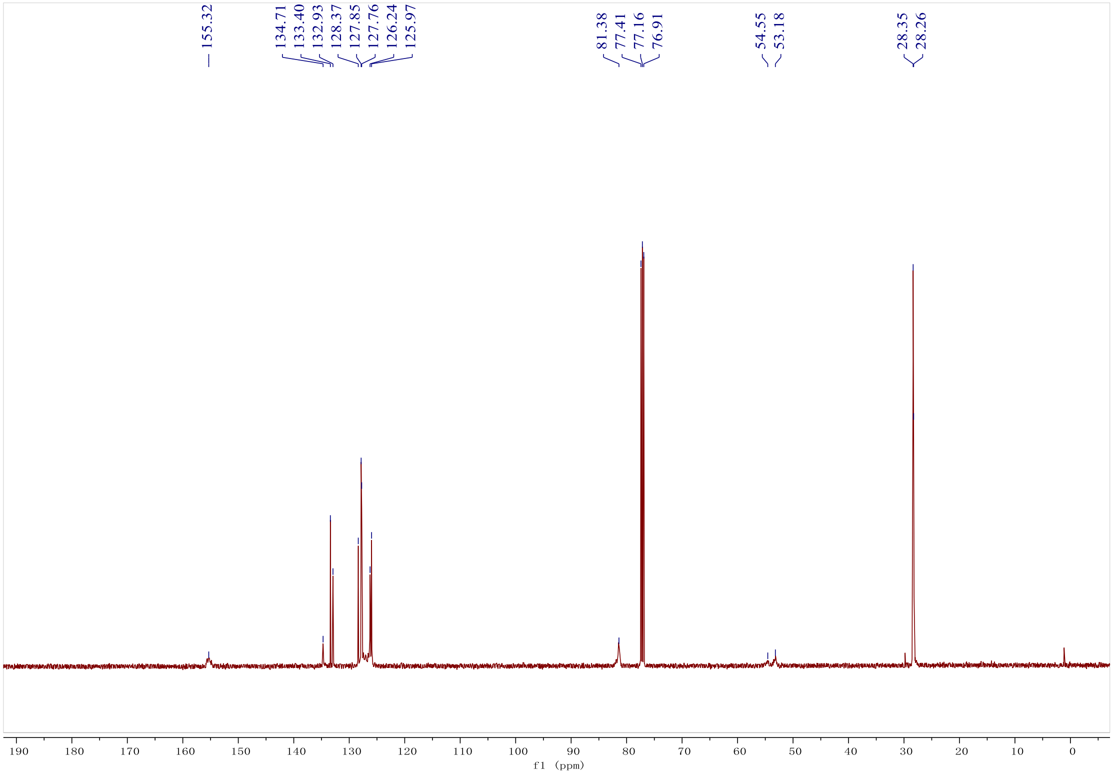
155.32
134.71
133.40
132.93
128.37
127.85
127.76
126.24
125.97
81.38
77.41
77.16
76.91
54.55
53.18
28.35
28.26
190
180
170
160
150
140
130
120
110
100
90
80
70
60
50
40
30
20
10
0
f1 (ppm)

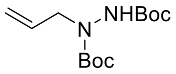
NHBoc
N
Boc

4h

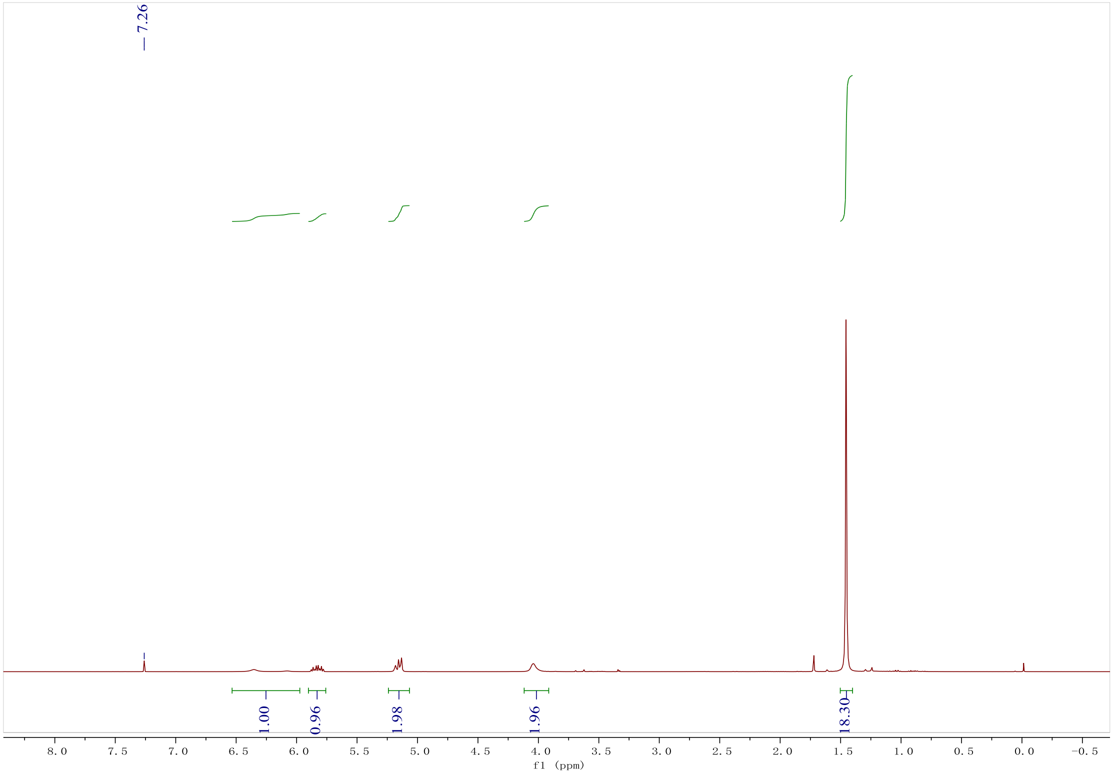
7.26
1.00
0.96
1.98
1.96
18.30
8.0 7.5 7.0 6.5 6.0 5.5 5.0 4.5 4.0 3.5 3.0 2.5 2.0 1.5 1.0 0.5 0.0 -0.5
f1 (ppm)

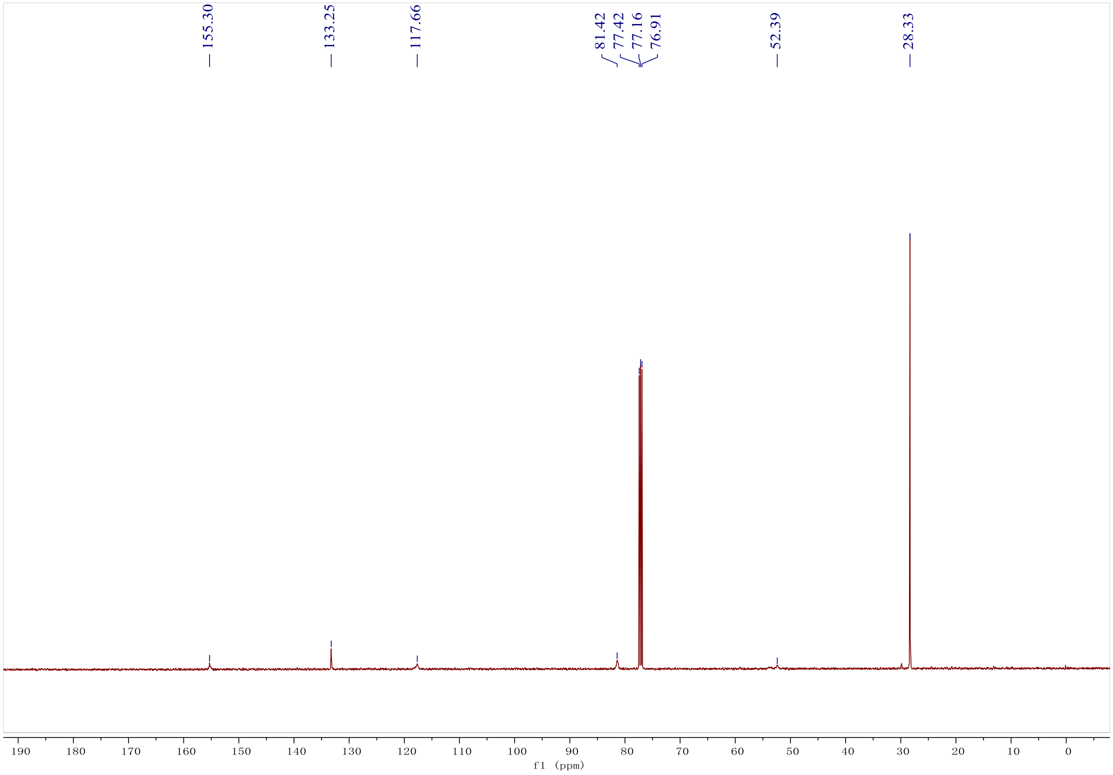
155.30
133.25
117.66
81.42
77.42
77.16
76.91
52.39
28.33
190 180 170 160 150 140 130 120 110 100 90 80 70 60 50 40 30 20 10 0
f1 (ppm)

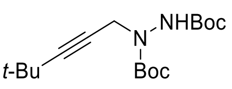
NHBoc
N
t-Bu
Boc

4i

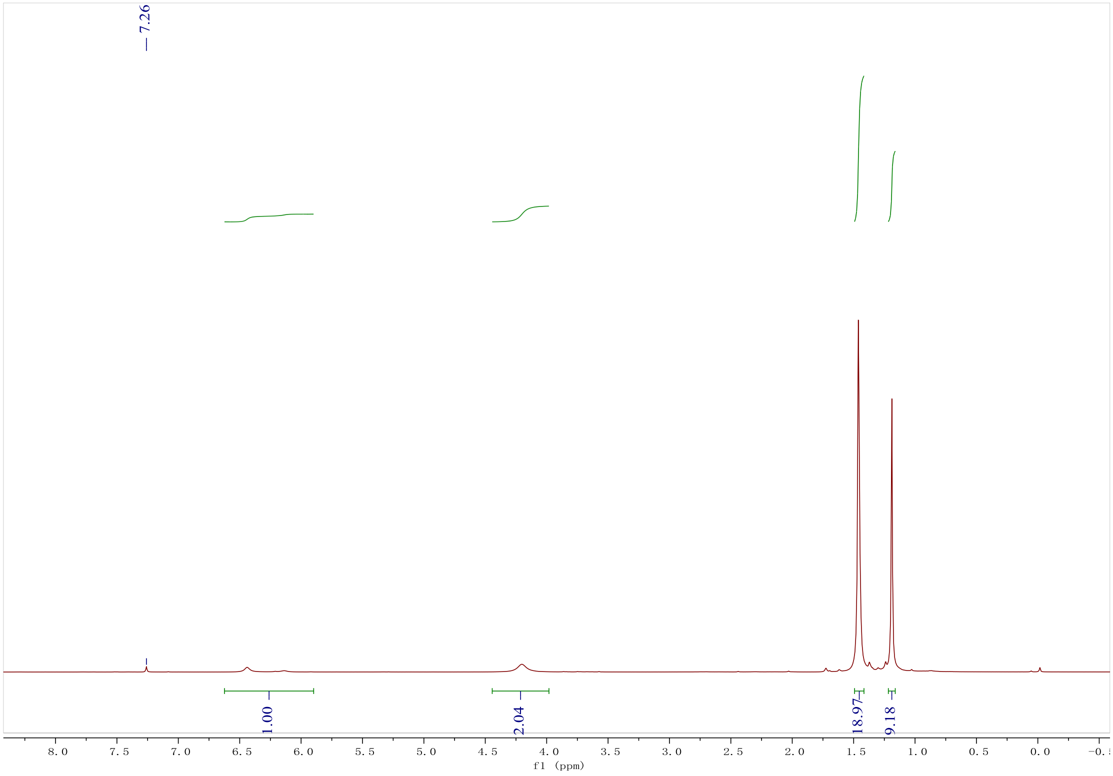
7.26
1.00
2.04
18.97
9.18
8.0 7.5 7.0 6.5 6.0 5.5 5.0 4.5 4.0 3.5 3.0 2.5 2.0 1.5 1.0 0.5 0.0
f1 (ppm)

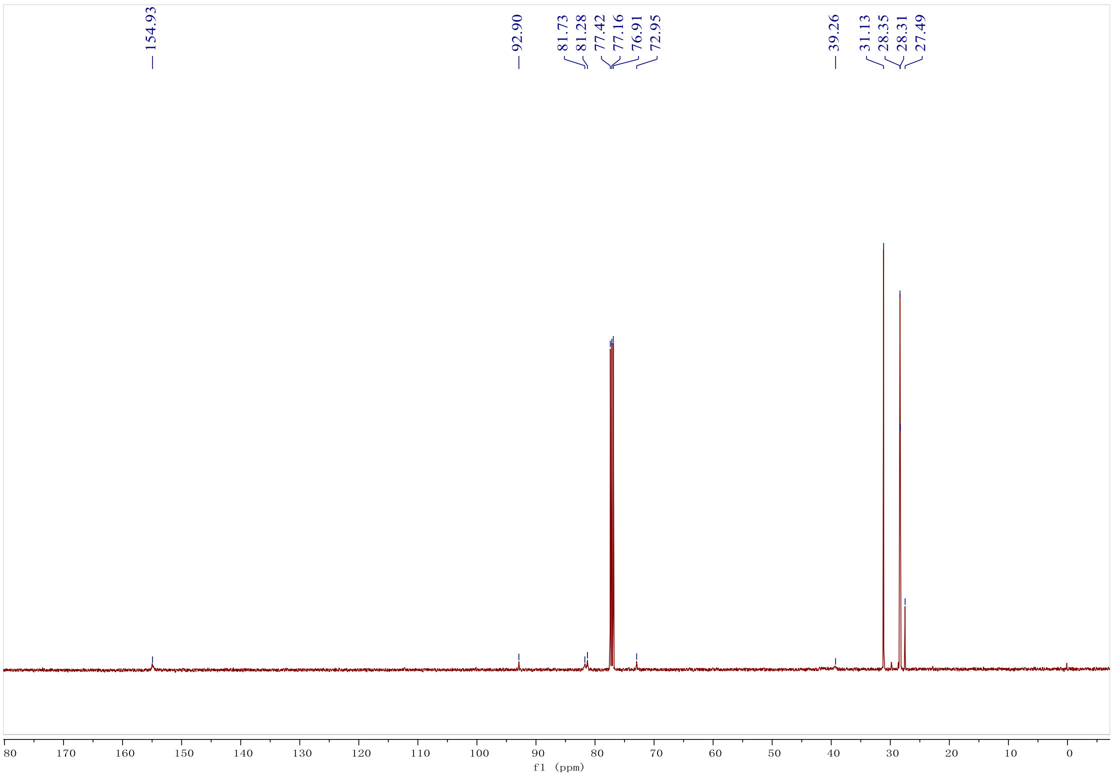
154.93
92.90
81.73
81.28
77.42
77.16
76.91
72.95
39.26
31.13
28.35
28.31
27.49
170 160 150 140 130 120 110 100 90 80 70 60 50 40 30 20 10 0
f1 (ppm)

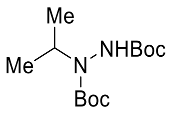
Me
Me
N
NHBoc
Boc

4j

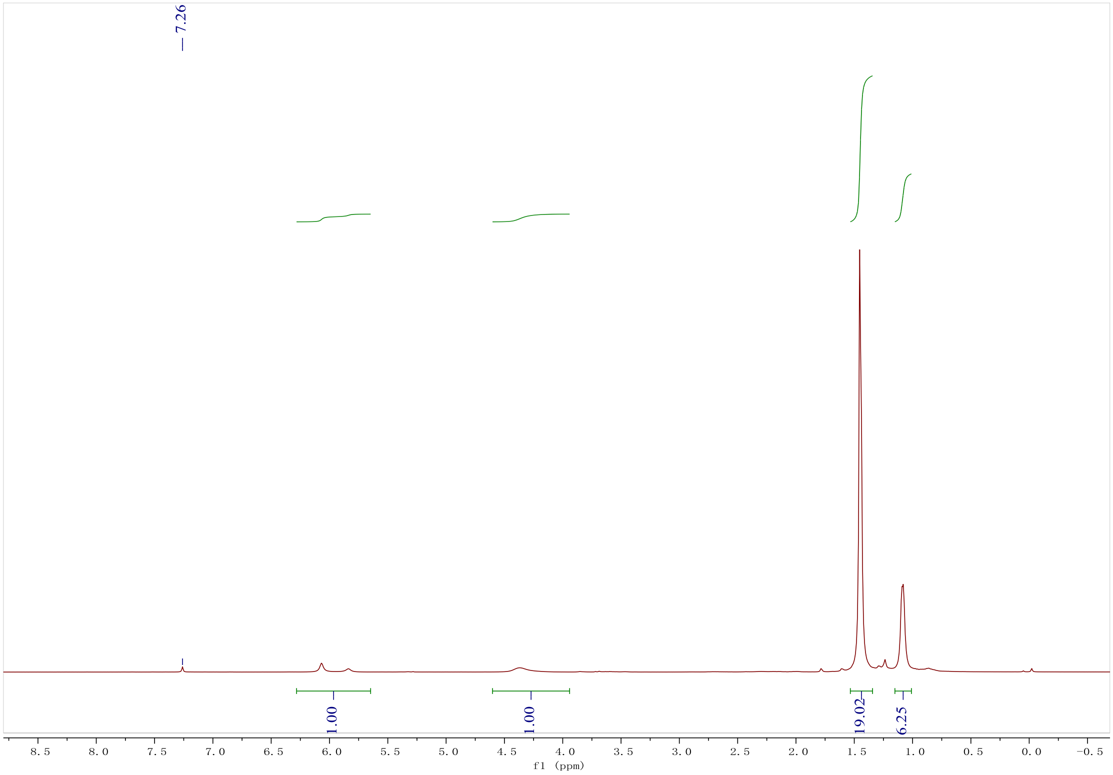
7.26
1.00
1.00
19.02
6.25
8.5
8.0
7.5
7.0
6.5
6.0
5.5
5.0
4.5
4.0
3.5
3.0
2.5
2.0
1.5
1.0
0.5
0.0
-0.5
f1 (ppm)

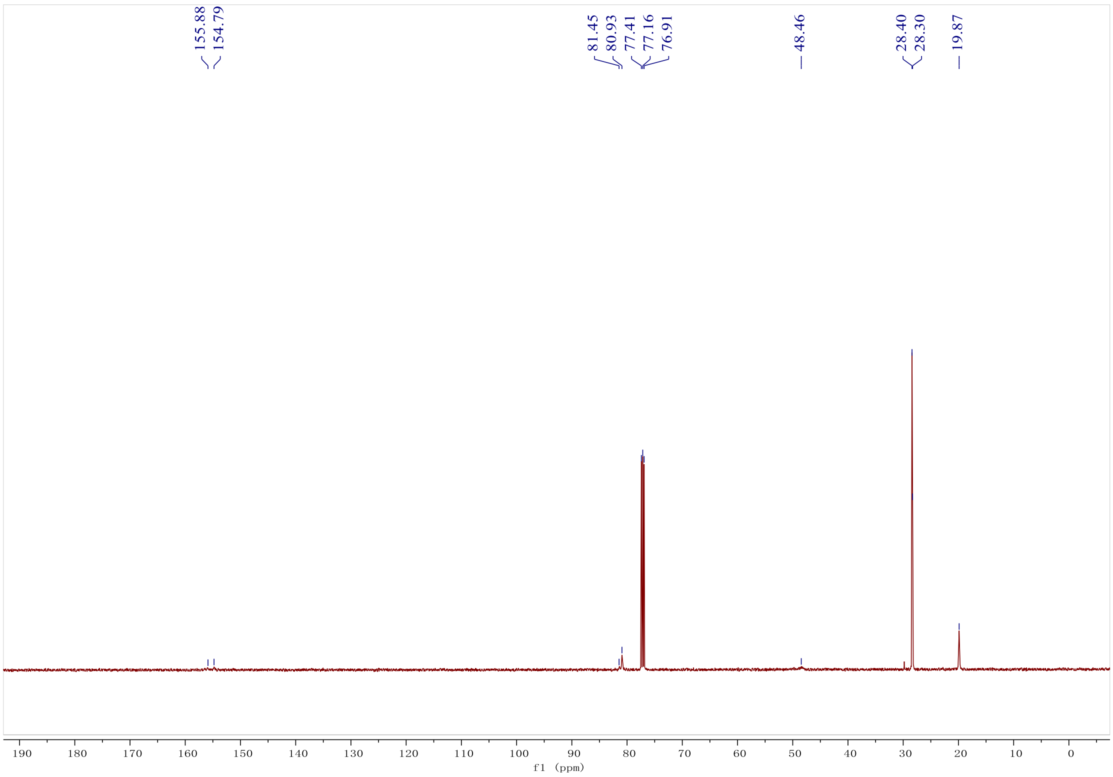
155.88
154.79
81.45
80.93
77.41
77.16
76.91
48.46
28.40
28.30
19.87
190
180
170
160
150
140
130
120
110
100
90
80
70
60
50
40
30
20
10
0
f1 (ppm)

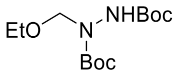
EtO
N
NHBoc
Boc

4k

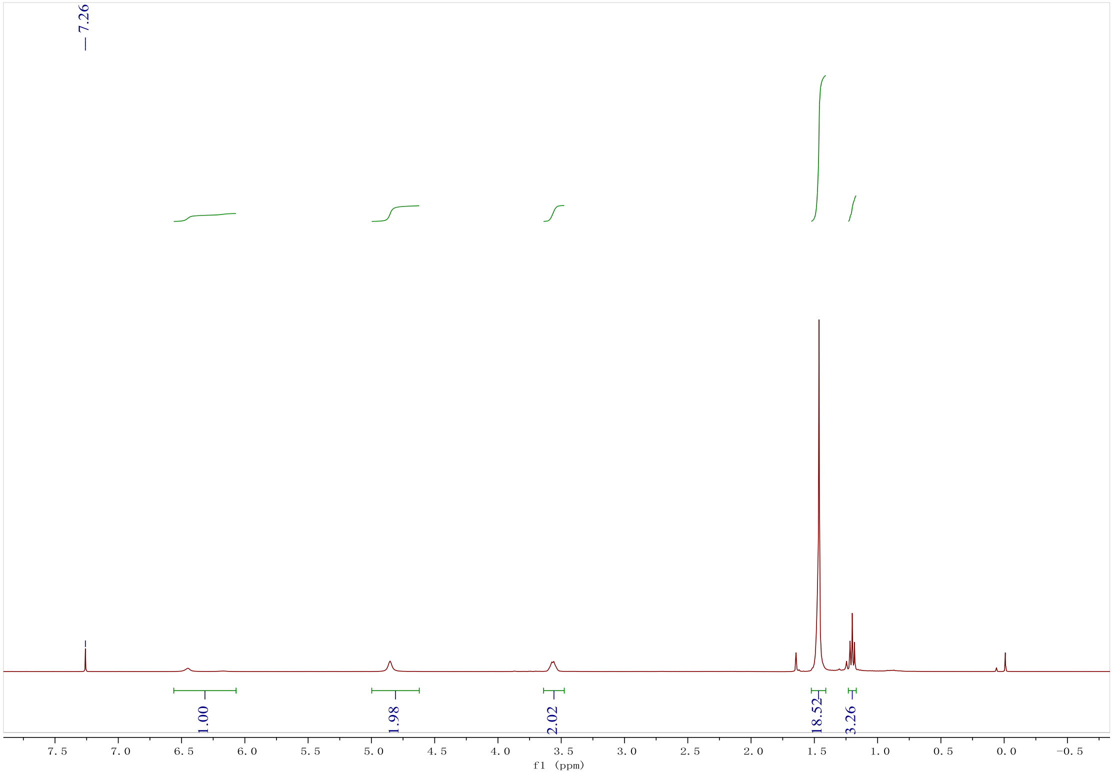
7.26
1.00
1.98
2.02
18.52
3.26
7.5 7.0 6.5 6.0 5.5 5.0 4.5 4.0 3.5 3.0 2.5 2.0 1.5 1.0 0.5 0.0 -0.5
f1 (ppm)

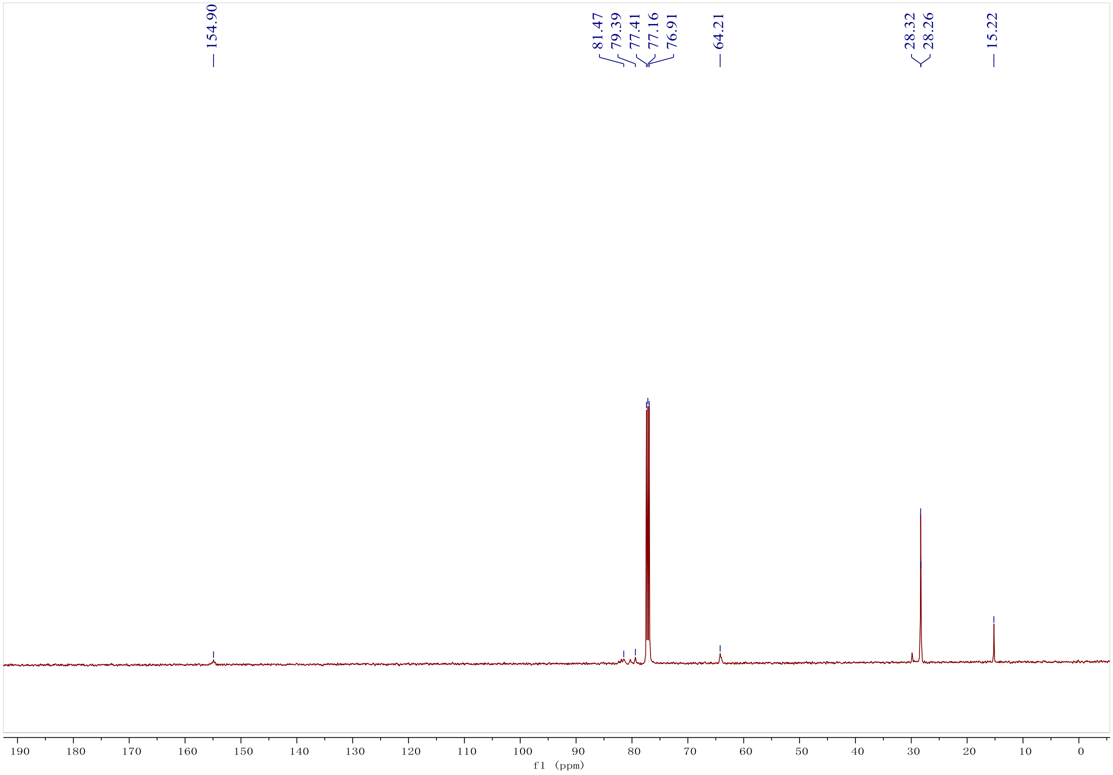
154.90
81.47
79.39
77.41
77.16
76.91
64.21
28.32
28.26
15.22
190 180 170 160 150 140 130 120 110 100 90 80 70 60 50 40 30 20 10 0
f1 (ppm)

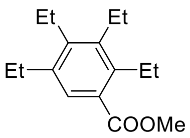
Et
Et
Et
Et
COOMe

5

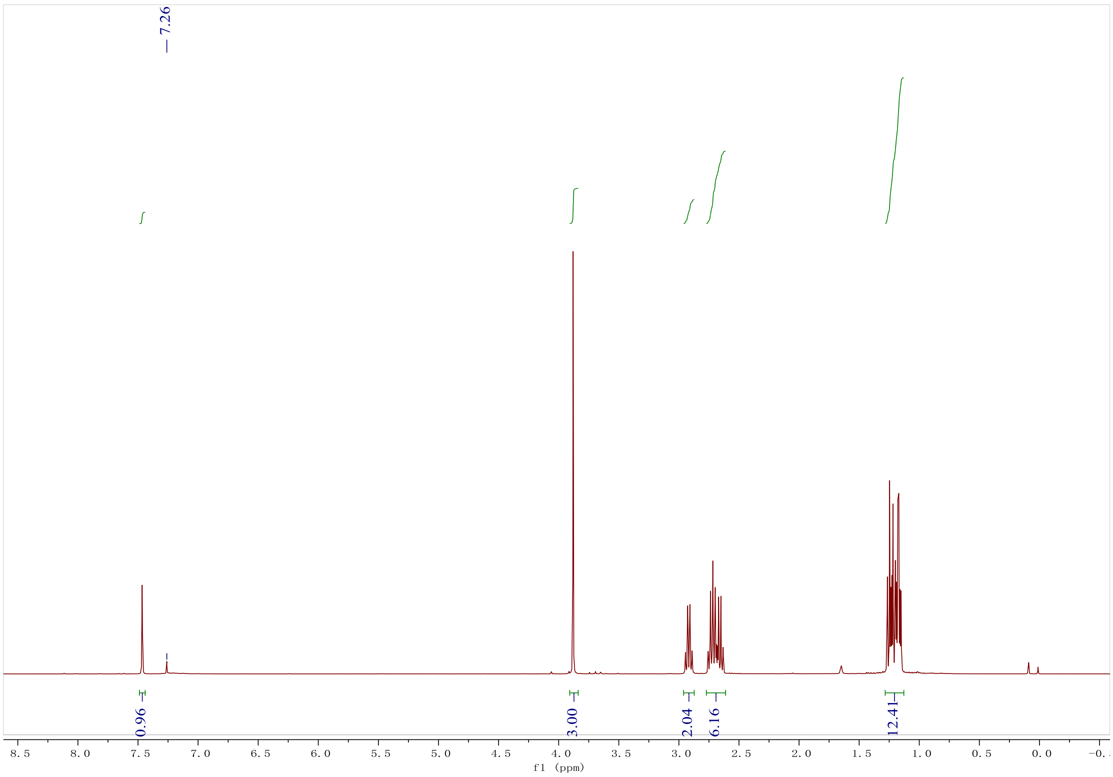
7.26
0.96
3.00
2.04
6.16
12.41
8.5 8.0 7.5 7.0 6.5 6.0 5.5 5.0 4.5 4.0 3.5 3.0 2.5 2.0 1.5 1.0 0.5 0.0
f1 (ppm)

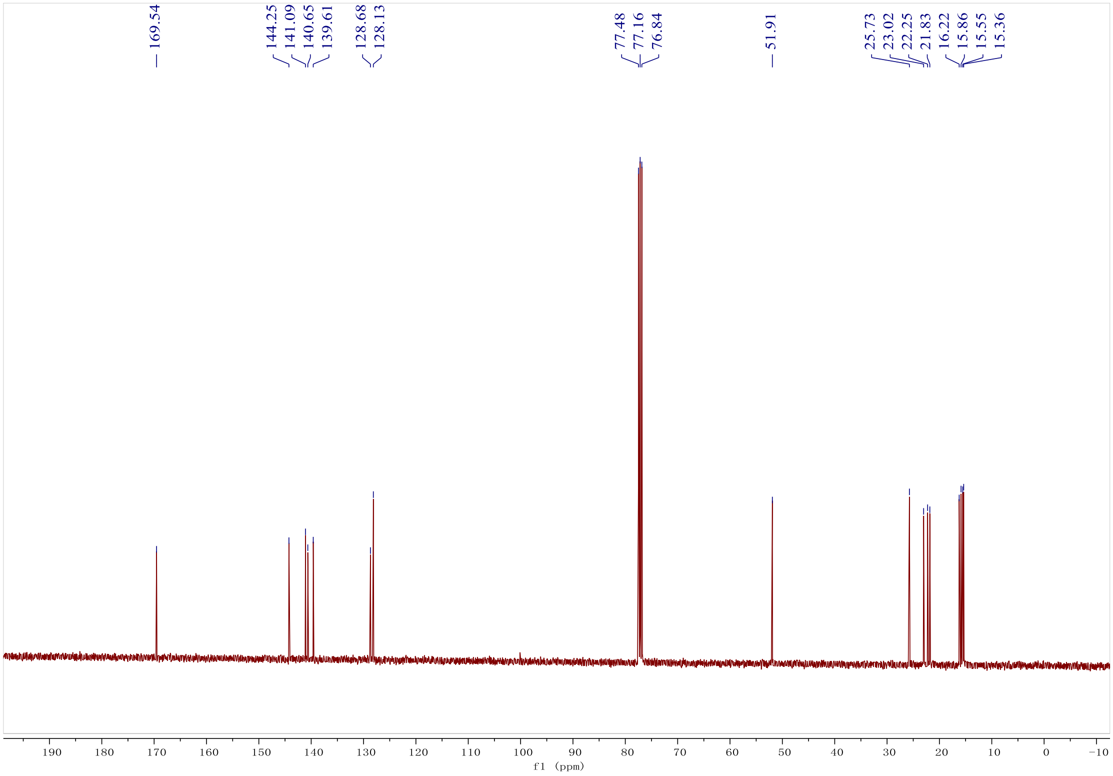
169.54
144.25
141.09
140.65
139.61
128.68
128.13
77.48
77.16
76.84
51.91
25.73
23.02
22.25
21.83
16.22
15.86
15.55
15.36
190 180 170 160 150 140 130 120 110 100 90 80 70 60 50 40 30 20 10 0 −10
f1 (ppm)

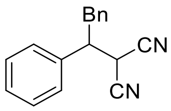
Bn
CN
CN

8b

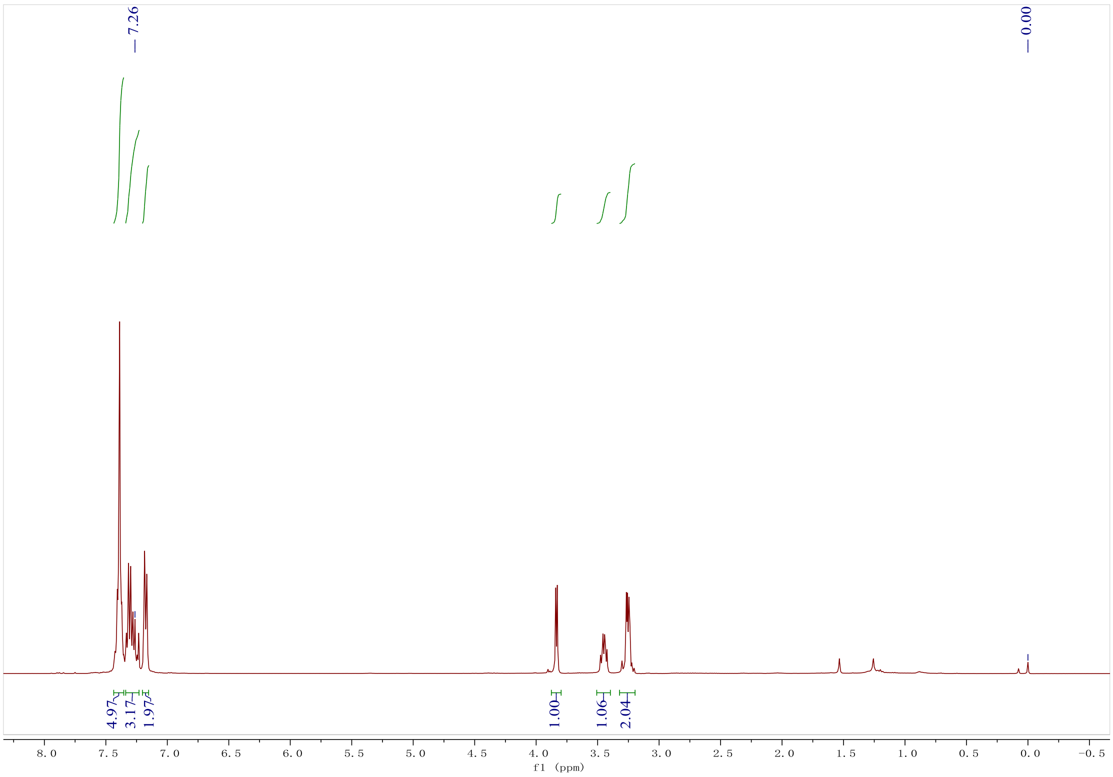
7.26
0.00
4.97
3.17
1.97
1.00
1.06
2.04
8.0 7.5 7.0 6.5 6.0 5.5 5.0 4.5 4.0 3.5 3.0 2.5 2.0 1.5 1.0 0.5 0.0 -0.5
f1 (ppm)

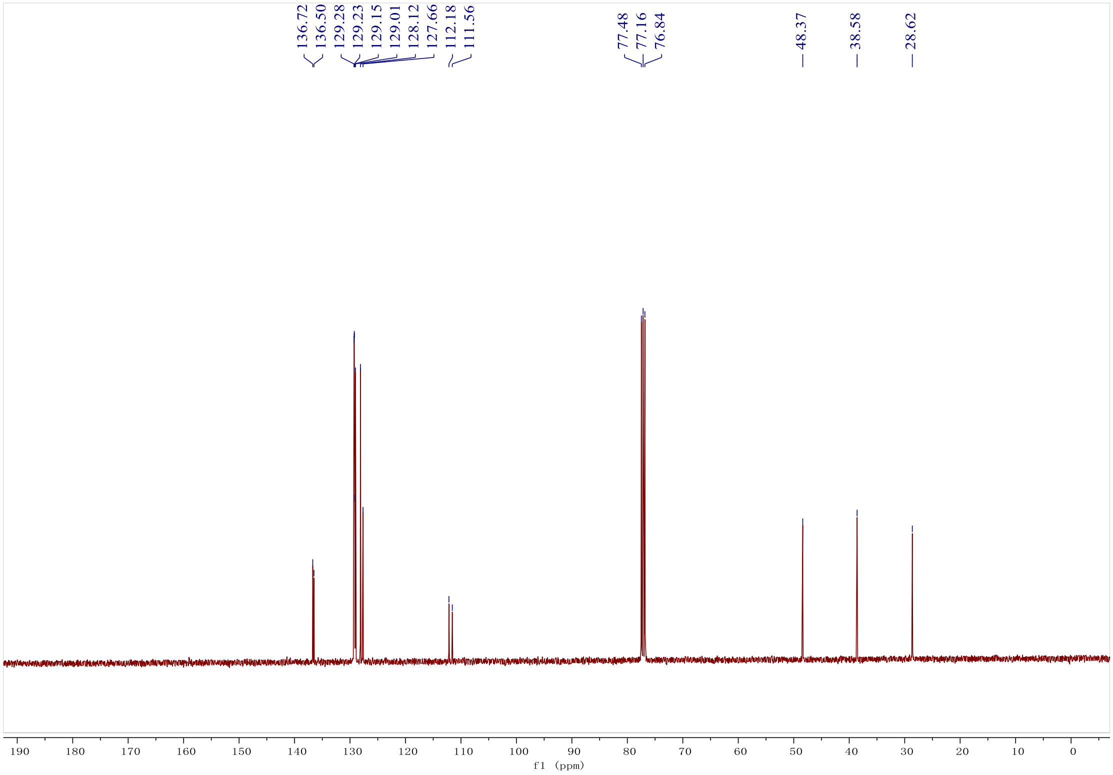
136.72
136.50
129.28
129.23
129.15
129.01
128.12
127.66
112.18
111.56
77.48
77.16
76.84
48.37
38.58
28.62
190 180 170 160 150 140 130 120 110 100 90 80 70 60 50 40 30 20 10 0
f1 (ppm)

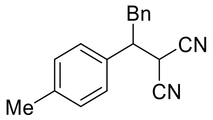
Bn
CN
CN
Me

8c

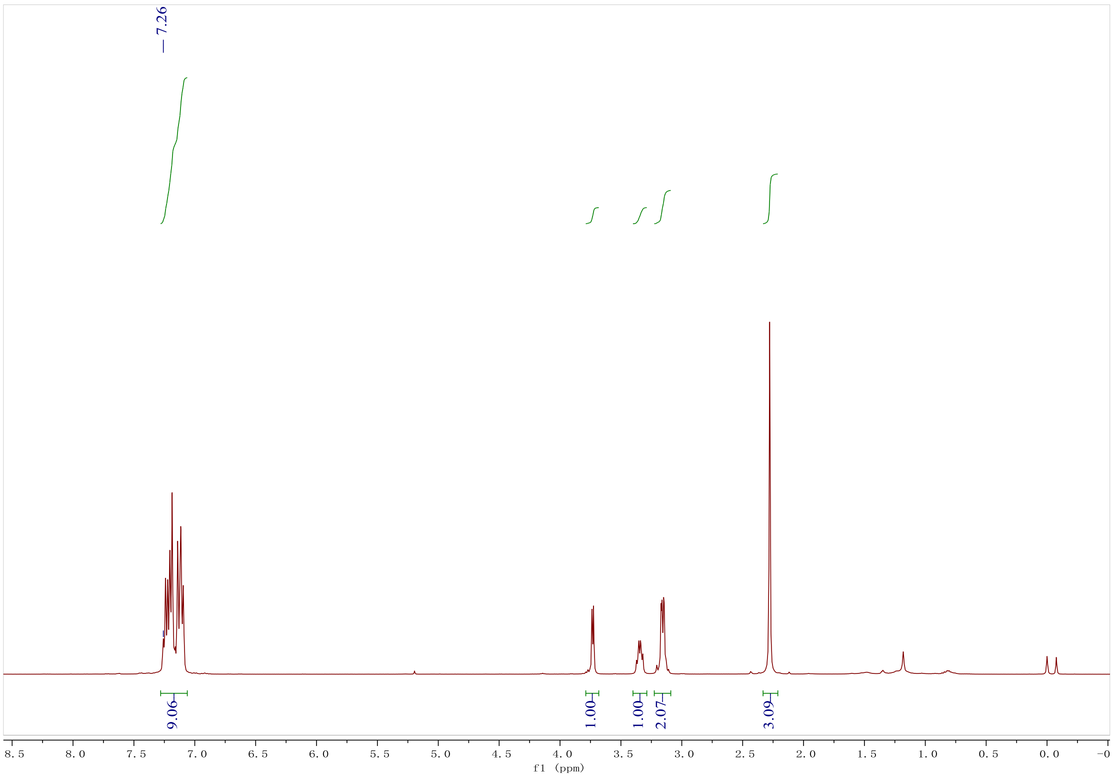
7.26
9.06
1.00
1.00
2.07
3.09
8.5
8.0
7.5
7.0
6.5
6.0
5.5
5.0
4.5
4.0
3.5
3.0
2.5
2.0
1.5
1.0
0.5
0.0
f1 (ppm)

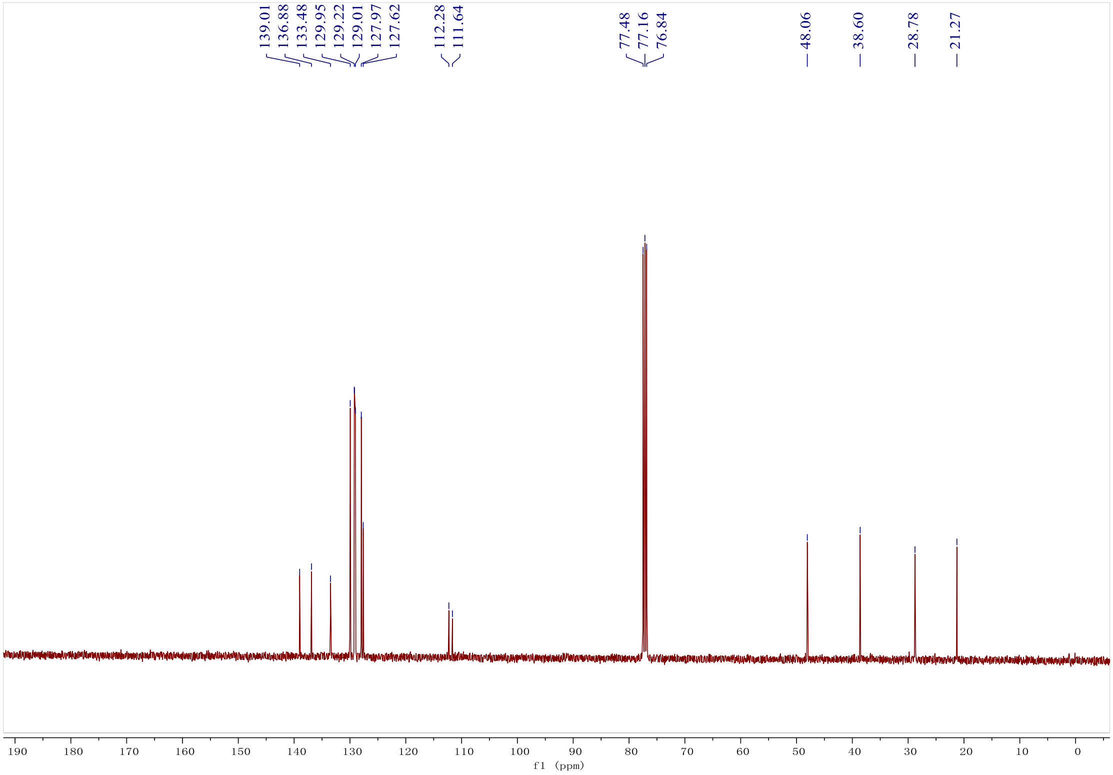
139.01
136.88
133.48
129.95
129.22
129.01
127.97
127.62
112.28
111.64
77.48
77.16
76.84
48.06
38.60
28.78
21.27
190
180
170
160
150
140
130
120
110
100
90
80
70
60
50
40
30
20
10
0
f1 (ppm)

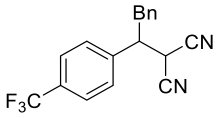

Bn
CN
CN
F3C

8d

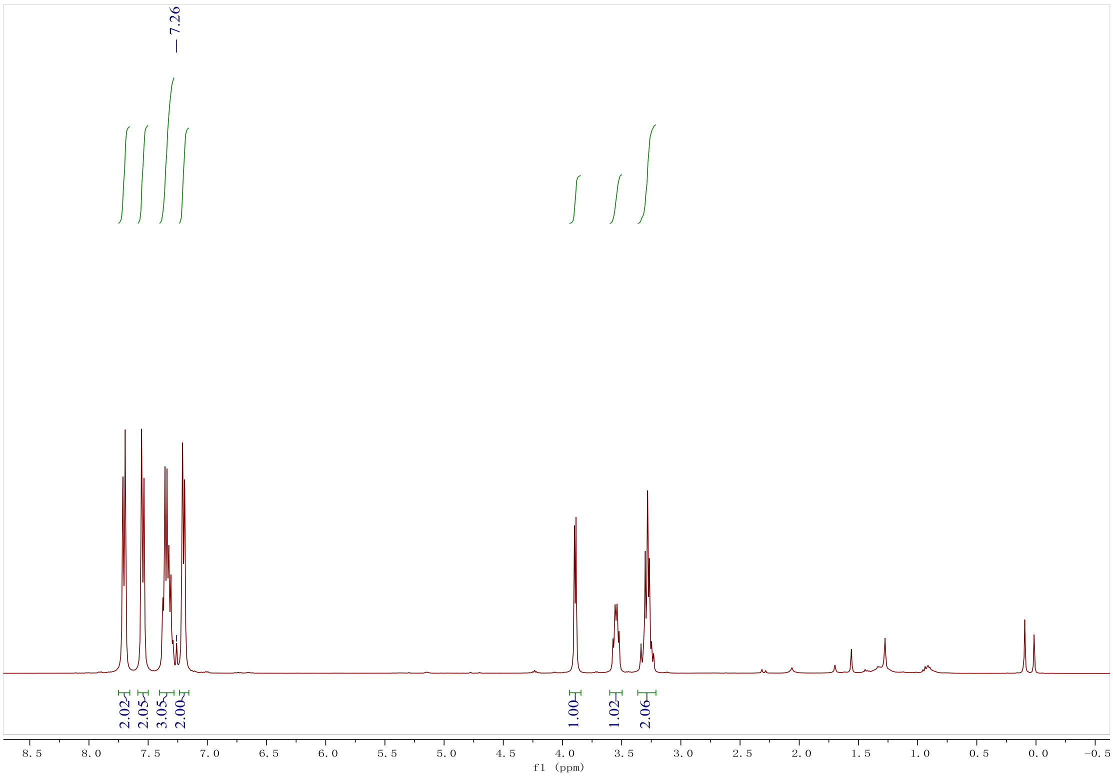

7.26
2.02
2.05
3.05
2.00
1.00
1.02
2.06
8.5 8.0 7.5 7.0 6.5 6.0 5.5 5.0 4.5 4.0 3.5 3.0 2.5 2.0 1.5 1.0 0.5 0.0 -0.5
f1 (ppm)

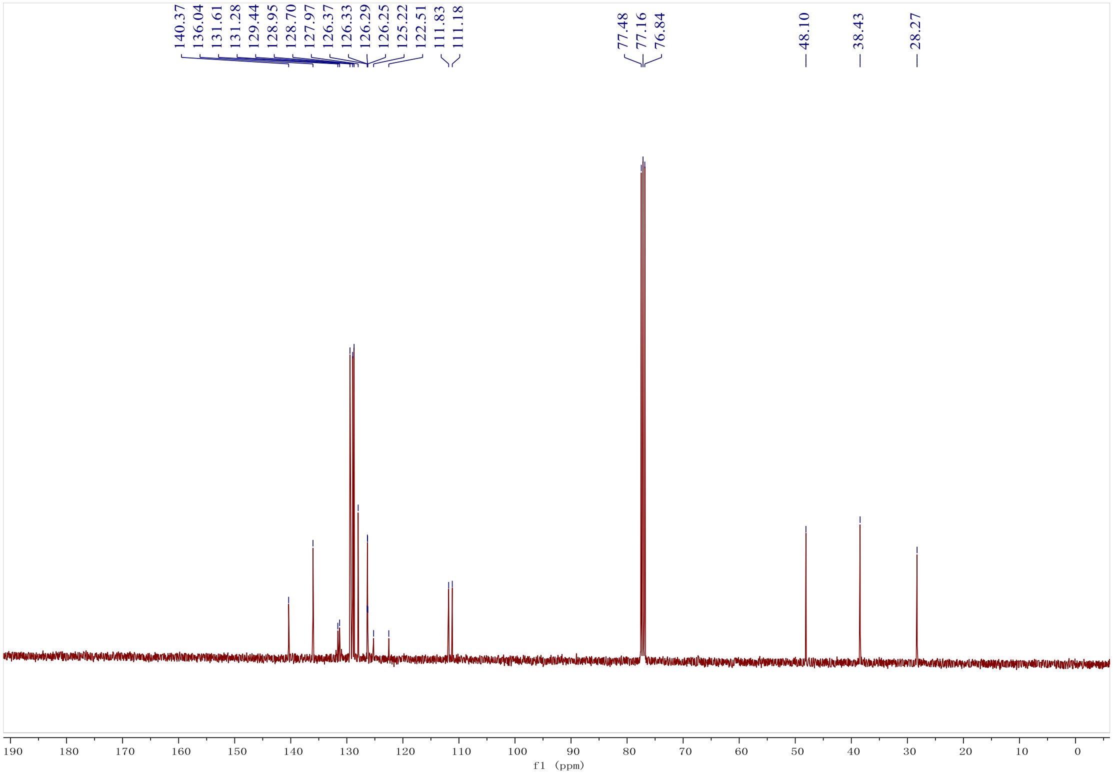

140.37
136.04
131.61
131.28
129.44
128.95
128.70
127.97
126.37
126.33
126.29
126.25
125.22
122.51
111.83
111.18
77.48
77.16
76.84
48.10
38.43
28.27
190 180 170 160 150 140 130 120 110 100 90 80 70 60 50 40 30 20 10 0
f1 (ppm)

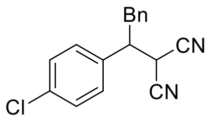
Bn
CN
CN
Cl

8e

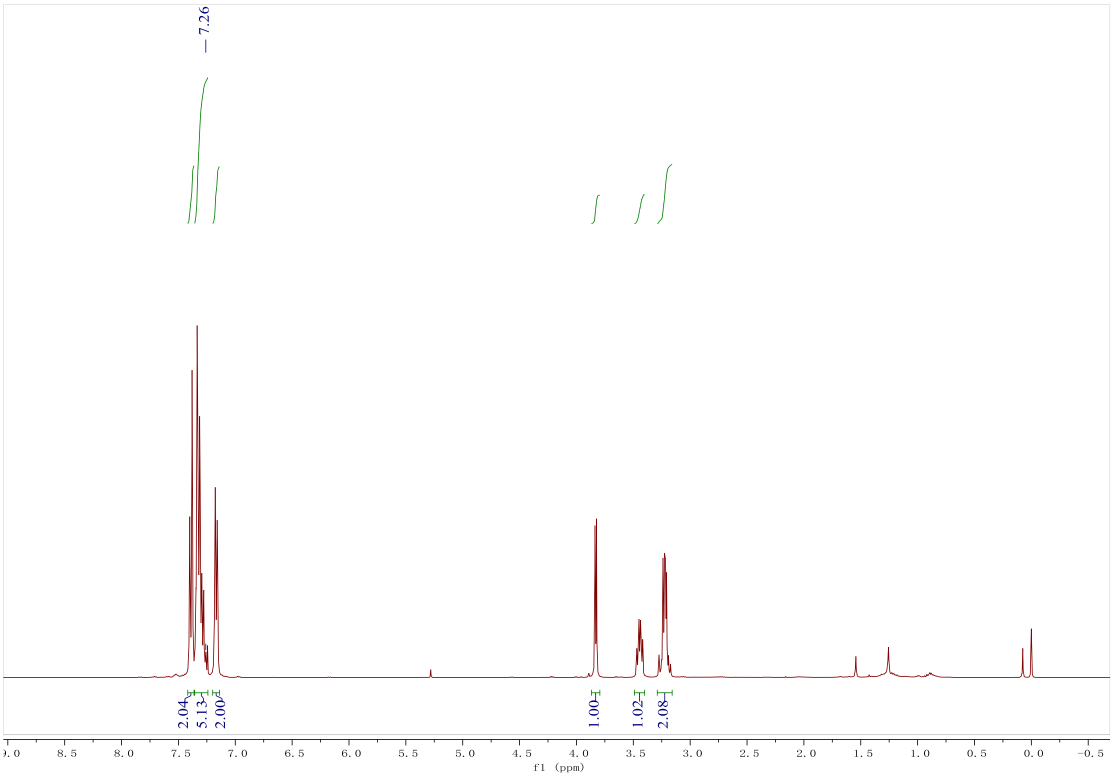
7.26
2.04
5.13
2.00
1.00
1.02
2.08
9.0 8.5 8.0 7.5 7.0 6.5 6.0 5.5 5.0 4.5 4.0 3.5 3.0 2.5 2.0 1.5 1.0 0.5 0.0 -0.5
f1 (ppm)

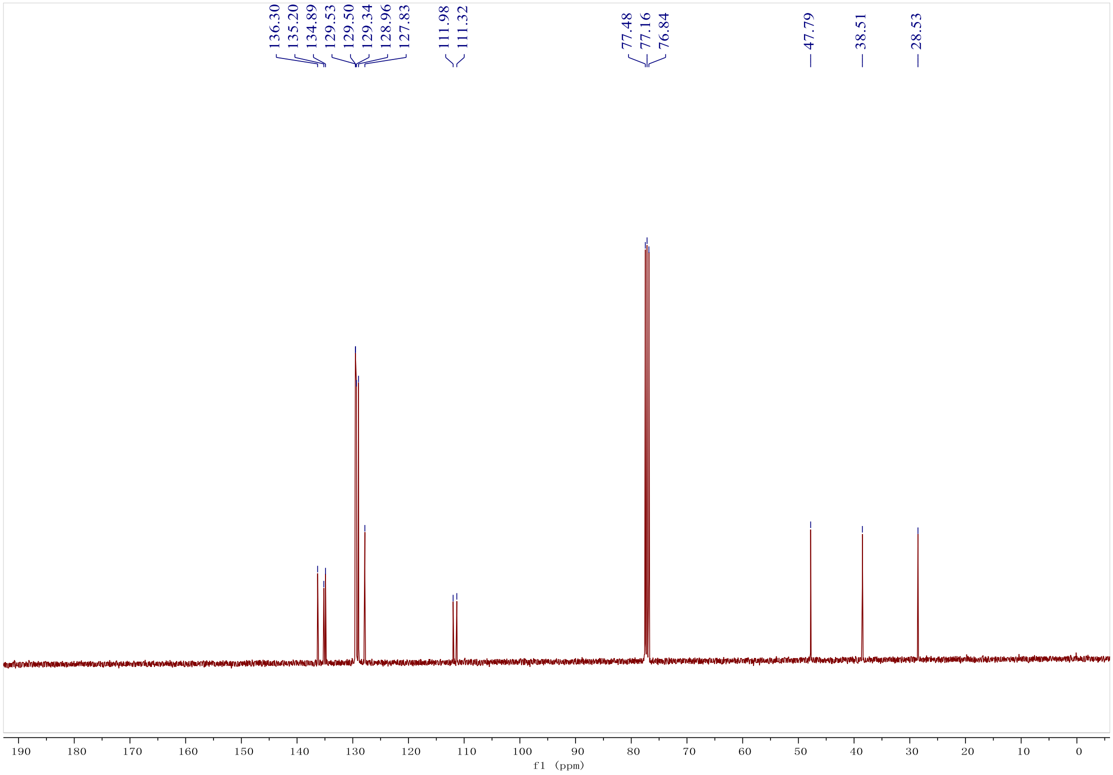
136.30
135.20
134.89
129.53
129.50
129.34
128.96
127.83
111.98
111.32
77.48
77.16
76.84
47.79
38.51
28.53
190 180 170 160 150 140 130 120 110 100 90 80 70 60 50 40 30 20 10 0
f1 (ppm)

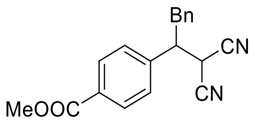
Bn
CN
CN
MeOOC

8f

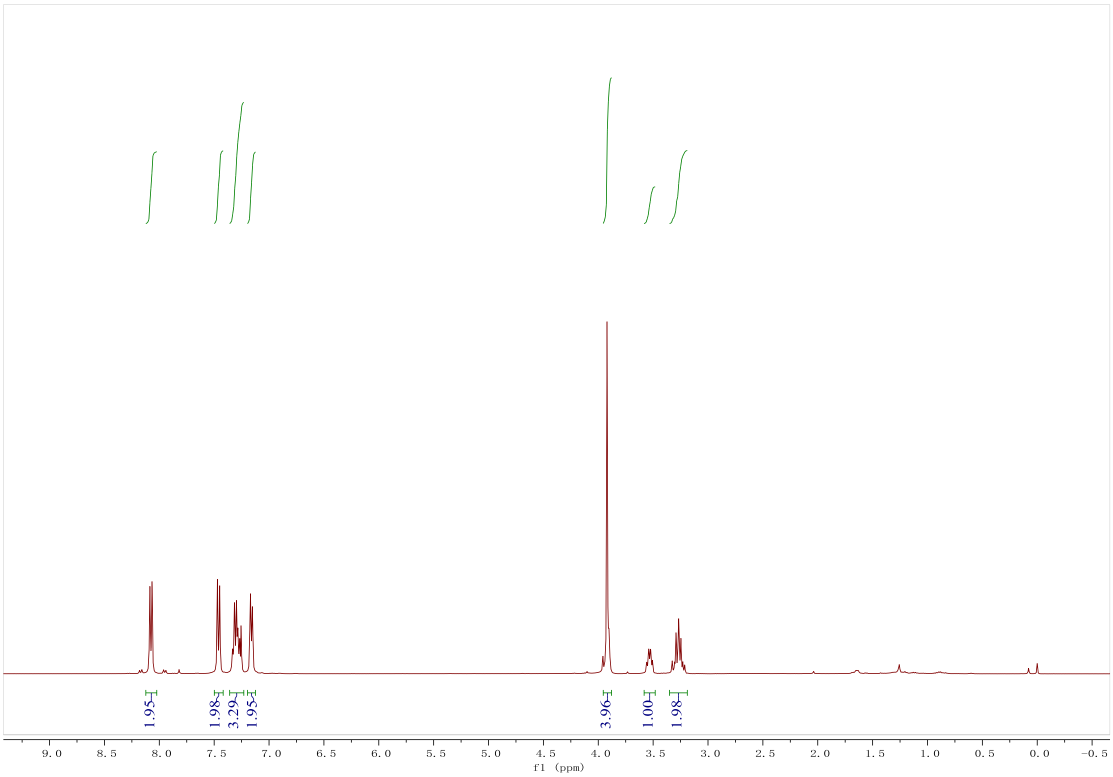
1.95
1.98
3.29
1.95
3.96
1.00
1.98
9.0 8.5 8.0 7.5 7.0 6.5 6.0 5.5 5.0 4.5 4.0 3.5 3.0 2.5 2.0 1.5 1.0 0.5 0.0 -0.5
f1 (ppm)

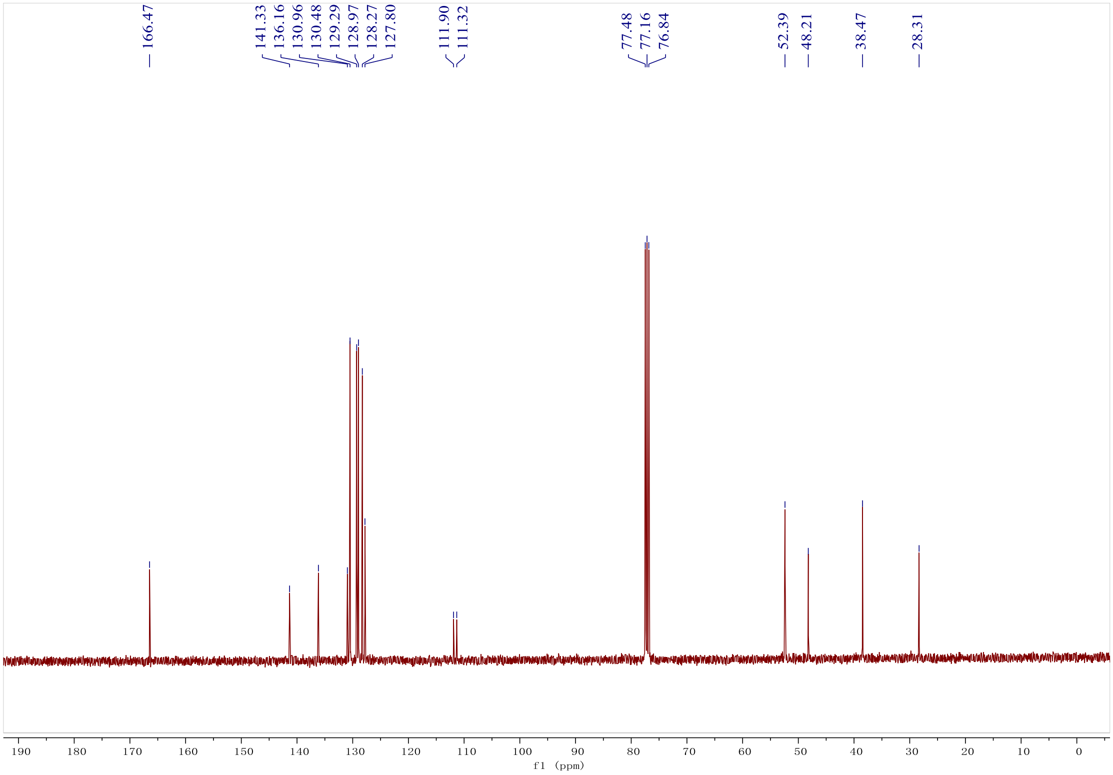
166.47
141.33
136.16
130.96
130.48
129.29
128.97
128.27
127.80
111.90
111.32
77.48
77.16
76.84
52.39
48.21
38.47
28.31
190 180 170 160 150 140 130 120 110 100 90 80 70 60 50 40 30 20 10 0
f1 (ppm)

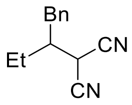
Bn
CN
Et
CN

8g

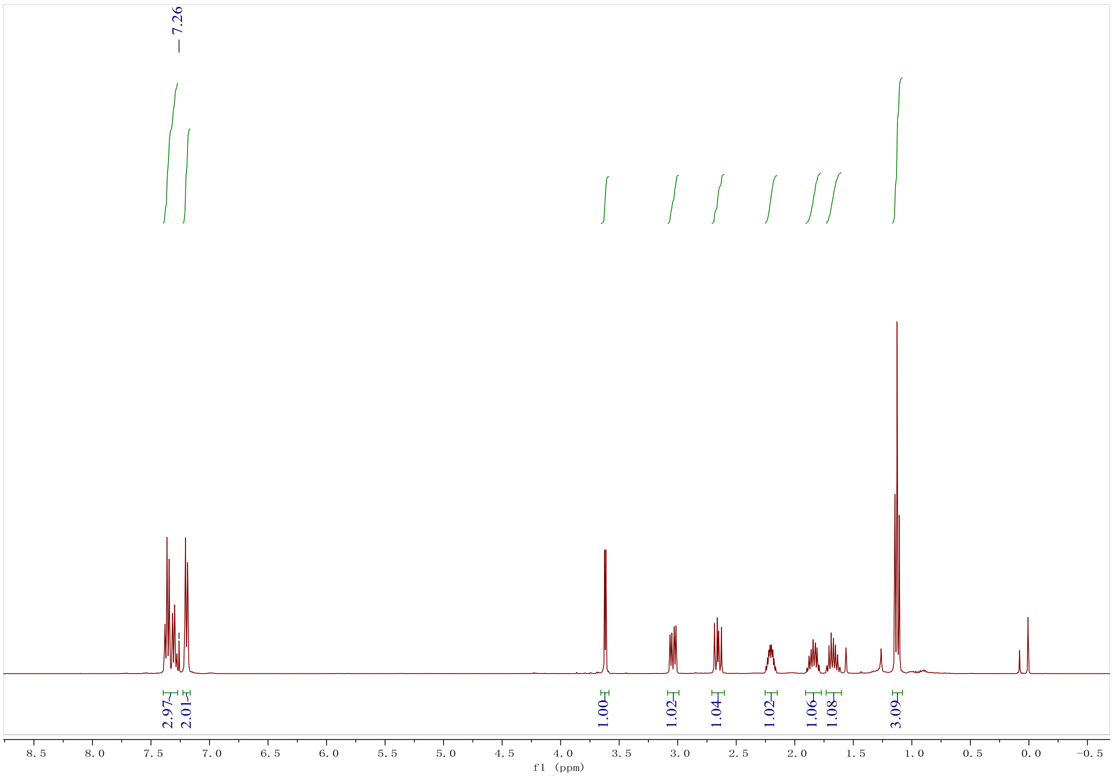
7.26
2.97
2.01
1.00
1.02
1.04
1.02
1.06
1.08
3.09
f1 (ppm)

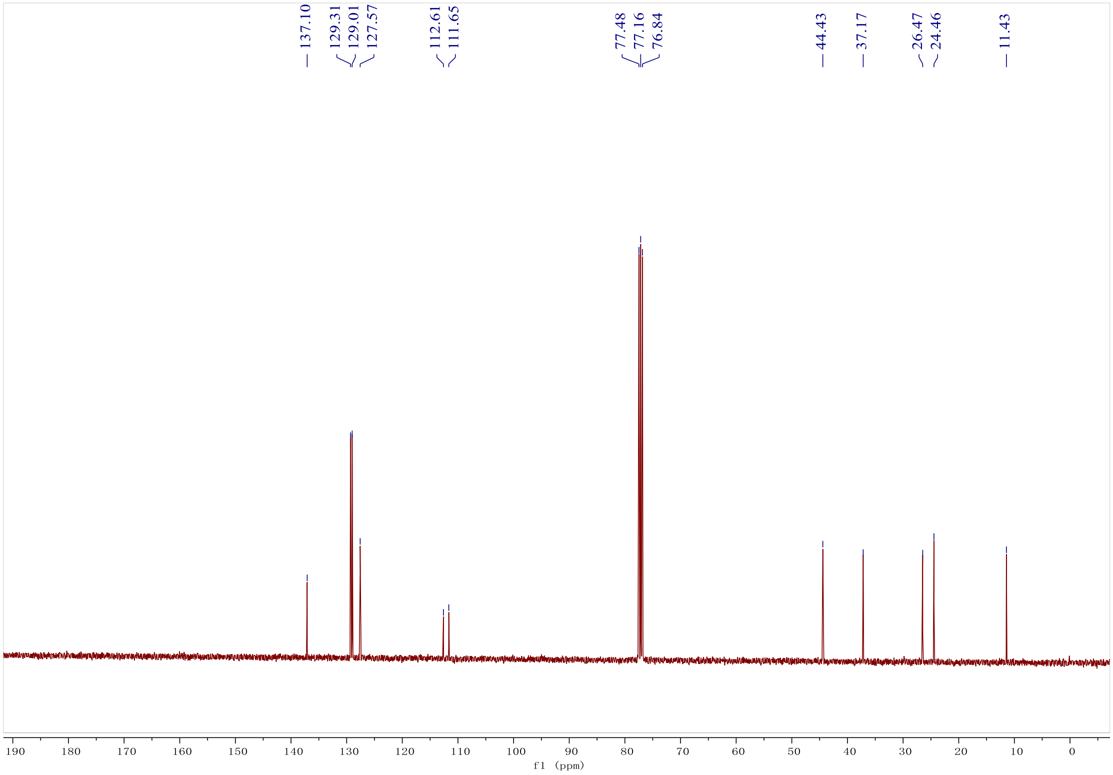
137.10
129.31
129.01
127.57
112.61
111.65
77.48
77.16
76.84
44.43
37.17
26.47
24.46
11.43
f1 (ppm)

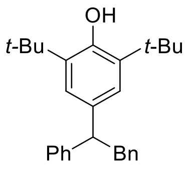
OH
t-Bu
t-Bu
Ph
Bn

8h

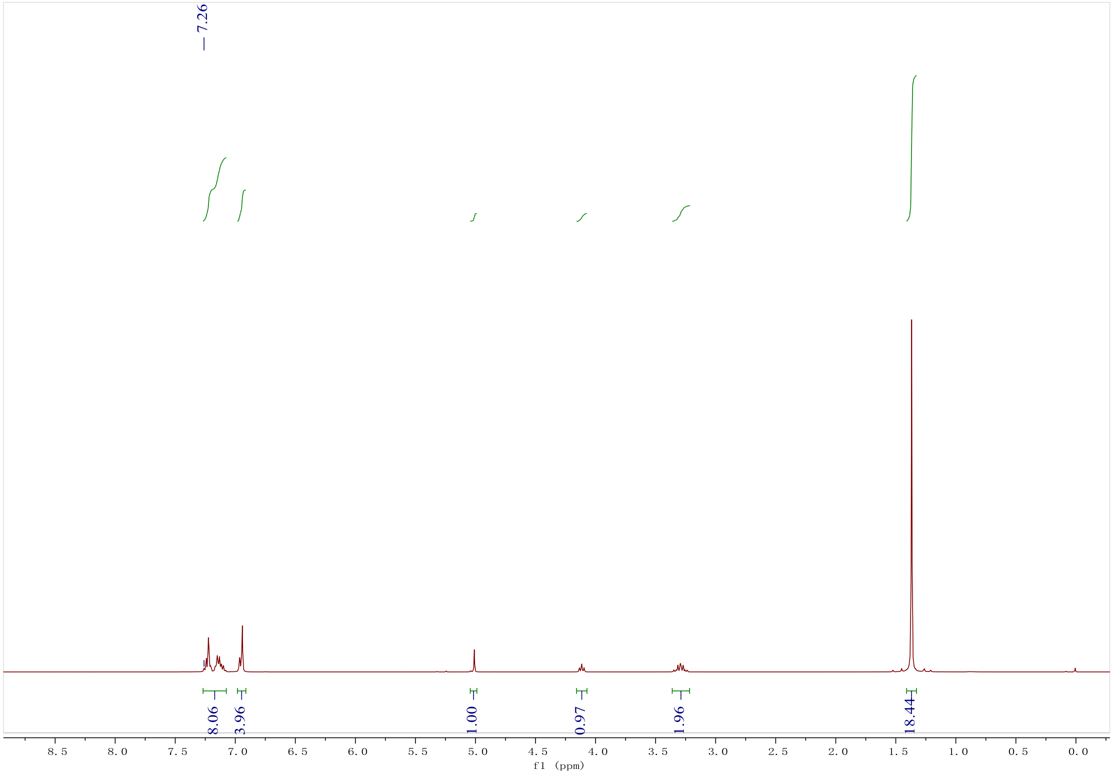
7.26
8.06
3.96
1.00
0.97
1.96
18.44
8.5 8.0 7.5 7.0 6.5 6.0 5.5 5.0 4.5 4.0 3.5 3.0 2.5 2.0 1.5 1.0 0.5 0.0
f1 (ppm)

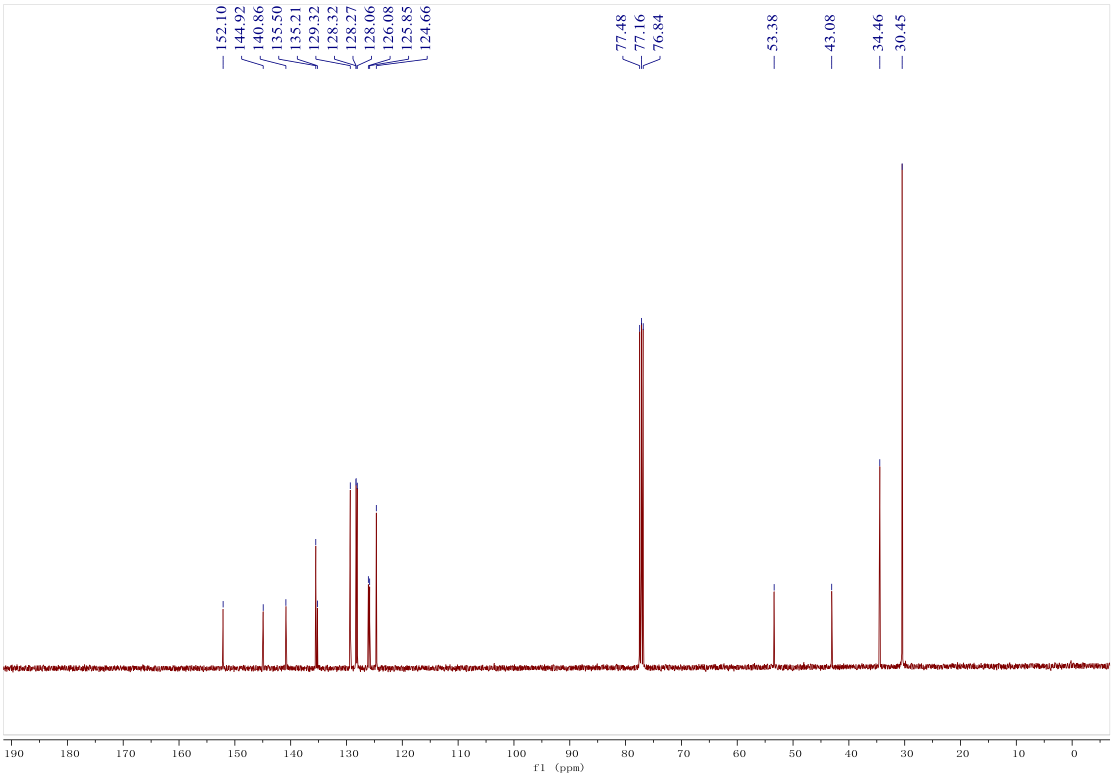
152.10
144.92
140.86
135.50
135.21
129.32
128.32
128.27
128.06
126.08
125.85
124.66
77.48
77.16
76.84
53.38
43.08
34.46
30.45
190 180 170 160 150 140 130 120 110 100 90 80 70 60 50 40 30 20 10 0
f1 (ppm)

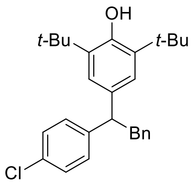
OH
t-Bu
t-Bu
Bn
Cl

8i

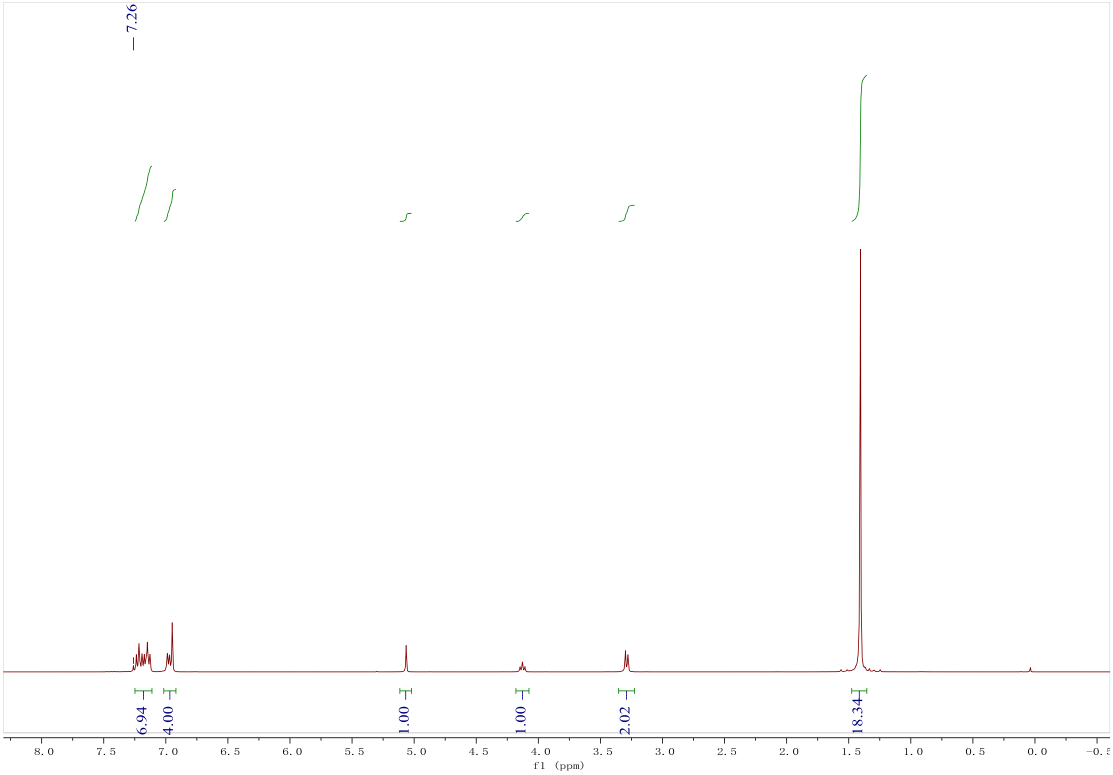
7.26
6.94
4.00
1.00
1.00
2.02
18.34
8.0
7.5
7.0
6.5
6.0
5.5
5.0
4.5
4.0
3.5
3.0
2.5
2.0
1.5
1.0
0.5
0.0
-0.5
f1 (ppm)

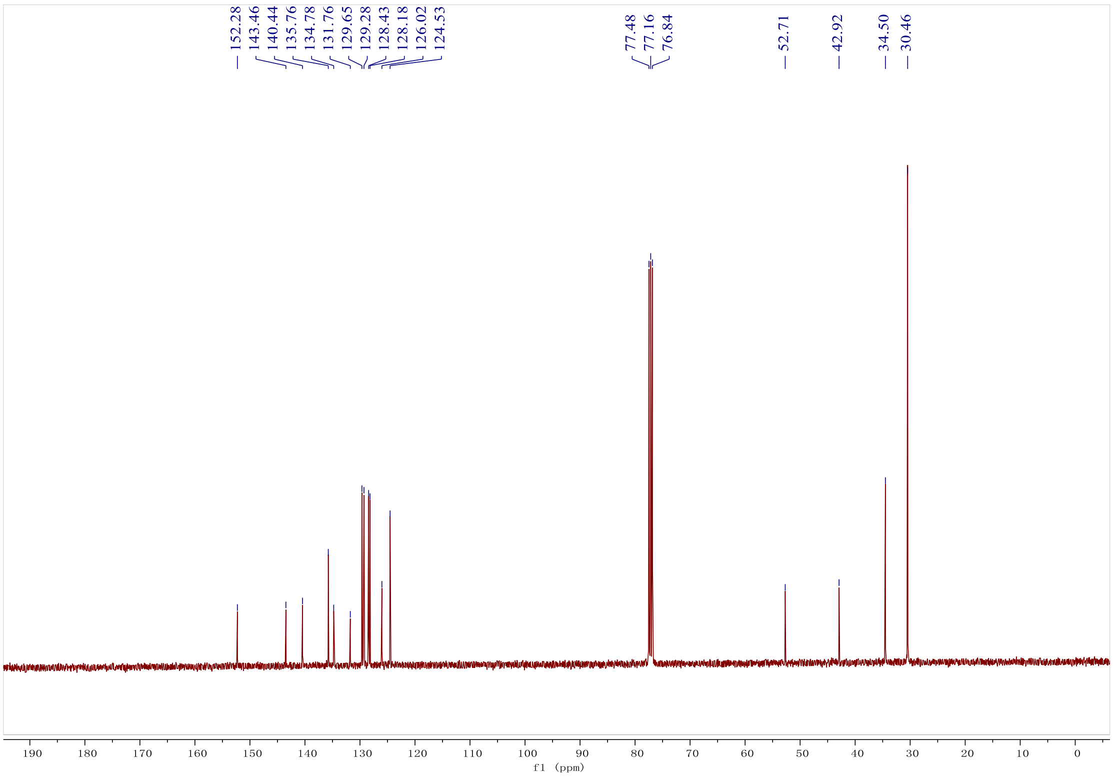
152.28
143.46
140.44
135.76
134.78
131.76
129.65
129.28
128.43
128.18
126.02
124.53
77.48
77.16
76.84
52.71
42.92
34.50
30.46
190
180
170
160
150
140
130
120
110
100
90
80
70
60
50
40
30
20
10
0
f1 (ppm)

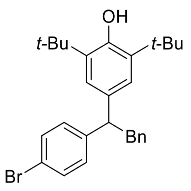
OH
t-Bu
t-Bu
Bn
Br

8j

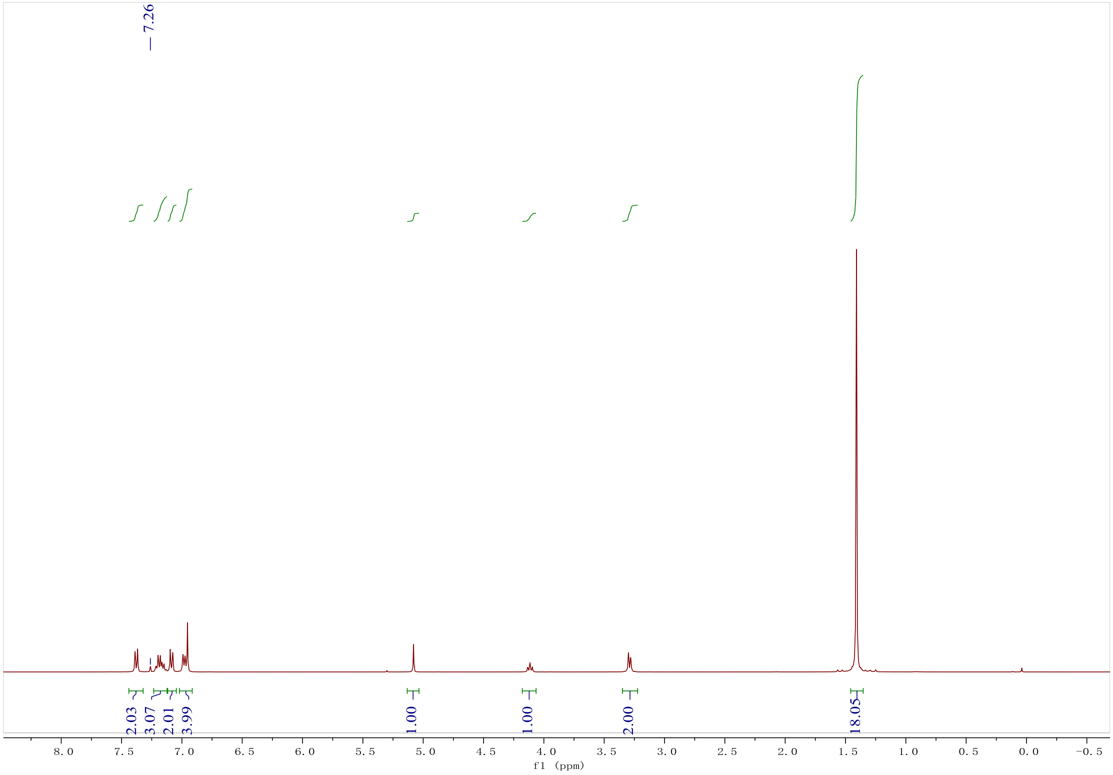
7.26
2.03
3.07
2.01
3.99
1.00
1.00
2.00
18.05
8.0
7.5
7.0
6.5
6.0
5.5
5.0
4.5
4.0
3.5
3.0
2.5
2.0
1.5
1.0
0.5
0.0
-0.5
f1 (ppm)

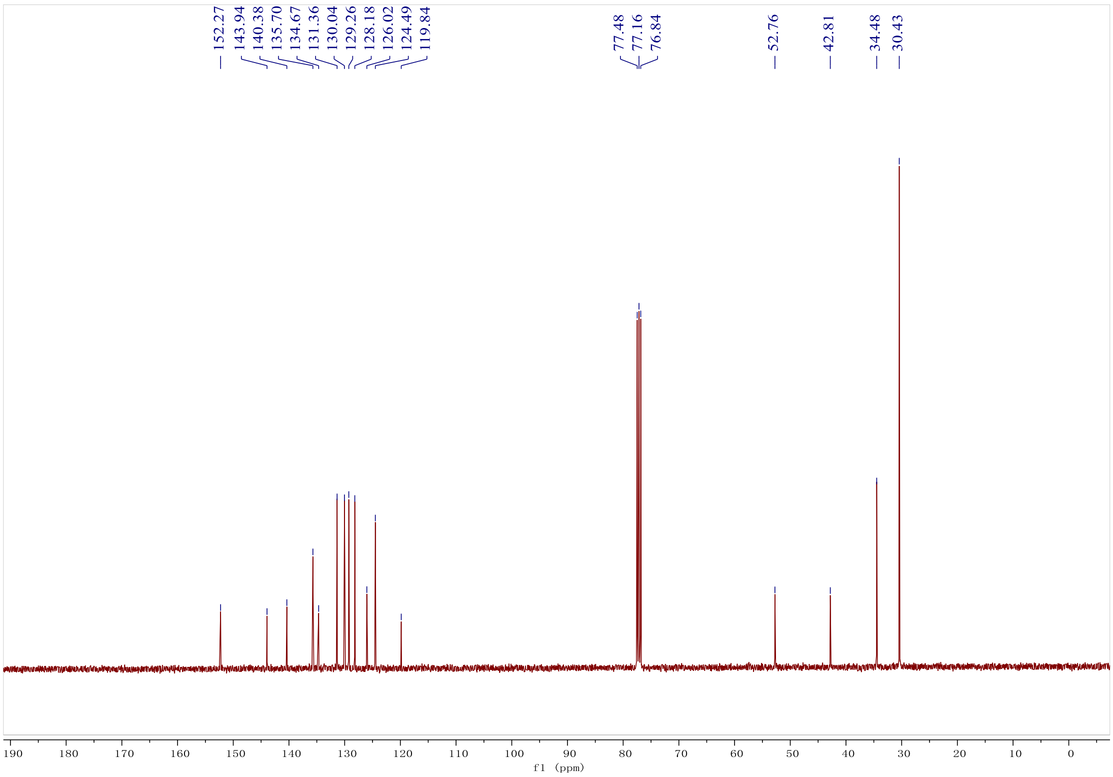
152.27
143.94
140.38
135.70
134.67
131.36
130.04
129.26
128.18
126.02
124.49
119.84
77.48
77.16
76.84
52.76
42.81
34.48
30.43
190
180
170
160
150
140
130
120
110
100
90
80
70
60
50
40
30
20
10
0
f1 (ppm)

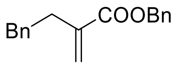
Bn
COOBn

10k

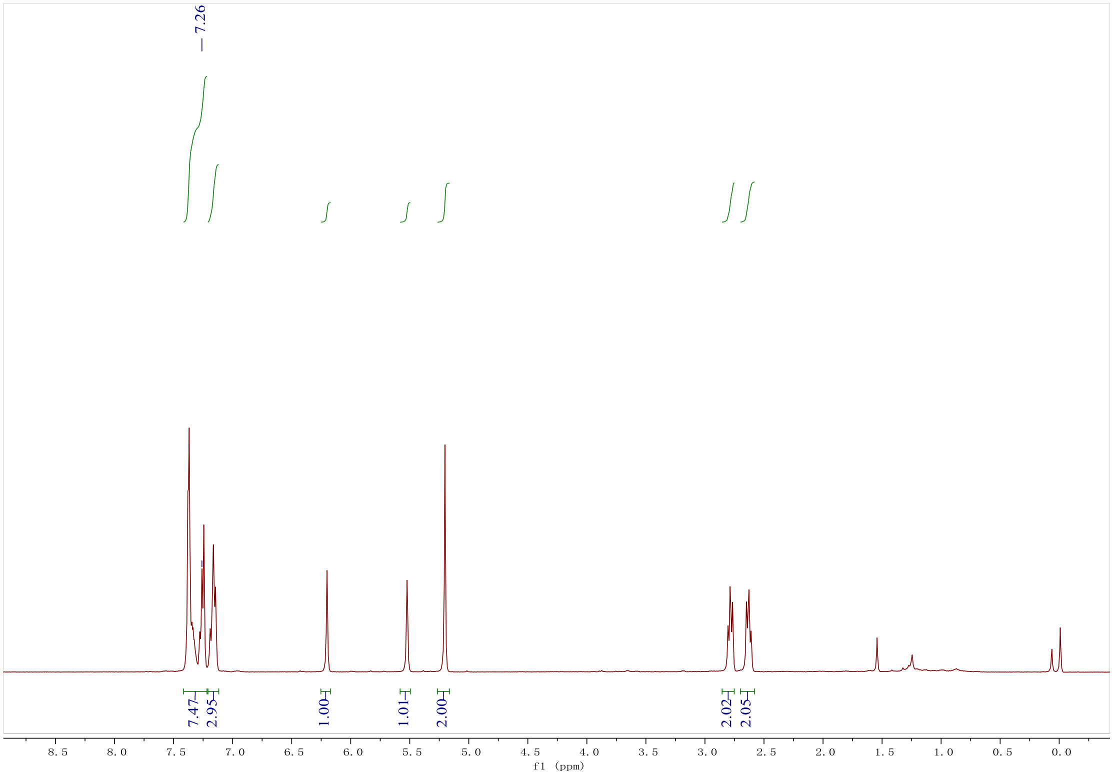
7.26
7.47
2.95
1.00
1.01
2.00
2.02
2.05
8.5 8.0 7.5 7.0 6.5 6.0 5.5 5.0 4.5 4.0 3.5 3.0 2.5 2.0 1.5 1.0 0.5 0.0
f1 (ppm)

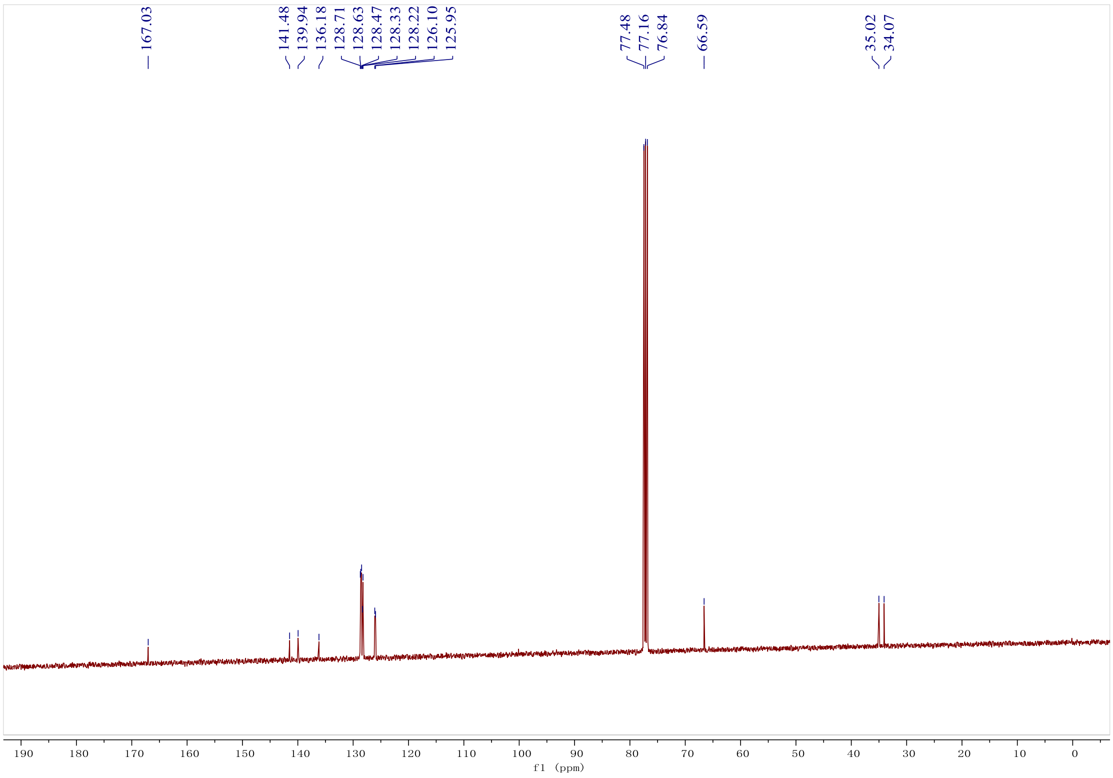
167.03
141.48
139.94
136.18
128.71
128.63
128.47
128.33
128.22
126.10
125.95
77.48
77.16
76.84
66.59
35.02
34.07
190 180 170 160 150 140 130 120 110 100 90 80 70 60 50 40 30 20 10 0
f1 (ppm)

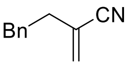
Bn
CN

10l

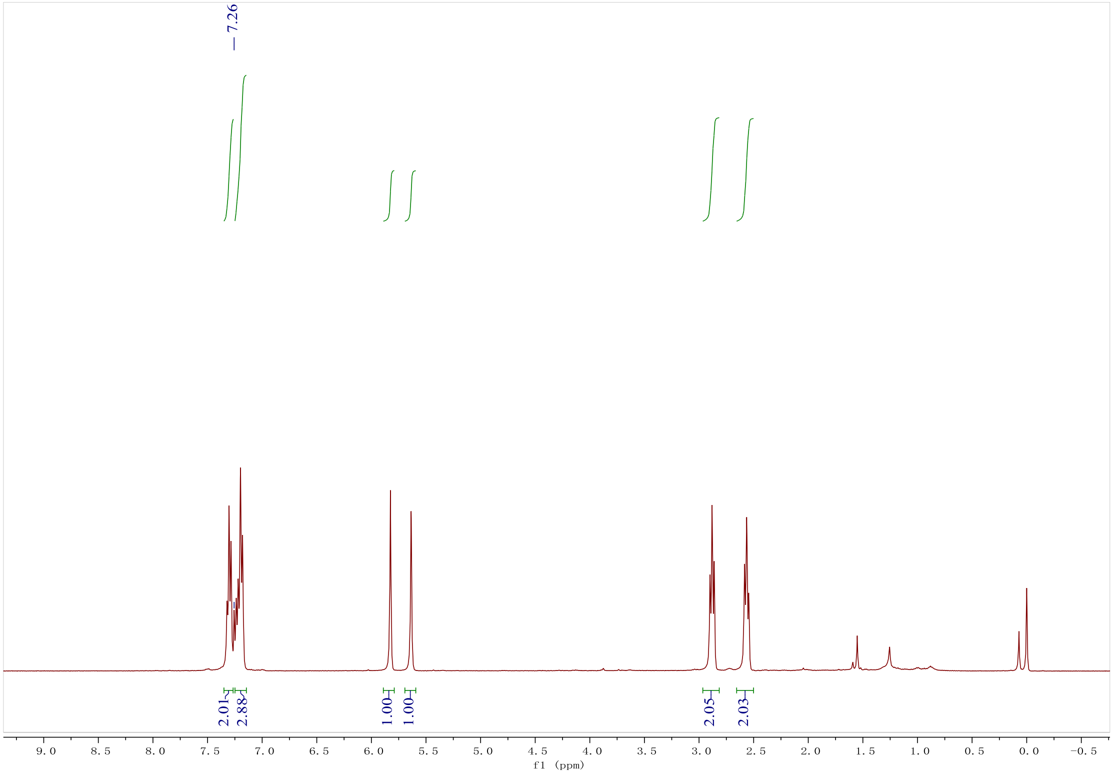
7.26
2.01
2.88
1.00
1.00
2.05
2.03
9.0 8.5 8.0 7.5 7.0 6.5 6.0 5.5 5.0 4.5 4.0 3.5 3.0 2.5 2.0 1.5 1.0 0.5 0.0 -0.5
f1 (ppm)

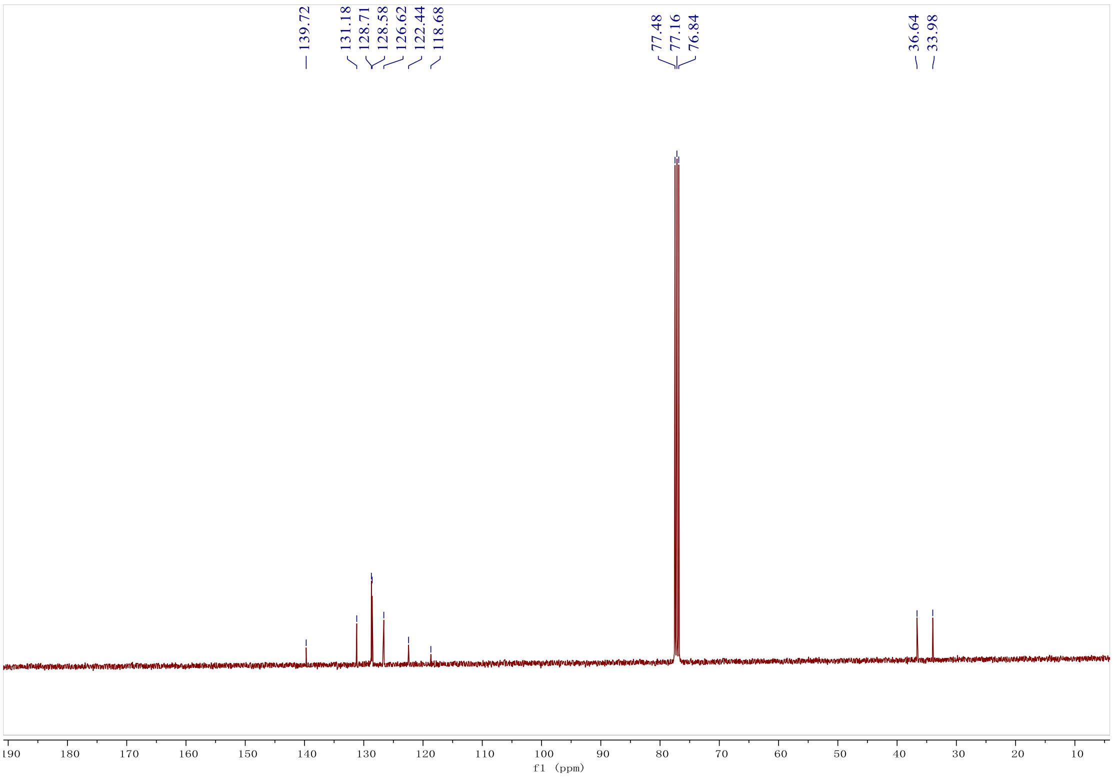
139.72
131.18
128.71
128.58
126.62
122.44
118.68
77.48
77.16
76.84
36.64
33.98
190 180 170 160 150 140 130 120 110 100 90 80 70 60 50 40 30 20 10
f1 (ppm)

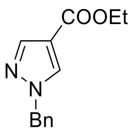
COOEt
N
N
Bn

11

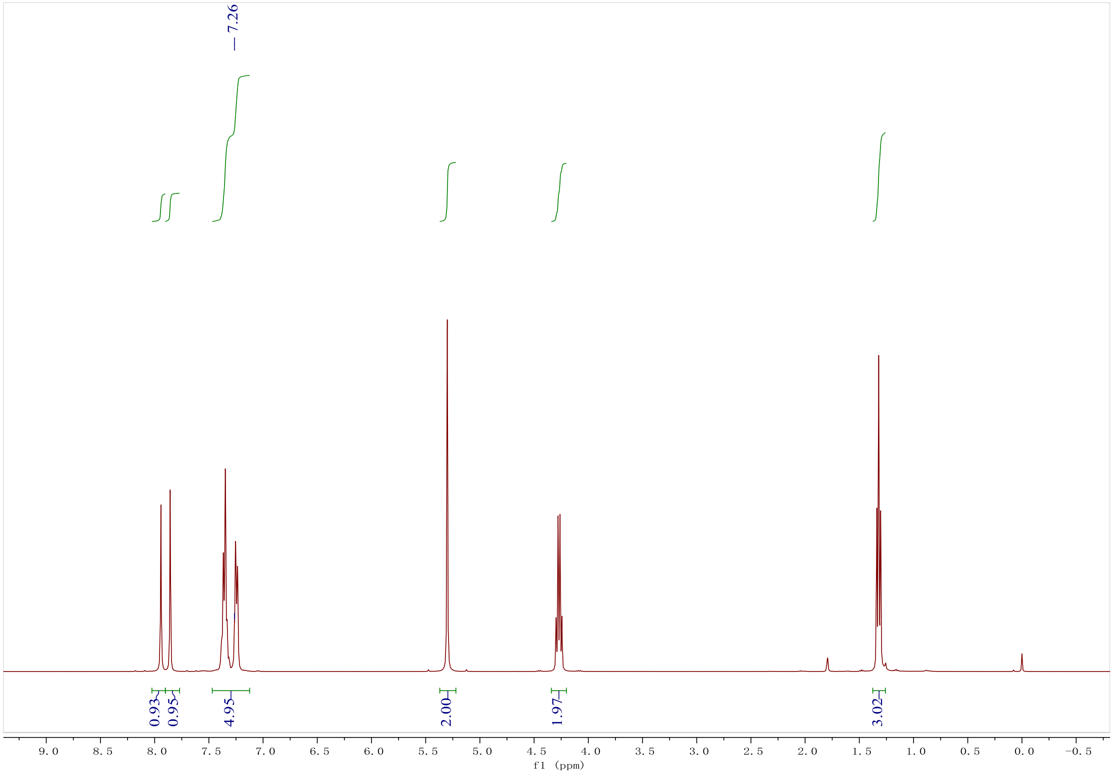
7.26
0.93
0.95
4.95
2.00
1.97
3.02
9.0 8.5 8.0 7.5 7.0 6.5 6.0 5.5 5.0 4.5 4.0 3.5 3.0 2.5 2.0 1.5 1.0 0.5 0.0 -0.5
f1 (ppm)

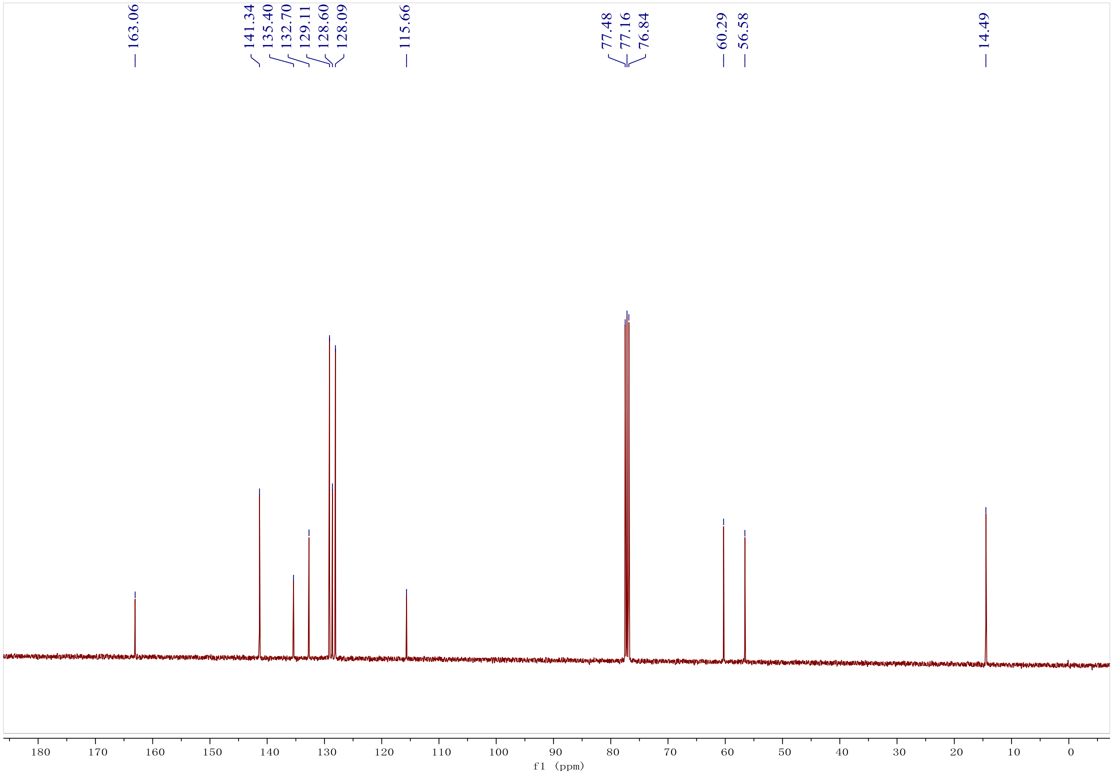
163.06
141.34
135.40
132.70
129.11
128.60
128.09
115.66
77.48
77.16
76.84
60.29
56.58
14.49
180 170 160 150 140 130 120 110 100 90 80 70 60 50 40 30 20 10 0
f1 (ppm)

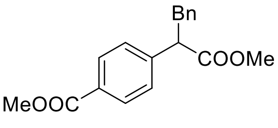
Bn
COOMe
MeOOC

12

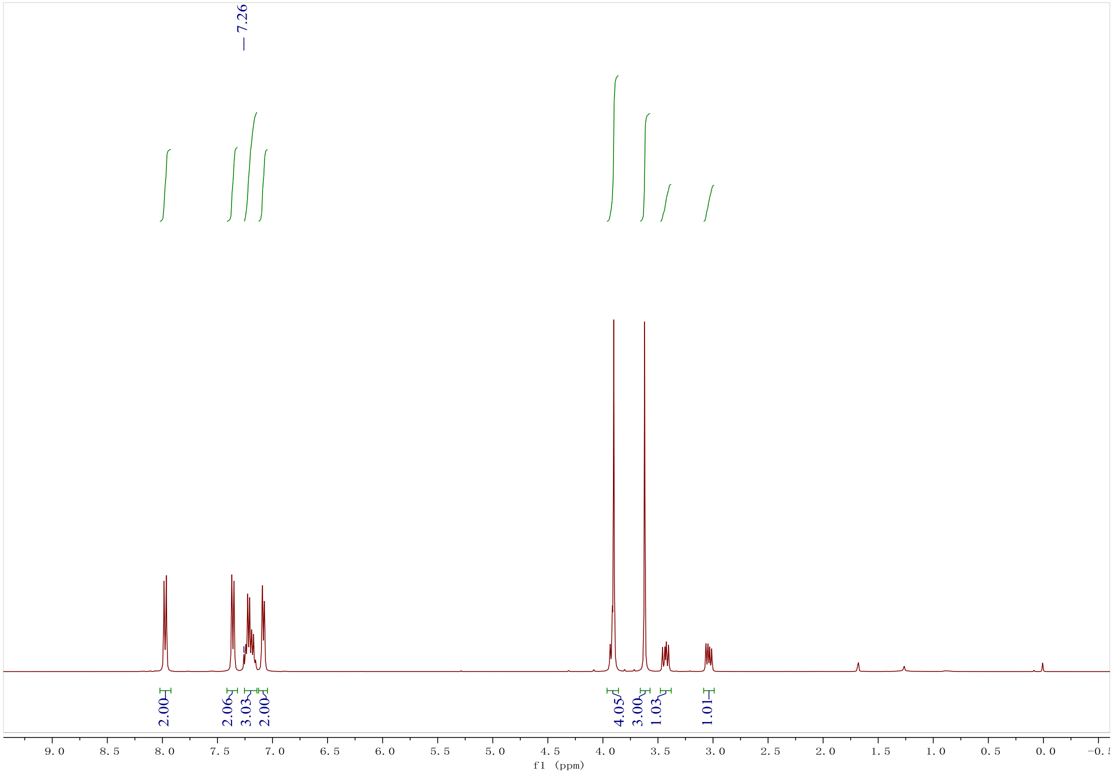
7.26
2.00
2.06
3.03
2.00
4.05
3.00
1.03
1.01
f1 (ppm)

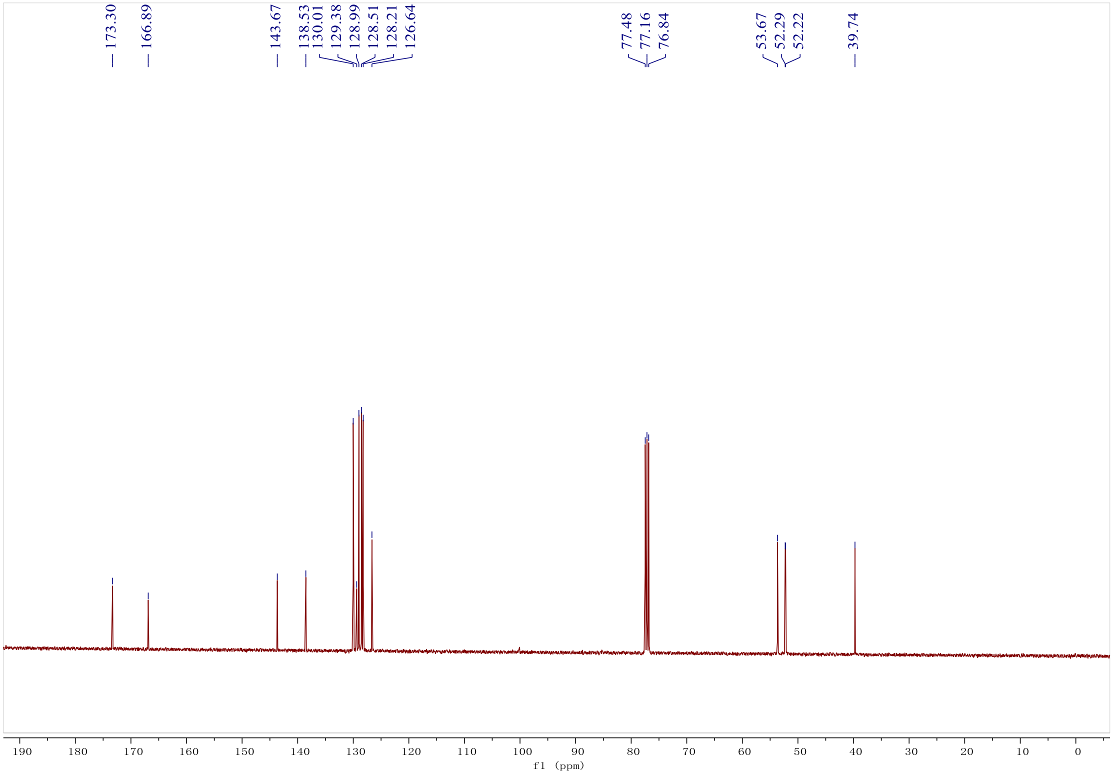
173.30
166.89
143.67
138.53
130.01
129.38
128.99
128.51
128.21
126.64
77.48
77.16
76.84
53.67
52.29
52.22
39.74
f1 (ppm)

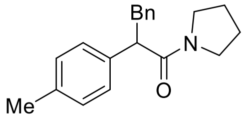
Bn
N
O
Me

13

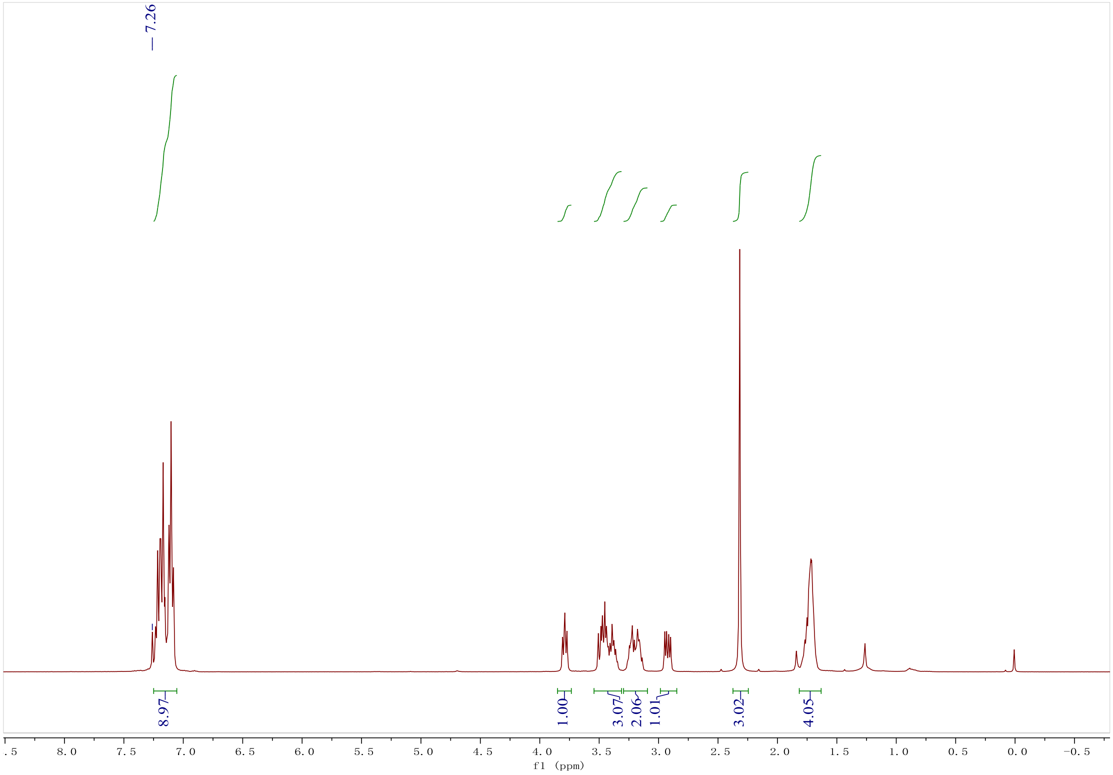
7.26
8.97
1.00
3.07
2.06
1.01
3.02
4.05
f1 (ppm)

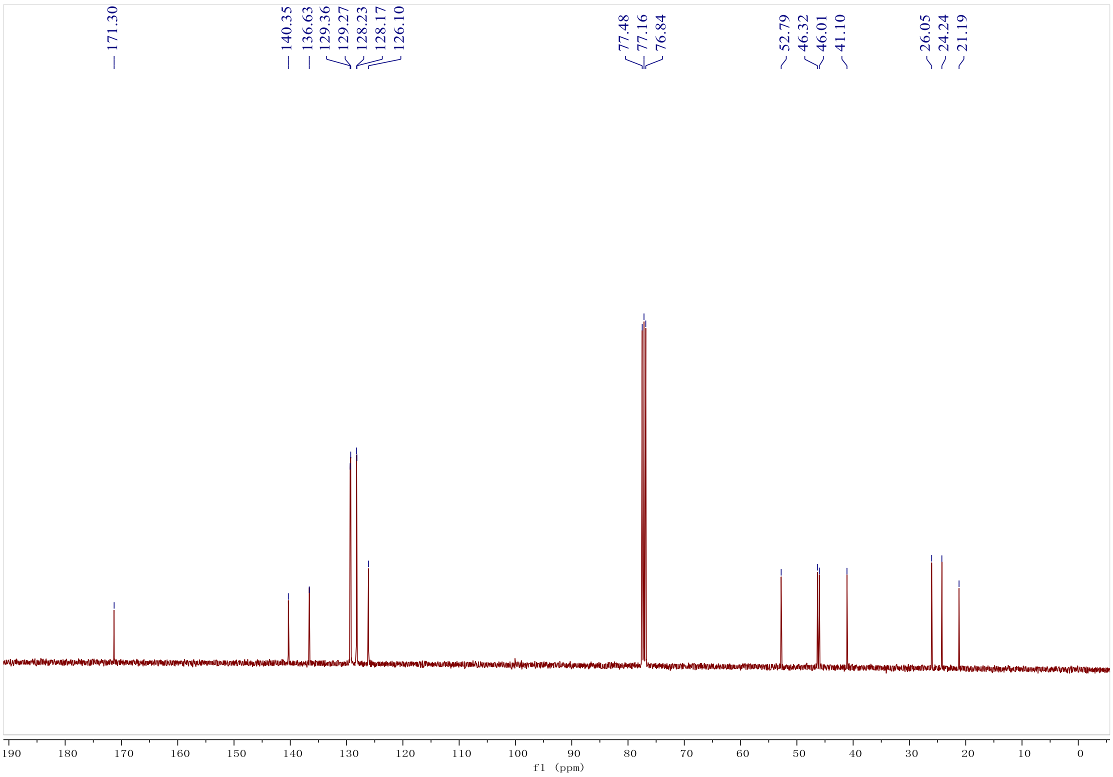
171.30
140.35
136.63
129.36
129.27
128.23
128.17
126.10
77.48
77.16
76.84
52.79
46.32
46.01
41.10
26.05
24.24
21.19
f1 (ppm)

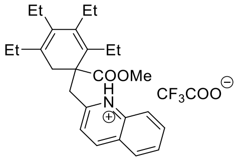
Et
Et
Et
Et
COOMe
H
N
CF3COO

14

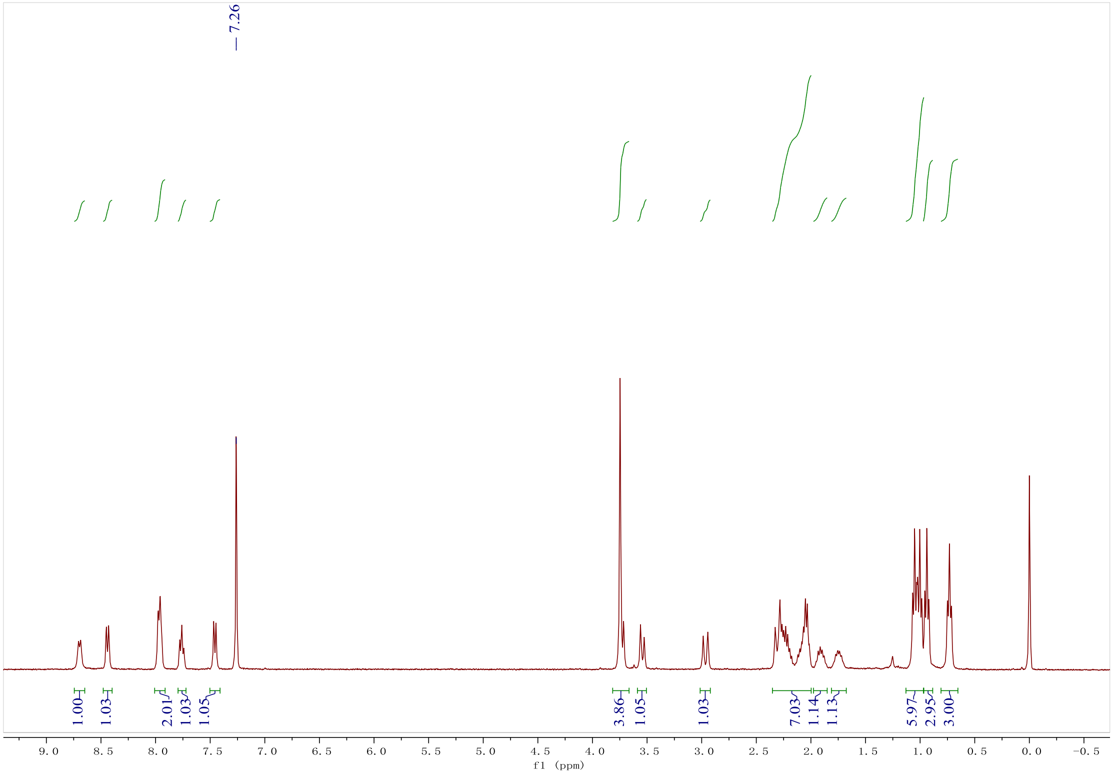
7.26
1.00
1.03
2.01
1.03
1.05
3.86
1.05
1.03
7.03
1.14
1.13
5.97
2.95
3.00
9.0 8.5 8.0 7.5 7.0 6.5 6.0 5.5 5.0 4.5 4.0 3.5 3.0 2.5 2.0 1.5 1.0 0.5 0.0 -0.5
f1 (ppm)

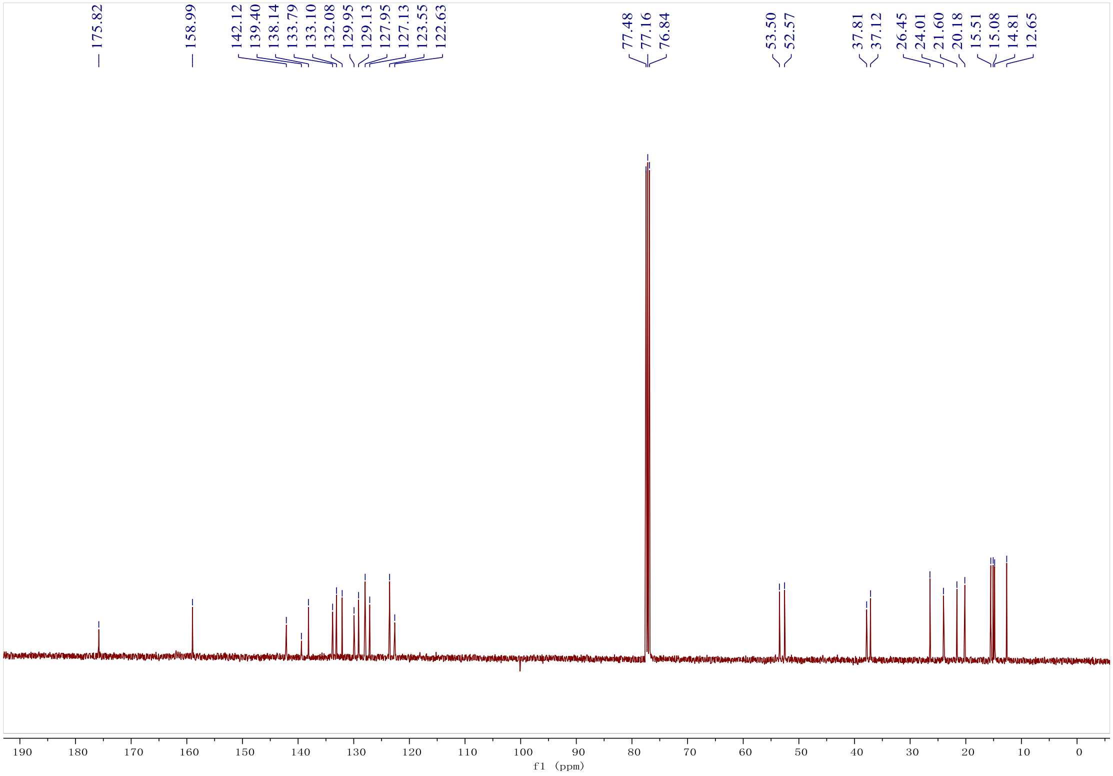
175.82
158.99
142.12
139.40
138.14
133.79
133.10
132.08
129.95
129.13
127.95
127.13
123.55
122.63
77.48
77.16
76.84
53.50
52.57
37.81
37.12
26.45
24.01
21.60
20.18
15.51
15.08
14.81
12.65
190 180 170 160 150 140 130 120 110 100 90 80 70 60 50 40 30 20 10 0
f1 (ppm)

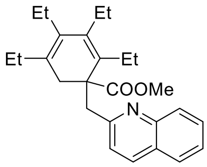

Et
Et
Et
Et
COOMe
N

S1

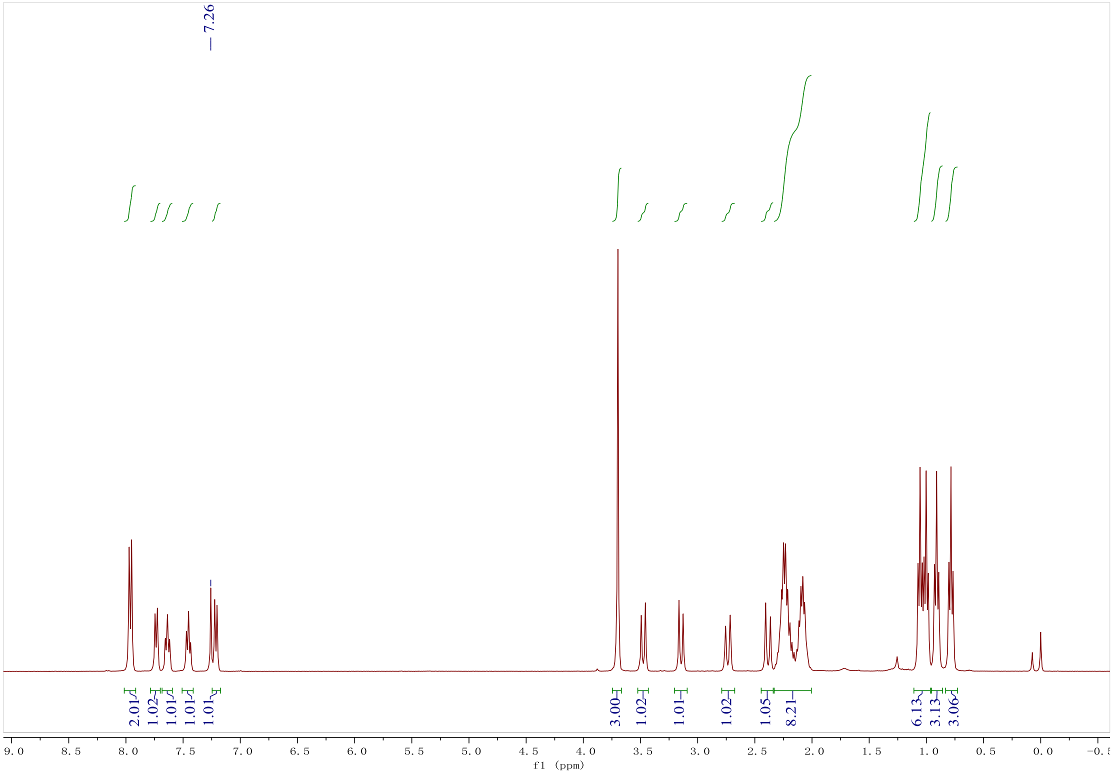

7.26
2.01
1.02
1.01
1.01
1.01
3.00
1.02
1.01
1.02
1.05
8.21
6.13
3.13
3.06
f1 (ppm)

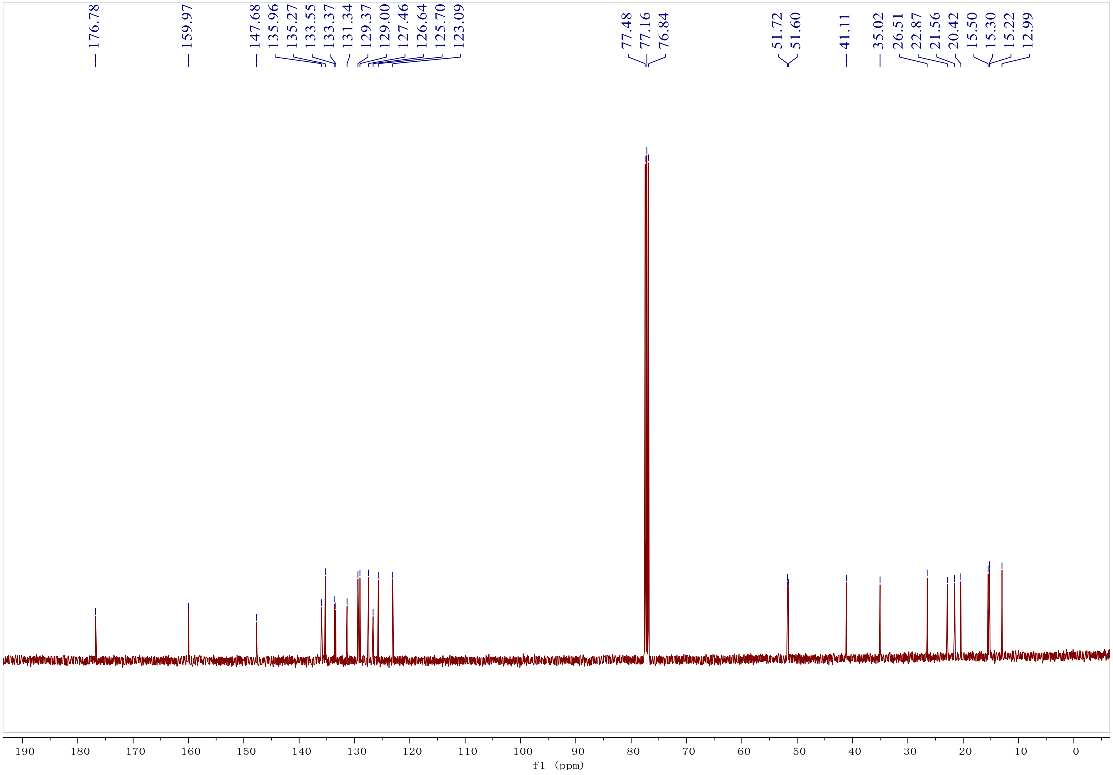

176.78
159.97
147.68
135.96
135.27
133.55
133.37
131.34
129.37
129.00
127.46
126.64
125.70
123.09
77.48
77.16
76.84
51.72
51.60
41.11
35.02
26.51
22.87
21.56
20.42
15.50
15.30
15.22
12.99
f1 (ppm)
